# Supplementary material for: Ammonium Dinitramide as a Prospective N–NO2 Synthon: Electrochemical Synthesis of Nitro-NNO-Azoxy Compounds from Nitrosoarenes
Source: Molecules. 2024 Nov 25;29(23):5563. doi: 10.3390/molecules29235563 (PMC11643399; doi:10.3390/molecules29235563)
Supplement: Supplementary file 1 [file molecules-29-05563-s001.zip › 2024-11-22 ESI_Molecules.pdf]

*Supporting Information for*

# Ammonium Dinitramide as a Perspective N–$NO_2$ Synthon: Electrochemical Synthesis of Nitro-*NNO*-azoxy Compounds from Nitrosoarenes

Alexander S. Budnikov,[a] Nikita E. Leonov,[a] Michael S. Klenov,*[a] Mikhail I. Shevchenko,[a] Tatiana Y. Dvinyaninova,[a] Igor B. Krylov,*[a] Aleksandr M. Churakov,[a] Ivan V. Fedyanin,[b] Vladimir A. Tartakovsky[a] and Alexander O. Terent'ev[a]

[a] N. D. Zelinsky Institute of Organic Chemistry of the Russian Academy of Sciences, 47 Leninsky prosp., 119991 Moscow, Russian Federation

[b] A. N. Nesmeyanov Institute of Organoelement Compounds of the Russian Academy of Sciences, 28 Vavilova Str., 119991 Moscow, Russian Federation

E-mail: krylovigor@yandex.ru (I.B.K.); klenov@ioc.ac.ru (M.S.K.)

## Table of contents

1. General information ........ S6

2. Experimental data ........ S7

2.1 General Procedure for the Optimization of the Reaction Conditions for the Synthesis of (Nitro-*NNO*-azoxy)benzene (2a) from Nitrosobenzene (1a) (Experimental details for Table 1) ........ S7

2.2 Typical Procedure for Electrochemical Synthesis of Nitro-*NNO*-Azoxy Benzenes 2a–2t (Experimental details for Scheme 2) ........ S9

2.3 Procedure for Gram Scale Electrochemical Synthesis of (Nitro-*NNO*-Azoxy)benzene (2a) (Experimental details for Scheme 3). ........ S9

2.4 Reaction in Divided Electrochemical Cell (Experimental details for Scheme 4a) ........ S9

2.5 Potential Monitoring During Electrolysis (Experimental details for Scheme 4b). ........ S10

2.6 Reaction under Constant Potential Electrolysis (Experimental details for Scheme 4c) ........ S10

2.7 Study of ADN Constant Current Electrolysis in an Undivided and Divided Electrochemical Cell (Experimental details for Scheme 4d) ........ S11

2.8. Cyclic Voltammetry Studies ........ S11

3. Characterization of Products ........ S13

4. Pictures of the Equipment Used ........ S20

5. Fungicidal activity tests (experimental details for Table 2). ........ S20

6. References ........ S22

7. NMR Data ........ S25

7.1.1 $^{1}H$ NMR spectrum of compound 2a [500.13 MHz, $CDCl_3$] ........ S25

7.1.2 $^{13}C$ NMR spectrum of compound 2a [125.76 MHz, $CDCl_3$] ........ S26

7.1.3 {$^{1}H$–$^{13}C$} HSQC spectrum of compound 2a [500.13 MHz, $CDCl_3$] ........ S27

7.1.4 {$^{1}H$–$^{13}C$} HMBC spectrum of compound 2a [500.13 MHz, $CDCl_3$] ........ S28

7.1.5 $^{14}N$ NMR spectrum of compound 2a [36.14 MHz, $CDCl_3$] ........ S29

7.2.1 $^{1}H$ NMR spectrum of compound 2b [500.13 MHz, $CDCl_3$] ........ S30

7.2.2 $^{13}C$ NMR spectrum of compound 2b [125.76 MHz, $CDCl_3$] ........ S31

7.2.3 {$^{1}H$–$^{13}C$} HSQC spectrum of compound 2b [500.13 MHz, $CDCl_3$] ........ S32

7.2.4 {$^{1}H$–$^{13}C$} HMBC spectrum of compound 2b [500.13 MHz, $CDCl_3$] ........ S33

7.2.5 $^{14}N$ NMR spectrum of compound 2b [36.14 MHz, $CDCl_3$] ........ S34

7.3.1 $^{1}H$ NMR spectrum of compound 2c [500.13 MHz, $CDCl_3$] ........ S35

7.3.2 $^{13}C$ NMR spectrum of compound 2c [125.76 MHz, $CDCl_3$] ........ S36

7.3.3 {$^{1}H$–$^{13}C$} HSQC spectrum of compound 2c [500.13 MHz, $CDCl_3$] ........ S37

7.3.4 {$^{1}H$–$^{13}C$} HMBC spectrum of compound 2c [500.13 MHz, $CDCl_3$] ........ S38

7.3.5 $^{14}N$ NMR spectrum of compound 2c [36.14 MHz, $CDCl_3$] ........ S39

7.4.1 $^{1}H$ NMR spectrum of compound 2d [600.13 MHz, $CDCl_3$] ........ S40

7.4.2 $^{13}C$ NMR spectrum of compound 2d [150.90 MHz, $CDCl_3$] ........ S41

7.4.3 {$^{1}H$–$^{13}C$} HSQC spectrum of compound 2d [600.13 MHz, $CDCl_3$] ........ S42

7.4.4 {$^1$H–$^{13}$C} HMBC spectrum of compound 2d [600.13 MHz, $CDCl_3$] ....S43
7.4.5 $^{14}$N NMR spectrum of compound 2d [43.37 MHz, $CDCl_3$] ....S44
7.5.1 $^1$H NMR spectrum of compound 2e [500.13 MHz, $CDCl_3$] ....S45
7.5.2 $^{13}$C NMR spectrum of compound 2e [125.76 MHz, $CDCl_3$] ....S46
7.5.3 {$^1$H–$^{13}$C} HSQC spectrum of compound 2e [500.13 MHz, $CDCl_3$] ....S47
7.5.4 {$^1$H–$^{13}$C} HMBC spectrum of compound 2e [500.13 MHz, $CDCl_3$] ....S48
7.5.5 $^{14}$N NMR spectrum of compound 2e [36.14 MHz, $CDCl_3$] ....S49
7.6.1 $^1$H NMR spectrum of compound 2f [600.13 MHz, $CDCl_3$] ....S50
7.6.2 $^{13}$C NMR spectrum of compound 2f [150.90 MHz, $CDCl_3$] ....S51
7.6.3 {$^1$H–$^{13}$C} HSQC spectrum of compound 2f [600.13 MHz, $CDCl_3$] ....S52
7.6.4 {$^1$H–$^{13}$C} HMBC spectrum of compound 2f [600.13 MHz, $CDCl_3$] ....S53
7.6.5 $^{14}$N NMR spectrum of compound 2f [43.37 MHz, $CDCl_3$] ....S54
7.7.1 $^1$H NMR spectrum of compound 2g [600.13 MHz, $CDCl_3$] ....S55
7.7.2 $^{13}$C NMR spectrum of compound 2g [150.90 MHz, $CDCl_3$] ....S56
7.7.3 {$^1$H–$^{13}$C} HSQC spectrum of compound 2g [600.13 MHz, $CDCl_3$] ....S57
7.7.4 {$^1$H–$^{13}$C} HMBC spectrum of compound 2g [600.13 MHz, $CDCl_3$] ....S58
7.7.5 $^{14}$N NMR spectrum of compound 2g [43.14 MHz, $CDCl_3$] ....S59
7.8.1 $^1$H NMR spectrum of compound 2i [600.13 MHz, $CDCl_3$] ....S60
7.8.2 $^{13}$C NMR spectrum of compound 2i [150.90 MHz, $CDCl_3$] ....S61
7.8.3 {$^1$H–$^{13}$C} HSQC spectrum of compound 2i [600.13 MHz, $CDCl_3$] ....S62
7.8.4 {$^1$H–$^{13}$C} HMBC spectrum of compound 2i [600.13 MHz, $CDCl_3$] ....S63
7.8.5 $^{14}$N NMR spectrum of compound 2i [43.37 MHz, $CDCl_3$] ....S64
7.9.1 $^1$H NMR spectrum of compound 2j [600.13 MHz, $CDCl_3$] ....S65
7.9.2 $^{13}$C NMR spectrum of compound 2j [150.90 MHz, $CDCl_3$] ....S66
7.9.3 {$^1$H–$^{13}$C} HSQC spectrum of compound 2j [600.13 MHz, $CDCl_3$] ....S67
7.9.4 {$^1$H–$^{13}$C} HMBC spectrum of compound 2j [600.13 MHz, $CDCl_3$] ....S68
7.9.5 $^{14}$N NMR spectrum of compound 2j [43.37 MHz, $CDCl_3$] ....S69
7.10.1 $^1$H NMR spectrum of compound 2k [300.13 MHz, $CDCl_3$] ....S70
7.10.2 $^{13}$C NMR spectrum of compound 2k [300.13 MHz, $CDCl_3$] ....S71
7.10.3 {$^1$H–$^{13}$C} HSQC spectrum of compound 2k [300.13 MHz, $CDCl_3$] ....S72
7.10.4 {$^1$H–$^{13}$C} HMBC spectrum of compound 2k [300.13 MHz, $CDCl_3$] ....S73
7.10.5 $^{14}$N NMR spectrum of compound 2k [43.37 MHz, $CDCl_3$] ....S74
7.11.1 $^1$H NMR spectrum of the mixture of compound 2l and 4-fluoro-1-nitrobenzene (1.5 : 1 molar ratio) [600.13 MHz, $CDCl_3$] ....S75
7.11.2 $^{13}$C NMR spectrum of the mixture of compound 2l and 4-fluoro-1-nitrobenzene (1.5 : 1 molar ratio) [150.90 MHz, $CDCl_3$] ....S76
7.11.3 {$^1$H–$^{13}$C} HSQC spectrum of the mixture of compound 2l and 4-fluoro-1-nitrobenzene [600.13 MHz, $CDCl_3$] ....S77

7.11.4 {$^1$H–$^{13}$C} HMBC spectrum of the mixture of compound 2l and 4-fluoro-1-nitrobenzene [600.13 MHz, $CDCl_3$] ........S78

7.11.5 $^{14}$N NMR spectrum of the mixture of compound 2l and 4-fluoro-1-nitrobenzene [43.37 MHz, $CDCl_3$] ........S79

7.12.1 $^1$H NMR spectrum of compound 2m [600.13 MHz, $CDCl_3$] ........S80

7.12.2 $^{13}$C NMR spectrum of compound 2m [150.90 MHz, $CDCl_3$] ........S81

7.12.3 {$^1$H–$^{13}$C} HSQC spectrum of compound 2m [600.13 MHz, $CDCl_3$] ........S82

7.12.4 {$^1$H–$^{13}$C} HMBC spectrum of compound 2m [600.13 MHz, $CDCl_3$] ........S83

7.12.5 $^{14}$N NMR spectrum of compound 2m [43.37 MHz, $CDCl_3$] ........S84

7.13.1 $^1$H NMR spectrum of compound 2n [500.13 MHz, $CDCl_3$] ........S85

7.13.2 $^{13}$C NMR spectrum of compound 2n [125.76 MHz, $CDCl_3$] ........S86

7.13.3 {$^1$H–$^{13}$C} HSQC spectrum of compound 2n [500.13 MHz, $CDCl_3$] ........S87

7.13.4 {$^1$H–$^{13}$C} HMBC spectrum of compound 2n [500.13 MHz, $CDCl_3$] ........S88

7.13.5 $^{14}$N NMR spectrum of compound 2n [36.14 MHz, $CDCl_3$] ........S89

7.14.1 $^1$H NMR spectrum of compound 2o [300.13 MHz, $CDCl_3$] ........S90

7.14.2 $^{13}$C NMR spectrum of compound 2o [75.49 MHz, $CDCl_3$] ........S91

7.14.3 {$^1$H–$^{13}$C} HSQC spectrum of compound 2o [300.13 MHz, $CDCl_3$] ........S92

7.14.4 {$^1$H–$^{13}$C} HMBC spectrum of compound 2o [300.13 MHz, $CDCl_3$] ........S93

7.14.5 $^{14}$N NMR spectrum of compound 2o [36.14 MHz, $CDCl_3$] ........S94

7.15.1 $^1$H NMR spectrum of compound 2p [500.13 MHz, $CDCl_3$] ........S95

7.15.2 $^{13}$C NMR spectrum of compound 2p [125.76 MHz, $CDCl_3$] ........S96

7.15.3 {$^1$H–$^{13}$C} HSQC spectrum of compound 2p [500.13 MHz, $CDCl_3$] ........S97

7.15.4 {$^1$H–$^{13}$C} HMBC spectrum of compound 2p [500.13 MHz, $CDCl_3$] ........S98

7.15.5 $^{14}$N NMR spectrum of compound 2p [36.14 MHz, $CDCl_3$] ........S99

7.16.1 $^1$H NMR spectrum of compound 2q [600.13 MHz, $CDCl_3$] ........S100

7.16.2 $^{13}$C NMR spectrum of compound 2q [150.90 MHz, $CDCl_3$] ........S101

7.16.3 {$^1$H–$^{13}$C} HSQC spectrum of compound 2q [600.13 MHz, $CDCl_3$] ........S102

7.16.4 {$^1$H–$^{13}$C} HMBC spectrum of compound 2q [600.13 MHz, $CDCl_3$] ........S103

7.16.5 $^{14}$N NMR spectrum of compound 2q [43.37 MHz, $CDCl_3$] ........S104

7.17.1 $^1$H NMR spectrum of compound 2r [600.13 MHz, $CDCl_3$] ........S105

7.17.2 $^{13}$C NMR spectrum of compound 2r [150.90 MHz, $CDCl_3$] ........S106

7.17.3 {$^1$H–$^{13}$C} HSQC spectrum of compound 2r [600.13 MHz, $CDCl_3$] ........S107

7.17.4 {$^1$H–$^{13}$C} HMBC spectrum of compound 2r [600.13 MHz, $CDCl_3$] ........S108

7.17.5 $^{14}$N NMR spectrum of compound 2r [36.14 MHz, $CDCl_3$] ........S109

7.18.1 $^1$H NMR spectrum of compound 2s [500.13 MHz, $CDCl_3$] ........S110

7.18.2 $^{13}$C NMR spectrum of compound 2s [125.76 MHz, $CDCl_3$] ........S111

7.18.3 {$^1$H–$^{13}$C} HSQC spectrum of compound 2s [500.13 MHz, $CDCl_3$] ........S112

7.18.4 {$^{1}$H–$^{13}$C} HMBC spectrum of compound 2s [500.13 MHz, $CDCl_3$] ........................................ S113
7.18.5 $^{14}$N NMR spectrum of compound 2s [36.14 MHz, $CDCl_3$] ........................................ S114
7.19.1 $^{1}$H NMR spectrum of compound 2t [500.13 MHz, $CDCl_3$] ........................................ S115
7.19.2 $^{13}$C NMR spectrum of compound 2t [125.76 MHz, $CDCl_3$] ........................................ S116
7.19.3 {$^{1}$H–$^{13}$C} HSQC spectrum of compound 2t [500.13 MHz, $CDCl_3$] ........................................ S117
7.19.4 {$^{1}$H–$^{13}$C} HMBC spectrum of compound 2t [500.13 MHz, $CDCl_3$] ........................................ S118
7.19.5 $^{14}$N NMR spectrum of compound 2t [36.14 MHz, $CDCl_3$] ........................................ S119
8. X-ray diffraction analysis ........................................ S120

# 1. General information

$^{1}$H, $^{13}$C, $^{14}$N and $^{15}$N NMR spectra were recorded with Bruker DRX-500 (500.1, 125.8, 36.1, 50.7 MHz, respectively) and Bruker AV600 (600.1, 150.9, 43.4, 60.8 MHz, respectively) spectrometers. Chemical shifts are reported in delta (*δ*) units, parts per million (ppm) downfield from internal TMS ($^{1}$H, $^{13}$C) or external $CH_3NO_2$ ($^{14}$N, $^{15}$N negative values of $δ_N$ correspond to upfield shifts). The IR spectra were recorded with a Bruker ALPHA-T spectrometer in the range 400–4000 $cm^{–1}$ (resolution 2 $cm^{–1}$) as pellets with KBr or as a thin layer. High-resolution ESI mass spectra (HRMS) were recorded with a Bruker micrOTOF II instrument. Silica gel 60 Merck (15–40 μm) was used for preparative column and thin-layer chromatography. Silica gel "Silpearl UV 254" was used for preparative column and thin-layer chromatography. Analytical thin-layer chromatography (TLC) was carried out on Merck silica gel 60 F254 and "Silufol" TLC silica gel UV-254 aluminum sheets. All reagents were purchased from Acros and Sigma-Aldrich. Solvents were purified before use, according to standard procedures. Solvents were purified before use, according to standard procedures. MeCN was distilled over $CaH_2$ and then over $P_2O_5$, acetone was distilled over $KMnO_4$, MeOH was distilled over magnesium and iodine, and DMF was distilled over $CaH_2$. 1,1,1,3,3,3-hexafluoro-2-propanol was used as is from commercial sources. All other reagents were used without further purification. Ammonium dinitramide,[1] nitrosobenzene (**1a**),[2] 1-methyl-4-nitrosobenzene (**1b**),[3] 1-methyl-3-nitrosobenzene (**1c**),[4] 1-methyl-2-nitrosobenzene (**1d**),[5] 1-nitro-4-nitrosobenzene (**1e**),[6] 1-nitro-3-nitrosobenzene (**1f**),[7] 1-nitro-2-nitrosobenzene (**1g**),[8] 1-methoxy-4-nitrosobenzene (**1i**),[9] 1-methoxy-3-nitrosobenzene (**1j**),[10] 1-methoxy-2-nitrosobenzene (**1k**),[11] 1-fluoro-4-nitrosobenzene (**1l**),[12] 1-chloro-4-nitrosobenzene (**1m**),[4] 2,4-dichloro-1-nitrosobenzene (**1n**),[13] 1,3-dichloro-2-nitrosobenzene (**1o**),[14] 1,3,5-trichloro-2-nitrosobenzene (**1p**),[14] 1-bromo-4-nitrosobenzene (**1q**),[15] 2,4-dibromo-1-nitrosobenzene (**1r**),[16] 1,3-dibromo-1-nitrosobenzene (**1s**)[14] and 1,3,5-tribromo-1-nitrosobenzene (**1t**)[14] were prepared according to the reported procedures.

## 2. Experimental data

### 2.1 General Procedure for the Optimization of the Reaction Conditions for the Synthesis of (Nitro-*NNO*-azoxy)benzene (2a) from Nitrosobenzene (1a) (Experimental details for Table 1)

An undivided 20 mL jacketed electrochemical cell was equipped with platinum, stainless steel, nickel, graphite, carbon felt, or glassy carbon plate anode (30 × 15 $mm^2$) and a platinum, stainless steel, nickel, graphite, glassy carbon plate (30 × 15 $mm^2$), or platinum wire anode, and connected to a DC regulated power supply. Electrodes were completely immersed in the solution given S = 4.5 $cm^2$ of working surface. A solution of nitrosobenzene (**1a**) (1.0 mmol, 107 mg), and ammonium dinitramide (ADN) (1–5 equiv., 1–5 mmol, 124–620 mg) in 20 mL of MeCN, acetone, DMF, MeOH or HFIP was electrolyzed using constant current conditions (*I* = 15–240 mA) at 23–25 °C under magnetic stirring. After passing 1–3 F·$mol^{-1}$ of electricity (reaction time 14–322 min), electrodes were washed with $CH_2Cl_2$ (3 × 20 mL). The combined organic phase was washed with $H_2O$ (20 mL) and brine (20 mL), dried over $Na_2SO_4$, and solvent removed in vacuo. The yields of **2a** were determined with the use of $^{1}H$ NMR spectroscopy using 2-methyl-2-nitropropane as an internal standard and checked by $^{14}N$ NMR. In the run 1 (nitro-*NNO*-azoxy)benzene (**2a**) was purified by preparative column chromatography on silica gel ($R_f$ = 0.51, petroleum ether/ethyl acetate, 10:1) to give compound **2a** (142 mg, 85%) as a colorless solid. mp: 83–85 °C. The product obtained was identical (TLC, $^{1}H$, $^{13}C$, $^{14}N$ NMR) to compound prepared according to the reported procedure.[17]

**Table S1.** Optimization of the Reaction Conditions of Nitro-*NNO*-Azoxylation of Nitrosobenzene (**1a**) with ADN[a].

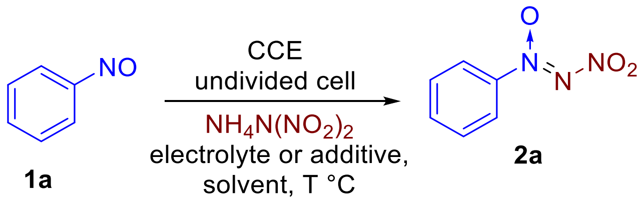


| Run | Solvent | Molar ratio **1a**: ADN | Electrodes (+/–) | *F* [F/mol of **1a**] | Yield, **2a**[b] |
|---|---|---|---|---|---|
| 1[c] | MeCN | 1:1 | Pt/$Pt_w$ | 2 | 11 |
| 2 | MeCN | 1:2 | Pt/$Pt_w$ | 2 | 43 |
| 3 | MeCN | 1:3 | Pt/$Pt_w$ | 2 | 49 |
| 4 | MeCN | 1:4 | Pt/$Pt_w$ | 2 | 72 |
| 5 | MeCN | 1:5 | Pt/$Pt_w$ | 2 | 75 |

| | | | | | |
|---|---|---|---|---|---|
| 6[d] | MeCN | 1:4 | Pt/$Pt_W$ | 2 | 68 |
| 7[e] | MeCN | 1:4 | Pt/$Pt_W$ | 2 | 26 |
| 8[f] | MeCN | 1:4 | Pt/$Pt_W$ | 2 | 34 |
| 9 | acetone | 1:4 | Pt/$Pt_W$ | 2 | 9 |
| 10 | DMF | 1:4 | Pt/$Pt_W$ | 2 | n.d. |
| 11 | MeOH | 1:4 | Pt/$Pt_W$ | 2 | n.d. |
| 12 | HFIP | 1:4 | Pt/$Pt_W$ | 2 | 54 |
| 13[g] | MeCN | 1:4 | Pt/$Pt_W$ | 2 | 73 |
| 14[h] | MeCN | 1:4 | Pt/$Pt_W$ | 2 | 67 |
| 15 | MeCN | 1:4 | Pt/$Pt_W$ | 1 | 45 |
| 16 | MeCN | 1:4 | Pt/$Pt_W$ | 1.5 | 54 |
| 17 | MeCN | 1:4 | Pt/$Pt_W$ | 3 | 77 |
| 18 | MeCN | 1:4 | Pt/$Pt_W$ | 2 | 81 |
| 19 | MeCN | 1:4 | Pt/$Pt_W$ | 2 | 76 |
| 20 | MeCN | 1:4 | Pt/$Pt_W$ | 2 | 66 |
| **21** | **MeCN** | **1:4** | **$C_F$/$Pt_W$** | **2** | **85 (70)** |
| 22 | MeCN | 1:4 | GC/$Pt_W$ | 2 | 76 |
| 23 | MeCN | 1:4 | C/$Pt_W$ | 2 | 60 |
| 24 | MeCN | 1:4 | Ni/$Pt_W$ | 2 | 50 |
| 25 | MeCN | 1:4 | SS/$Pt_W$ | 2 | 38 |
| 26 | MeCN | 1:4 | $C_F$/$C_F$ | 2 | <5 |
| 27 | MeCN | 1:4 | $C_F$/Ni | 2 | 27 |
| 28 | MeCN | 1:4 | $C_F$/SS | 2 | 52 |
| 29 | MeCN | 1:4 | $C_F$/GC | 2 | 49 |
| 30 | MeCN | 1:4 | $C_F$/C | 2 | <5 |
| 31 | MeCN | 1:4 | $C_F$/$Pt_W$ | 2 | 84 |
| 32 | MeCN | 1:4 | $C_F$/$Pt_W$ | 2 | 80 |
| 33[i] | MeCN | 1:4 | - | - | n.d. |

[a]**Reaction conditions**: nitrosobenzene **1a** (107 mg, 1.0 mmol), ADN (124–620 mg, 1–5 mmol), electrolyte or additive (329–500 mg), solvent (20 mL), undivided cell, constant current electrolysis with *I* = 15–240 mA, *F* = 1–3 F·mol$^{-1}$ **1a** (reaction time 13–322 min), 0–40 °C, air atmosphere. C – graphite plate, GC – glassy carbon plate, $C_F$ – carbon felt, Pt – platinum plate, $Pt_W$ – platinum wire, SS – stainless steel plate, Ni – nickel plate. Unless otherwise stated, all electrodes were 30×15 mm$^2$ and were completely immersed in the solution given S = 4.5 cm$^2$ of working surface. [b]The yield was determined by $^1$H NMR using 2-methyl-2-nitropropane as an internal standard and checked by $^{14}$N NMR; the isolated yields are given in parentheses. n. d. – not detected. The bold line of entry 20 indicates conditions as optimal. [c]*n*-$Bu_4NBF_4$ (1.0 mmol) was added as a supporting electrolyte. [d]Ar atmosphere. [e]$H_2O$ (0.5 mL) was added. [f]KDN (potassium dinitramide, 4.0 mmol) was used instead of ADN, *n*-$Bu_4NBF_4$ (1.0 mmol) was added as a supporting electrolyte. [g]0 °C. [h]40 °C. [i]Without electricity.

**2.2 Typical Procedure for Electrochemical Synthesis of Nitro-*NNO*-Azoxy Benzenes 2a–2t (Experimental details for Scheme 2)**

An undivided 20 mL jacketed electrochemical cell was equipped with a carbon felt anode (30 × 15 mm$^2$) and a platinum wire cathode, and connected to a DC regulated power supply. Electrodes were completely immersed in the solution given S = 4.5 cm$^2$ of working anode surface. A mixture of nitrosobenzene **1a–1t** (1.0 mmol, 107–329 mg), and ADN (4.0 equiv., 4.0 mmol, 496 mg) in MeCN (20 mL) was electrolyzed using constant current conditions ($I$ = 60 mA) at 23–25 °C under magnetic stirring. After passing 2.0 F·mol$^{-1}$ of electricity (reaction time 54 min), electrodes were washed with $CH_2Cl_2$ (3 × 20 mL). The combined organic phase was washed with $H_2O$ (20 mL) and brine (20 mL), dried over $Na_2SO_4$, and solvent removed in vacuo. Products **2a–2t** were isolated by column chromatography on silica gel.

**2.3 Procedure for Gram Scale Electrochemical Synthesis of (Nitro-*NNO*-Azoxy)benzene (2a) (Experimental details for Scheme 3).**

An undivided 200 mL jacketed three-necked electrochemical cell was equipped with a cylindrical carbon felt anode (90 × 55 mm$^2$, S = 49.5 cm$^2$) and a platinum wire cathode placed inside the anode space, and connected to a DC regulated power supply. A solution of nitrosobenzene **1a** (10.0 mmol, 1.07 g) and ADN (4.0 equiv., 40.0 mmol, 4.96 g) in MeCN (200 mL) was electrolyzed using constant current conditions ($I$ = 660 mA) employing water-jet cooling (water temperature *ca*. 20 °C) to prevent reaction heating. After passing 2.0 F·mol$^{-1}$ of electricity (reaction time 49 min), electrodes were washed with $CH_2Cl_2$ (3 × 100 mL). The combined organic phase was washed with $H_2O$ (200 mL) and brine (200 mL), dried over $Na_2SO_4$, and solvent removed in vacuo. Product **2a** (1.086 g, 6.5 mmol, 65%) was isolated by column chromatography on silica gel ($R_f$ = 0.51, petroleum ether/ethyl acetate, 10:1).

**2.4 Reaction in Divided Electrochemical Cell (Experimental details for Scheme 4a)**

A divided H-type electrochemical cell (volume of each compartment – 30 mL, divided with DuPont Nafion® N-117 membrane) was equipped with a carbon felt anode (30 × 15 mm$^2$) and a platinum plate cathode (30 × 15 mm$^2$), and connected to a DC regulated power supply. Electrodes were completely immersed in the solution given S = 4.5 cm$^2$ of working surface. A solution of nitrosobenzene **1a** (1.0 mmol, 107 mg) and ADN (4.0 equiv., 4.0 mmol, 496 mg) in MeCN (20 mL) was placed in the anodic compartment of the cell, and a solution of ADN (4.0 mmol, 496 mg) in MeCN (20 mL) was placed in the

cathodic compartment of the cell. Solutions were electrolyzed using constant current conditions ($I$ = 60 mA) at 23–25 °C under magnetic stirring. After passing 2.0 F/mol of electricity (reaction time 54 min), electrodes were washed with $CH_2Cl_2$ (2 × 20 mL). The organic phases from anodic and cathodic compartments were separately evaporated under water-jet vacuum. The yield of **2a** was determined according to $^1$H NMR spectroscopy using 2-methyl-2-nitropropane as an internal standard and checked by $^{14}$N NMR.

### 2.5 Potential Monitoring During Electrolysis (Experimental details for Scheme 4b)

An undivided 20 mL jacketed electrochemical cell was equipped with a carbon felt anode (30 × 15 mm$^2$), a platinum wire cathode, and reference $Ag/AgNO_3$ electrode linked to the solution by a porous glass diaphragm, and connected to a computer-assisted potentiostat. Electrodes were completely immersed in the solution given S = 4.5 cm$^2$ of working anode surface. A mixture of nitrosobenzene **1a** (1.0 mmol, 107 mg), and ADN (4.0 equiv., 4.0 mmol, 496 mg) in MeCN (20 mL) was electrolyzed using constant current conditions ($I$ = 60 mA) at 23–25 °C under magnetic stirring. The observed electrochemical potential at anode was recorded as the reaction proceeded

### 2.6 Reaction under Constant Potential Electrolysis (Experimental details for Scheme 4c)

An undivided 20 mL jacketed electrochemical cell was equipped with a carbon felt anode (30×15 mm$^2$), a platinum wire cathode, and reference $Ag/AgNO_3$ electrode linked to the solution by a porous glass diaphragm, and connected to a computer-assisted potentiostat. Electrodes were completely immersed in the solution given S = 4.5 cm$^2$ of working anode surface. A mixture of nitrosobenzene **1a** (1.0 mmol, 107 mg), and ADN (4.0 equiv., 4.0 mmol, 496 mg) in MeCN (20 mL) was electrolyzed using constant potential conditions ($E_{cell}$ = 1.8 V vs. $Ag/AgNO_3$) at 23–25 °C under magnetic stirring. After passing 2.0 or 5.5 F/mol of electricity (reaction time was 1 h and 3 h respectively), electrodes were washed with $CH_2Cl_2$ (3 × 20 mL). The combined organic phase was washed with $H_2O$ (20 mL) and brine (20 mL), dried over $Na_2SO_4$, and solvent removed in vacuo. The yield of **2a** was determined according to $^1$H NMR spectroscopy using 2-methyl-2-nitropropane as an internal standard and checked by $^{14}$N NMR.

### 2.7 Study of ADN Constant Current Electrolysis in an Undivided and Divided Electrochemical Cell (Experimental details for Scheme 4d)

a) An undivided 20 mL jacketed electrochemical cell was equipped with a carbon felt anode (30×15 mm$^2$) and a platinum wire cathode, and connected to a DC regulated power supply. Electrodes were completely immersed in the solution given S = 4.5 cm$^2$ of working anode surface. A solution ADN (4.0 mmol, 496 mg) in MeCN (20 mL) was electrolyzed using constant current conditions ($I$ = 60 mA) at 23–25 °C under magnetic stirring. After passing 2×10$^{-3}$ F of electricity (reaction time 54 min), electrodes were washed with $CH_2Cl_2$ (3 × 20 mL). The solvent removed in vacuo, and the resulting reaction mixture was analyzed by $^{14}$N spectroscopy in $D_2O$.

a) A divided H-type electrochemical cell (volume of each compartment – 30 mL, divided with DuPont Nafion® N-117 membrane) was equipped with a carbon felt anode (30×15 mm$^2$) and a platinum plate cathode (30×15 mm$^2$), and connected to a DC regulated power supply. Electrodes were completely immersed in the solution given S = 4.5 cm$^2$ of working surface. Solutions of ADN (4.0 mmol, 496 mg) in MeCN (20 mL) were placed in the anodic and cathodic compartments of the cell. Solutions were electrolyzed using constant current conditions ($I$ = 60 mA) at 23–25 °C under magnetic stirring. After passing 2×10$^{-3}$ F of electricity (reaction time 54 min), electrodes were washed with $CH_2Cl_2$ (2 × 20 mL). The organic phases from anodic and cathodic compartments were separately evaporated under water-jet vacuum. The resulting reaction mixtures were analyzed by $^{14}$N spectroscopy in $D_2O$.

### 2.8. Cyclic Voltammetry Studies

Cyclic voltammetry (CV) was implemented on an IPC-Pro M computer-assisted potentiostat manufactured by «Econix» (scan rate error 1.0%; potential setting 0.25 mV; scan rate 100 mV·s$^{-1}$). The experiments were performed in a 10 mL five-neck glass conic electrochemical cell with a water jacket. CV curves were recorded using a three-electrode scheme. In a typical case, 5 mL of a solution was utilized. The working electrode was a disc glassy-carbon electrode (d = 3 mm). A platinum wire served as an auxiliary electrode. An $Ag/AgNO_3$ (0.1 M) in 0.1 M $n$-$Bu_4NBF_4$/MeCN electrode was used as the reference electrode and was linked to the solution by a porous glass diaphragm. The solutions were kept under thermally controlled conditions at 21 ± 0.5 °C and deaerated by bubbling argon. Electrochemical experiments were performed under an argon atmosphere. The working electrode was polished before recording each CV curve.

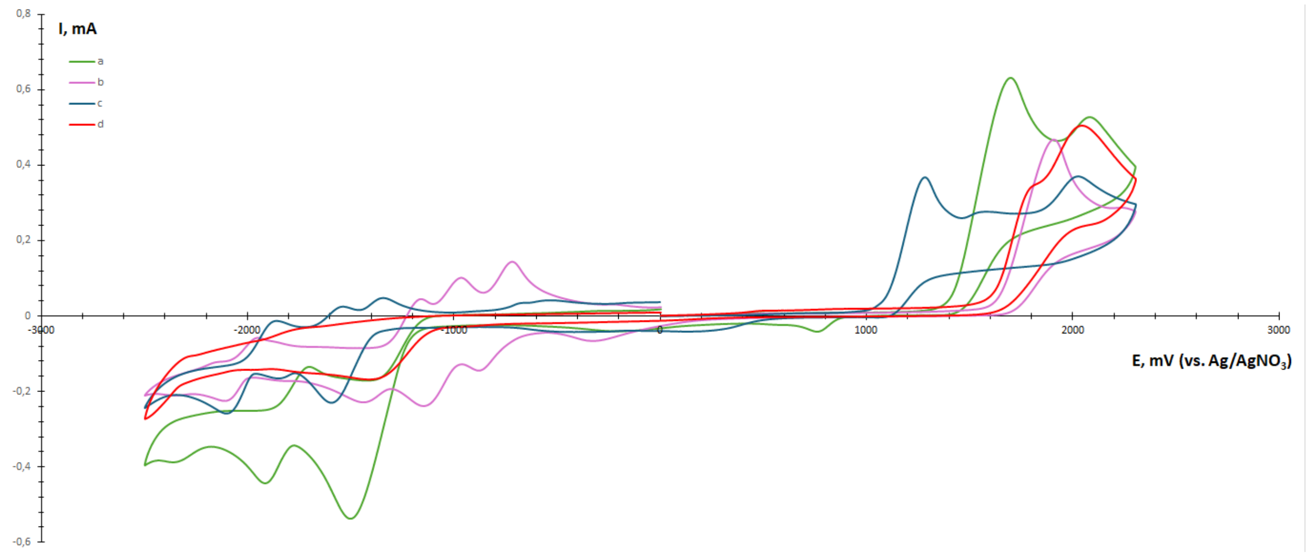


**Figure S1.** CV-curves of 0.01 M solutions of a) **1a** (green), b) **1e** (pink), c) **1i** (blue) and d) ADN (red) in 0.1M *n*-$Bu_4NBF_4$ solution in MeCN on a working glassy-carbon electrode (d = 3 mm) under a scan rate of 0.1 V·s$^{-1}$ at 298 K.

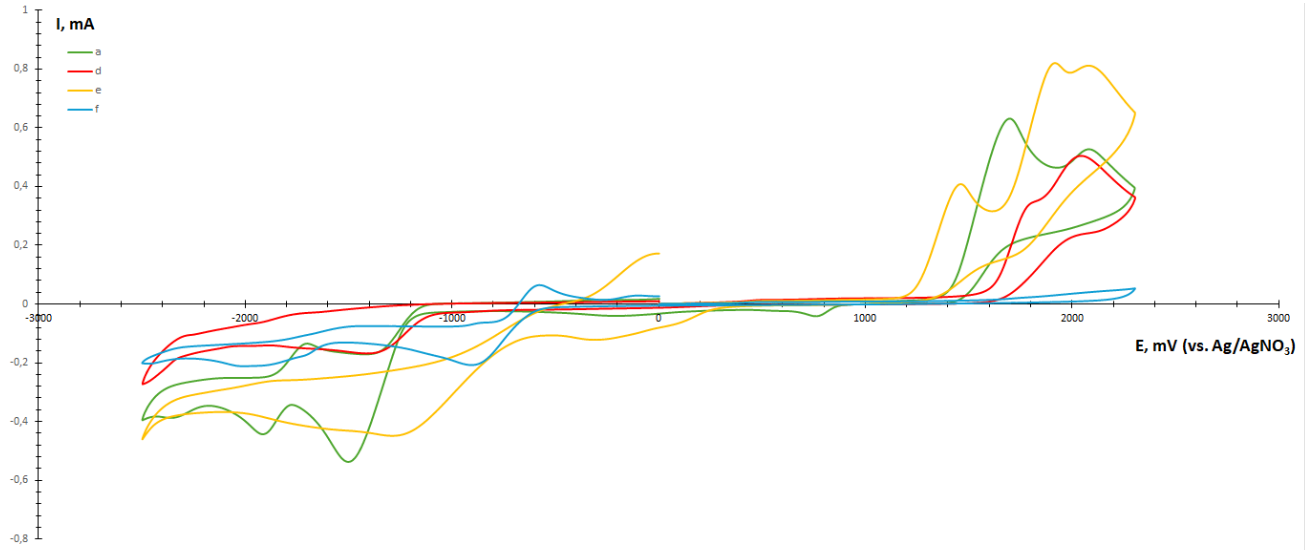


**Figure S2.** CV-curves of 0.01 M solutions of a) **1a** (green), d) ADN (red), e) the mixture of **1a** and ADN (yellow), f) **2a** (blue) in 0.1M *n*-$Bu_4NBF_4$ solution in MeCN on a working glassy-carbon electrode (d = 3 mm) under a scan rate of 0.1 V·s$^{-1}$ at 298 K.

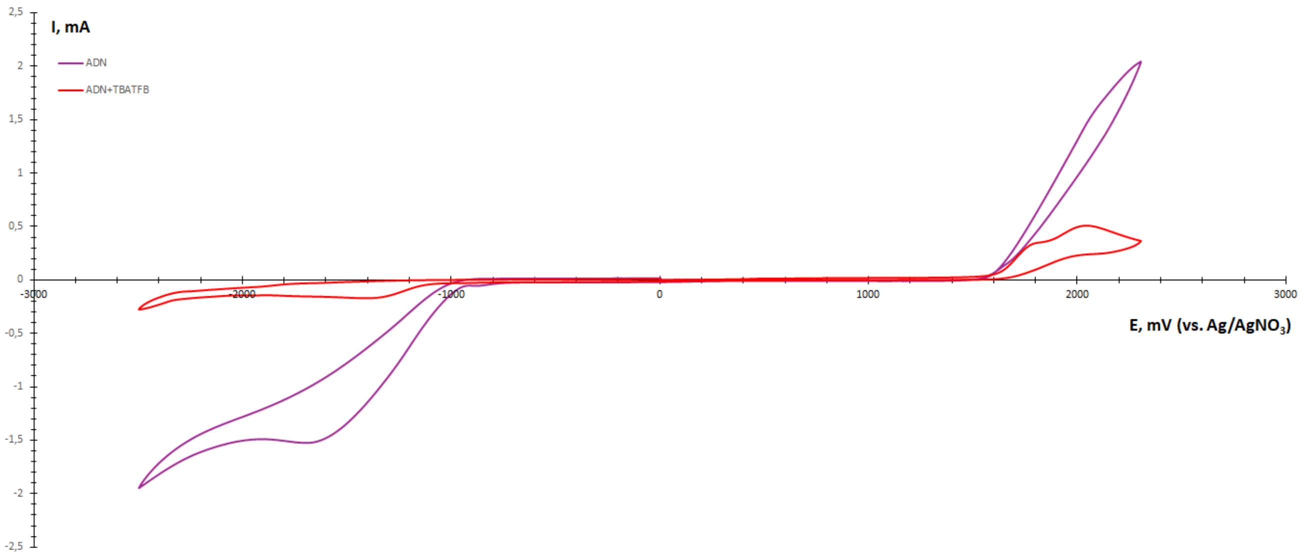


**Figure S3.** CV-curves of 0.1 M solution of ADN in absence of electrolyte in MeCN (purple) and ADN (0.01 M) in 0.1 M $n$-$Bu_4NBF_4$ solution in MeCN (red) on a working glassy-carbon electrode (d = 3 mm) under a scan rate of 0.1 V·s$^{-1}$ at 298 K.

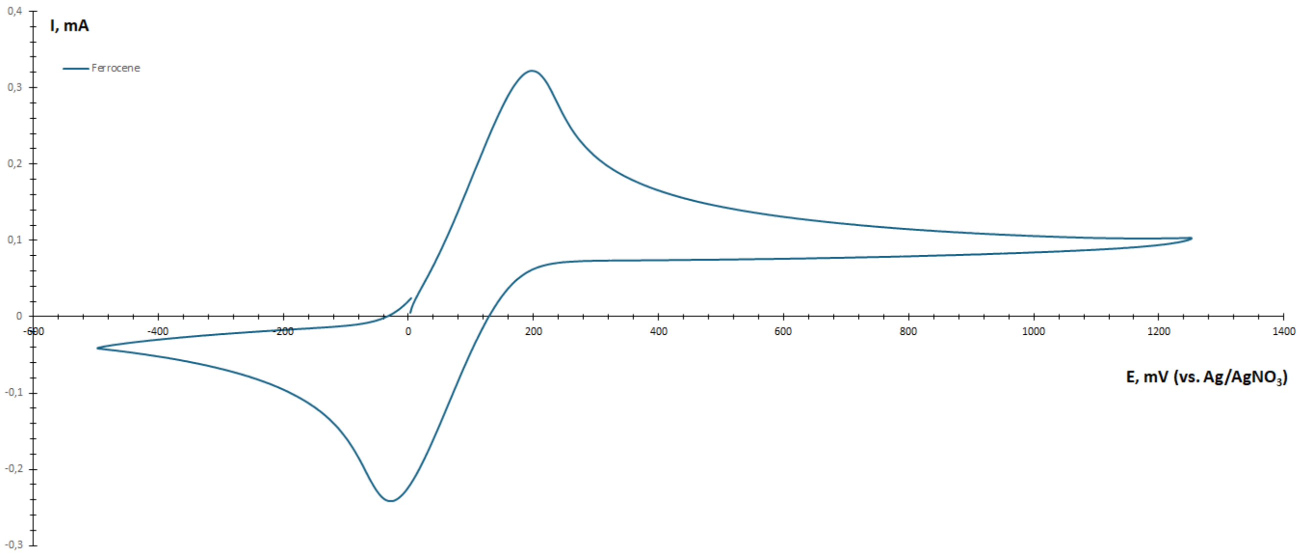


**Figure S4.** CV-curve of 0.01 M solution of ferrocene in 0.1 M $n$-$Bu_4NBF_4$ solution in MeCN on a working glassy-carbon electrode (d = 3 mm) under a scan rate of 0.1 V·s$^{-1}$ at 298 K.

## 3. Characterization of Products

**1-(nitro-*NNO*-azoxy)benzene (2a)**: White crystals, m.p. 26–27 °C (lit. m.p. 26–27 °C).[17]

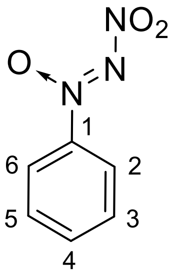


75% yield. $R_f$ (petroleum ether/ethyl acetate, 10:1) = 0.51. $^1$H NMR (500.13 MHz, $CDCl_3$)$\delta$: 7.57 (t, 2H, H(3), H(5), $^3J_{HH}$ = 8.2 Hz), 7.21 (t, 1H, H(4), $^3J_{HH}$ = 7.5 Hz), 8.10 (dd, 2H, H(2), H(6), $^3J_{HH}$ = 8.8 Hz, $^4J_{HH}$ = 1.1 Hz) ppm. $^{13}$C NMR (125.76 MHz, $CDCl_3$)$\delta$: 122.2 (s, C(2), C(6)), 129.8 (s, C(3), C(5)), 134.7 (s, C(4)), 142.8 (br. s, C(1)) ppm. The $^1$H–$^{13}$C HSQC and HMBC experiments were used to assign the signals. $^{14}$N NMR (36.14 MHz, $CDCl_3$)$\delta$: –32 (N(O)=N–<u>N</u>$O_2$, $\Delta\nu_{1/2}$ = 20 Hz), –43 (<u>N</u>(O)=N–$NO_2$, $\Delta\nu_{1/2}$ = 130 Hz) ppm. IR (KBr): $\nu$ = 2955 (w), 2884 (w), 1635 (s), 1505 (w), 1328 (w), 1282 (s)

cm$^{-1}$. Elemental analysis calcd (%) for $C_6H_5N_3O_3$: C 43.12, H 3.02, N 25.14; found: C 43.15, H 3.03, N 25.01.

**1-Methyl-4-(nitro-*NNO*-azoxy)benzene (2b)**: Orange oil. 80% yield. $R_f$ (petroleum ether/ethyl acetate, 40:1) = 0.25. $^1$H NMR (500.13 MHz, $CDCl_3$)*δ*: 2.48 (s, 3H, Me), 7.35 (d, 2H, H(2), H(6), $^3J_{HH}$ = 8.2 Hz), 7.99 (d, 2H, H(3), H(5), $^3J_{HH}$ = 8.2 Hz) ppm. $^{13}$C NMR (125.76 MHz, $CDCl_3$)*δ*: 21.1 (s, Me), 121.5 (s, C(3), C(5)), 129.7 (s, C(2), C(6)), 139.9 (br. s, C(4)), 145.6 (s, C(1)) ppm. The $^1$H–$^{13}$C HSQC and HMBC experiments were used to assign the signals. $^{14}$N NMR (36.14 MHz, $CDCl_3$)*δ*: –33 (N(O)=N–NO$_2$, $\Delta\nu_{½}$ = 20 Hz), –45 (N(O)=N–NO$_2$, $\Delta\nu_{½}$ = 130 Hz) ppm. IR (KBr): ν = 2952 (w), 2889 (w), 1611 (s), 1519 (w), 1478 (s), 1383 (w), 1345 (w), 1318 (w), 1268 (s), 1181 (m), 1116 (w) cm$^{-1}$. Elemental analysis calcd (%) for $C_7H_7N_3O_3$: C 46.41, H 3.90, N 23.20; found: C 46.45, H 3.93, N 23.01.

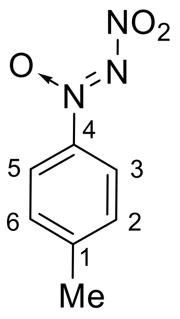


**1-Methyl-3-(nitro-*NNO*-azoxy)benzene (2c)**: Orange oil. 74% yield. $R_f$ (petroleum ether/ethyl acetate, 40:1) = 0.50. $^1$H NMR (500.13 MHz, $CDCl_3$)*δ*: 2.47 (s, 3H, Me), 7.45 (t, 1H, H(5), $^3J_{HH}$ = 7.8 Hz), 7.52 (m, 1H, H(6)), 7.90–7.92 (m, 2H, H(2), H(4)) ppm. $^{13}$C NMR (125.76 MHz, $CDCl_3$)*δ*: 21.3 (s, Me), 119.3 (s, C(4)), 122.5 (s, C(2)), 129.4 (s, C(5)), 135.3 (s, C(6)), 140.3 (s, C(1)), 142.7 (br. s, C(3)) ppm. The $^1$H–$^{13}$C HSQC and HMBC experiments were used to assign the signals. $^{14}$N NMR (43.4 MHz, $CDCl_3$)*δ*: –35 (N(O)=N–NO$_2$, $\Delta\nu_{½}$ = 15 Hz), –46 (N(O)=N–NO$_2$, $\Delta\nu_{½}$ = 100 Hz) ppm. IR (KBr): ν = 2924 (w), 2871 (w), 1610 (s), 1502 (s), 1470 (m), 1427 (w), 1383 (w), 1316 (w), 1295 (w), 1270 (s), 1220 (w), 1169 (w) cm$^{-1}$. Elemental analysis calcd (%) for $C_7H_7N_3O_3$: C 46.41, H 3.90, N 23.20; found: C 46.46, H 3.91, N 23.05.

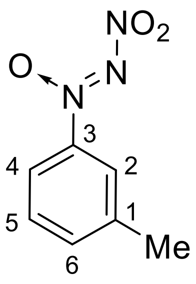


**1-Methyl-2-(nitro-*NNO*-azoxy)benzene (2d)**: Orange oil. 71% yield. $R_f$ (petroleum ether/ethyl acetate, 10:1) = 0.83. $^1$H NMR (600.13 MHz, $CDCl_3$)*δ*: 2.51 (s, 3H, Me), 7.36–7.39 (m, 2H, H(4), H(6)), 7.52 (t, 1H, H(5), $^3J_{HH}$ = 7.5 Hz), 7.69 (d, 1H, H(3), $^3J_{HH}$ = 8.0 Hz) ppm. $^{13}$C NMR (150.90 MHz, $CDCl_3$)*δ*: 19.1 (s, Me), 124.7 (s, C(3)), 127.9 (s, C(4)), 133.3 (s, C(1), C(6)), 133.4 (s, C(5)), 143.8 (br. s, C(2)) ppm. The $^1$H–$^{13}$C HSQC and HMBC experiments were used to assign the signals. $^{14}$N NMR (43.4 MHz, $CDCl_3$)*δ*: –34 (N(O)=N–NO$_2$, $\Delta\nu_{½}$ = 10 Hz), –41 (N(O)=N–NO$_2$, $\Delta\nu_{½}$ = 90 Hz) ppm. IR (KBr): ν = 2933 (w), 2878 (w), 1612 (s), 1524 (w), 1492 (m), 1473 (m), 1430 (w),

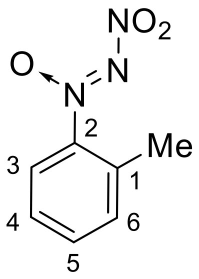

1385 (w), 1348 (w), 1302 (w), 1427 (w), 1273 (s), 1207 (w), 1163 (w), 1148 (w) $cm^{-1}$. Elemental analysis calcd (%) for $C_7H_7N_3O_3$: C 46.41, H 3.90, N 23.20; found: C 46.50, H 3.94, N 23.09.

**4-Nitro-1-(nitro-*NNO*-azoxy)benzene (2e)**: Pale yellow crystals, m.p. 49–50 °C (lit. m.p. 49–51 °C).[17] 79% yield. $R_f$ (petroleum ether/ethyl acetate, 5:1) = 0.76. $^1$H NMR (500.13 MHz, $CDCl_3$)$\delta$: 8.39 (d, 2H, H(2), H(6), $^3J_{HH}$ = 8.9 Hz), 8.49 (d, 2H, H(3), H(5), $^3J_{HH}$ = 8.9 Hz) ppm. $^{13}$C NMR (150.9 MHz, $CDCl_3$)$\delta$: 123.9 (s, C(2), C(6)), 125.2 (s, C(3), C(5)), 146.4 (br. s, C(1)), 151.1 (br. s, C(4)) ppm. The $^1$H–$^{13}$C HSQC and HMBC experiments were used to assign the signals. $^{14}$N NMR (36.14 MHz, $CDCl_3$)$\delta$: –17 ($NO_2$, $\Delta\nu_{½}$ = 155 Hz), –37 (N(O)=N–N̲O₂, $\Delta\nu_{1/2}$ = 20 Hz), –50 (N̲(O)=N–$NO_2$, $\Delta\nu_{½}$ = 120 Hz) ppm. IR (KBr): $\nu$ = 2873 (w), 1656 (m), 1631 (s), 1602 (s), 1539 (s), 1484 (m), 1407 (w), 1383 (w), 1319 (s), 1274 (m), 1172 (w), 1149 (w), 1125 (w), 1108 (w) $cm^{-1}$. Elemental analysis calcd (%) for $C_6H_4N_4O_5$: C 33.97, H 1.90, N 26.41; found: C 33.99, H 1.94, N 26.09.

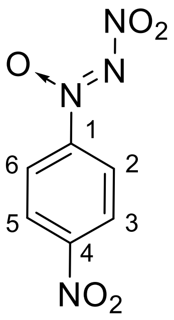


**3-Nitro-1-(nitro-*NNO*-azoxy)benzene (2f)**: Pale yellow crystals, m.p. 53–54 °C (lit. m.p. 53–54 °C).[17] 46% yield. $R_f$ (petroleum ether/ethyl acetate, 10:1) = 0.61.$^1$H NMR (600.13 MHz, $CDCl_3$)$\delta$: 7.92 (t, 1H, H(5), $^3J_{HH}$ = 8.3 Hz), 8.56 (d, 1H, H(4) or H(6), $^3J_{HH}$ = 8.3 Hz), 8.64 (d, 1H, H(6) or H(4), $^3J_{HH}$ = 8.3 Hz), 9.02 (s, 1H, H(2) ppm. $^{13}$C NMR (150.90 MHz, $CDCl_3$)$\delta$: 118.0 (s, C(2)), 127.8 (s, C(4) or C(6)), 129.0 (s, C(4) or C(6)), 131.3 (s, C(5)), 143.3 (br. s, C(1)), 148.5 (br. s, C(3)) ppm. The $^1$H–$^{13}$C HSQC and HMBC experiments were used to assign the signals. $^{14}$N NMR (43.37 MHz, $CDCl_3$)$\delta$: –18 ($NO_2$, $\Delta\nu_{½}$ = 150 Hz), –38 (N(O)=N–N̲O₂, $\Delta\nu_{½}$ = 30 Hz), –51 (*N*(O)=N–$NO_2$, $\Delta\nu_{½}$ = 130 Hz) ppm. IR (KBr): $\nu$ = 2923 (w), 2877 (w), 2854 (w), 1625 (s), 1535 (s), 1493 (s), 1485 (m), 1434 (w), 1351 (s), 1322 (m), 1286 (m), 1265 (s), 1165 (w), 1148 (w) $cm^{-1}$. Elemental analysis calcd (%) for $C_6H_4N_4O_5$: C 33.97, H 1.90, N 26.41; found: C 34.01, H 1.92, N 26.21.

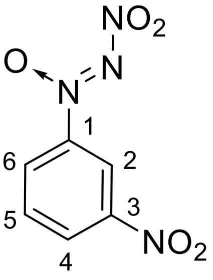


**2-Nitro-1-(nitro-*NNO*-azoxy)benzene (2g)**: Pale yellow crystals, m.p. 38–39 °C (lit. m.p. 39–40 °C).[17] 70% yield. $R_f$ (petroleum ether/ethyl acetate, 10:1) = 0.55. $^1$H NMR (600.13 MHz, $CDCl_3$)$\delta$: 7.88–7.92 (m, 3H, H(4), H(5), H(6)), 8.22 (d, 1H, H(3), $^3J_{HH}$ = 7.3 Hz) ppm. $^{13}$C NMR (150.90 MHz, $CDCl_3$)$\delta$: 126.0 (s, C(6)), 126.3 (s, C(3)), 133.8 (s, C(4)), 134.7 (s, C(5)), 137.1 (br. s, C(1)), 142.4 (br. s, C(2)) ppm. The $^1$H–$^{13}$C HSQC and HMBC

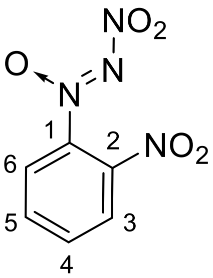

experiments were used to assign the signals. $^{14}$N NMR (43.37 MHz, $CDCl_3$)$\delta$: –20 ($NO_2$, $\Delta\nu_{½}$ = 55 Hz), –39 (N(O)=N–<u>N</u>$O_2$, $\Delta\nu_{½}$ = 15 Hz), –51 (<u>N</u>(O)=N–$NO_2$, $\Delta\nu_{½}$ = 50 Hz) ppm. IR (KBr): $\nu$ = 2918 (w), 2887 (w), 1621 (s), 1542 (s), 1494 (s), 1449 (w), 1437 (w), 1406 (w), 1351 (s), 1313 (w), 1280 (s), 1265 (s), 1169 (w), 1147 (w) cm$^{-1}$. Elemental analysis calcd (%) for $C_6H_4N_4O_5$: C 33.97, H 1.90, N 26.41; found: C 34.00, H 1.94, N 26.25.

**1-Methoxy-4-(nitro-*NNO*-azoxy)benzene (2i)**: Pale yellow oil. 83% yield. $R_f$ (petroleum ether/ethyl acetate, 10:1) = 0.35. An analytical sample was obtained by vacuum distillation at 65 °C (0.75 Torr). $^1$H NMR (600.13 MHz, $CDCl_3$)$\delta$: 3.93 (s, 3H, OMe), 7.01 (d, 2H, H(2), H(6), $^3J_{HH}$ = 9.2 Hz), 8.09 (d, 2H, H(3), H(5), $^3J_{HH}$ = 9.2 Hz) ppm. $^{13}$C NMR (150.90 MHz, $CDCl_3$)$\delta$: 56.1 (s, OMe), 114.5 (s, C(2), C(6)), 124.3 (s, C(3), C(5)), 135.3 (br. s, C(4)), 164.6 (s, C(1)) ppm. The $^1$H–$^{13}$C HSQC and HMBC experiments were used to assign the signals. $^{14}$N NMR (43.4 MHz, $CDCl_3$)$\delta$: –33 (N(O)=N–<u>N</u>$O_2$, $\Delta\nu_{½}$ = 20 Hz), –46 (<u>N</u>(O)=N–$NO_2$, $\Delta\nu_{½}$ = 200 Hz) ppm. IR (KBr): $\nu$ = 2978 (w), 2948 (w), 1594 (s), 1541 (m), 1499 (s), 1471 (s), 1417 (m), 1336 (m), 1316 (m), 1258 (s), 1174 (s), 1118 (s) cm$^{-1}$. Elemental analysis calcd (%) for $C_7H_7N_3O_4$: C 42.65, H 3.58, N 21.31; found: C 42.70, H 3.64, N 21.15.

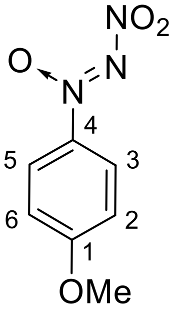


**1-Methoxy-3-(nitro-*NNO*-azoxy)benzene (2j)**: Yellow oil. 79% yield. $R_f$ (petroleum ether/ethyl acetate, 10:1) = 0.57. $^1$H NMR (600.13 MHz, $CDCl_3$)$\delta$: 3.88 (s, 3H, OMe), 7.24 (dd, 1H, H(6), $^3J_{HH}$ = 8.3 Hz, $^4J_{HH}$ = 2.3 Hz), 7.46 (t, 1H, H(5), $J_{HH}$ = 8.3 Hz), 7.57 (s, 1H, H(2)), 7.69 (dd, 1H, H(4), $^3J_{HH}$ = 8.3 Hz, $^4J_{HH}$ = 1.8 Hz) ppm. $^{13}$C NMR (150.9 MHz, $CDCl_3$)$\delta$: 55.9 (s, OMe), 107.1 (s, C(2)), 114.2 (s, C(4)), 121.0 (s, C(6)), 130.4 (s, C(5)), 143.5 (br. s, C(3)), 160.2 (s, C(1)) ppm. The $^1$H–$^{13}$C HSQC and HMBC experiments were used to assign the signals. $^{14}$N NMR (43.4 MHz, $CDCl_3$)$\delta$: –34 (N(O)=N–<u>N</u>$O_2$, $\Delta\nu_{½}$ = 30 Hz), –46 (<u>N</u>(O)=N–$NO_2$, $\Delta\nu_{½}$ = 215 Hz) ppm. IR (KBr): $\nu$ = 2970 (w), 2942 (w), 2841 (w), 1612 (s), 1585 (m), 1530 (m), 1502 (s), 1472 (m), 1447 (m), 1384 (w), 1335 (m), 1319 (m), 1290 (m), 1271 (s), 1250 (s), 1185 (w), 1106 (w) cm$^{-1}$. Elemental analysis calcd (%) for $C_7H_7N_3O_4$: C 42.65, H 3.58, N 21.31; found: C 42.72, H 3.59, N 21.05.

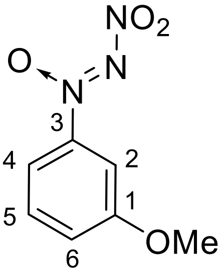

**1-Methoxy-2-(nitro-*NNO*-azoxy)benzene (2k)**: Yellow oil. 51% yield. $R_f$ (petroleum ether/ethyl acetate, 10:1) = 0.71. $^1$H NMR (300.13 MHz, $CDCl_3$)$\delta$: 3.89 (s, 3H, OMe), 7.24 (dd, 1H, H(6), $^3J_{HH}$ = 8.3 Hz, $^4J_{HH}$ = 2.5 Hz), 7.46 (t, 1H, H(5), $^3J_{HH}$ = 8.3 Hz), 7.60 (t, 1H, H(4), $^3J_{HH}$ = 2.3 Hz), 7.69 (dd, 1H, H(3), $^3J_{HH}$ = 8.1 Hz, $^4J_{HH}$ = 2.2 Hz) ppm. $^{13}$C NMR (75.49 MHz, $CDCl_3$)$\delta$: 55.9 (s, OMe), 107.2 (s, C(4)), 114.3 (s, C(3)), 121.0 (s, C(6)), 130.4 (s, C(5)), 143.8 (br. s, C(2)), 160.4 (s, C(1)) ppm. The $^1$H–$^{13}$C HSQC and HMBC experiments were used to assign the signals. $^{14}$N NMR (43.4 MHz, $CDCl_3$)$\delta$: –34 (N(O)=N–<u>N</u>$O_2$, $\Delta\nu_{½}$ = 20 Hz), –45 (<u>N</u>(O)=N–$NO_2$, $\Delta\nu_{½}$ = 115 Hz) ppm. IR (KBr): ν = 2969 (w), 2941 (w), 2841 (w), 1612 (s), 1503 (s), 1472 (m), 1447 (m), 1384 (w), 1335 (m), 1319 (m), 1290 (m), 1271 (s), 1250 (s), 1185 (w), 1106 (w) cm$^{-1}$. Elemental analysis calcd (%) for $C_7H_7N_3O_4$: C 42.65, H 3.58, N 21.31; found: C 42.66, H 3.58, N 21.21.

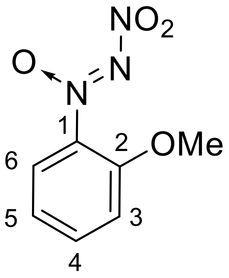


**4-Fluoro-1-(nitro-*NNO*-azoxy)benzene (2l):** Yellow oil (mixture with 4-fluoro-1-nitrobenzene in 1.5 : 1 ratio). 36% yield. $R_f$ (petroleum ether/ethyl acetate, 10:1) = 0.65. $^1$H NMR (600.13 MHz, $CDCl_3$)δ = 7.28 (m, 2H, H(2), H(6)), 8.19 (m, 2H, H(3), H(5)) ppm. $^{13}$C NMR (150.9 MHz, $CDCl_3$)δ = 117.0 (d, C(3), C(5), $^2J_{CF}$ = 23.8 Hz), 125.0 (d, C(2), C(6), $^3J_{CF}$ = 9.9 Hz), 138.7 (br. s, C(1)) ppm. The $^1$H–$^{13}$C HSQC and HMBC experiments were used to assign the signals. $^{14}$N NMR (43.37 MHz, $CDCl_3$)δ = –35 (N(O)=N–<u>N</u>$O_2$, $\Delta\nu_{½}$ = 30 Hz), –48 (<u>N</u>(O)=N–$NO_2$, $\Delta\nu_{½}$ = 130 Hz) ppm.

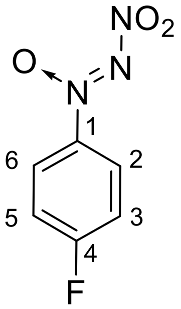


**4-Chloro-1-(nitro-*NNO*-azoxy)benzene (2m):** Yellowish oil. 52% yield. $R_f$ (petroleum ether/ethyl acetate, 30:1) = 0.44. An analytical sample was obtained by two-fold vacuum distillation at 65 °C (0.75 Torr). $^1$H NMR (600.13 MHz, $CDCl_3$) δ = 7.57 (d, 2H, H(3), H(5), $^3J_{H,H}$ = 9.0 Hz), 8.11 (d, 2H, H(2), H(6), $^3J_{H,H}$ = 9.0 Hz) ppm. $^{13}$C NMR (150.9 MHz, $CDCl_3$) δ = 123.6 (s, C(2), C(6)), 130.0 (s, C(3), C(5)), 141.3 (br. s, C(1), C(4)) ppm. The $^1$H–$^{13}$C HSQC and HMBC experiments were used to assign the signals. $^{14}$N NMR (43.37 MHz, $CDCl_3$) δ = –36 (N(O)=N–<u>N</u>$O_2$, $\Delta\nu_{½}$ = 35 Hz), –48 (<u>N</u>(O)=N–$NO_2$, $\Delta\nu_{½}$ = 135 Hz) ppm. IR (KBr): ν = 2873 (w), 1610 (s), 1584 (m), 1523 (w), 1478 (s), 1401 (w), 1344 (w), 1315 (m), 1269 (s), 1174 (m), 1139 (w), 1123 (w), 1108 (w) cm$^{-1}$. Elemental analysis calcd (%) for $C_6H_4ClN_3O_3$: C 35.75, H 2.00, N 20.85; found: C 35.79, H 2.03, N 20.63.

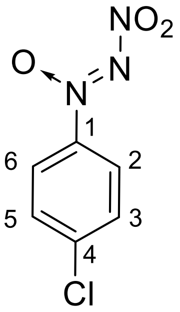

**2,4-Dichloro-1-(nitro-*NNO*-azoxy)benzene (2n):** Brown crystals, m.p. 45–46 °C. 53% yield. $R_f$ (petroleum ether/ethyl acetate, 10:1) = 0.50. An analytical sample was obtained by recrystallization from hexane at –20 °C. $^{1}$H NMR (500.13 MHz, $CDCl_3$) δ = 7.47 (d, 1H, H(5), $^{3}J_{H,H}$ = 8.6 Hz), 7.62 (s, 1H, H(3)), 7.71 (d, 1H, H(6), $^{3}J_{H,H}$ = 8.6 Hz) ppm. $^{13}$C NMR (125.76 MHz, $CDCl_3$) δ = 126.5 (s, C(6)), 128.4 (s, C(5)), 131.7 (s, C(3)), 139.5 (s, C(2)), 140.0 (br.s, C(1)) ppm. The $^{1}$H–$^{13}$C HSQC and HMBC experiments were used to assign the signals. $^{14}$N NMR (36.14 MHz, $CDCl_3$) δ = –36 (N(O)=N–N$O_2$, $\Delta\nu_{1/2}$ = 25 Hz), –49 (N(O)=N–N$O_2$, $\Delta\nu_{1/2}$ = 100 Hz) ppm. IR (KBr): ν = 2940 (w), 2875 (w), 1619 (s), 1582 (s), 1566 (s), 1482 (s), 1464 (s), 1393 (w), 1376 (m), 1342 (w), 1317 (m), 1274 (s), 1145 (m), 1107 (m) cm$^{-1}$. Elemental analysis calcd (%) for $C_6H_3Cl_2N_3O_3$: C 30.54, H 1.28, N 17.80; found: C 30.55, H 1.31, N 17.63.

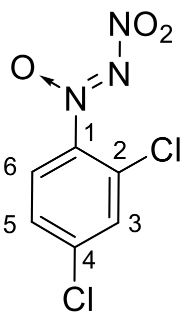


**2,6-Dichloro-1-(nitro-*NNO*-azoxy)benzene (2o):** Brown crystals, m.p. 53–54 °C (lit. m.p. 54–56 °C).[17] 64% yield. $R_f$ (petroleum ether/ethyl acetate, 10:1) = 0.57. $^{1}$H NMR (500.13 MHz, $CDCl_3$) δ = 7.80–7.93 (m, 3H, H(3), H(4), H(5)) ppm. $^{13}$C NMR (125.76 MHz, $CDCl_3$) δ = 128.4 (s, C(2), C(6)), 130.6 (s, C(3), C(5)), 135.1 (s, C(4)), 140.0 (br.s, C(1)) ppm. The $^{1}$H–$^{13}$C HSQC and HMBC experiments were used to assign the signals. $^{14}$N NMR (36.14 MHz, $CDCl_3$) δ = –37 (N(O)=N–N$O_2$, $\Delta\nu_{1/2}$ = 25 Hz), –53 (N(O)=N–N$O_2$, $\Delta\nu_{1/2}$ = 85 Hz) ppm. IR (KBr): ν = 2917 (w), 2892 (w), 1624 (s), 1578 (m), 1556 (m), 1492 (s), 1448 (m), 1388 (w), 1370 (w), 1330 (m), 1304 (s), 1277 (s), 1206 (m), 1168 (m) cm$^{-1}$. Elemental analysis calcd (%) for $C_6H_3Cl_2N_3O_3$: C 30.54, H 1.28, N 17.80; found: C 30.60, H 1.35, N 17.53.

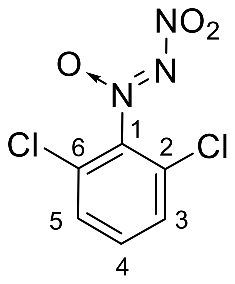


**2,4,6-Trichloro-1-(nitro-*NNO*-azoxy)benzene (2p):** Beige crystals, m.p. 80–81 °C (lit. m.p. 80–81 °C).[18] 80% yield. $R_f$ (petroleum ether/ethyl acetate, 40:1) = 0.70. $^{1}$H NMR (500.13 MHz, $CDCl_3$) δ = 7.84 (s, 2H, H(3), H(5)) ppm. $^{13}$C NMR (125.76 MHz, $CDCl_3$) δ = 129.5 (br. s, C(4)), 130.8 (s, C(3), C(5)), 135.1 (s, C(2), C(6)), 140.3 (br.s, C(1)) ppm. The $^{1}$H–$^{13}$C HSQC and HMBC experiments were used to assign the signals. $^{14}$N NMR (36.14 MHz, $CDCl_3$) δ = –36 (N(O)=N–N$O_2$, $\Delta\nu_{1/2}$ = 30 Hz), –52 (N(O)=N–N$O_2$, $\Delta\nu_{1/2}$ = 90 Hz) ppm. IR (KBr): ν = 2952 (w), 2926 (w), 2901 (w), 2855 (w), 1624 (s), 1566 (s), 1494 (s), 1451 (m), 1430 (m), 1390 (m), 1371 (m), 1330 (w), 1286 (s), 1196 (w), 1128 (m) cm$^{-1}$. Elemental analysis calcd (%) for $C_6H_2Cl_3N_3O_3$: C 26.65, H 0.75, N 15.54; found: C 26.76, H 0.77, N 15.32.

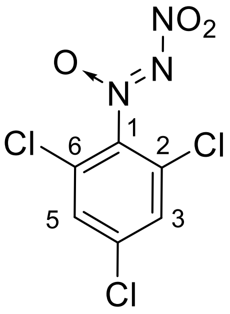

**4-Bromo-1-(nitro-*NNO*-azoxy)benzene (2q):** Yellowish crystals, m.p. 74–75 °C (lit. m.p. 74–76 °C).[17] 50% yield. $R_f$ (petroleum ether/ethyl acetate, 40:1) = 0.42. An analytical sample was obtained by three-fold recrystallization from mixture hexane/$CH_2Cl_2$ (10:1) at –20 °C. $^1H$ NMR (600.13 MHz, $CDCl_3$) δ = 7.73 (d, 2H, H(3), H(5), $^3J_{H,H}$ = 8.9 Hz), 8.01 (d, 2H, H(2), H(6), $^3J_{H,H}$ = 8.9 Hz) ppm. $^{13}C$ NMR (150.9 MHz, $CDCl_3$) δ = 123.7 (s, C(2), C(6)), 129.8 (br. s, C(4)), 133.0 (s, C(3), C(5)), 141.7 (br. s, C(1)) ppm. The $^1H$–$^{13}C$ HSQC and HMBC experiments were used to assign the signals. $^{14}N$ NMR (43.37 MHz, $CDCl_3$) δ = –36 (N(O)=N–<u>N</u>$O_2$, $\Delta\nu_{½}$ = 20 Hz), –48 (<u>N</u>(O)=N–$NO_2$, 75 Hz) ppm. IR (KBr): ν = 2921 (w), 2870 (w), 1606 (s), 1576 (m), 1519 (w), 1473 (s), 1396 (w), 1310 (w), 1280 (m), 1175 (w), 1108 (w) $cm^{-1}$. Elemental analysis calcd (%) for $C_6H_4BrN_3O_3$: C 29.29, H 1.64, N 17.08; found: C 29.31, H 1.65, N 16.91.

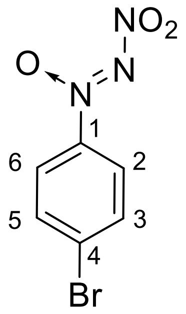


**2,4-Dibromo-1-(nitro-*NNO*-azoxy)benzene (2r):** Orange crystals, m.p. 71–72 °C. 34% yield. $R_f$ (petroleum ether/ethyl acetate, 40:1) = 0.54. $^1H$ NMR (600.13 MHz, $CDCl_3$) δ = 7.60 (d, 1H, H(6), $^3J_{H,H}$ = 8.5 Hz), 7.67 (d, 1H, H(5), $^3J_{H,H}$ = 8.4 Hz), 7.96 (s, 1H, H(3)) ppm. $^{13}C$ NMR (150.9 MHz, $CDCl_3$) δ = 117.4 (s, C(4) or C(2)), 127.3 (s, C(6)), 128.1 (s, C(2) or C(4)), 132.6 (s, C(5)), 138.2 (s, C(3)), 143.0 (br. s, C(1)) ppm. The $^1H$–$^{13}C$ HSQC and HMBC experiments were used to assign the signals. $^{14}N$ NMR (43.37 MHz, $CDCl_3$) δ = –37 (N(O)=N–<u>N</u>$O_2$, $\Delta\nu_{½}$ = 35 Гц), –47 (<u>N</u>(O)=N–$NO_2$, $\Delta\nu_{½}$ = 80 Гц) ppm. IR (KBr): ν = 2931 (w), 2881 (w), 2811 (w), 1606 (s), 1568 (s), 1535 (s), 1479 (s), 1459 (s), 1387 (w), 1371 (m), 1331 (m), 1288 (s), 1245 (s), 1149 (m), 1109 (w) $cm^{-1}$. Elemental analysis calcd (%) for $C_6H_3Br_2N_3O_3$: C 22.18, H 0.93, N 12.93; found: C 22.18, H 0.94, N 12.71.

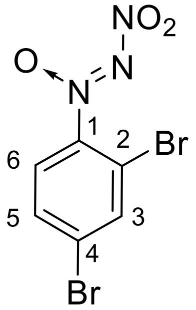


**2,6-Dibromo-1-(nitro-*NNO*-azoxy)benzene (2s):** Beige crystals, m.p. 97–98 °C. 53% yield. $R_f$ (petroleum ether/ethyl acetate, 6:1) = 0.49. $^1H$ NMR (600.13 MHz, $CDCl_3$) δ = 7.35 (t, $^3J_{H,H}$ = 8.1 Hz, 1H, H(4)), 7.70 (d, $^3J_{H,H}$ = 8.2 Hz, 2H, H(3), H(5)) ppm. $^{13}C$ NMR (150.9 MHz, $CDCl_3$) δ = 118.1 (s, C(2), C(6)), 133.8 (s, C(3), C(5)), 134.0 (s, C(4)), 143.7 (br. s, C(1)) ppm. The $^1H$–$^{13}C$ HSQC and HMBC experiments were used to assign the signals. $^{14}N$ NMR (43.37 MHz, $CDCl_3$) δ = –37 (N(O)=N–<u>N</u>$O_2$, $\Delta\nu_{½}$ = 25 Hz), –48 (<u>N</u>(O)=N–$NO_2$, $\Delta\nu_{½}$ = 125 Hz) ppm. IR (KBr): ν = 2917 (w), 2889 (w), 2849 (w), 1633 (s), 1567 (m), 1535 (s), 1490 (s), 1441 (m), 1422 (w), 1370 (w), 1327 (w), 1295

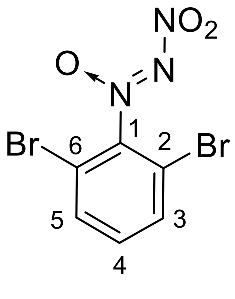

(m), 1274 (s), 1288 (s), 1201 (m), 1161 (w) $cm^{-1}$. Elemental analysis calcd (%) for $C_6H_3Br_2N_3O_3$: C 22.18, H 0.93, N 12.93; found: C 22.18, H 0.94, N 12.71.

**2,4,6-Tribromo-1-(nitro-*NNO*-azoxy)benzene (2t):** Beige crystals, m.p. 113–114 °C.

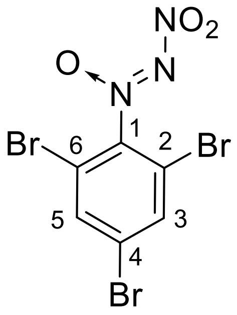

53% yield. $R_f$ (petroleum ether/ethyl acetate, 40:1) = 0.46. $^1H$ NMR (500.13 MHz, $CDCl_3$) δ = 7.87 (s, 2H, H(3), H(5)) ppm. $^{13}C$ NMR (125.76 MHz, $CDCl_3$) δ = 117.4 (s, C(2), C(6)), 126.1 (s, C(4)), 134.8 (s, C(3), C(5)), 141.6 (s, C(4)) ppm. The $^1H$–$^{13}C$ HSQC and HMBC experiments were used to assign the signals. $^{14}N$ NMR (43.37 MHz, $CDCl_3$) δ = –38 (N(O)=N–<u>N</u>$O_2$, $\Delta\nu_{½}$ = 25 Hz), –50 (<u>N</u>(O)=N–$NO_2$, $\Delta\nu_{½}$ = 115 Hz) ppm. IR (KBr): ν = 2891 (w), 2814 (w), 1620 (s), 1551 (m), 1493 (m), 1430 (w), 1415 (w), 1370 (w), 1350 (w), 1329 (w), 1282 (m), 1248 (w), 1198 (w), 1161 (w), 1131 (w), 1112 (w) $cm^{-1}$. Elemental analysis calcd (%) for $C_6H_2Br_3N_3O_3$: C 17.85, H 0.50, N 10.41; found: C 17.87, H 0.52 N 10.20.

## 4. Pictures of the Equipment Used

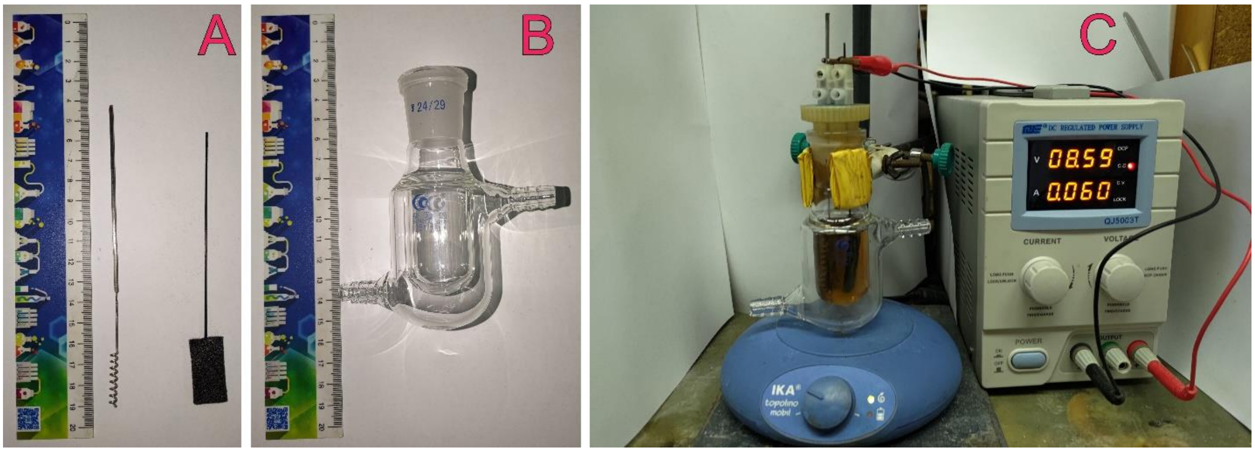


**Figure S5.** (A) Electrodes used in the reaction – platinum wire (left) and carbon felt (right). (B) Reaction vessel. (C) Assembled electrochemical setup.

## 5. Fungicidal activity tests (experimental details for Table 2).

The strains used in this work were obtained from the collection of the All-Russian Research Institute for Phytopathology (B. Vyazemy, Moscow reg., Russia).

Fungicidal activity was measured against six phytopathogenic fungi from different taxonomic classes (*V.i.*—Venturia inaequalis MRA-16-2, *R.s.*—Rhizoctonia solani 100063, *F.o.*—Fusarium oxysporum FO-8, *F.m.*—Fusarium moniliforme 100146, *B.s.*—Bipolaris sorokiniana MRB(V)-1, *S.s.*—Sclerotinia sclerotiorum 100033) using the standard poison food technique.[19–24] The tested substances were dissolved in acetone (1 mg/mL) and incorporated into liquid sugar-potato agar at 50–55 °C to achieve a final

concentration of 10 mg/L. The agar-substance mixture was poured into sterile Petri dishes and allowed to cool to room temperature. Mycelial pieces from the peripheral growth zone of 3–5 day old fungal cultures were transferred to the test dishes using a needle. Colonies grown in medium with acetone alone served as controls. After 72 hours, the diameters of the fungal colonies were measured. Each experiment was repeated 3 times, except for tests with *V. inaequalis* which had 5 replicates. Mycelial growth suppression was calculated as $((D_c – D_s)/D_c) \times 100\%$, where $D_c$ is the average control colony diameter and $D_s$ is the average colony diameter in the presence of the tested substance.

| № | Compound | Mycelium growth inhibition, % | | | | | |
|---|---|---|---|---|---|---|---|
| | | *V. i.* | *R. s.* | *F. o.* | *F. m.* | *B. s.* | *S. s.* |
| 1 | 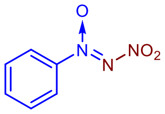 **2a** | 100 | 100 | 100 | 100 | 100 | 100 |
| 2 | 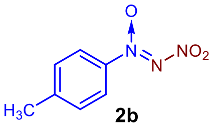 **2b** | 94 | 66 | 97 | 100 | 30 | 100 |
| 3 | 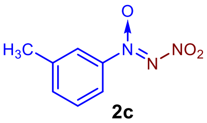 **2c** | 35 | 35 | 92 | 100 | 16 | 100 |
| 4 | 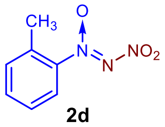 **2d** | 26 | 93 | 94 | 100 | 16 | 100 |
| 5 | 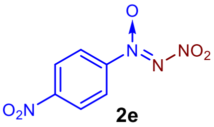 **2e** | 38 | 70 | 22 | 42 | 53 | 19 |
| 6 | 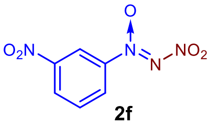 **2f** | 28 | 57 | 24 | 61 | 51 | 25 |
| 7 | 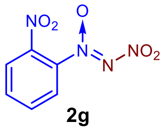 **2g** | 9 | 54 | 8 | 8 | 31 | 8 |
| 8 | 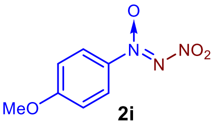 **2i** | 84 | 100 | 26 | 100 | 26 | 100 |
| 9 | 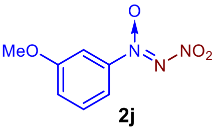 **2j** | 26 | 36 | 23 | 92 | 0 | 11 |
| 10 | 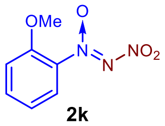 **2k** | 83 | 57 | 34 | 100 | 17 | 100 |

| | | | | | | | |
|---|---|---|---|---|---|---|---|
| 11 | 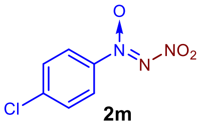 2m | 100 | 100 | 100 | 100 | 66 | 100 |
| 12 | 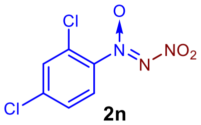 2n | 64 | 33 | 17 | 18 | 71 | 30 |
| 13 | 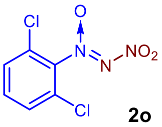 2o | 71 | 78 | 100 | 100 | 82 | 100 |
| 14 | 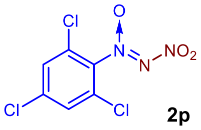 2p | 45 | 63 | 37 | 46 | 56 | 28 |
| 15 | 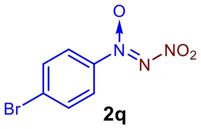 2q | 56 | 33 | 23 | 38 | 55 | 34 |
| 16 | 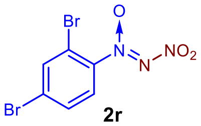 2r | 41 | 15 | 12 | 17 | 63 | 20 |
| 17 | 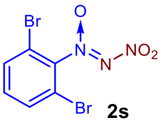 2s | 93 | 100 | 100 | 100 | 78 | 100 |
| 18 | 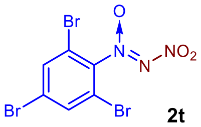 2t | 62 | 100 | 57 | 72 | 60 | 35 |
| 19 | Triadimefon 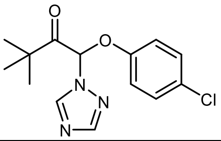 | 41 | 43 | 77 | 87 | 44 | 61 |

## 6. References


[1] Luk'yanov, O. A., Gorelik, V. P., Tartakovskii, V. A. Dinitramide and its salts. *Russ. Chem. Bull.* **1994**, *43* (1), 89–92. DOI: 10.1007/bf00699142

[2] Coleman, G. H., McCloskey, C. M., Stuart, F. A. Nitrosobenzene. *Organic Syntheses*. **1945**, *25*, 80–83. DOI: 10.15227/orgsyn.025.0080

[3] Hu, W., Yu, J.-T., Liu, S., Jiang, Y., Cheng, J. Copper-mediated annulation of 2-(1-arylvinyl) anilines and aryl nitrosos towards 2,3-diaryl-2*H*-indazoles. *Org. Chem. Front*. **2017**, *4* (1), 22–25. DOI: 10.1039/c6qo00540c

[4] Wang, Q., Li, X., Synthesis of 1*H*-Indazoles from Imidates and Nitrosobenzenes via Synergistic Rhodium/Copper Catalysis. *Org. Lett.* **2016**, *18* (9), 2102–2105. DOI: 10.1021/acs.orglett.6b00727

[5] Xiong, D., Wang, L., Jie, L., Yang, Z., Li, L., Cui, X. Rhodium-Catalyzed Mild C7-Amination of Indolines with Nitrosobenzenes. *ChemistrySelect*. **2018**, *3* (47), 13497–13500. DOI: 10.1002/slct.201803692

[6] Halasz, I., Biljan, I., Novak, P., Mestrovic, E., Plavec, J., Mali, G., Smrecki, V., Vancik, H., Cross-dimerization of nitrosobenzenes in solution and in solid state. *J. Mol. Struct.* **2009**, *918* (1), 19–25. DOI: 10.1016/j.molstruc.2008.07.035

[7] Dudek, M., Pakladek, Z., Deiana, M., Matcztszyn, K. Molecular design and structural characterization of photoresponsive azobenzene-based polyamide units. *Dyes Pigm.* **2020**, *180*, 108501. DOI: 10.1016/j.dyepig.2020.108501

[8] Roscales, S., Csaky, A. G., Synthesis of Di(hetero)arylamines from Nitrosoarenes and Boronic Acids: A General, Mild, and Transition-Metal-Free Coupling. *Org. Lett.* **2018**, *20* (6), 1667–1671. DOI: 10.1021/acs.orglett.8b00473

[9] Hu, W., Zheng, Q., Sun, S., Cheng, J. Catalyzed bilateral cyclization of aldehydes with nitrosos toward unsymmetrical acridines proceeding with C–H functionalization enabled by a transient directing group. *ChemComm.* **2017**, *53* (46), 6263–6266. DOI: 10.1039/c7cc03006a

[10] Yanagisawa, A., Lin, Y., Takeishi, A., Yoshida, K. Enantioselective Nitroso Aldol Reaction Catalyzed by a Chiral Phosphine–Silver Complex. *Eur. J. Org. Chem.,* **2016**, 2016 (32), 5355–5359. DOI: 10.1002/ejoc.201601143.

[11] Teders, M., Pogodaev, A. A.; Bojanov, G., Huck, W. T. S. Reversible Photoswitchable Inhibitors Generate Ultrasensitivity in Out-of-Equilibrium Enzymatic Reactions. *J. Am. Chem. Soc*. **2021**, 143 (15), p. 5709–5716. DOI: 10.1021/jacs.0c12956

[12] Hauwert, N. J., Mocking, T. A. M., Da Costa Pereira, D., Lion, K., Huppelschoten, Y., Vischer, H. F., De Esch, I. J. P.; Wijtmans, M., Leurs, R. A Photoswitchable Agonist for the Histamine H3 Receptor, a prototypic family A G protein-coupled receptor. *Angew. Chem. Int. Ed.* **2019**, *58* (14), 4531–4535. DOI: 10.1002/anie.201813110.

[13] Ge, Z., Yang, Z., Liang, J., Dong, D., Zhu, M. Optical Control of the GTP Affinity of K-Ras(G12C) by a Photoswitchable Inhibitor. *ChemBioChem.* **2019**, *20* (23), 2916–2920. DOI: 10.1002/cbic.201900342

[14] Holmes, R. R., Bayer, R. P. A simple method for the direct oxidation of aromatic amines to nitroso compounds. *J. Am. Chem. Soc.* **1960**, *82*, 3454–3456. DOI: 10.1021/ja01498a054

[15] Oubaha, H., Demitri, N., Rault-Berthelot, J., Dubois, P., Coulembier, O., Bonifazi, D Photoactive Boron–Nitrogen–Carbon Hybrids: From Azo-borazines to Polymeric Materials. *J. Org. Chem.* **2019**, *84* (14), 9101–9116. DOI: 10.1021/acs.joc.9b01046

[16] Ricardo, M. G., Schwark, M., Llanes, D., Niedermeyer, T. H. J., Westermann, B. Total Synthesis of Aetokthonotoxin, the Cyanobacterial Neurotoxin Causing Vacuolar Myelinopathy. *Chem. Eur. J.* **2021**, *27* (47), 12032–12035. DOI: 10.1002/chem.202101848

[17] Churakov, A. M., Ioffe, S. L., Tartakovsky, V. A. Synthesis of 1-Aryl-2-nitrodiazene 1-*N*-oxides. *Mendeleev Commun.* **1996**, 6, 20–22. DOI: 10.1070/MC1996v006n01ABEH000560.

[18] Churakov, A. M., Semenov, S. E., Ioffe, S. L., Strelenko, Y. A., Tartakovsky, V. A. A new approach to synthesis of 1-aryl-2-nitrozodiazene 1-N-oxides. *Russ. Chem. Bull.* **1997**, *46*, 1042–1043. DOI: 10.1007/BF02496149

[19] Dhingra, O. D., Sinclair, J. B. Basic Plant Pathology Methods; CRC Press: Boca Raton, FL, USA, 1985; ISBN 978-0-8493-5921-7.

[20] Xu, H.; Fan, L. Antifungal Agents. Part 4: Synthesis and Antifungal Activities of Novel Indole[1,2-c]-1,2,4-Benzotriazine Derivatives against Phytopathogenic Fungi in Vitro. *Eur. J. Med. Chem.* **2011**, *46*, 364–369. DOI:10.1016/j.ejmech.2010.10.022.

[21] Singh, P.K. Synthesis and Fungicidal Activity of Novel 3-(Substituted/Unsubstituted Phenylselenonyl)-1-Ribosyl/Deoxyribosyl-1 *H* -1,2,4-Triazole. *J. Agric. Food Chem.* **2012**, *60*, 5813–5818. DOI:10.1021/jf300730f.

[21] Itoh, H.; Kajino, H.; Tsukiyama, T.; Tobitsuka, J.; Ohta, H.; Takahi, Y.; Tsuda, M.; Takeshiba, H. Synthesis of Silicon-Containing Azole Derivatives with Magnesium Bromide Diethyl Etherate, and an Investigation of Their Fungicidal Activities. *Bioorg. Med. Chem.* **2002**, *10*, 4029–4034. DOI:10.1016/S0968-0896(02)00302-4.

[22] Popkov, S.V.; Kovalenko, L.V.; Bobylev, M.M.; Molchanov, O.Yu.; Krimer, M.Z.; Tashchi, V.P.; Putsykin, Y.G. The Synthesis and Fungicidal Activity of 2-Substituted 1-Azol-1-Ylmethyl-6-Arylidenecyclohexanols. *Pestic. Sci.* **1997**, *49*, 125–129. DOI:10.1002/(SICI)1096-9063(199702)49:2<125::AID-PS506>3.0.CO;2-0.

[23] Budnikov, A.S.; Lopat'eva, E.R.; Krylov, I.B.; Segida, O.O.; Lastovko, A.V.; Ilovaisky, A.I.; Nikishin, G.I.; Glinushkin, A.P.; Terent'ev, A.O. 4-Nitropyrazolin-5-Ones as Readily Available Fungicides of the Novel Structural Type for Crop Protection: Atom-Efficient Scalable Synthesis and Key Structural Features Responsible for Activity. *J. Agric. Food Chem.* **2022**, *70*, 4572–4581. DOI:10.1021/acs.jafc.1c07413.

[24] Budnikov, A.S.; Krylov, I.B.; Shevchenko, M.I.; Segida, O.O.; Lastovko, A.V.; Alekseenko, A.L.; Ilovaisky, A.I.; Nikishin, G.I.; Terent'ev, A.O. C–O Coupling of Hydrazones with Diacetyliminoxyl Radical Leading to Azo Oxime Ethers—Novel Antifungal Agents. *Molecules* **2023**, *28*, 7863. DOI:10.3390/molecules28237863.

## 7. NMR Data

### 7.1.1 $^{1}H$ NMR spectrum of compound 2a [500.13 MHz, $CDCl_3$]

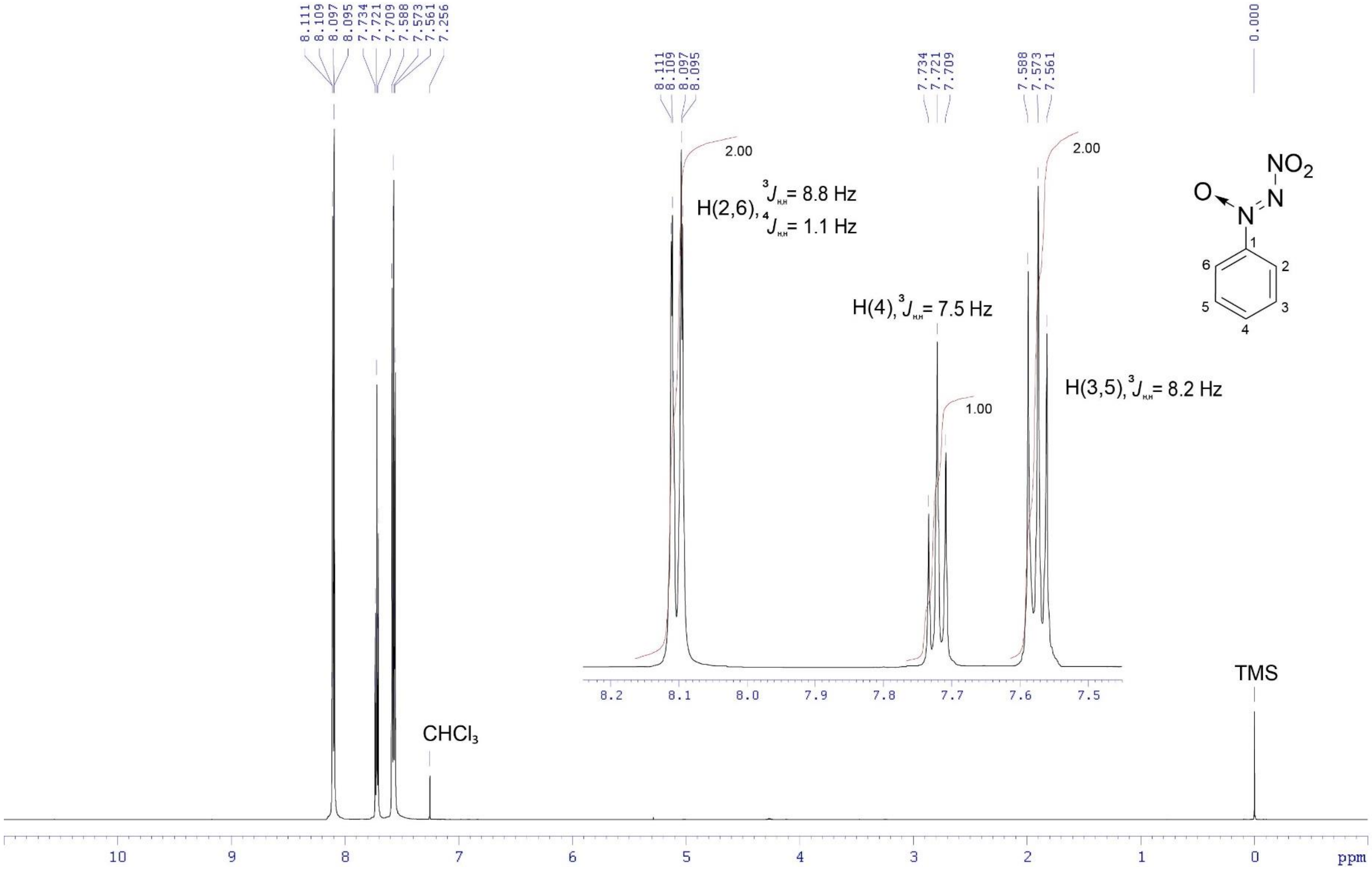

### 7.1.2 $^{13}$C NMR spectrum of compound 2a [125.76 MHz, $CDCl_3$]

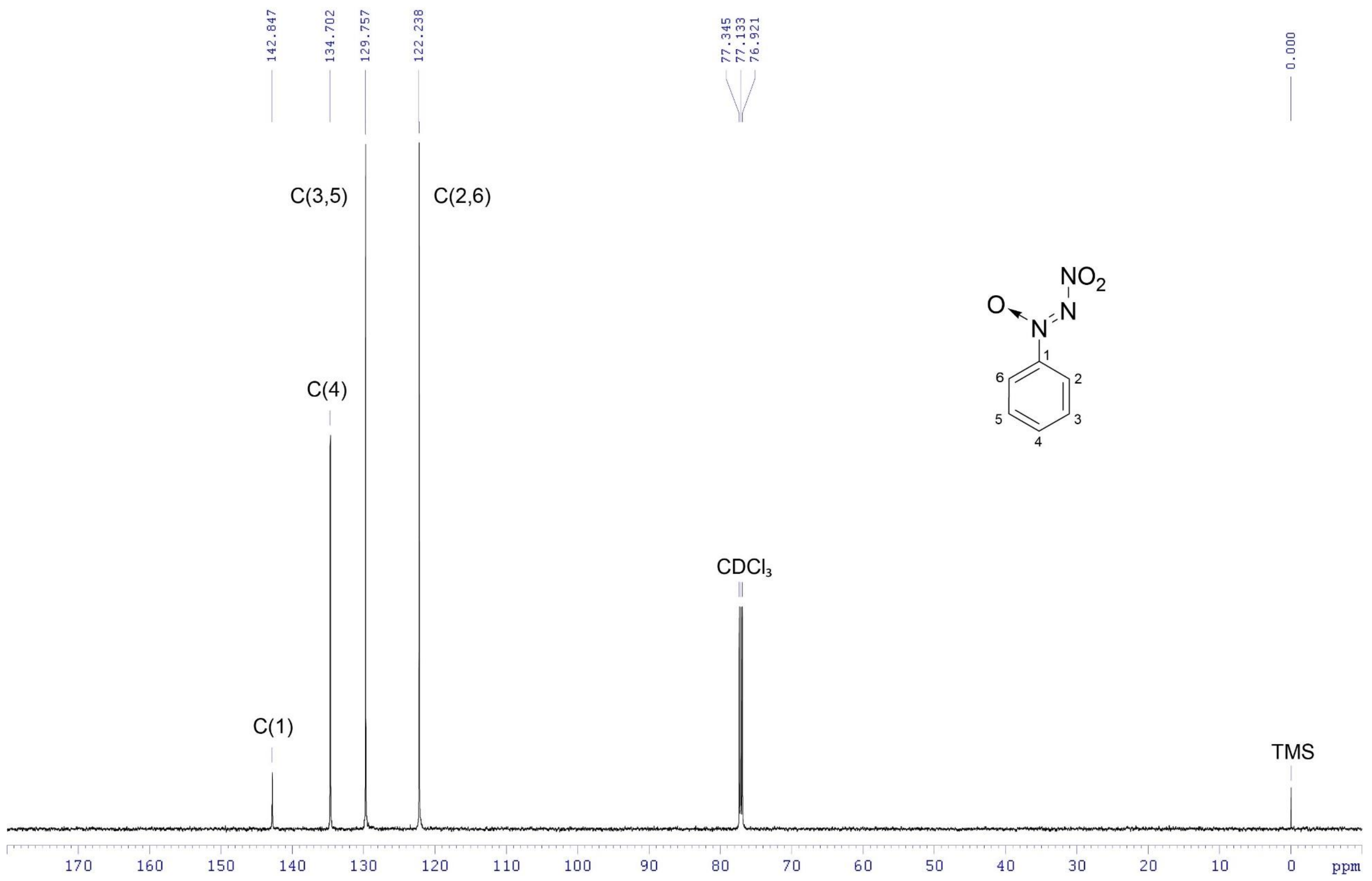

### 7.1.3 {$^1$H–$^{13}$C} HSQC spectrum of compound 2a [500.13 MHz, $CDCl_3$]

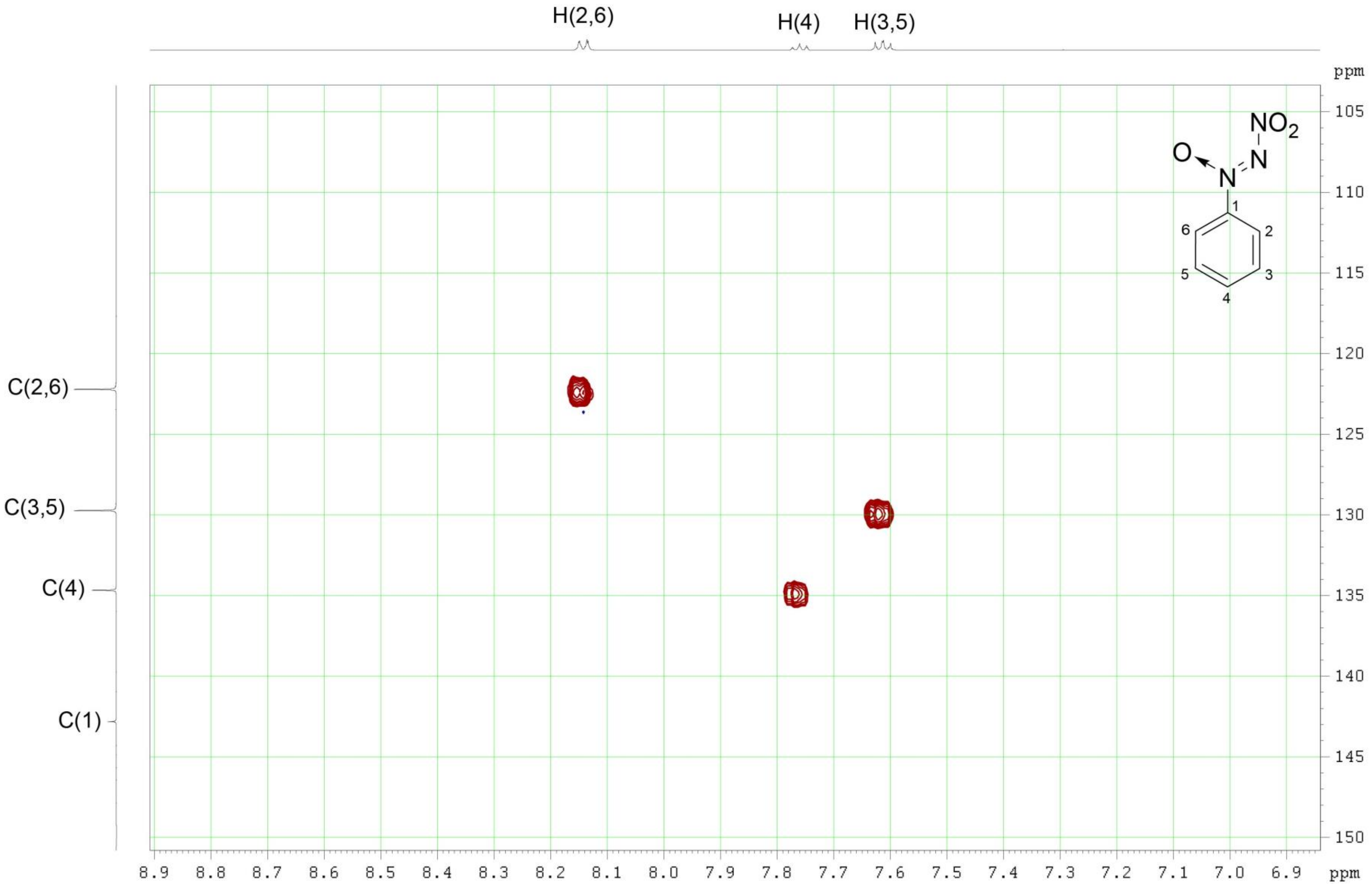

### 7.1.4 {$^1$H–$^{13}$C} HMBC spectrum of compound 2a [500.13 MHz, $CDCl_3$]

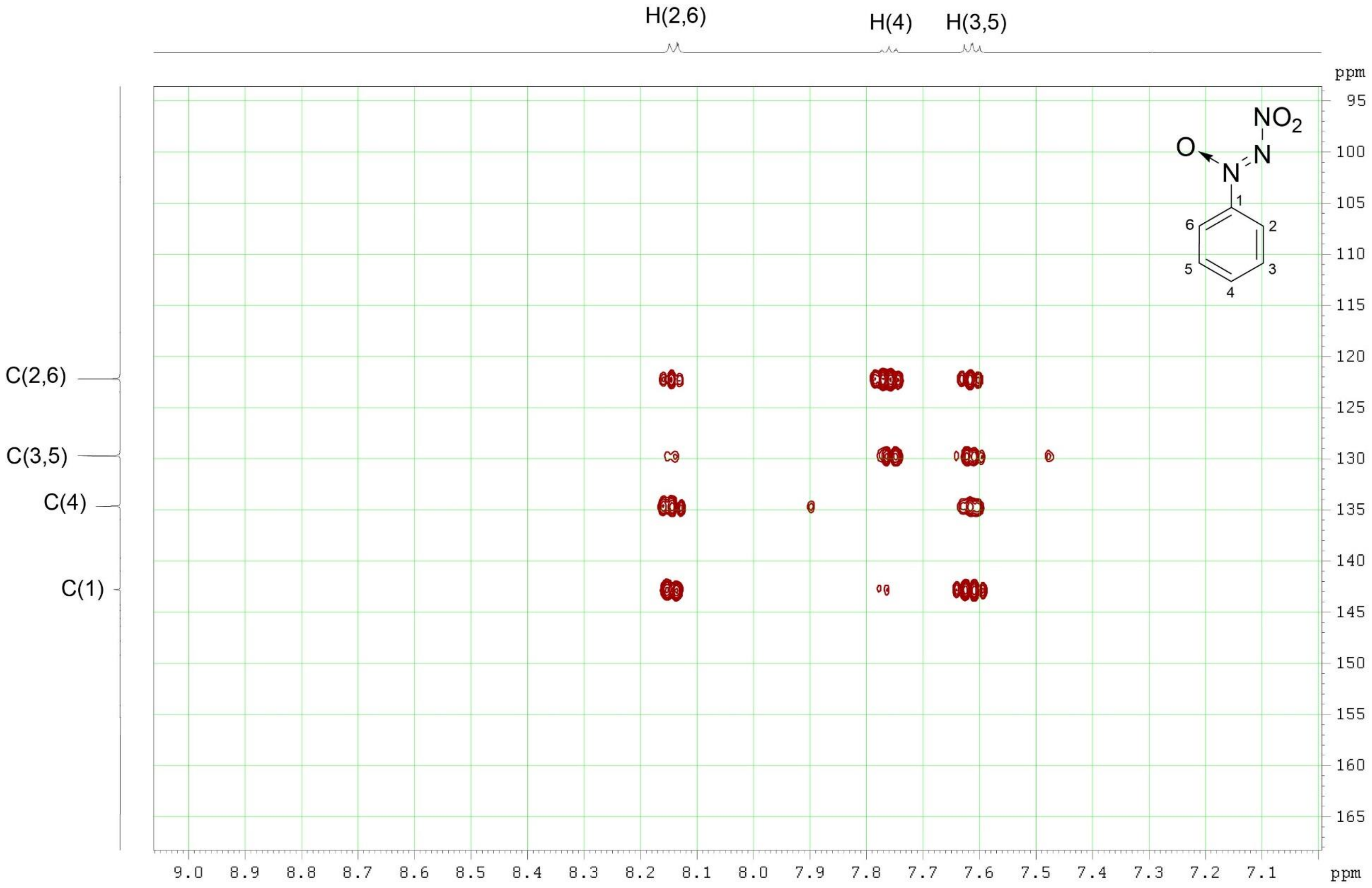

### 7.1.5 $^{14}$N NMR spectrum of compound 2a [36.14 MHz, $CDCl_3$]

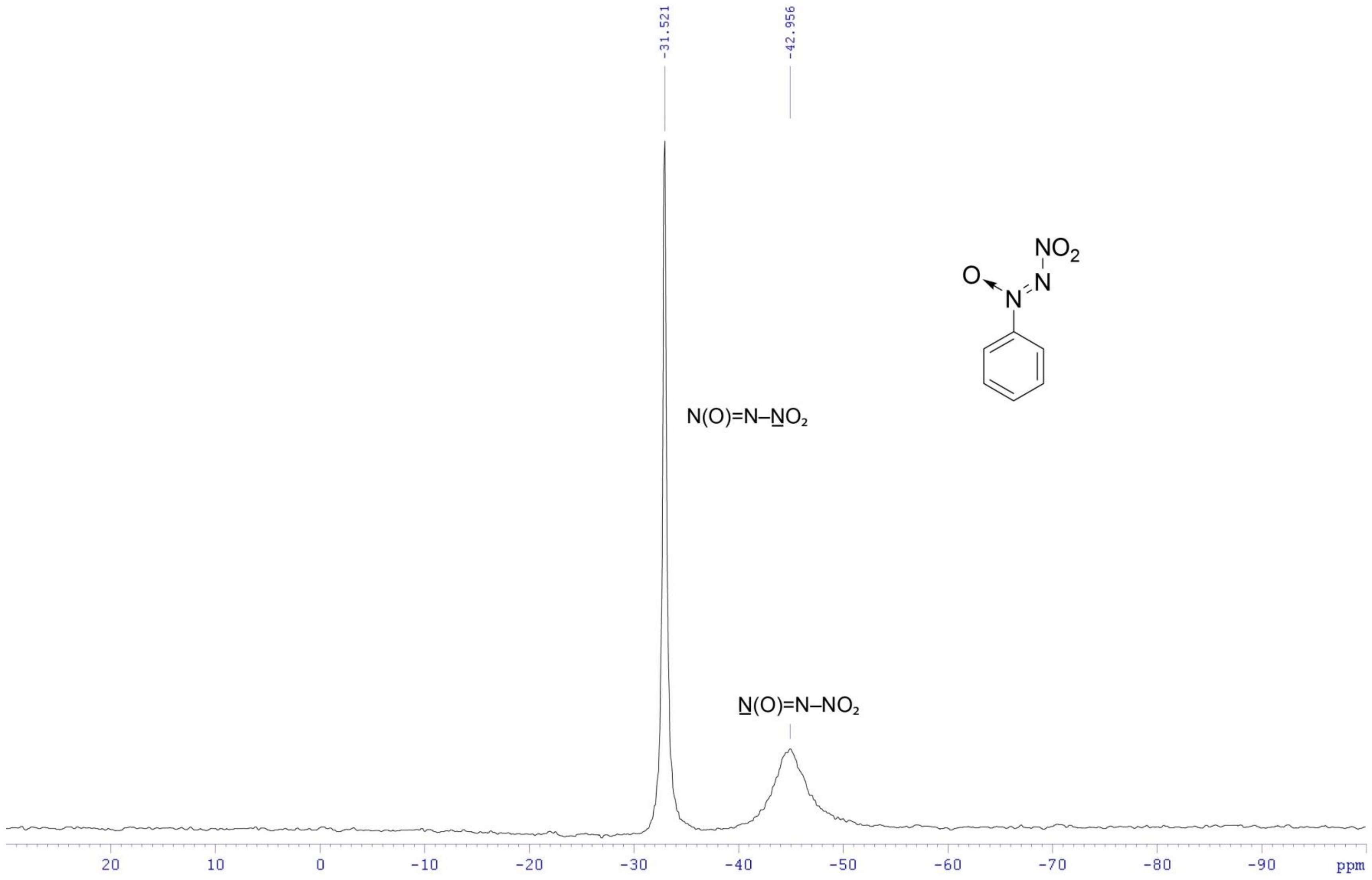

### 7.2.1 $^{1}$H NMR spectrum of compound 2b [500.13 MHz, $CDCl_3$]

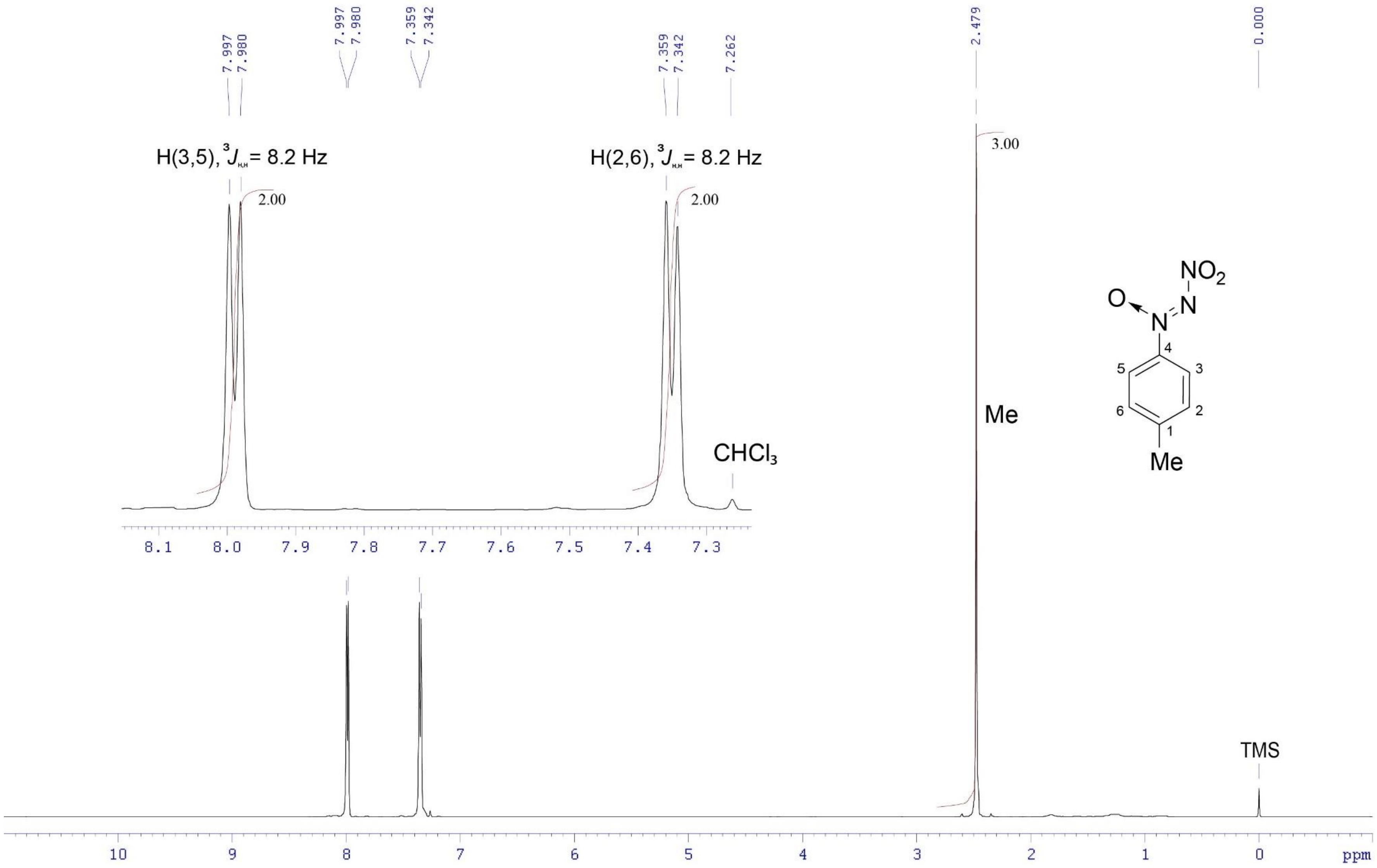

### 7.2.2 $^{13}$C NMR spectrum of compound 2b [125.76 MHz, $CDCl_3$]

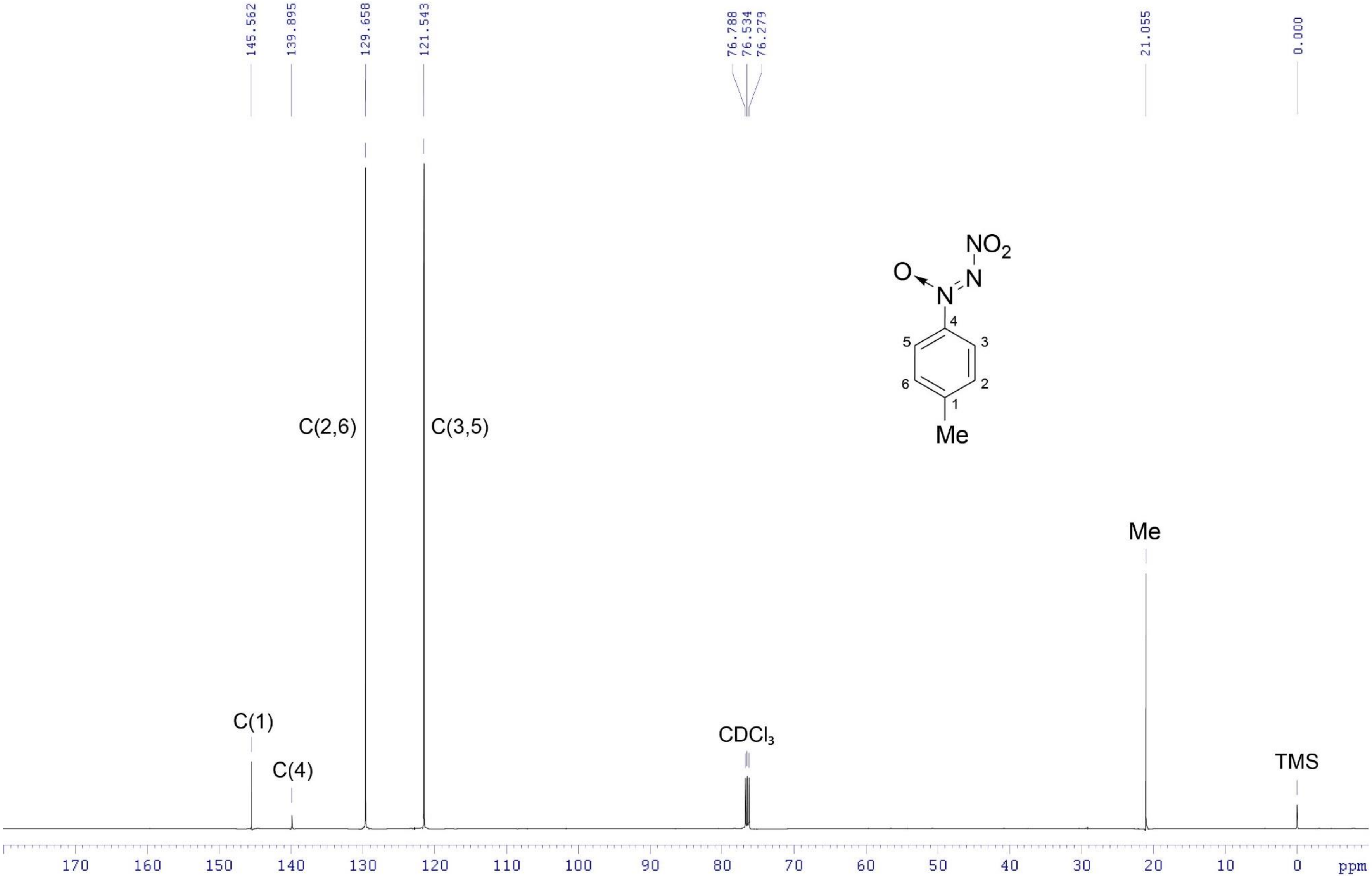

7.2.3 {$^{1}$H–$^{13}$C} HSQC spectrum of compound 2b [500.13 MHz, $CDCl_3$]

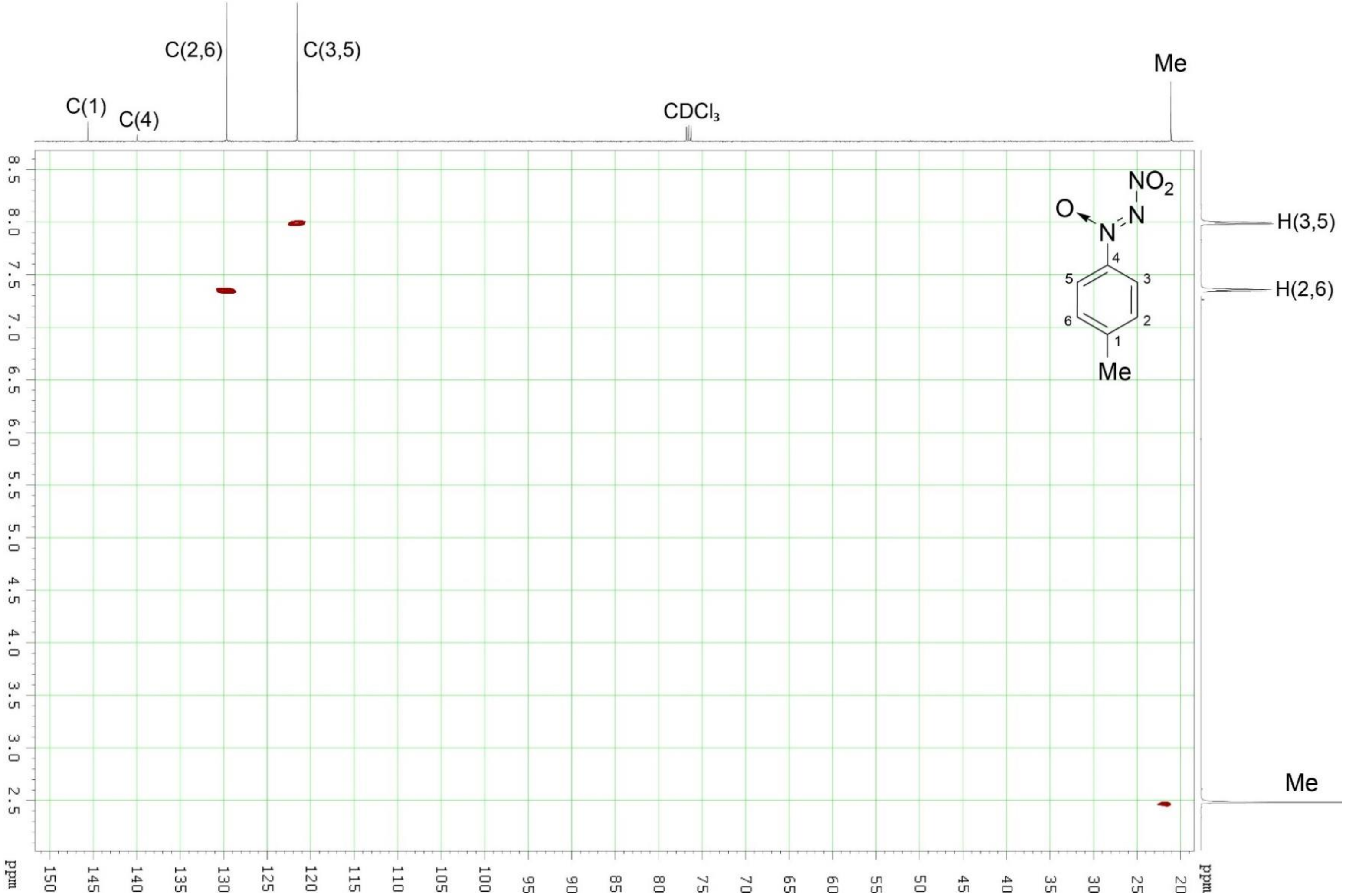

7.2.4 {$^1$H–$^{13}$C} HMBC spectrum of compound 2b [500.13 MHz, $CDCl_3$]

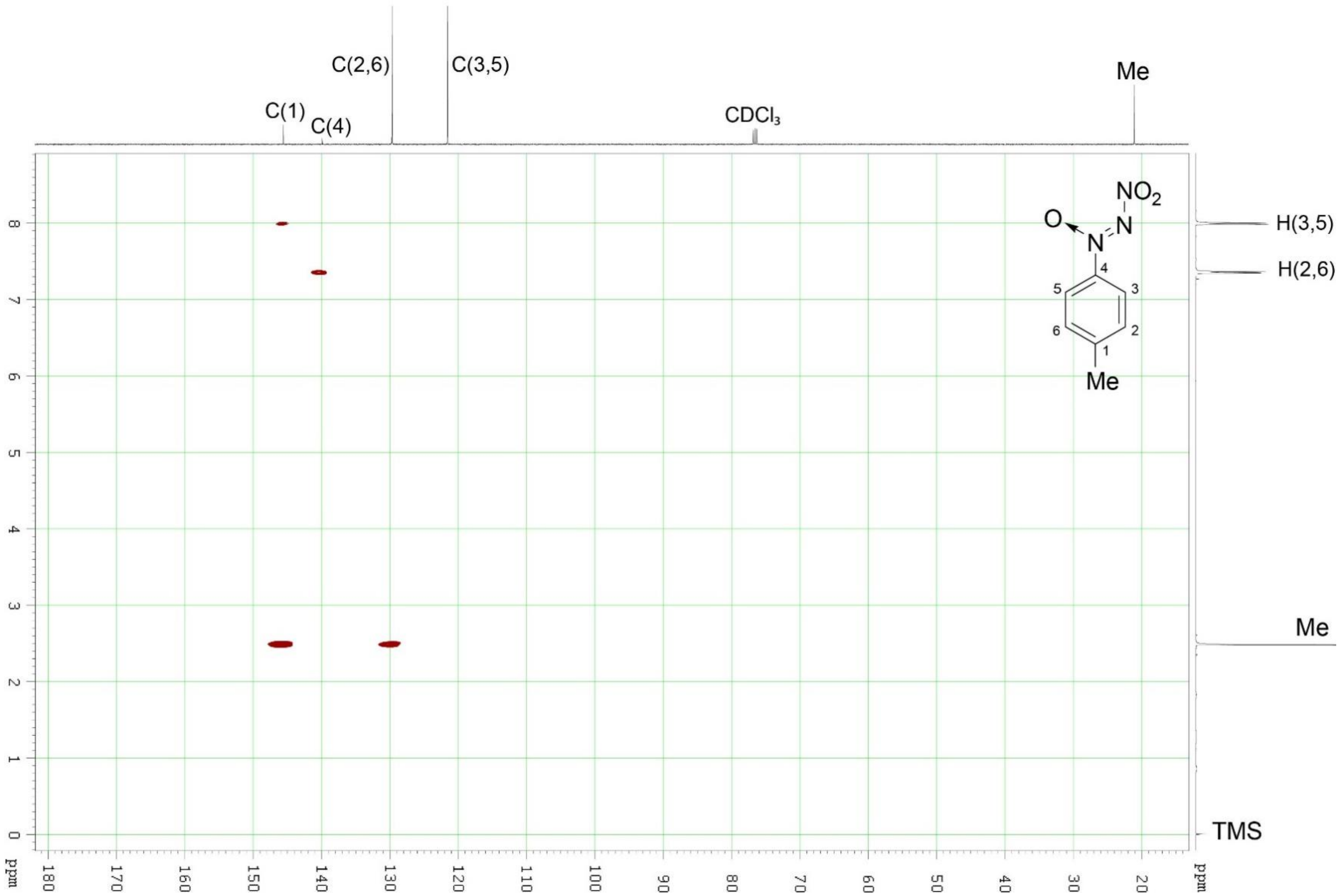

### 7.2.5 $^{14}$N NMR spectrum of compound 2b [36.14 MHz, $CDCl_3$]

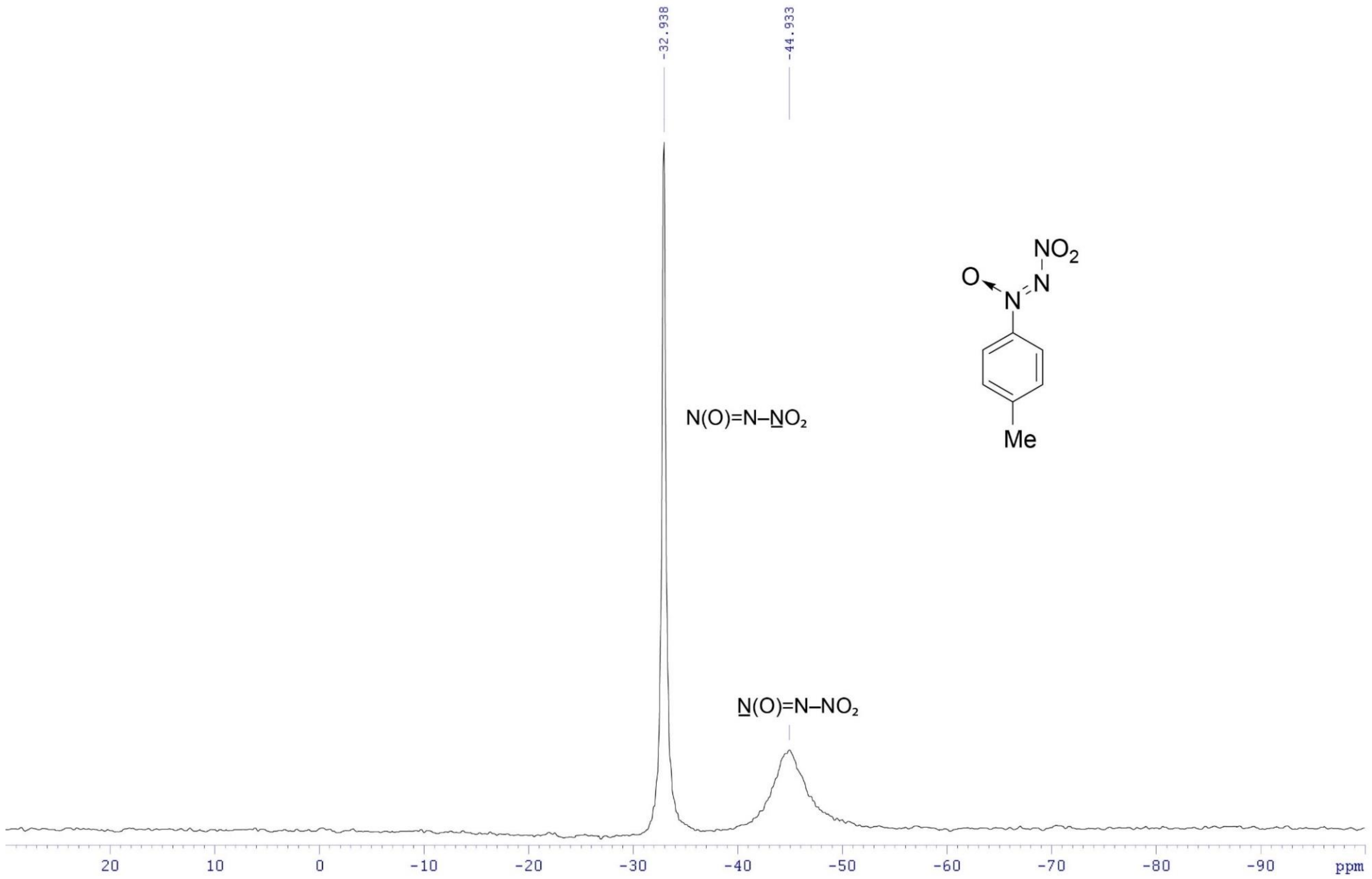

### 7.3.1 $^{1}$H NMR spectrum of compound 2c [500.13 MHz, $CDCl_3$]

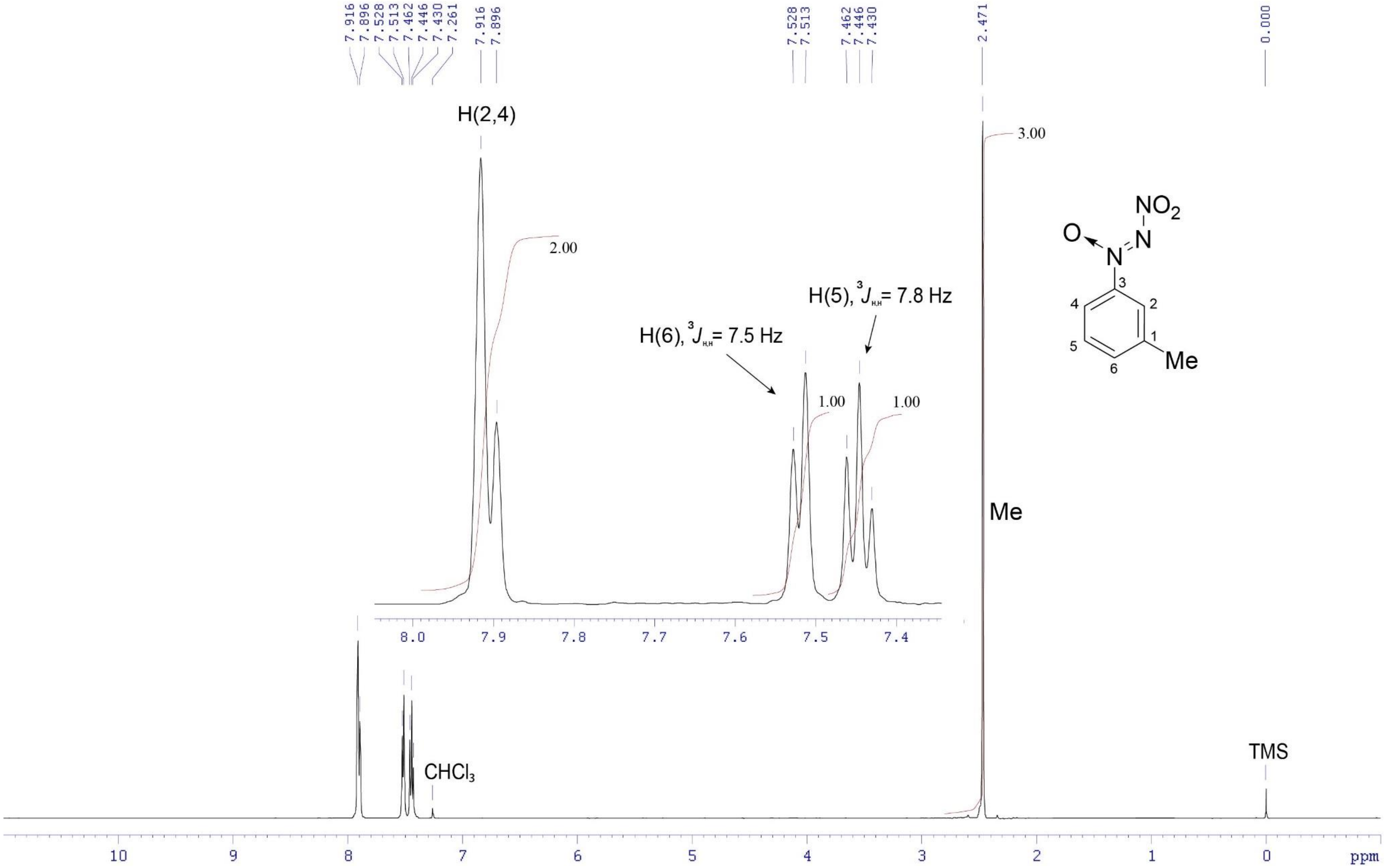

### 7.3.2 $^{13}C$ NMR spectrum of compound 2c [125.76 MHz, $CDCl_3$]

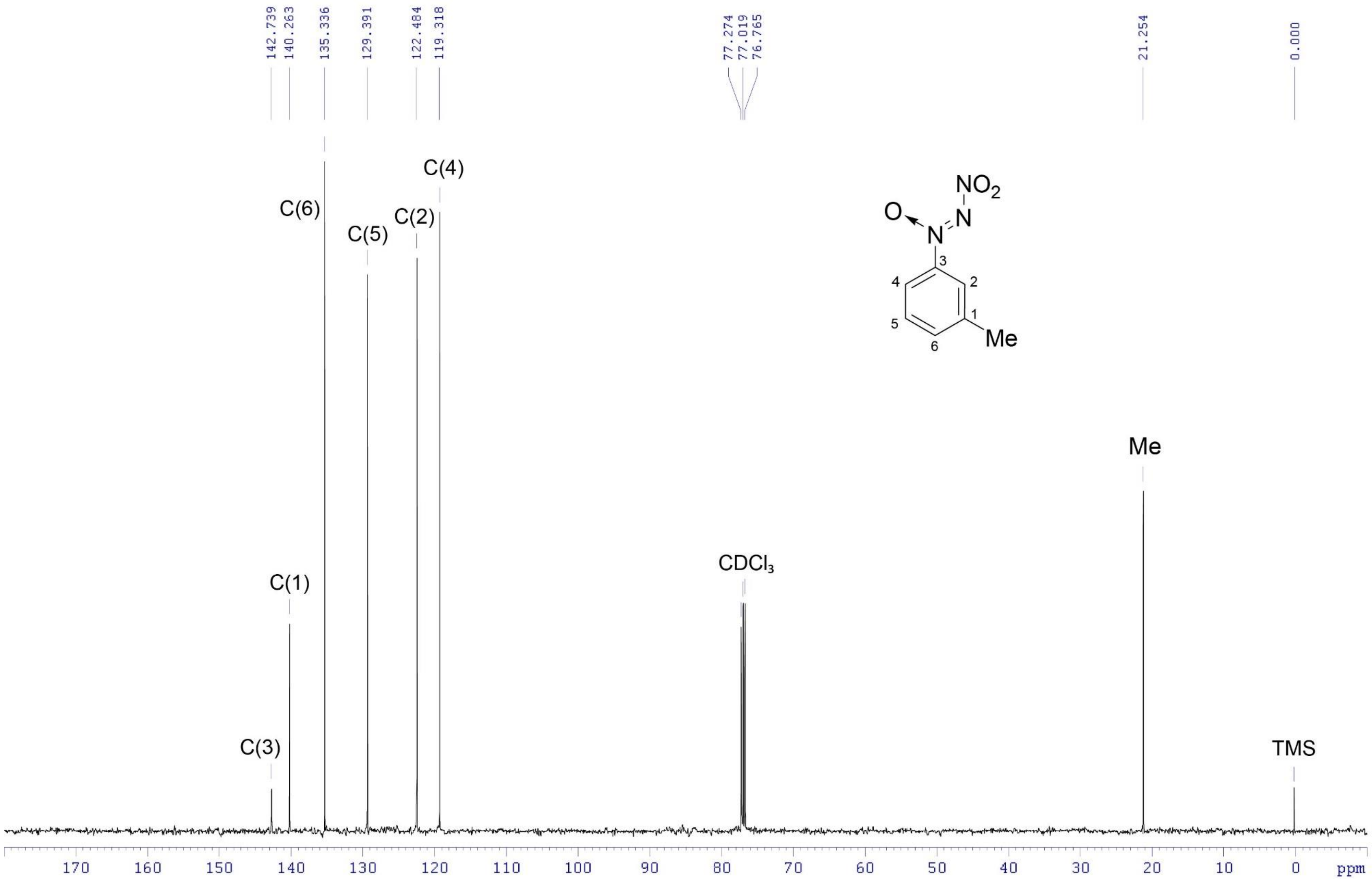

### 7.3.3 {$^1H$–$^{13}C$} HSQC spectrum of compound 2c [500.13 MHz, $CDCl_3$]

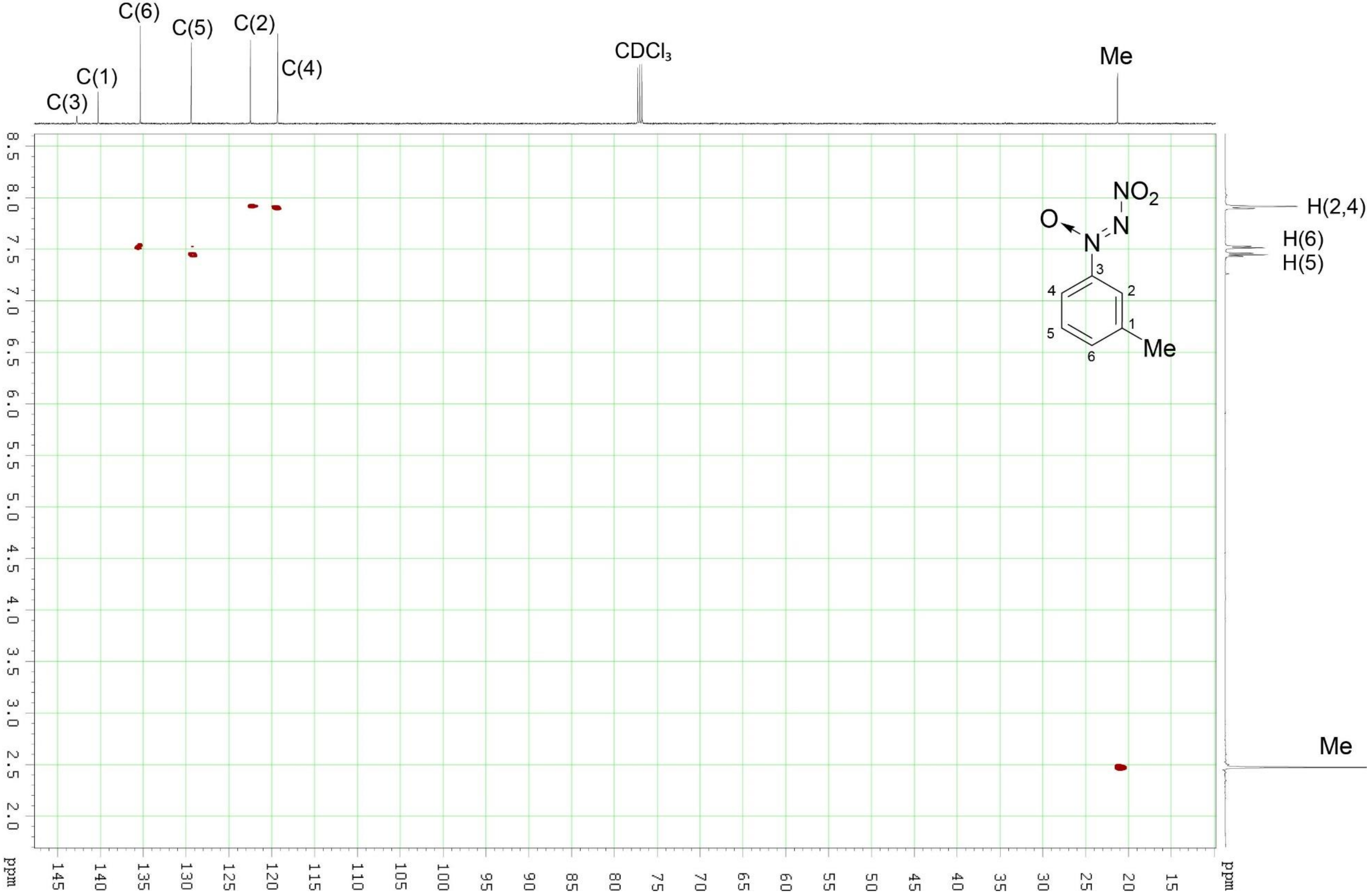

### 7.3.4 {$^{1}$H–$^{13}$C} HMBC spectrum of compound 2c [500.13 MHz, $CDCl_3$]

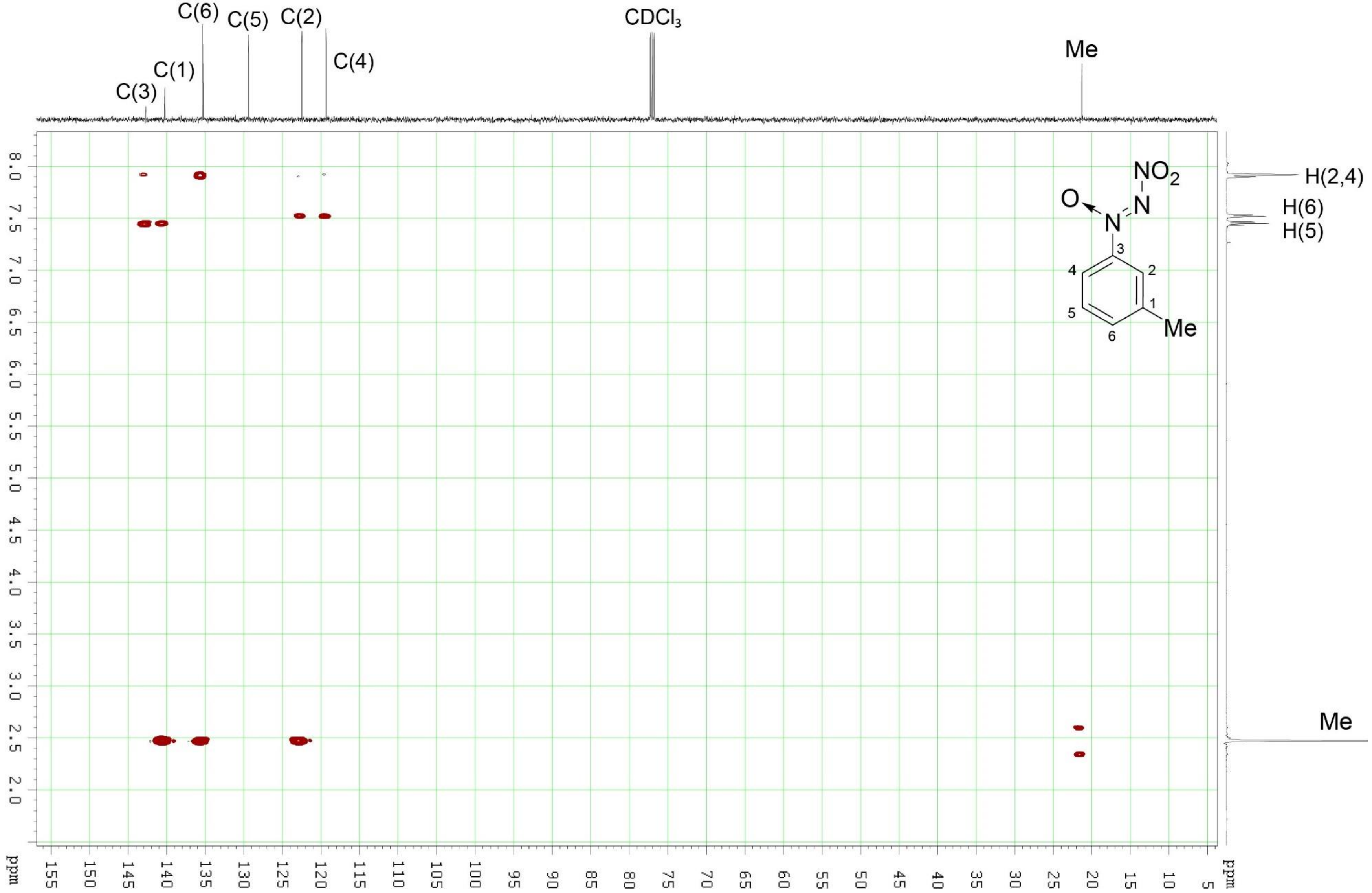

**7.3.5 $^{14}N$ NMR spectrum of compound 2c [36.14 MHz, $CDCl_3$]**

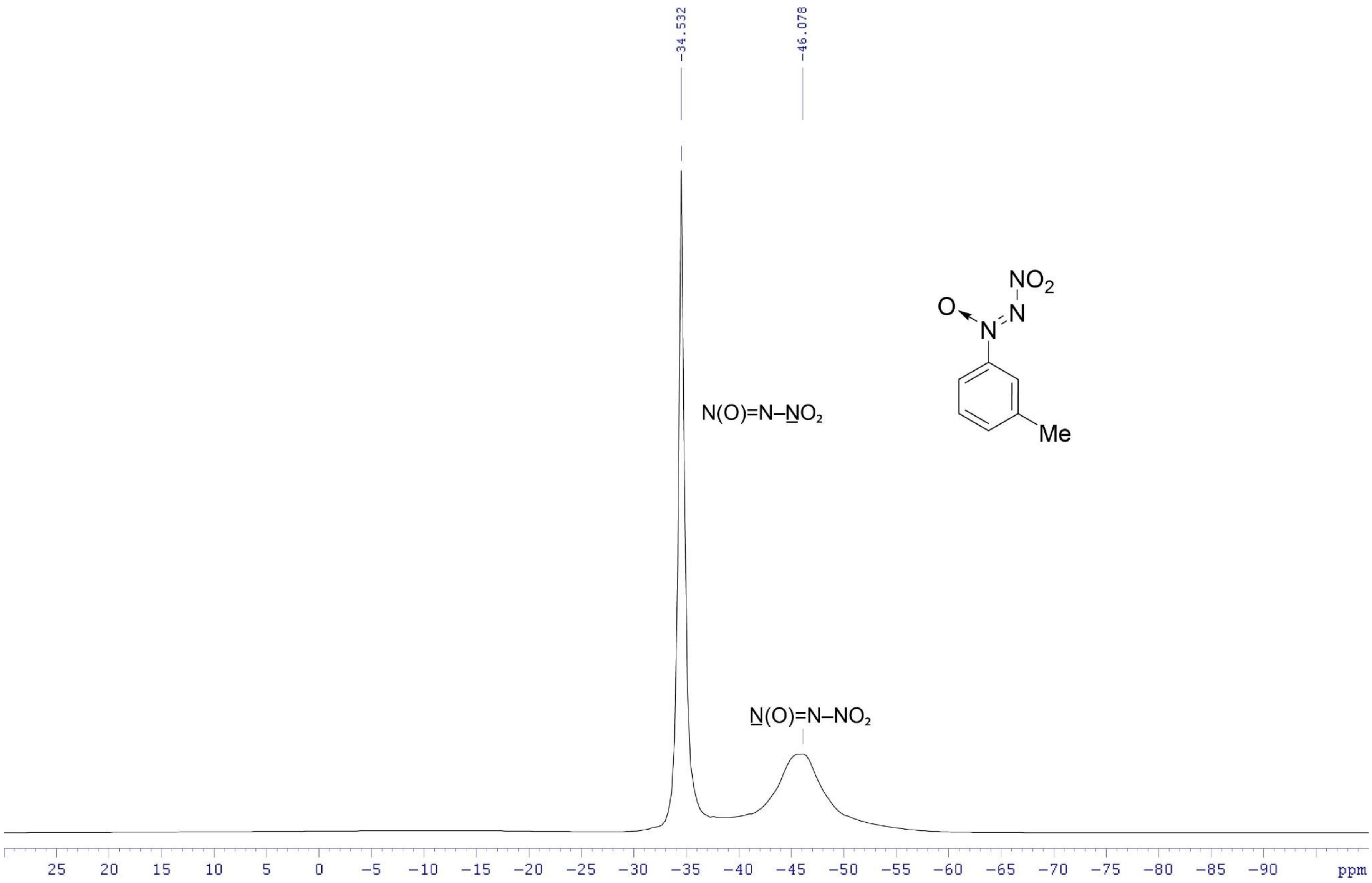

### 7.4.1 $^1$H NMR spectrum of compound 2d [600.13 MHz, $CDCl_3$]

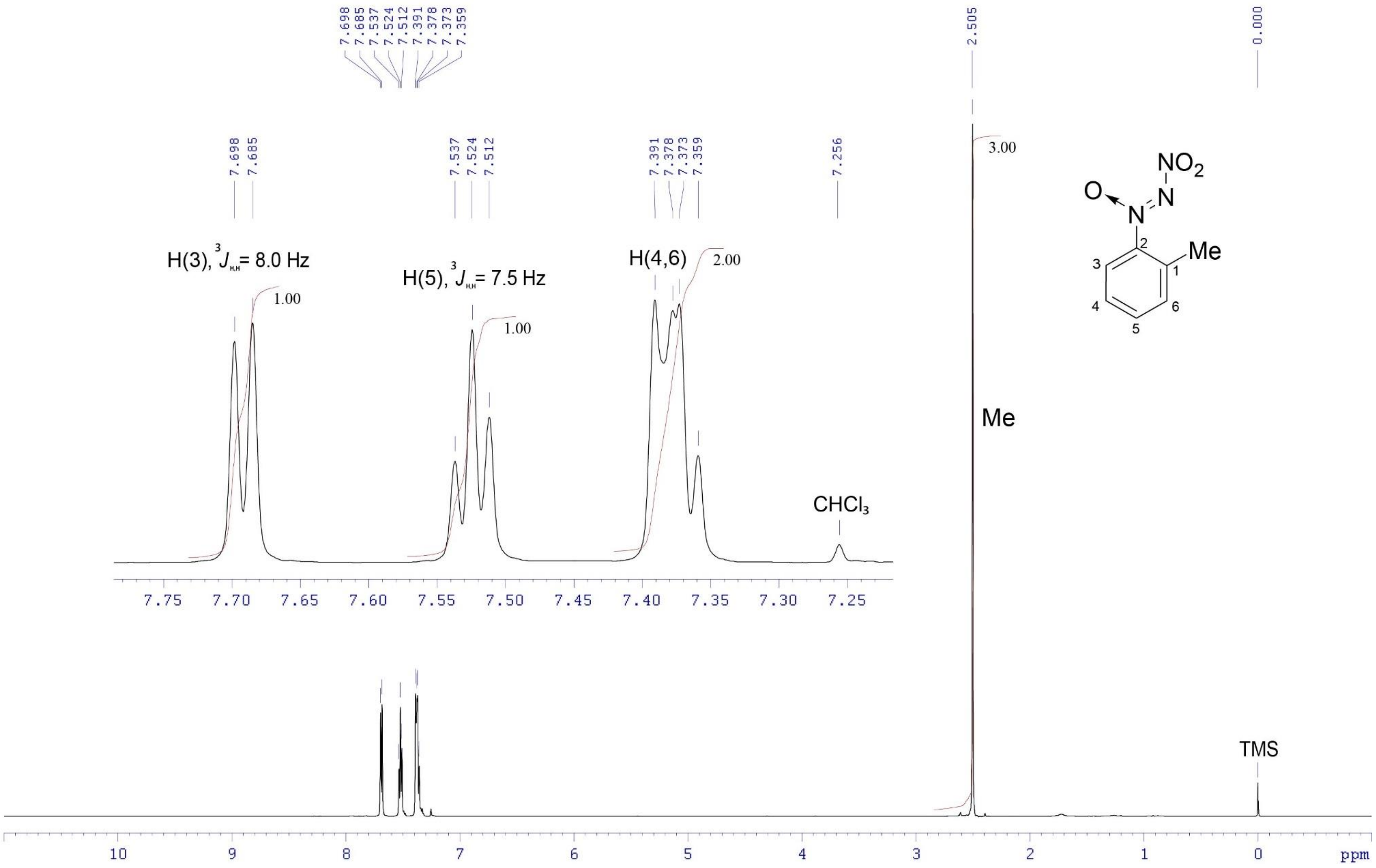

### 7.4.2 $^{13}$C NMR spectrum of compound 2d [150.90 MHz, $CDCl_3$]

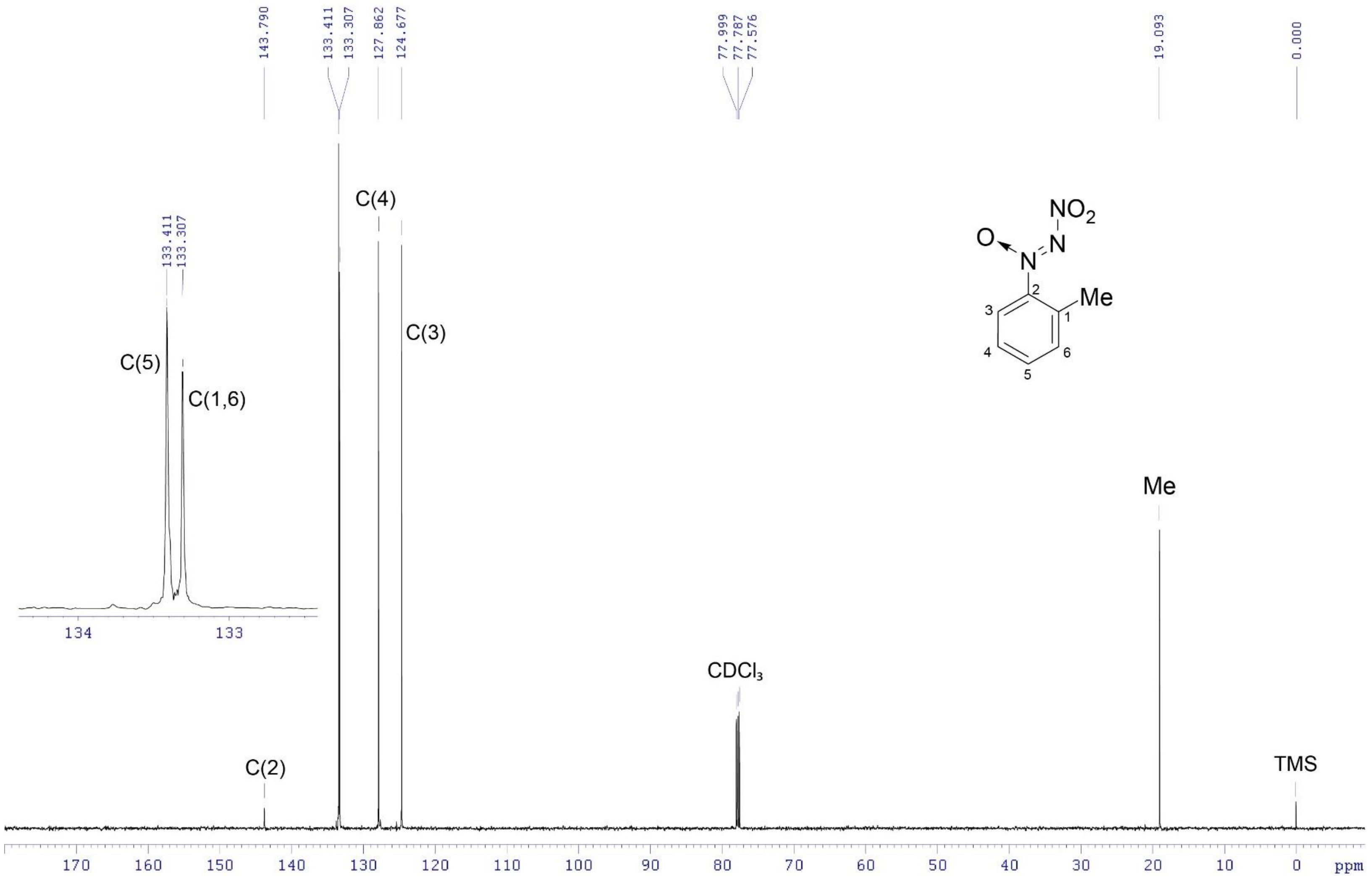

### 7.4.3 {$^1H$–$^{13}C$} HSQC spectrum of compound 2d [600.13 MHz, $CDCl_3$]

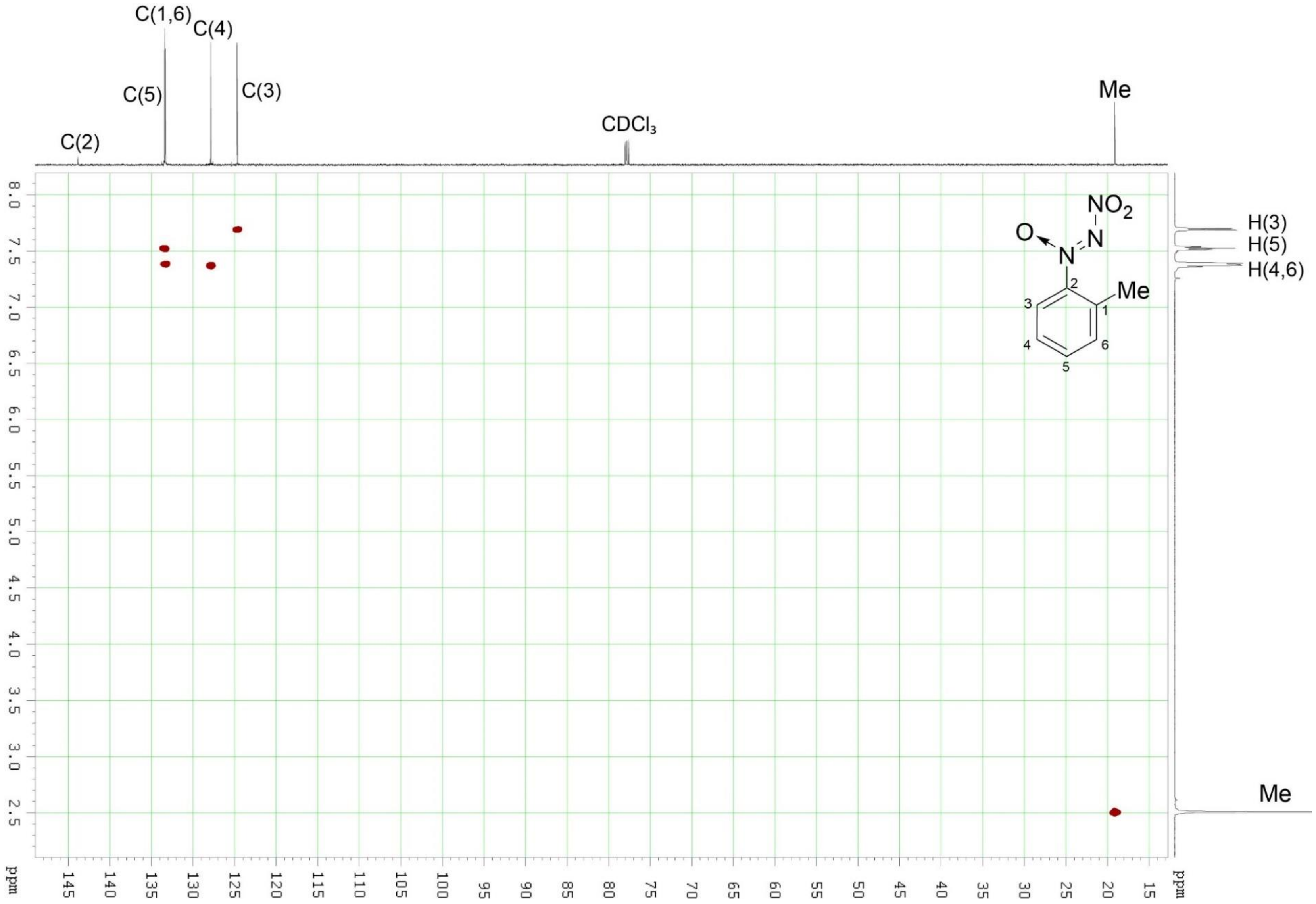

### 7.4.4 {$^{1}H–^{13}C$} HMBC spectrum of compound 2d [600.13 MHz, $CDCl_3$]

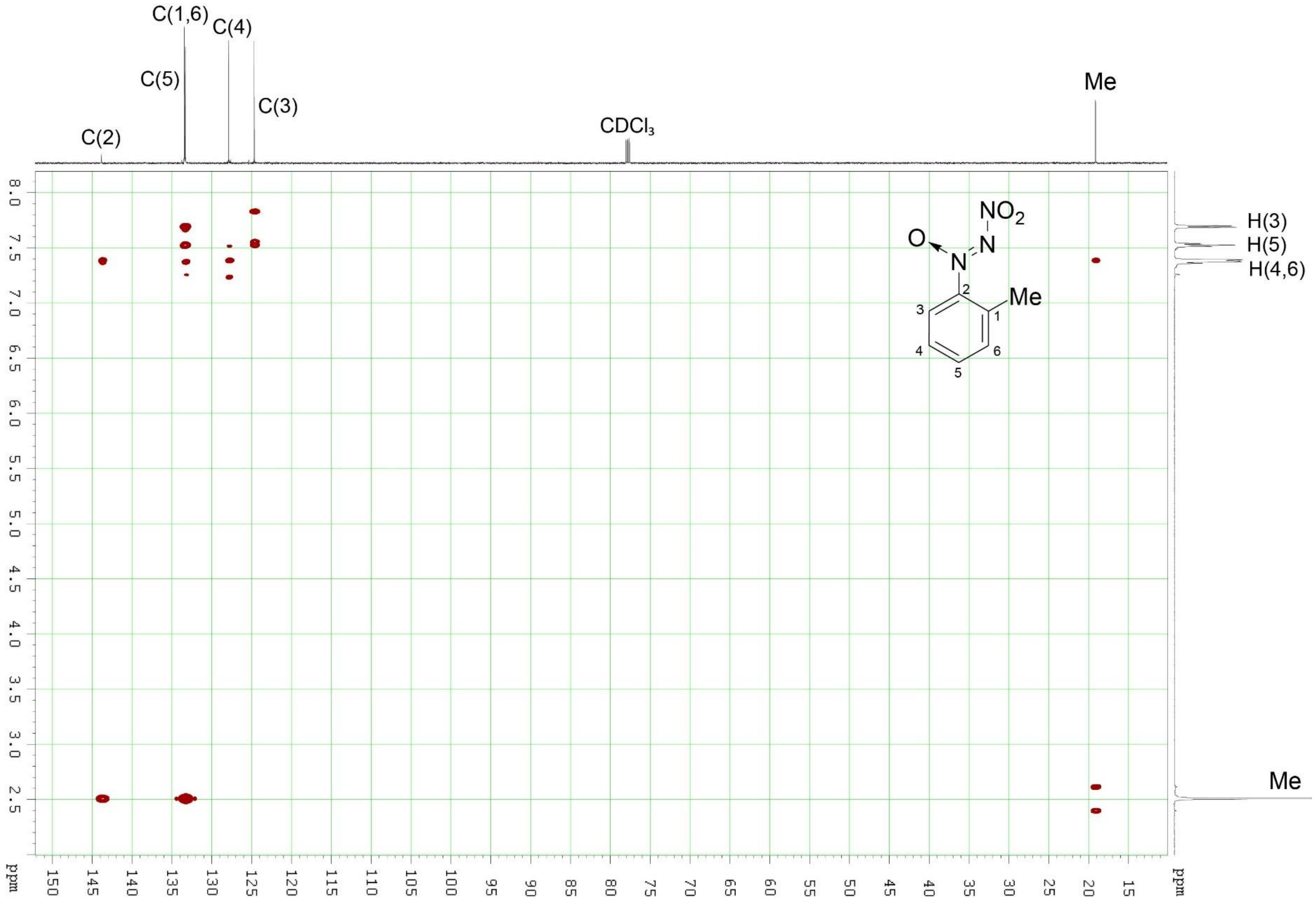

### 7.4.5 $^{14}N$ NMR spectrum of compound 2d [43.37 MHz, $CDCl_3$]

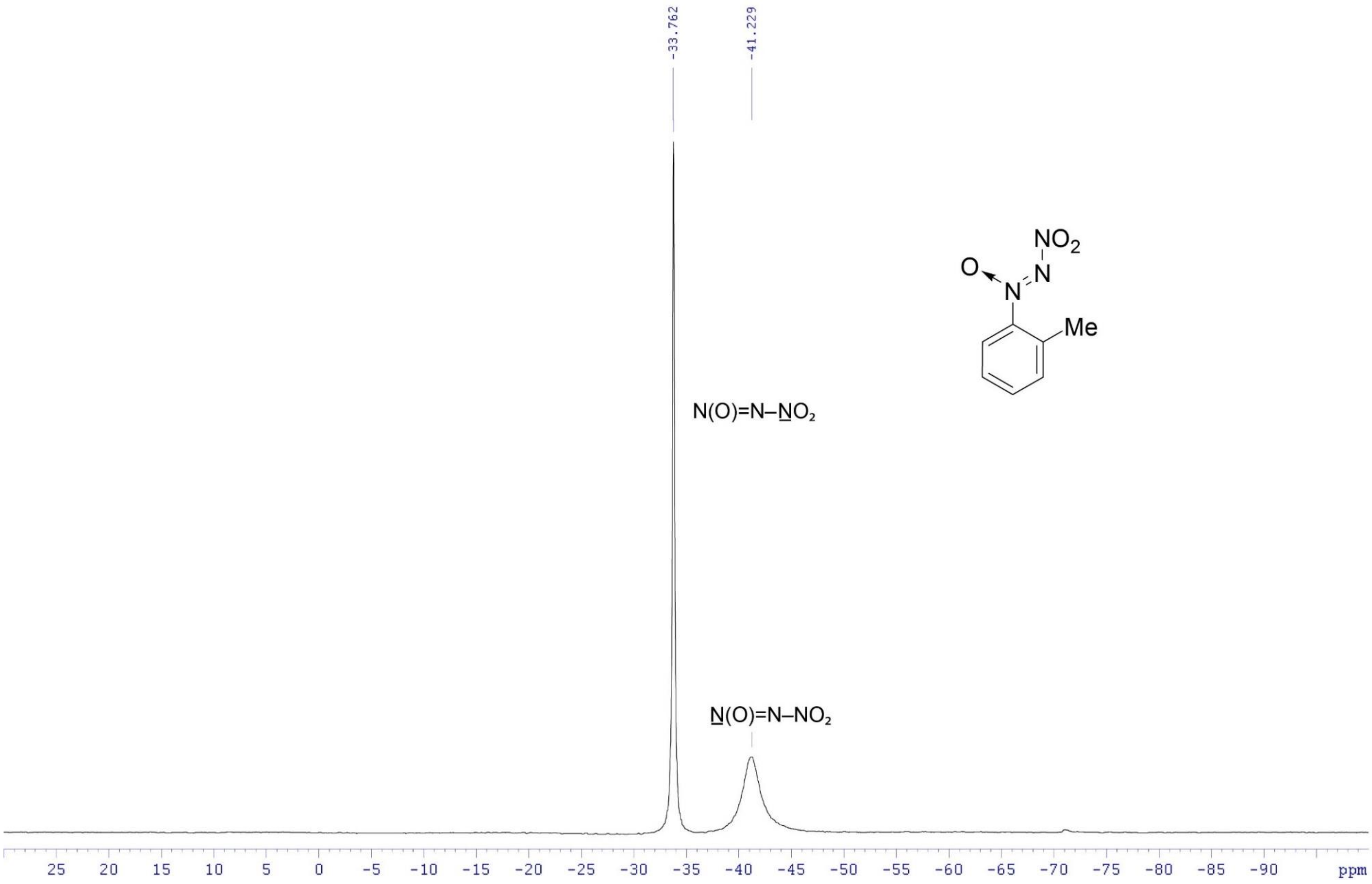

### 7.5.1 $^1$H NMR spectrum of compound 2e [500.13 MHz, $CDCl_3$]

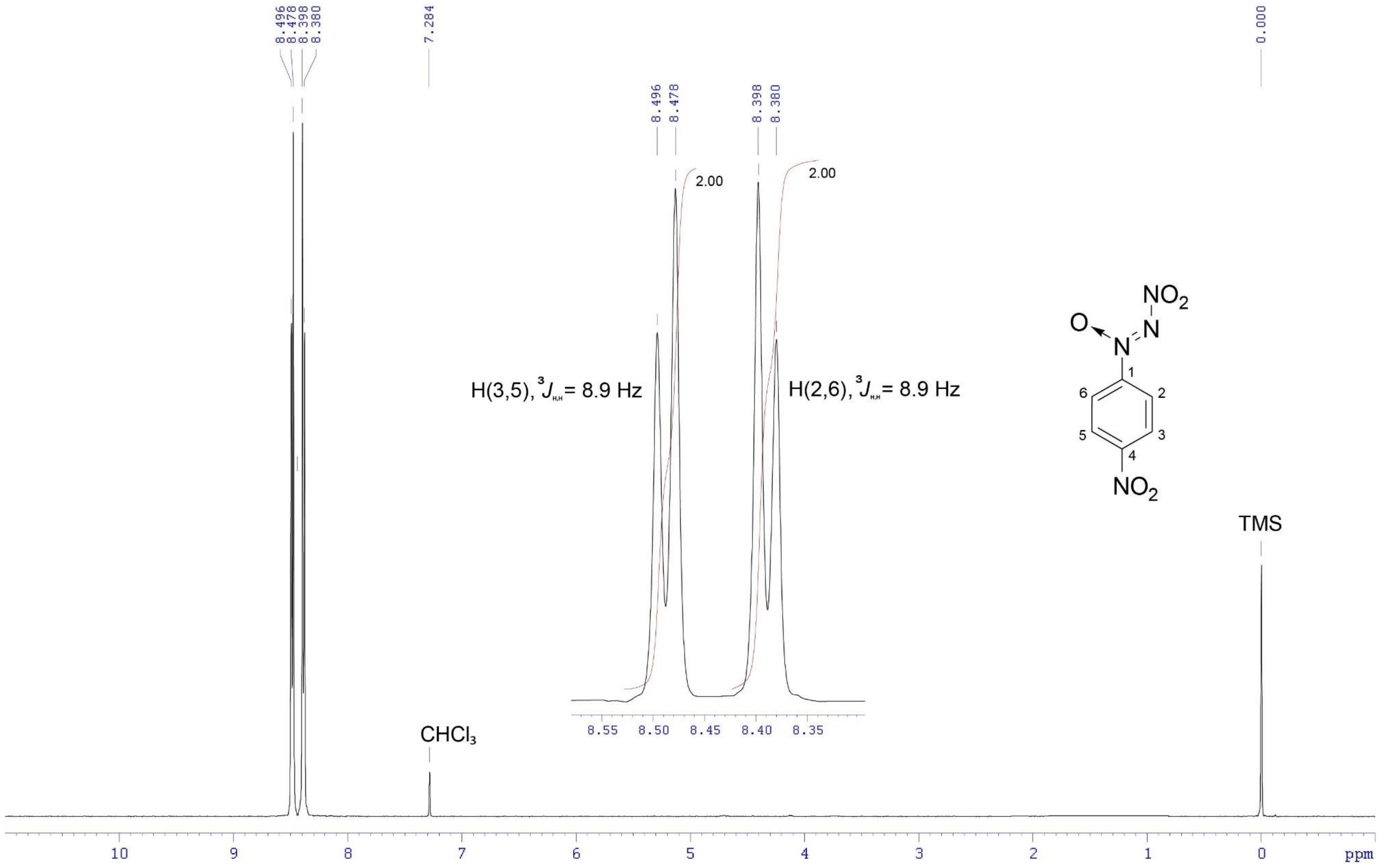

### 7.5.2 $^{13}C$ NMR spectrum of compound 2e [125.76 MHz, $CDCl_3$]

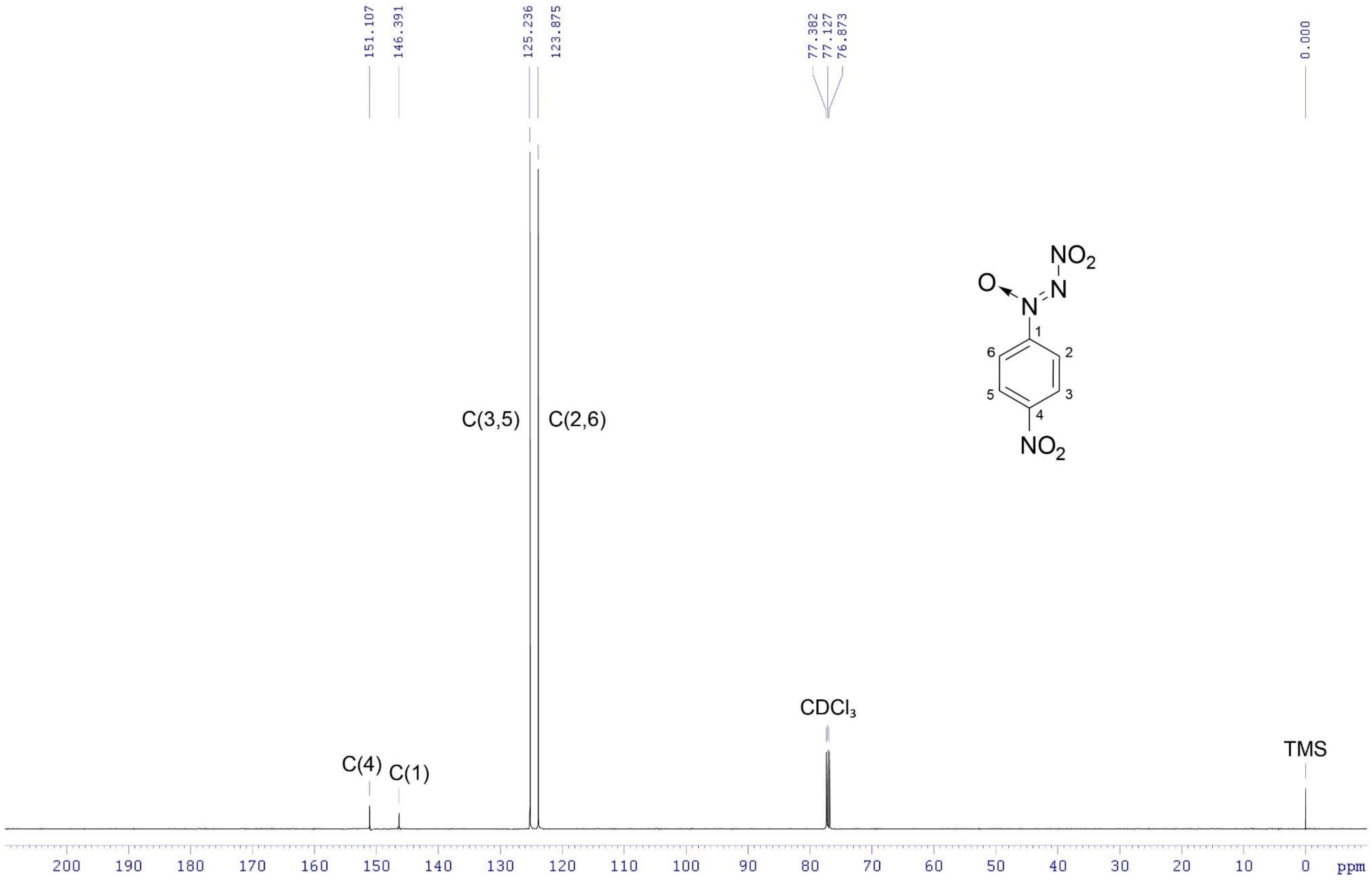

### 7.5.3 {$^{1}H$–$^{13}C$} HSQC spectrum of compound 2e [500.13 MHz, $CDCl_3$]

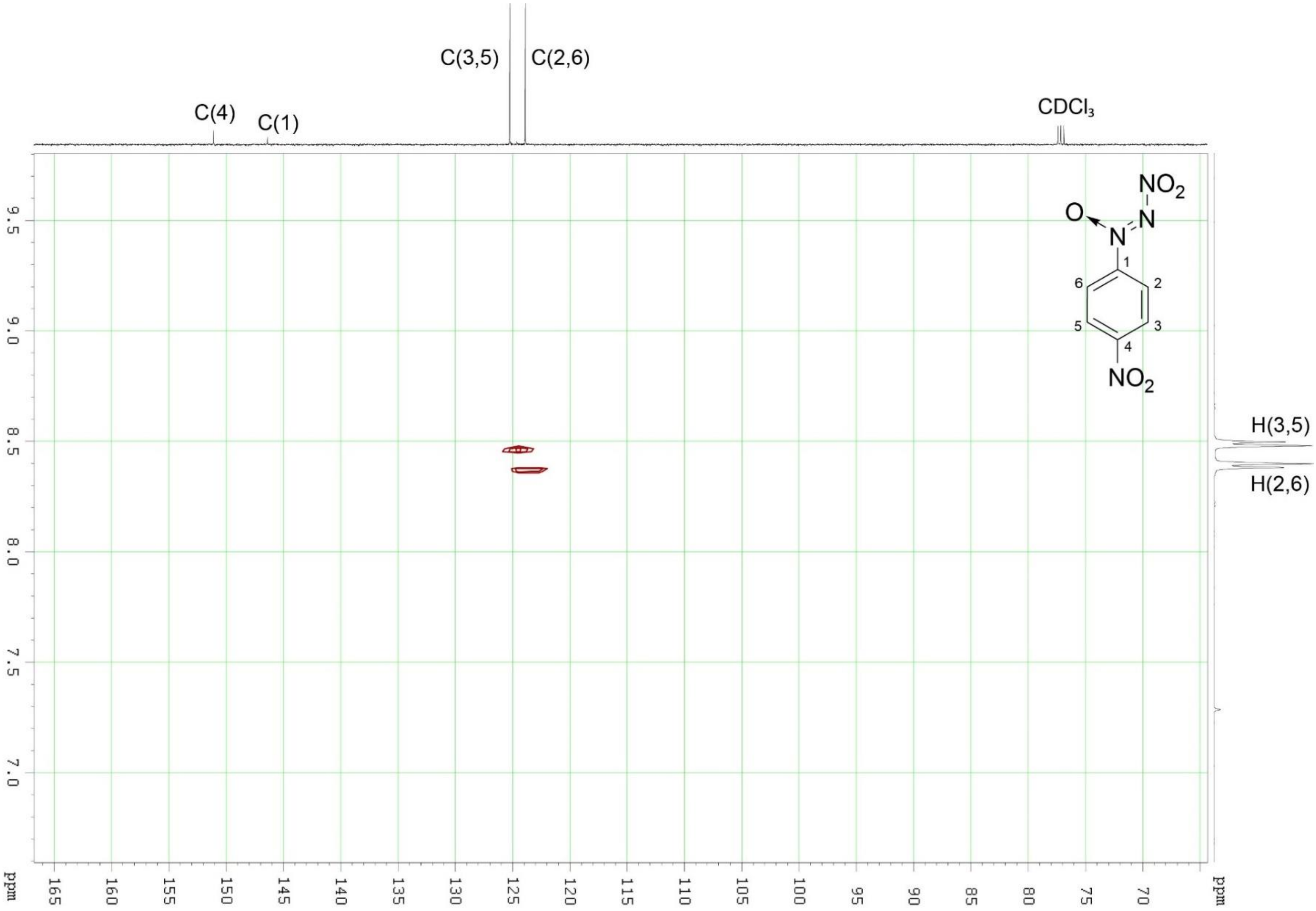

### 7.5.4 {$^1$H–$^{13}$C} HMBC spectrum of compound 2e [500.13 MHz, $CDCl_3$]

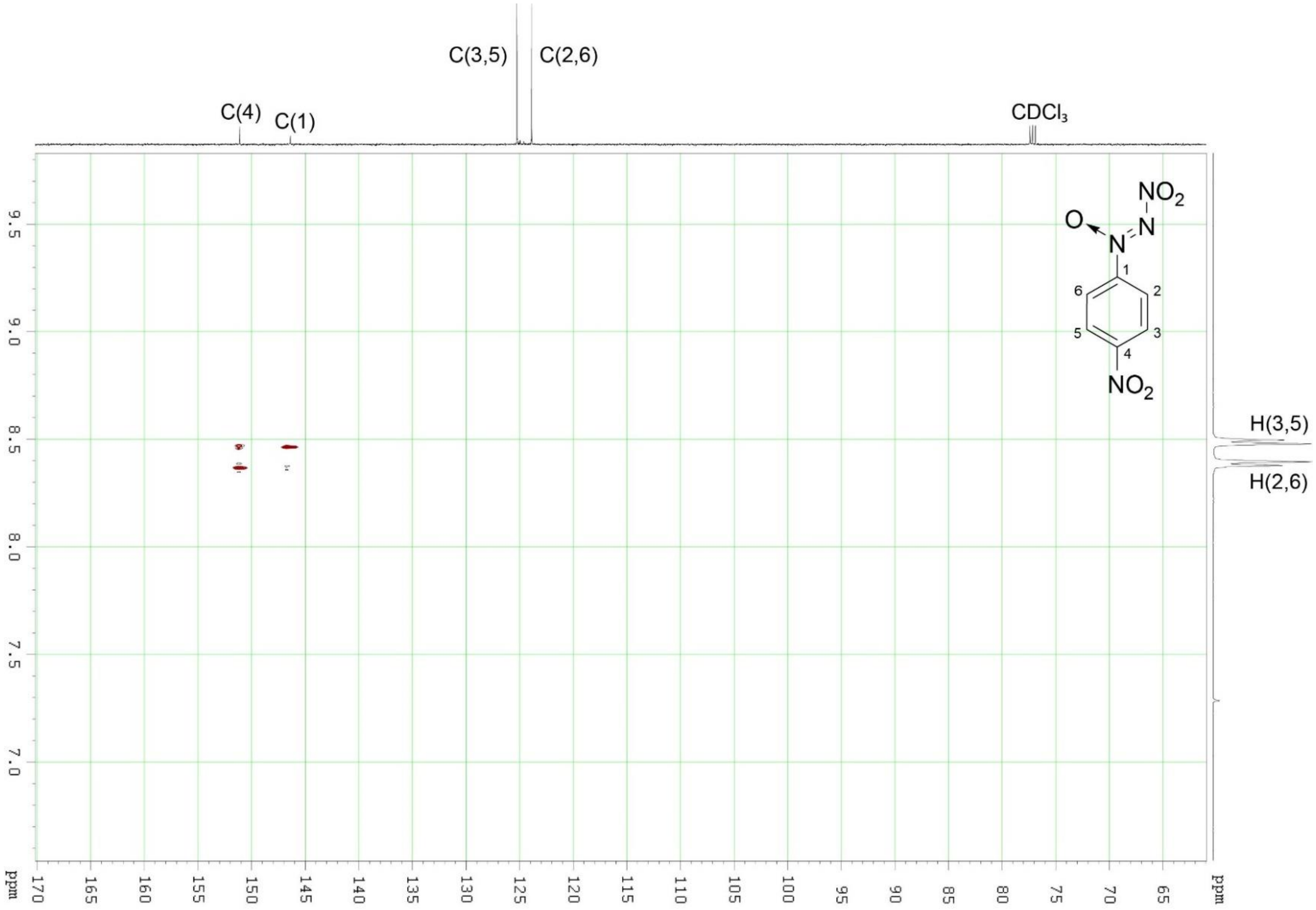

7.5.5 ¹⁴N NMR spectrum of compound 2e [36.14 MHz, $CDCl_3$]

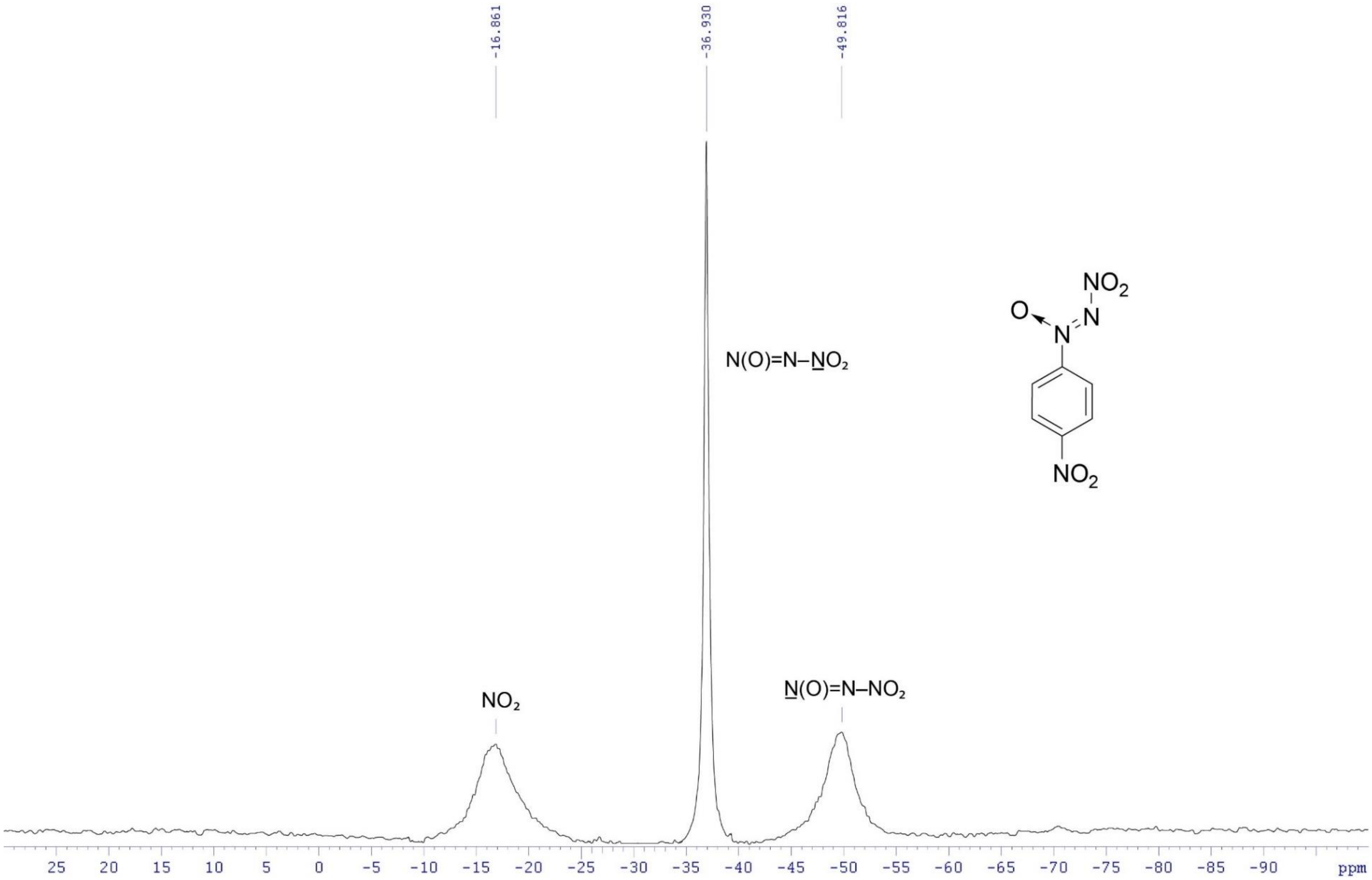

**7.6.1 $^1$H NMR spectrum of compound 2f [600.13 MHz, $CDCl_3$]**

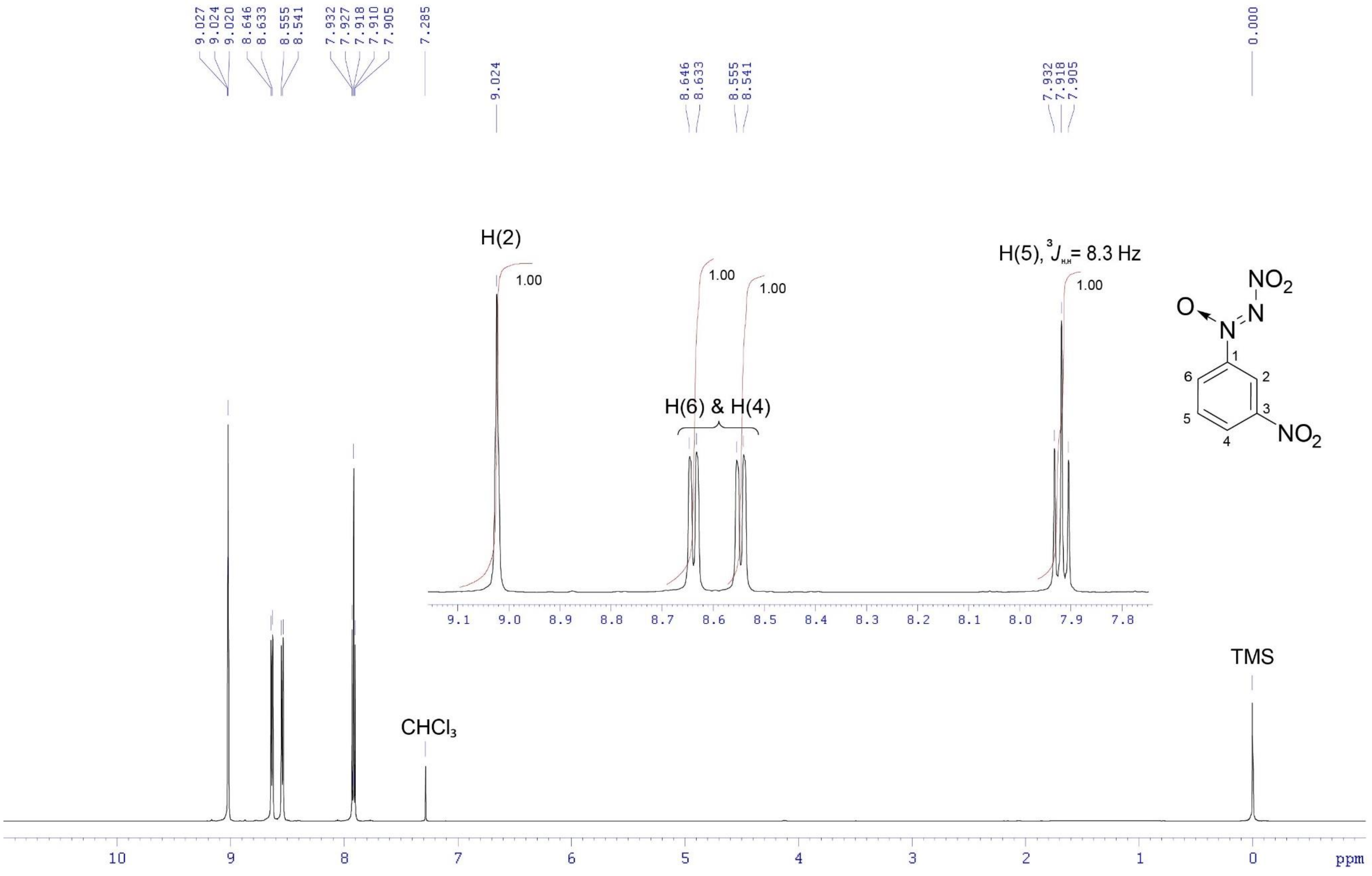

**7.6.2 $^{13}$C NMR spectrum of compound 2f [150.90 MHz, $CDCl_3$]**

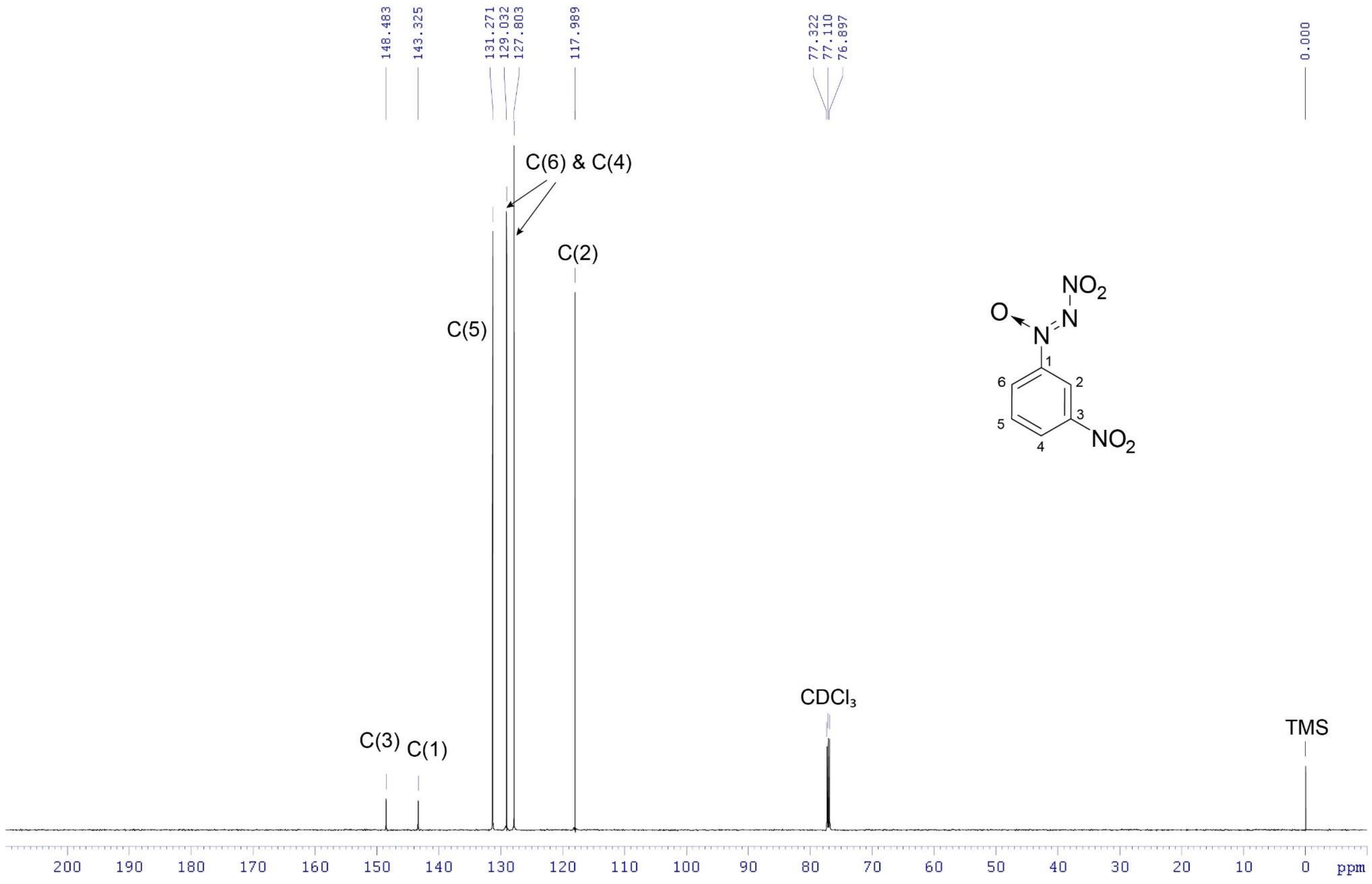

### 7.6.3 {$^{1}H$–$^{13}C$} HSQC spectrum of compound 2f [600.13 MHz, $CDCl_3$]

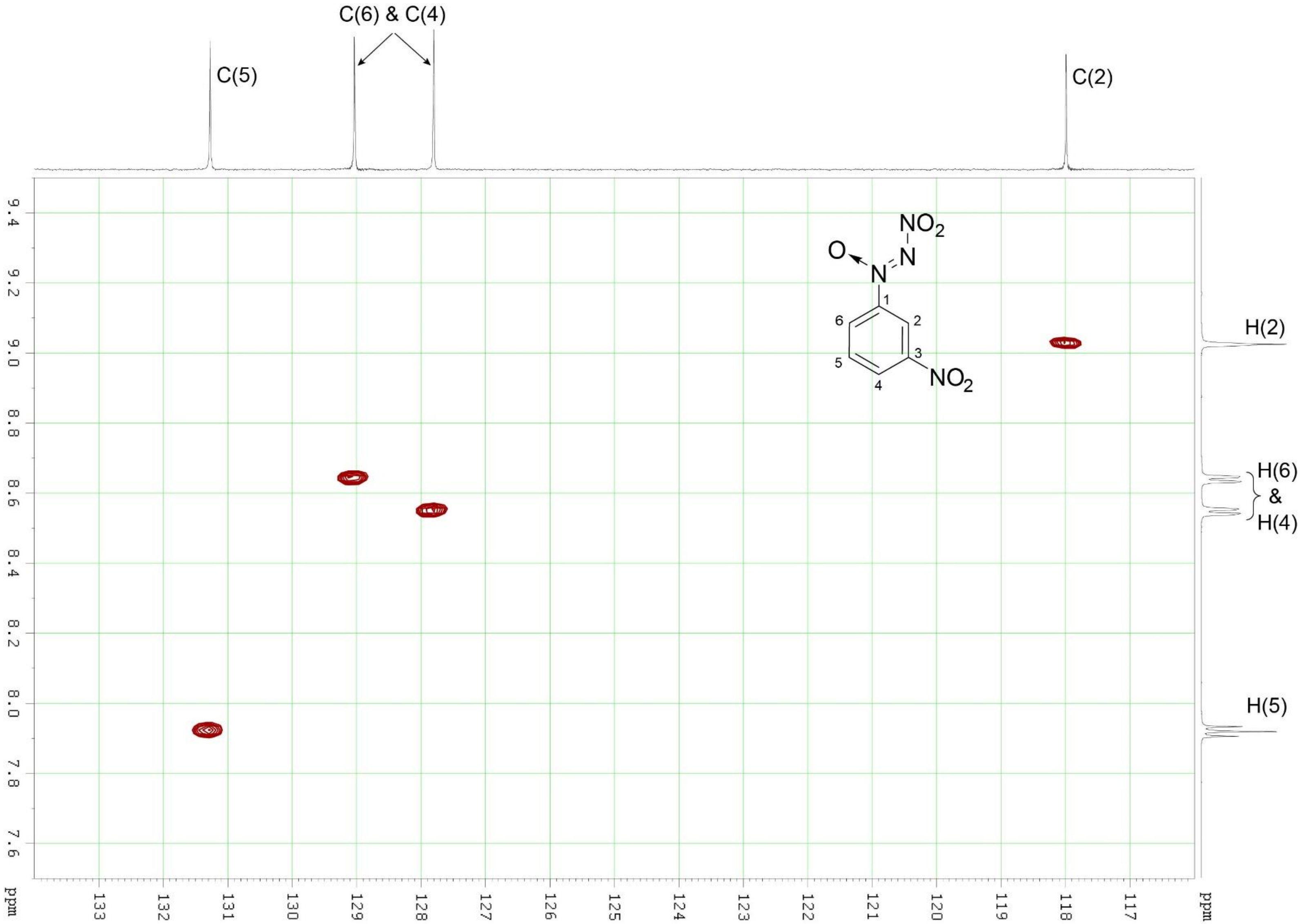

### 7.6.4 {$^1H$–$^{13}C$} HMBC spectrum of compound 2f [600.13 MHz, $CDCl_3$]

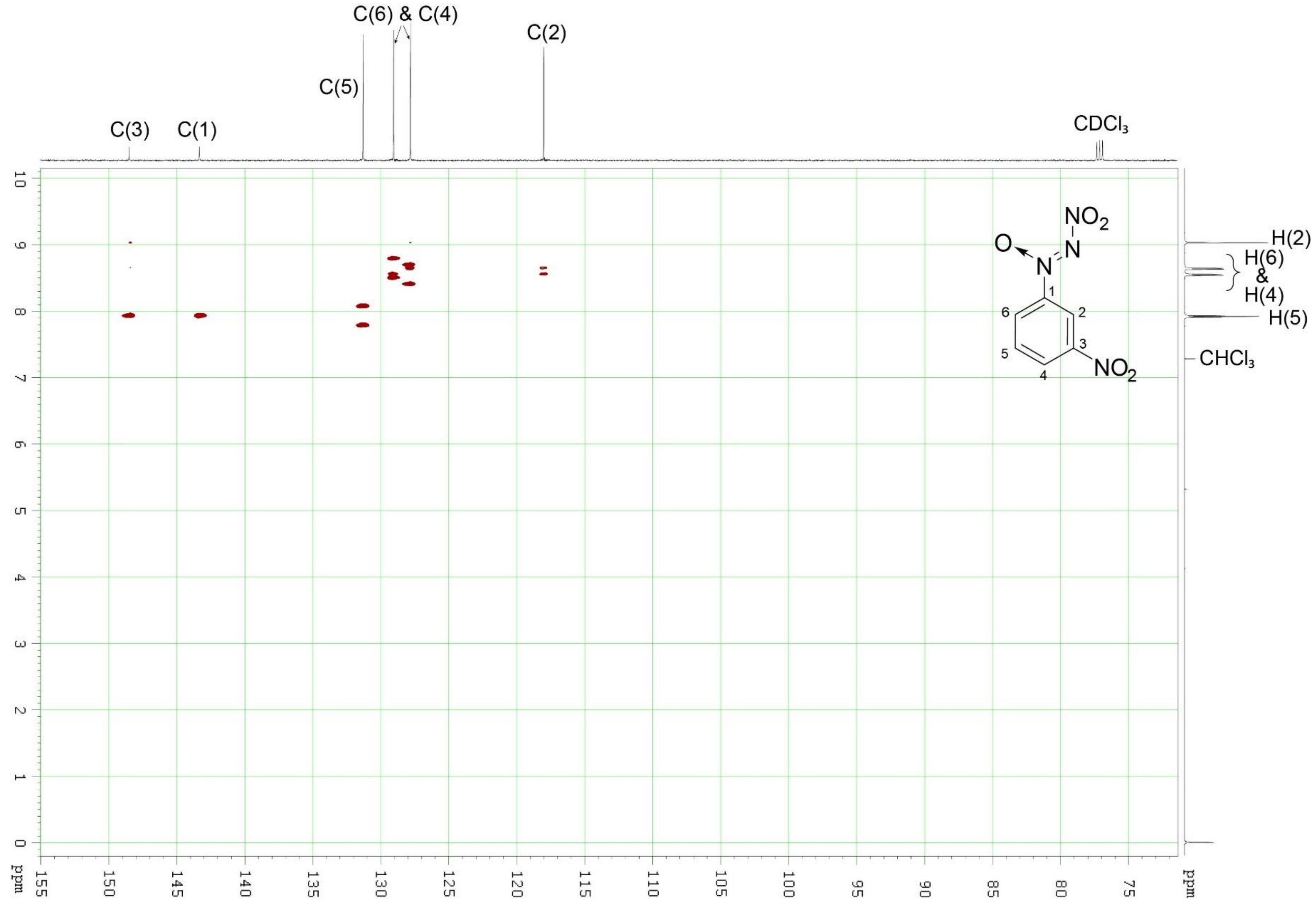

### 7.6.5 $^{14}$N NMR spectrum of compound 2f [43.37 MHz, $CDCl_3$]

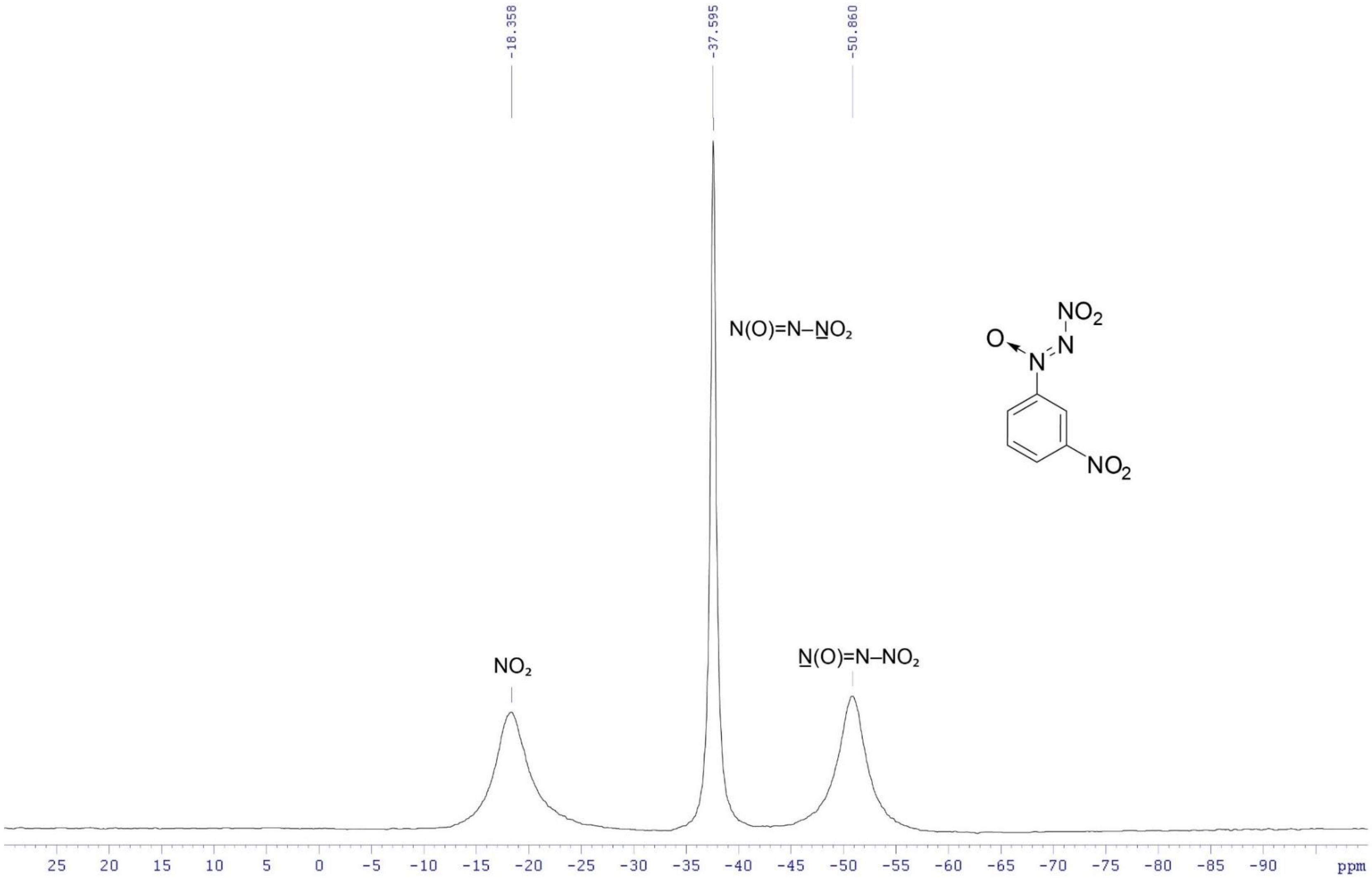

**7.7.1 $^1$H NMR spectrum of compound 2g [600.13 MHz, $CDCl_3$]**

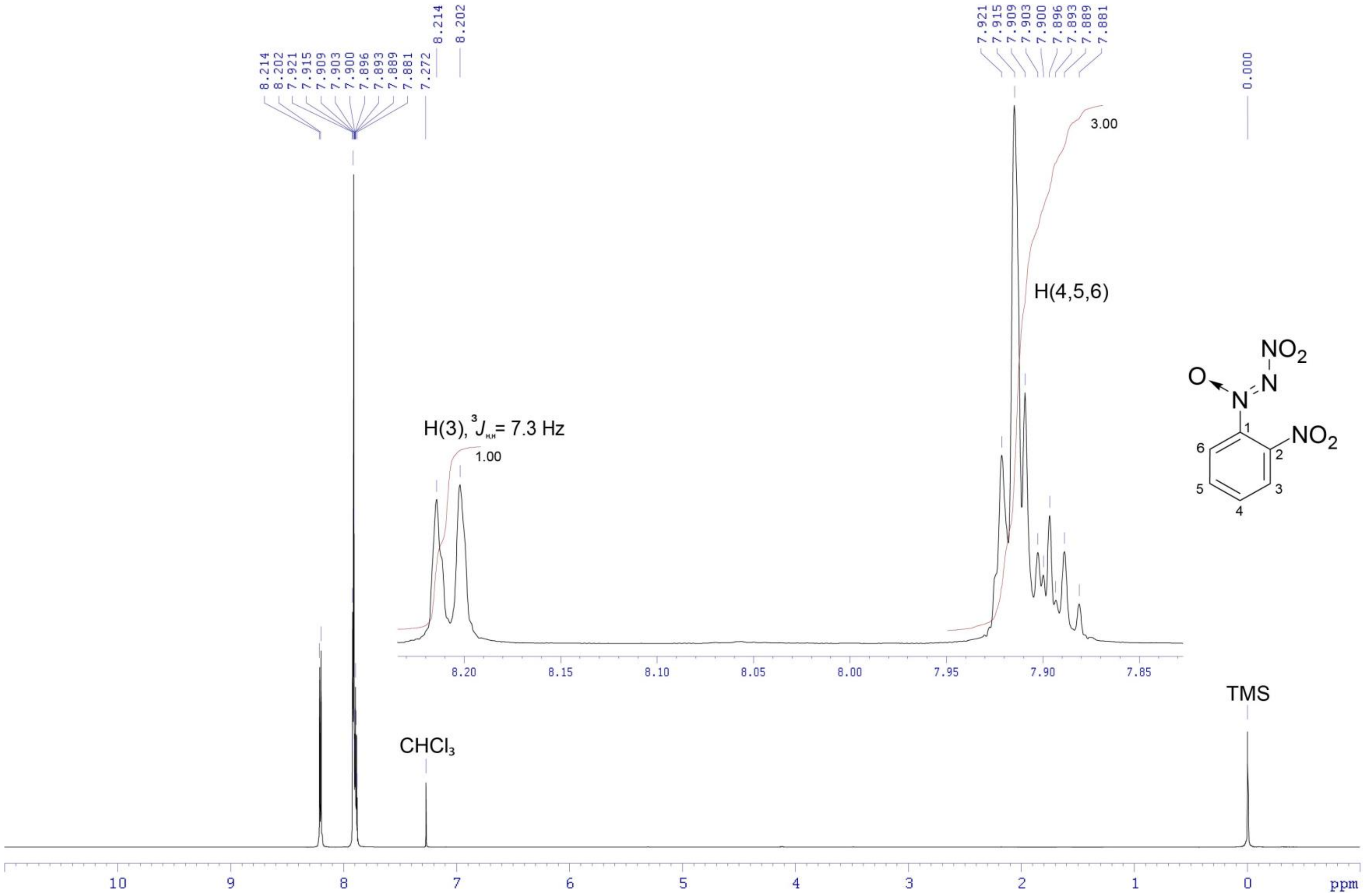

### 7.7.2 $^{13}C$ NMR spectrum of compound 2g [150.90 MHz, $CDCl_3$]

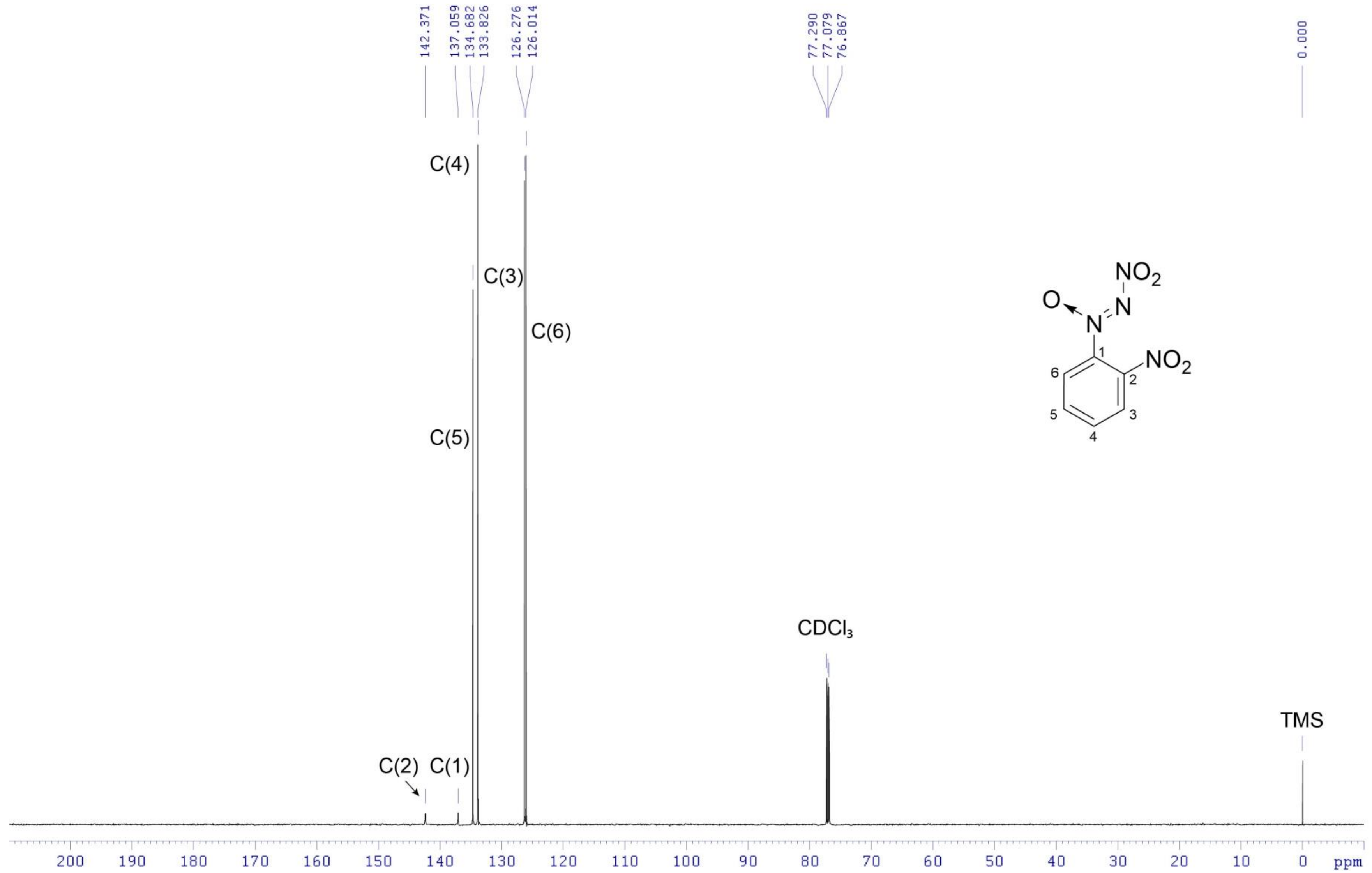

### 7.7.3 {$^{1}$H–$^{13}$C} HSQC spectrum of compound 2g [600.13 MHz, $CDCl_3$]

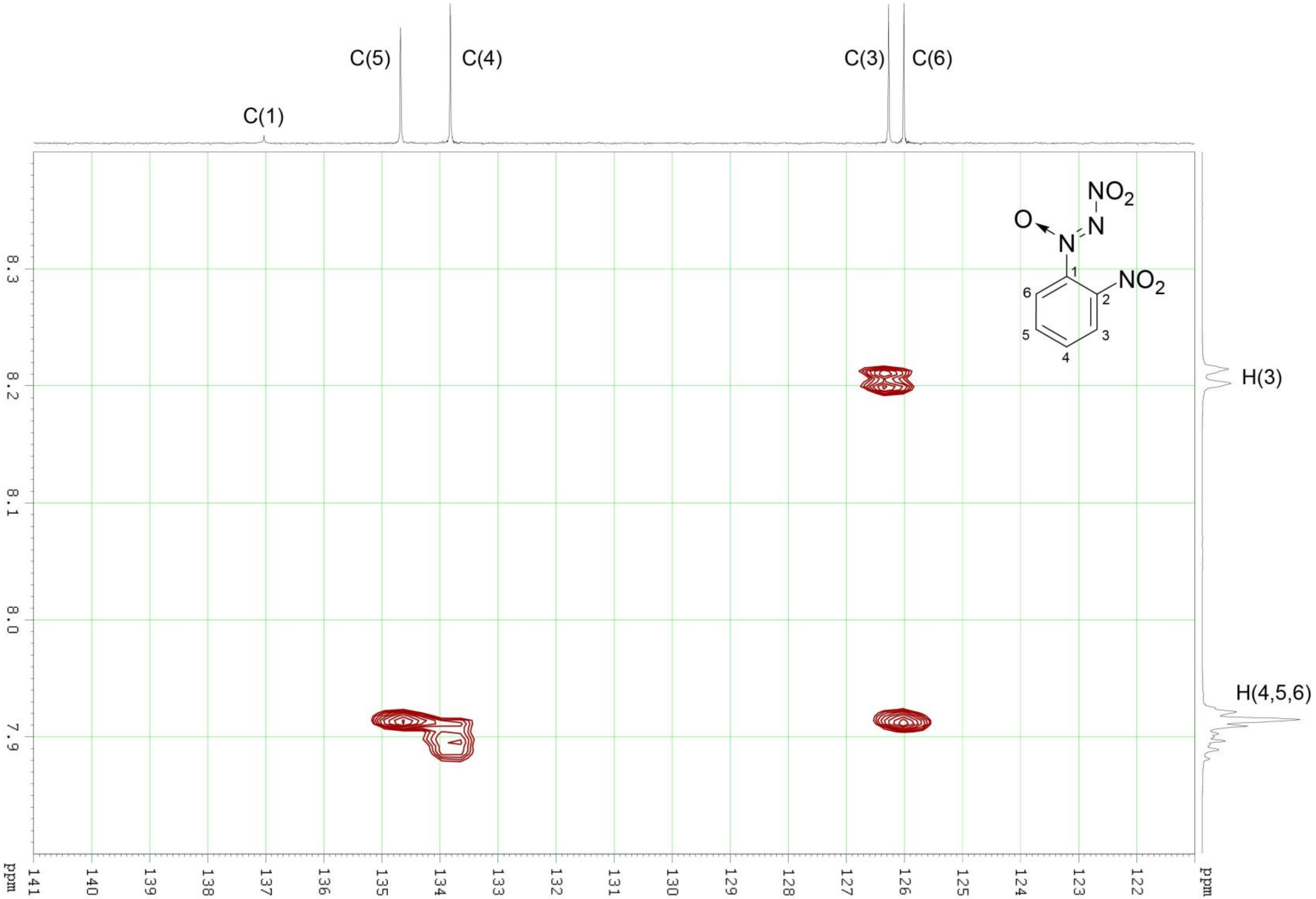

### 7.7.4 {$^{1}$H–$^{13}$C} HMBC spectrum of compound 2g [600.13 MHz, $CDCl_3$]

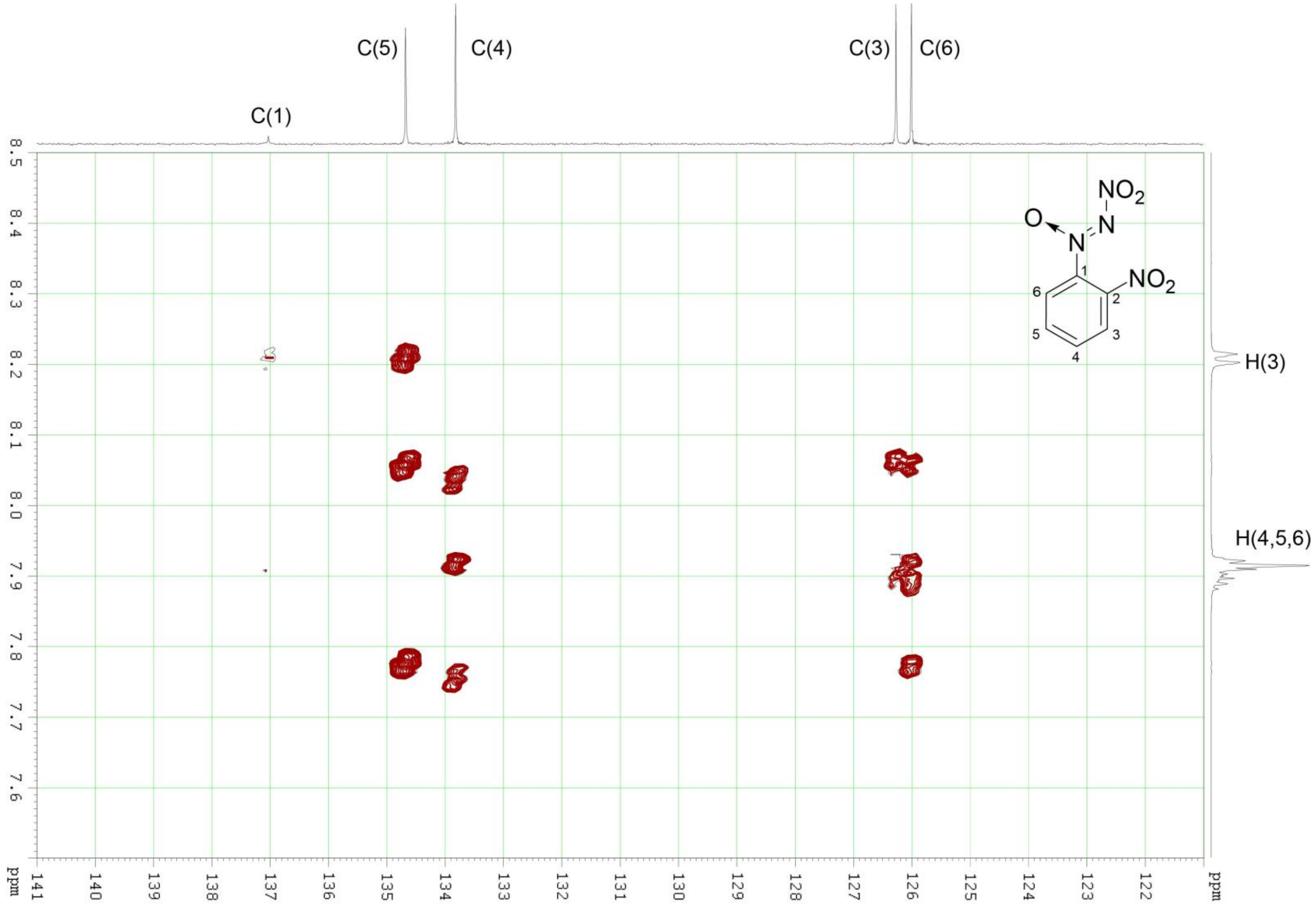

**7.7.5 $^{14}N$ NMR spectrum of compound 2g [43.14 MHz, $CDCl_3$]**

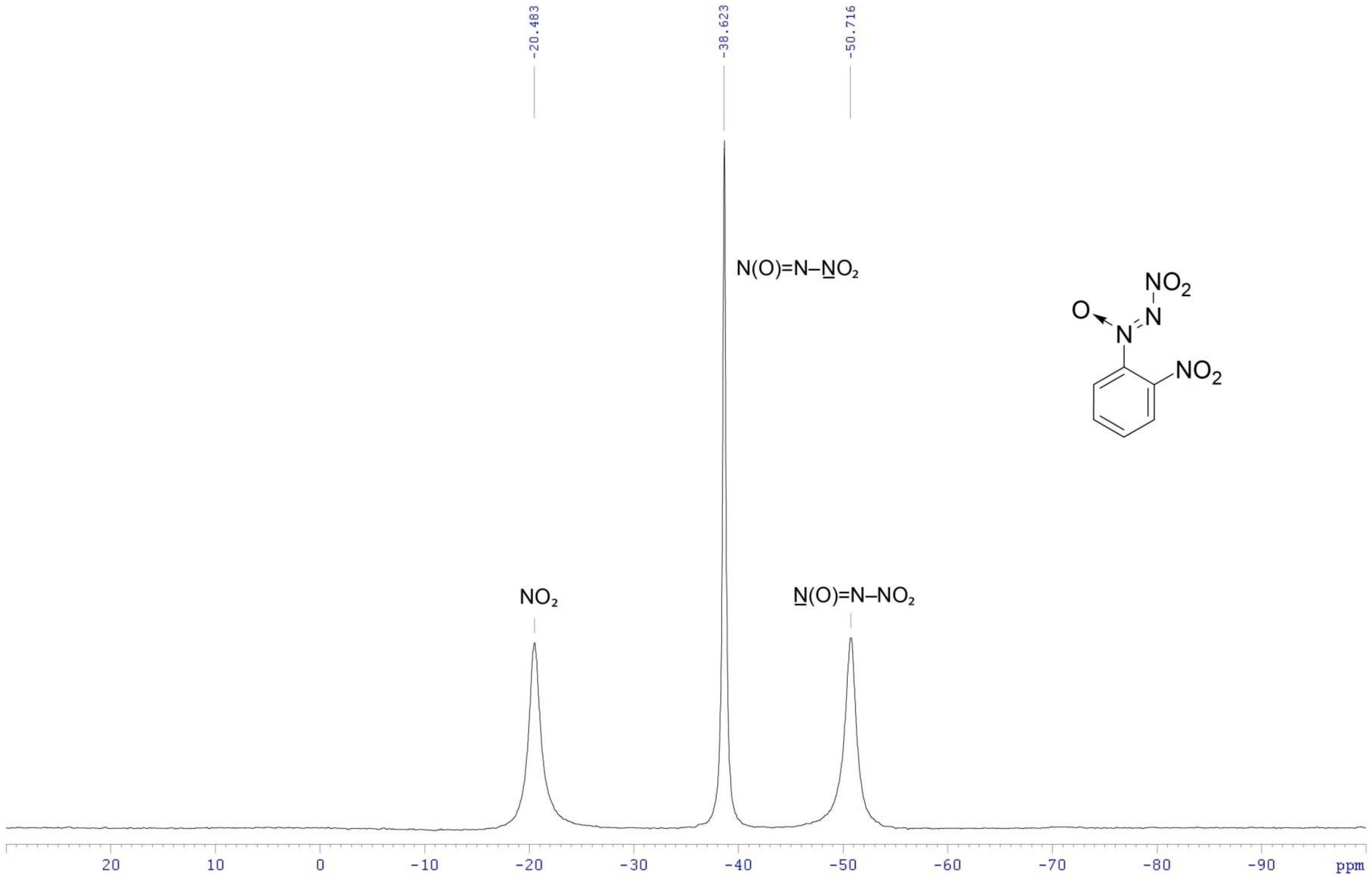

7.8.1 $^{1}$H NMR spectrum of compound 2i [600.13 MHz, $CDCl_3$]

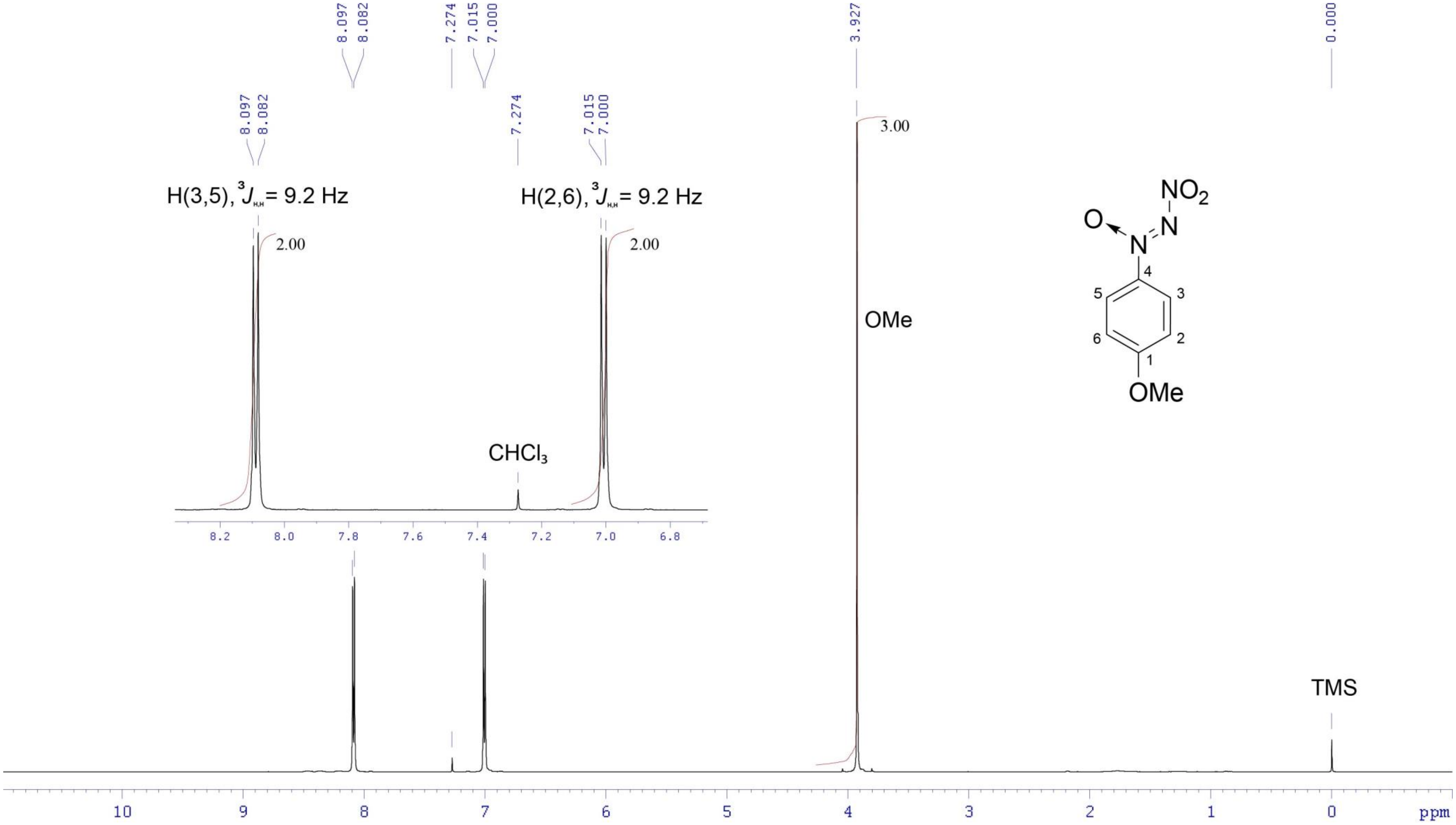

### 7.8.2 $^{13}$C NMR spectrum of compound 2i [150.90 MHz, $CDCl_3$]

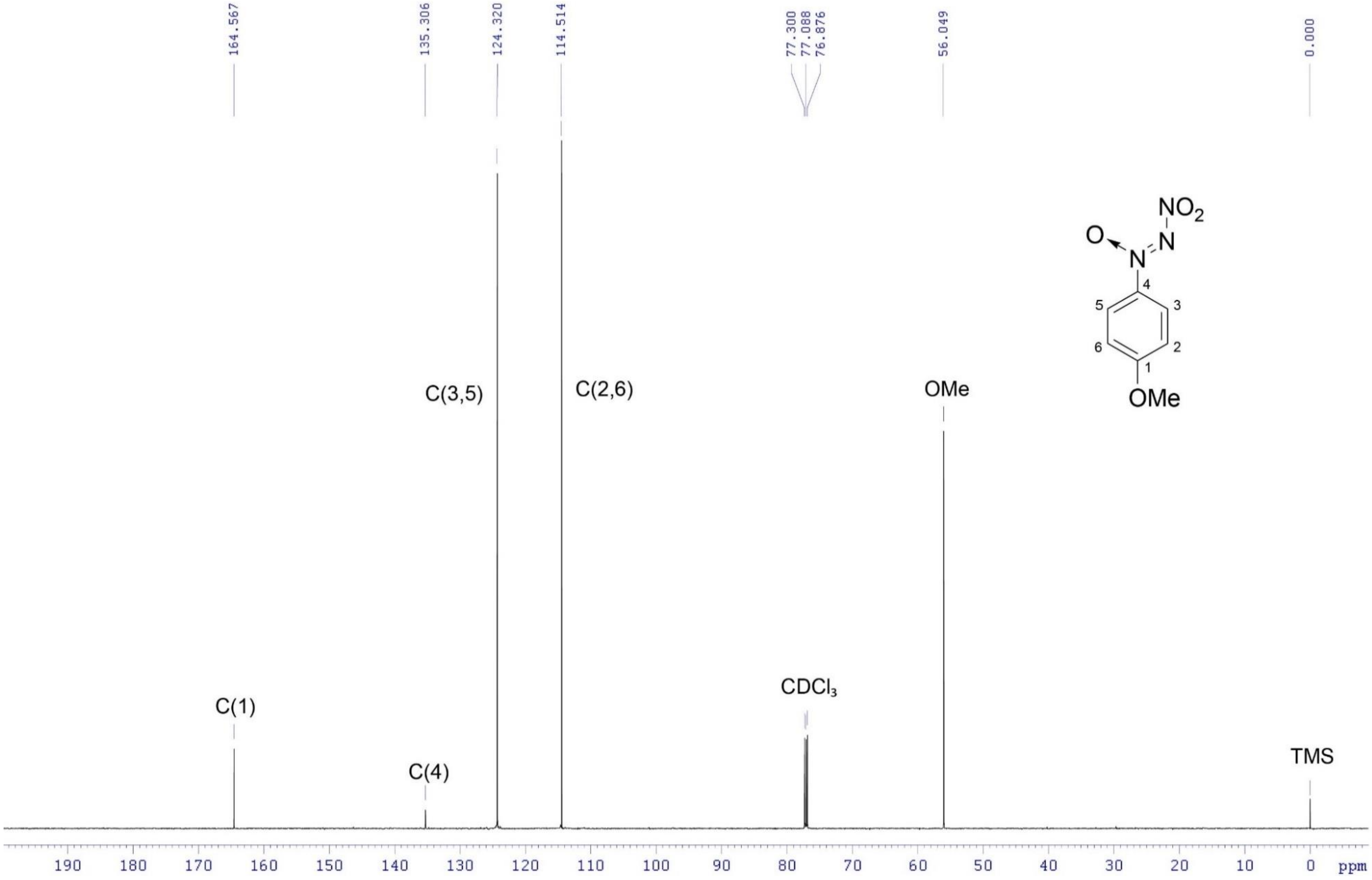

### 7.8.3 {$^1H$–$^{13}C$} HSQC spectrum of compound 2i [600.13 MHz, $CDCl_3$]

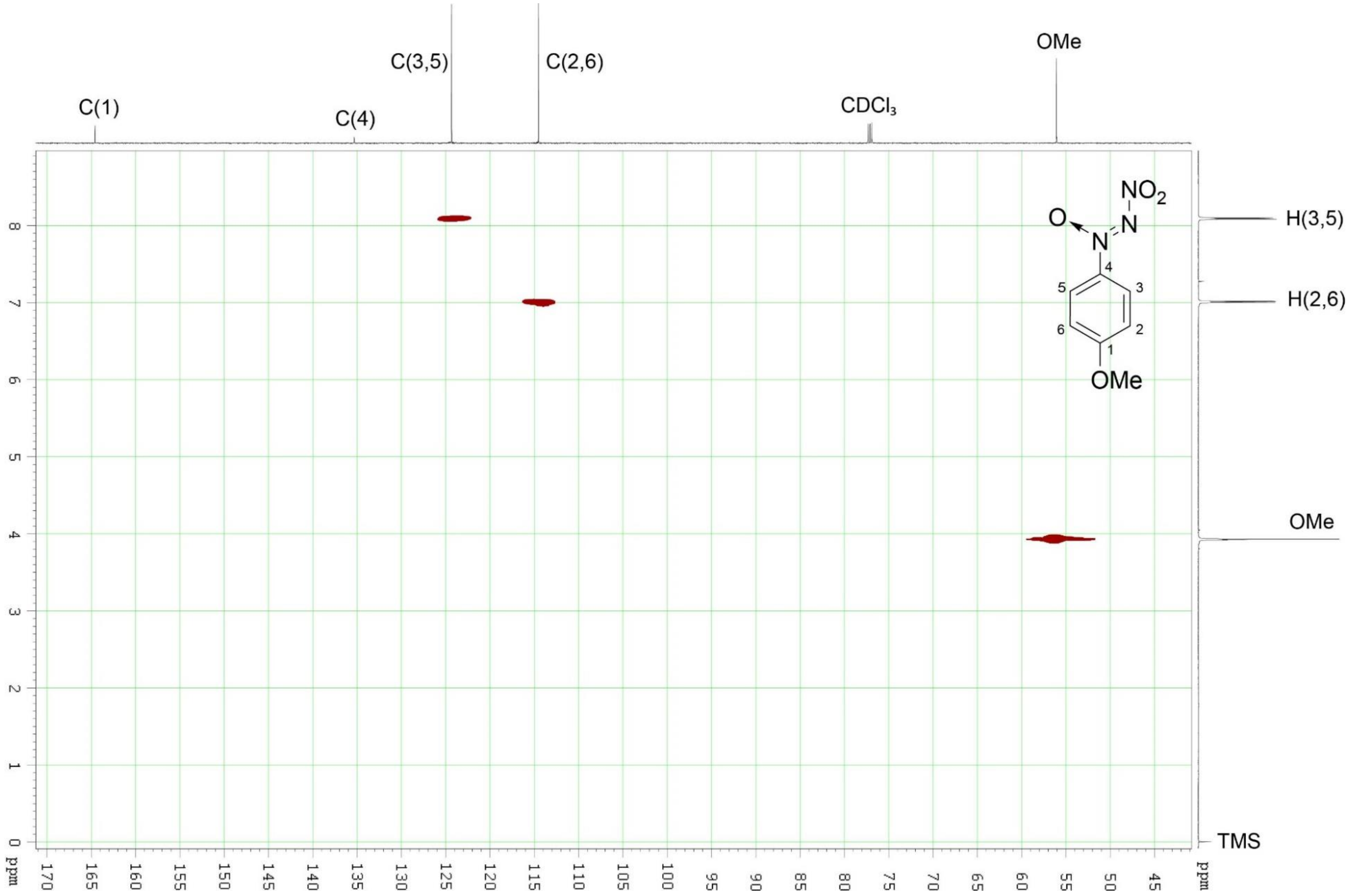

#### 7.8.4 {$^1H$–$^{13}C$} HMBC spectrum of compound 2i [600.13 MHz, $CDCl_3$]

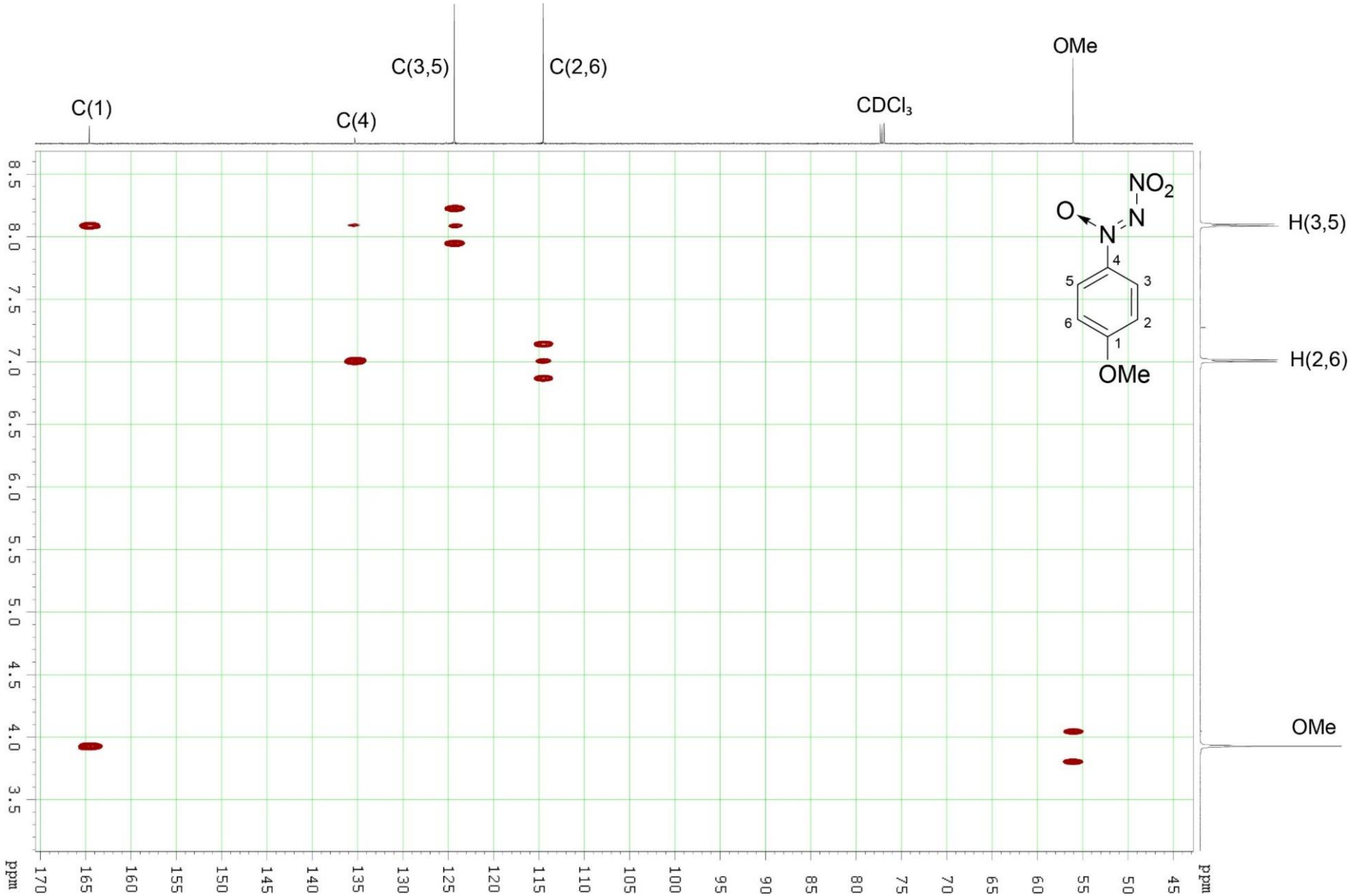

**7.8.5 $^{14}$N NMR spectrum of compound 2i [43.37 MHz, $CDCl_3$]**

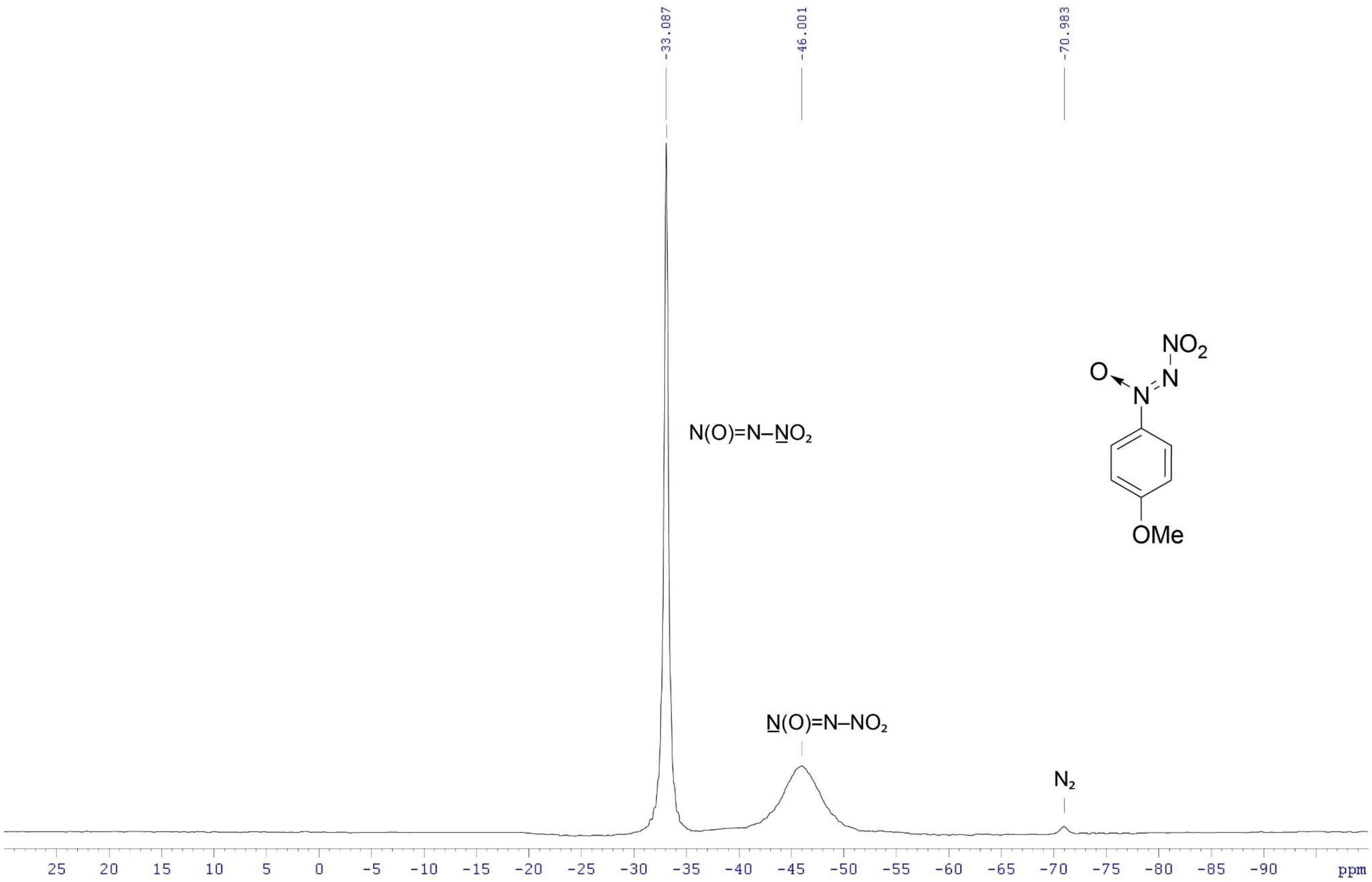

7.9.1 $^{1}$H NMR spectrum of compound 2j [600.13 MHz, $CDCl_3$]

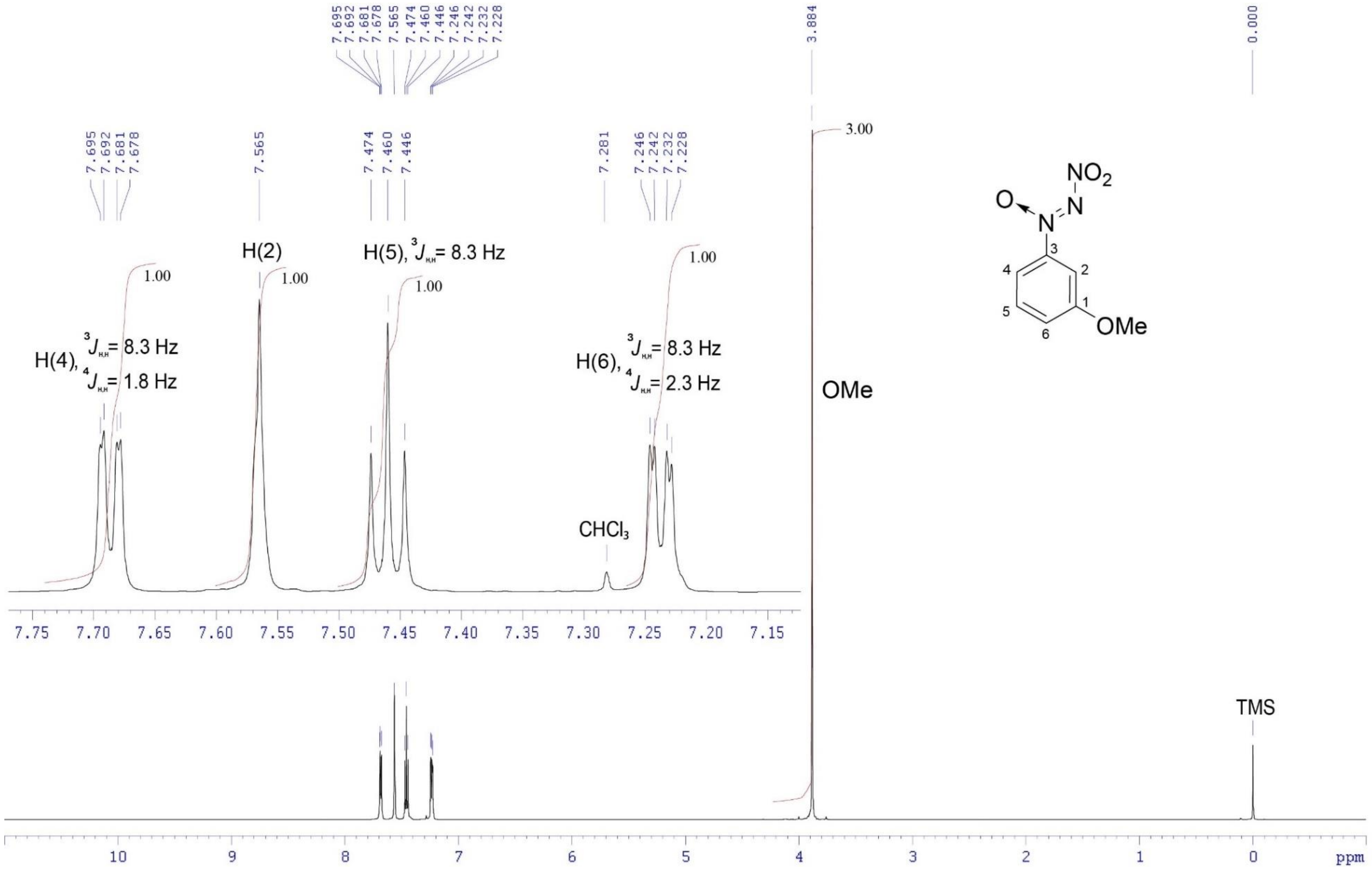

### 7.9.2 $^{13}$C NMR spectrum of compound 2j [150.90 MHz, $CDCl_3$]

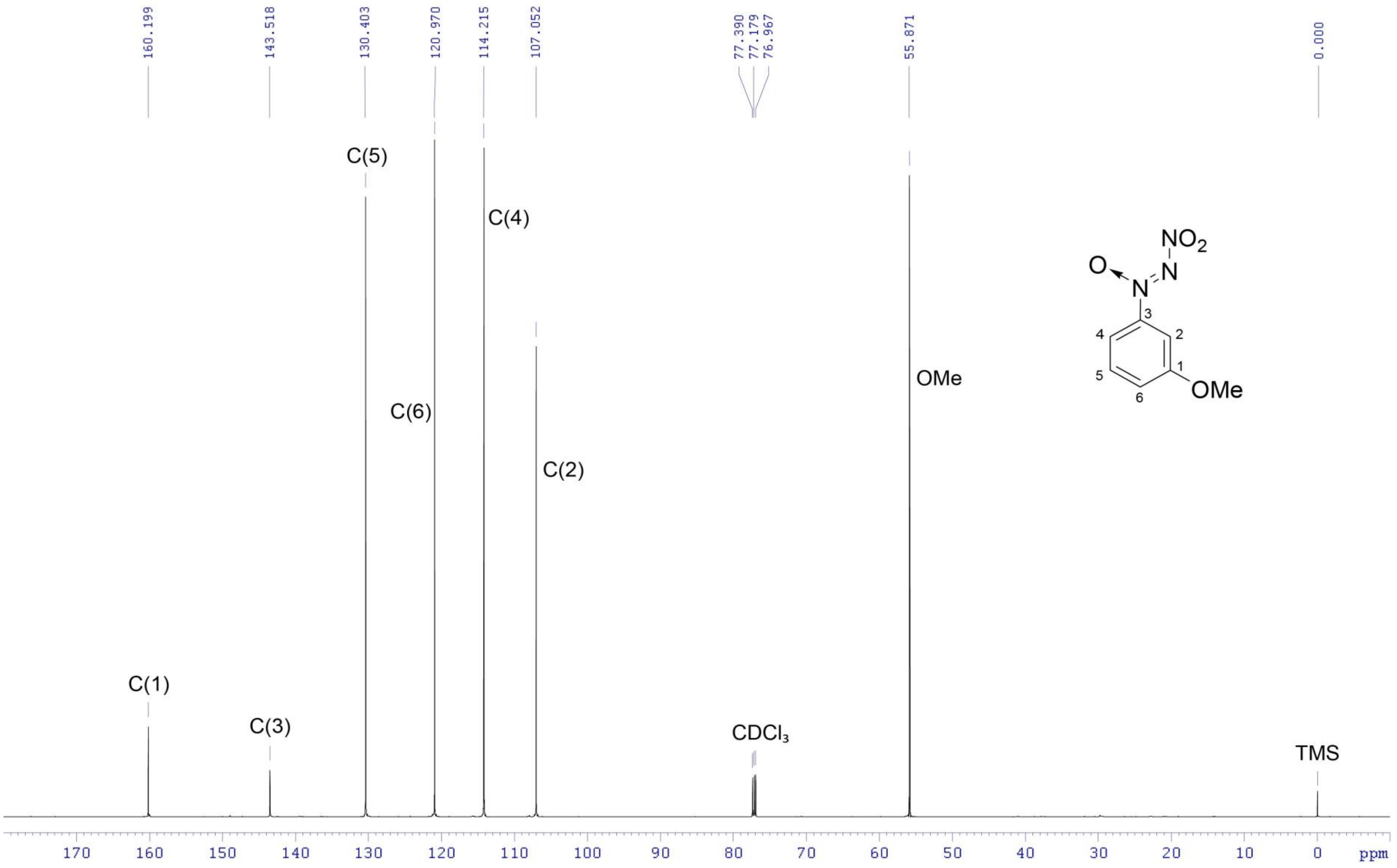

### 7.9.3 {$^1H$–$^{13}C$} HSQC spectrum of compound 2j [600.13 MHz, $CDCl_3$]

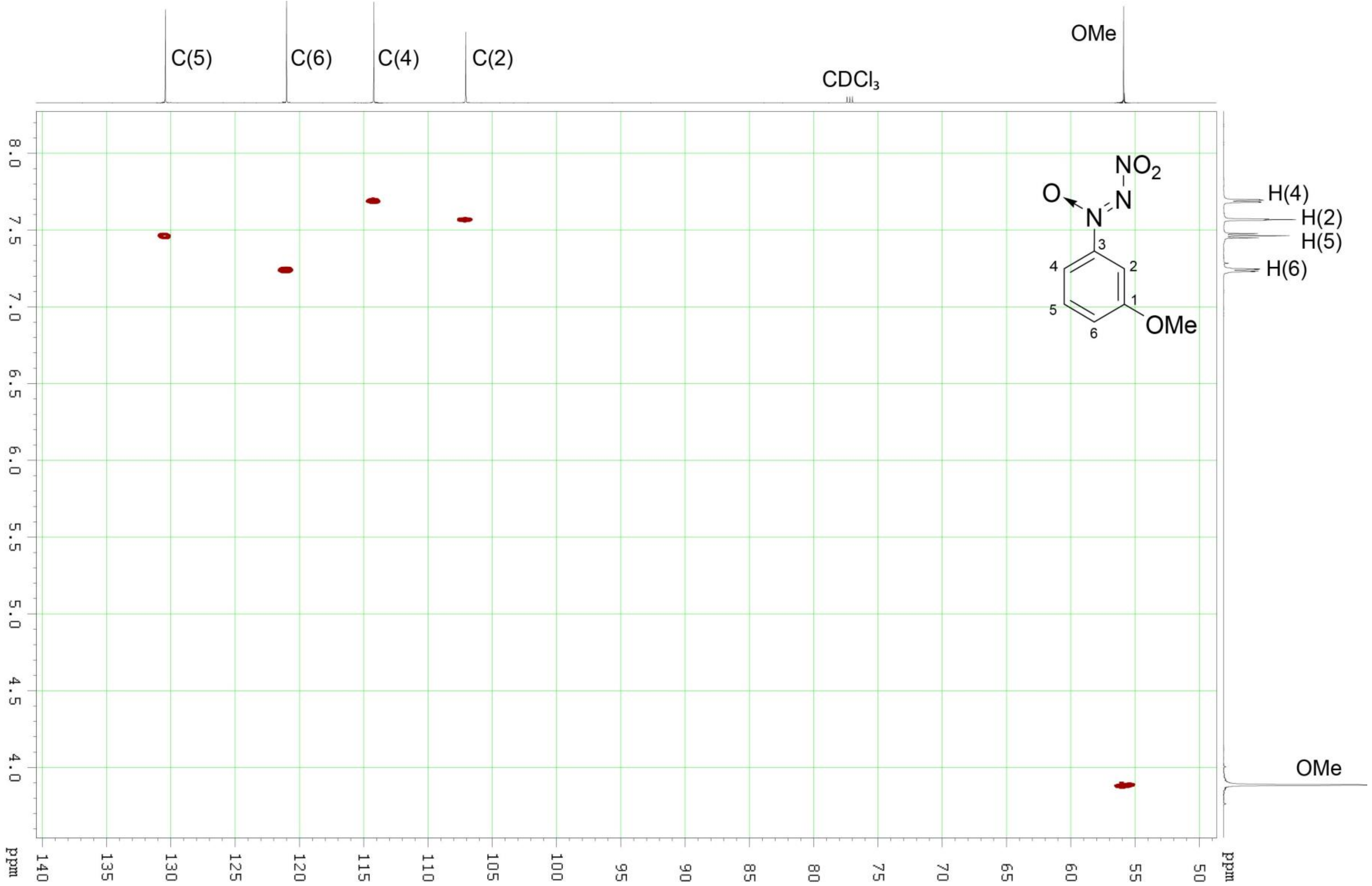

### 7.9.4 {$^1$H–$^{13}$C} HMBC spectrum of compound 2j [600.13 MHz, $CDCl_3$]

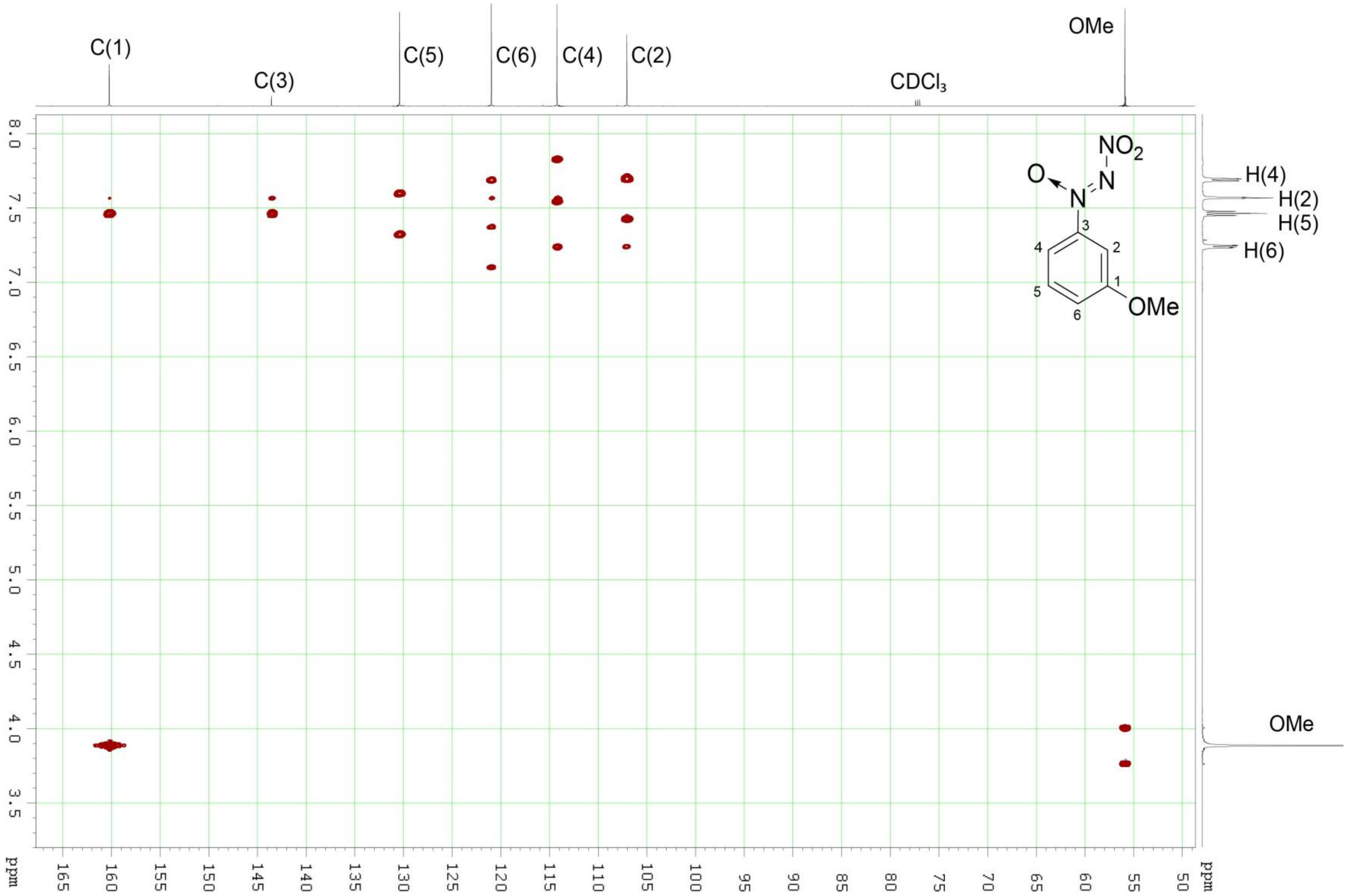

7.9.5 $^{14}$N NMR spectrum of compound 2j [43.37 MHz, $CDCl_3$]

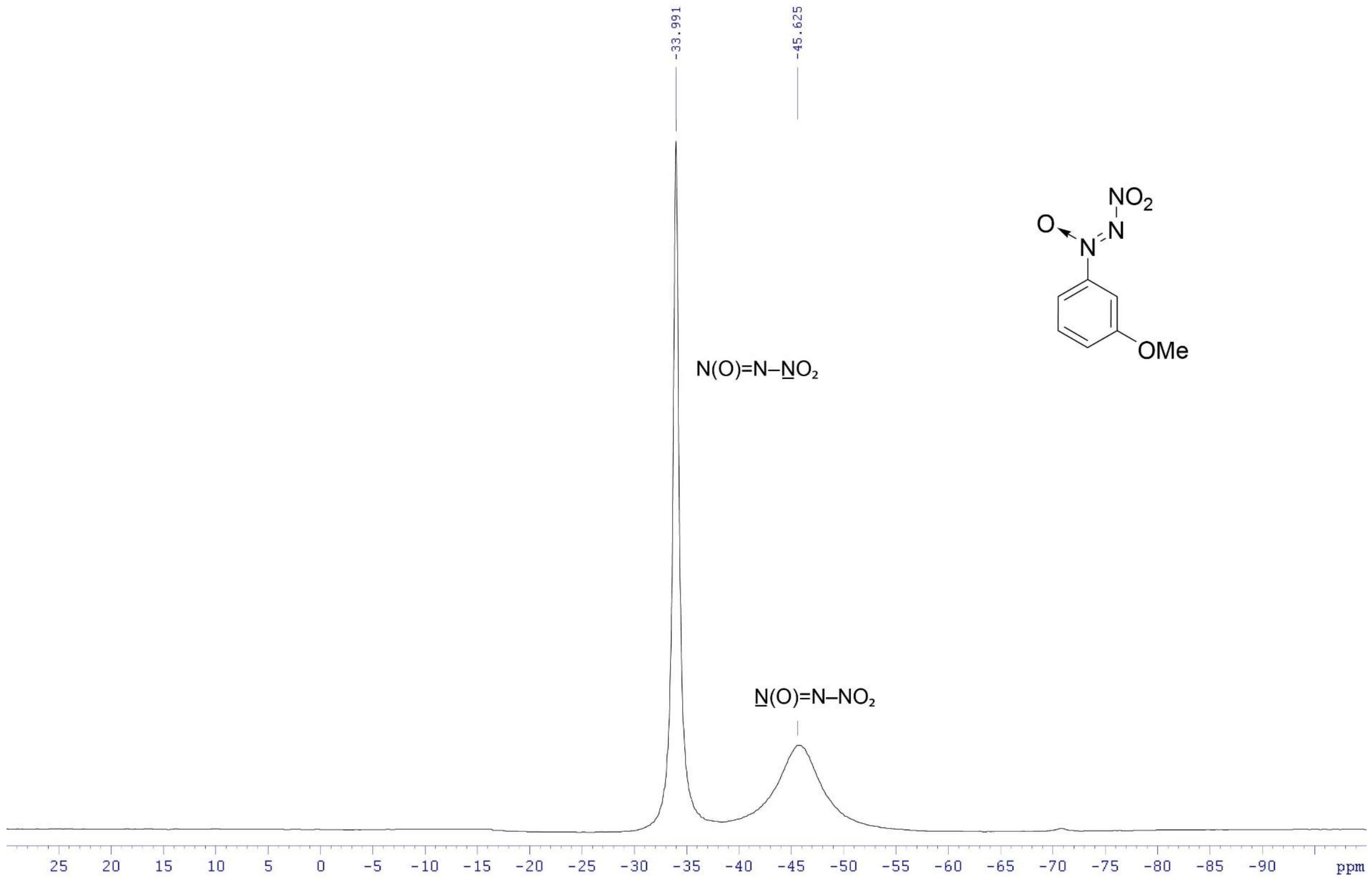

**7.10.1 $^1$H NMR spectrum of compound 2k [300.13 MHz, $CDCl_3$]**

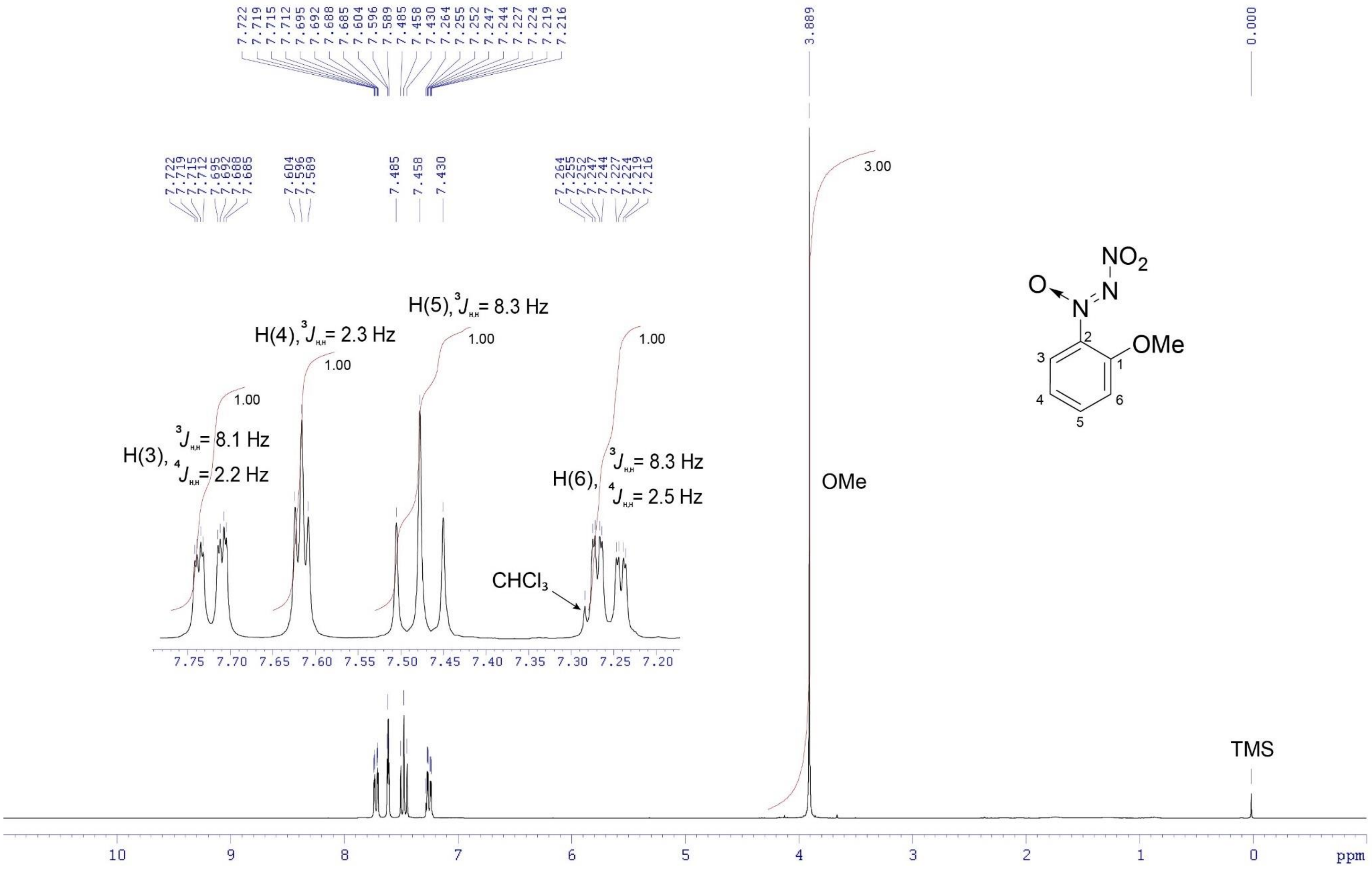

**7.10.2 $^{13}C$ NMR spectrum of compound 2k [300.13 MHz, $CDCl_3$]**

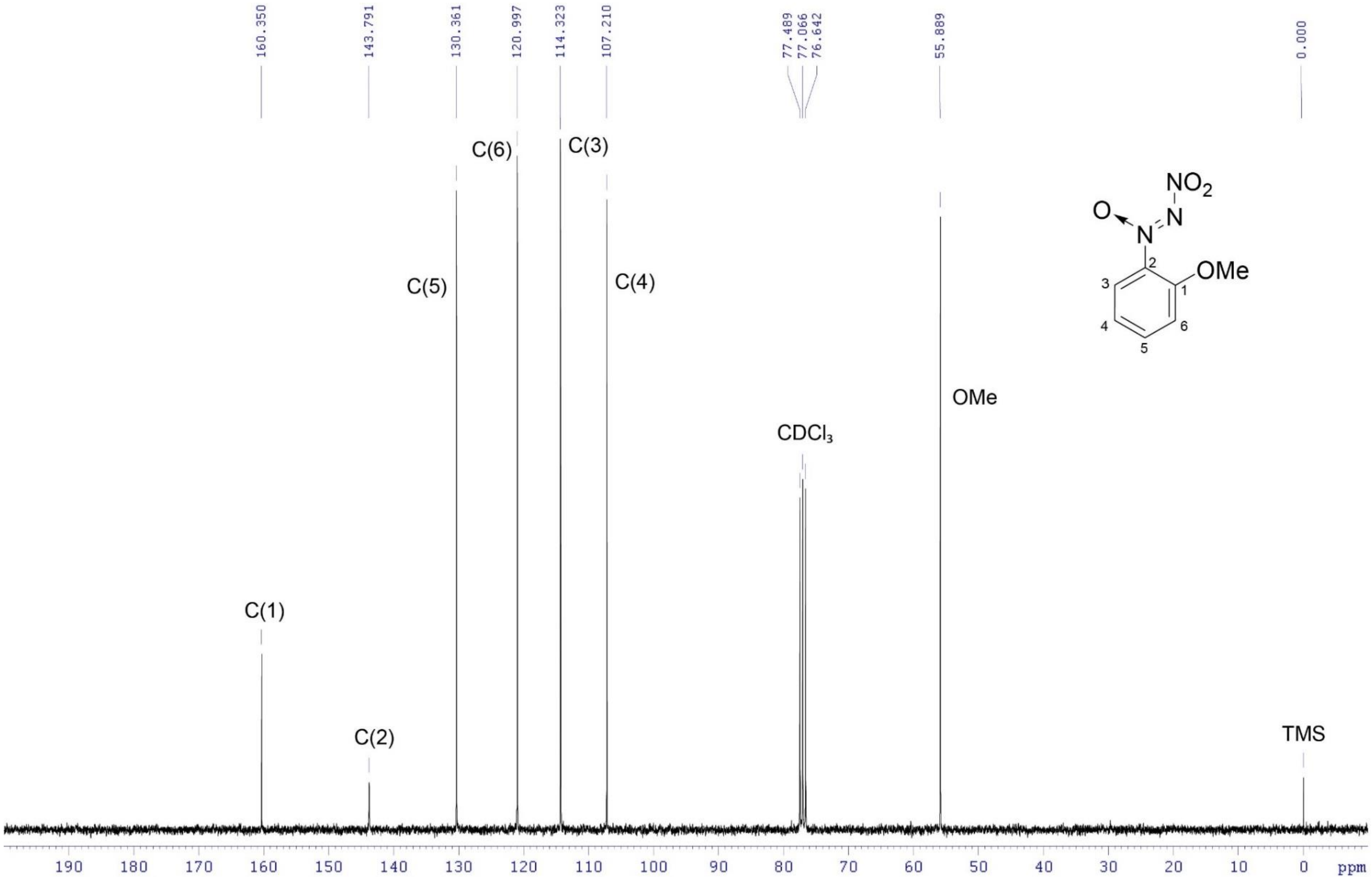

### 7.10.3 {$^1H$–$^{13}C$} HSQC spectrum of compound 2k [300.13 MHz, $CDCl_3$]

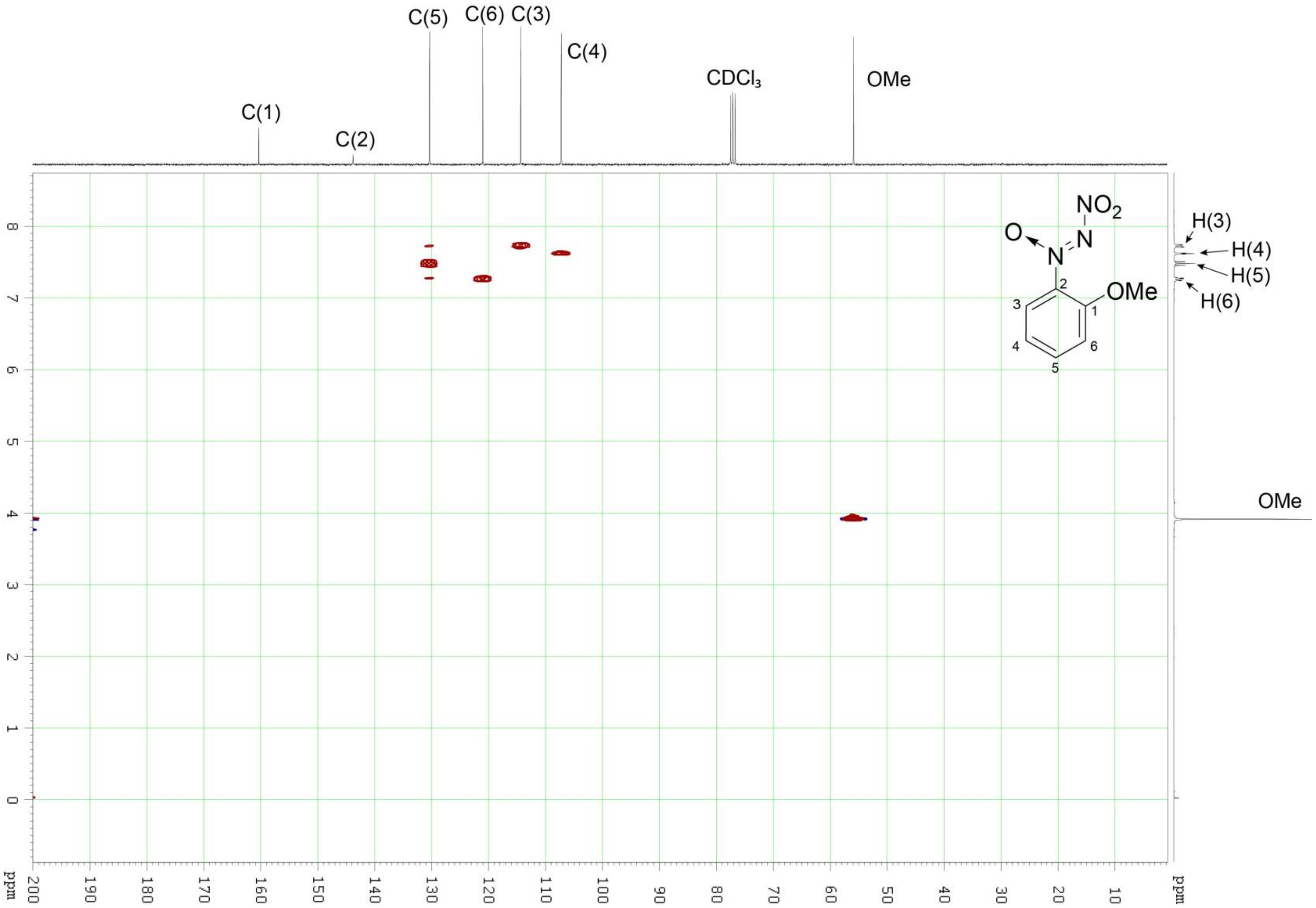

**7.10.4 {$^1H$–$^{13}C$} HMBC spectrum of compound 2k [300.13 MHz, $CDCl_3$]**

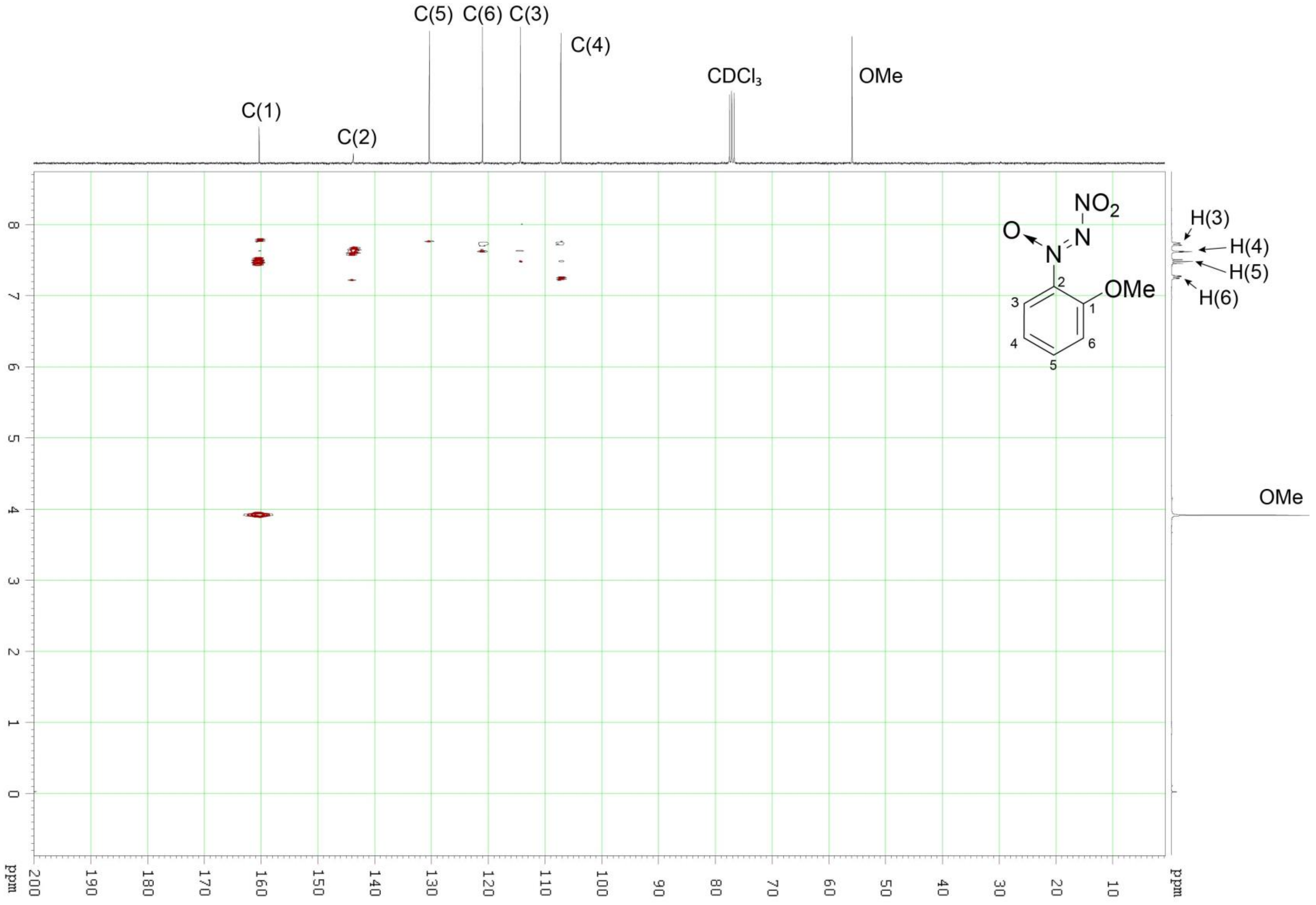

**7.10.5 $^{14}N$ NMR spectrum of compound 2k [43.37 MHz, $CDCl_3$]**

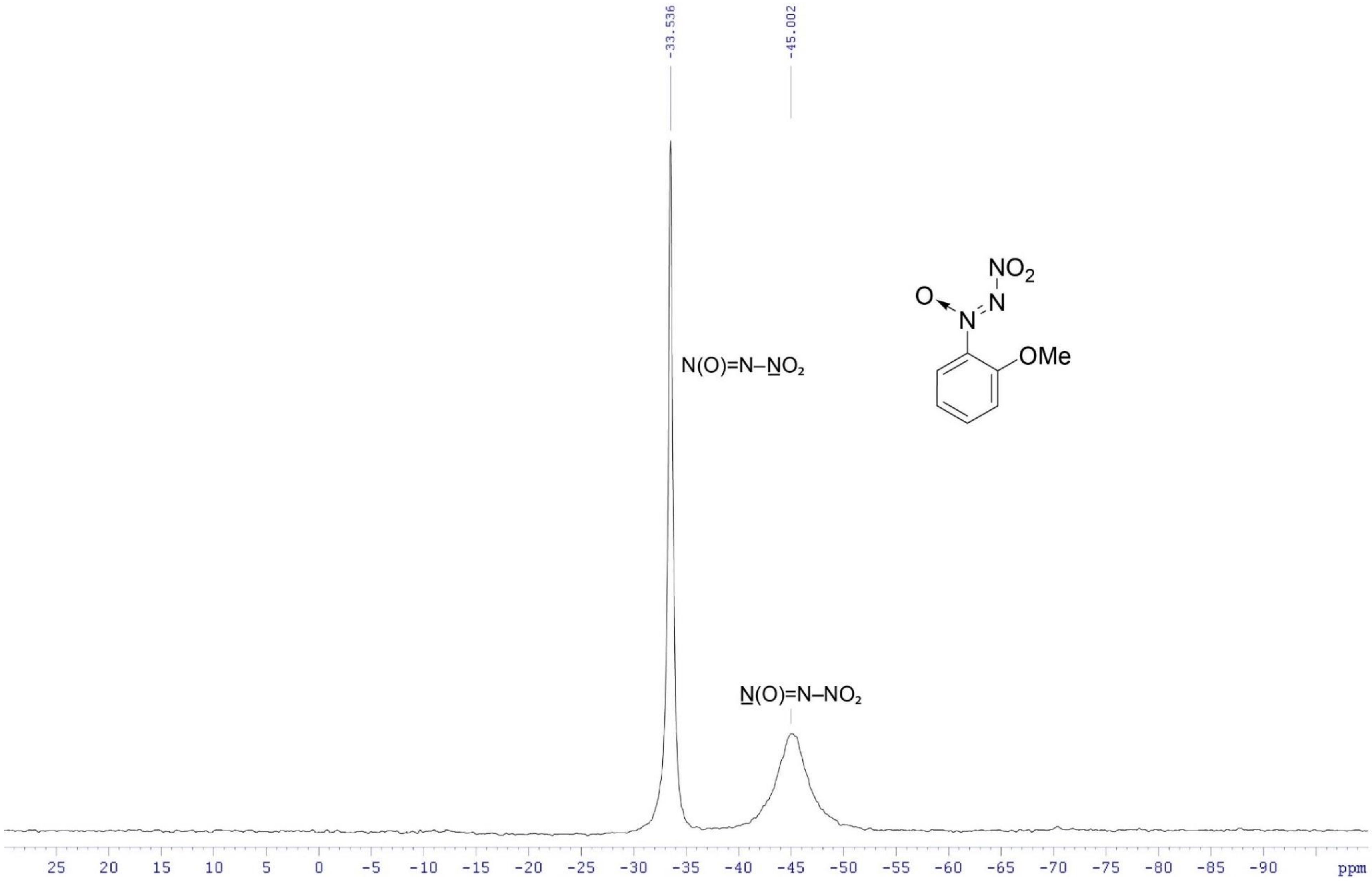

### 7.11.1 $^1$H NMR spectrum of the mixture of compound 2l and 4-fluoro-1-nitrobenzene (1.5 : 1 molar ratio) [600.13 MHz, $CDCl_3$]

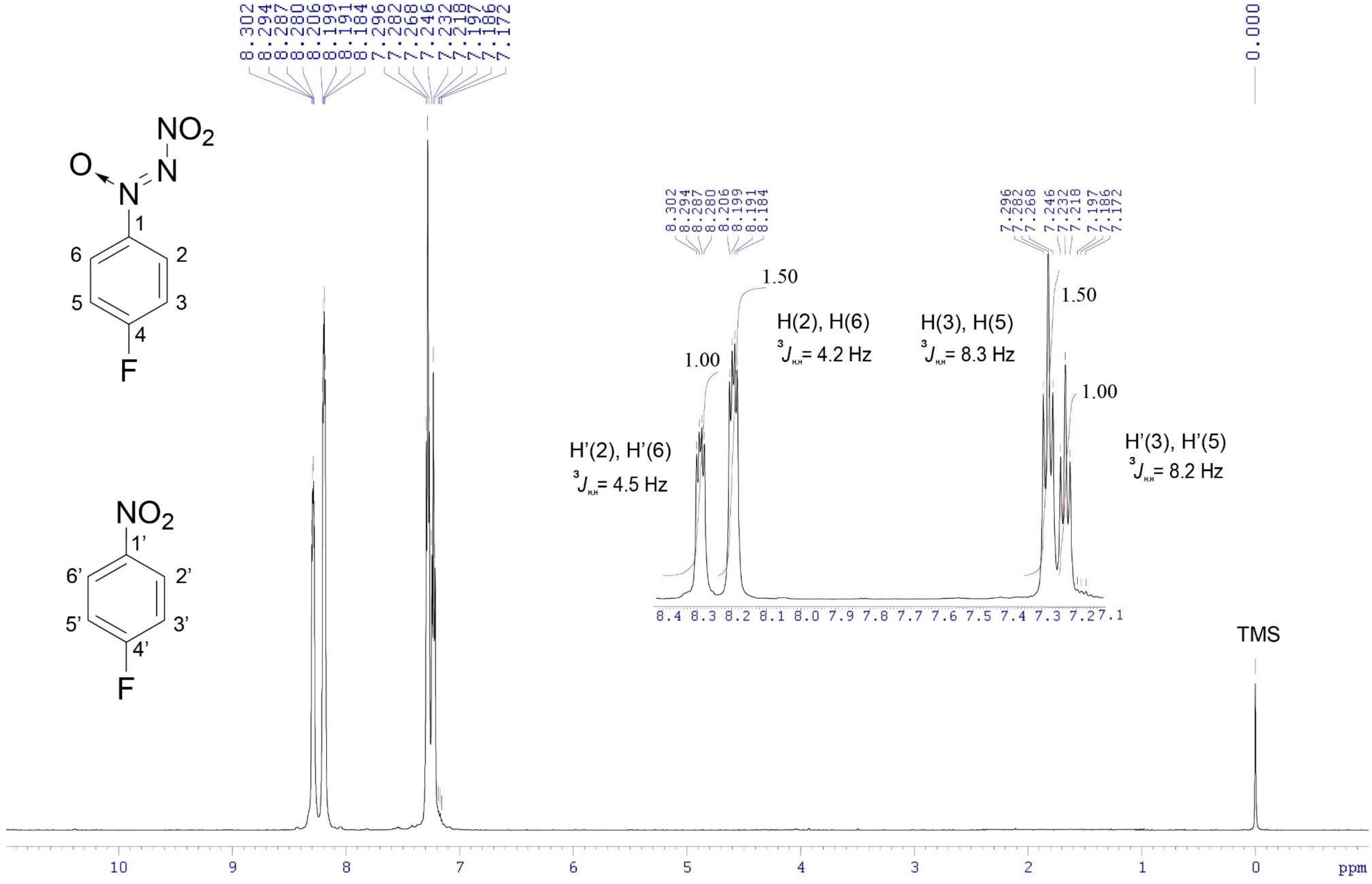

### 7.11.2 $^{13}$C NMR spectrum of the mixture of compound 2l and 4-fluoro-1-nitrobenzene (1.5 : 1 molar ratio) [150.90 MHz, $CDCl_3$]

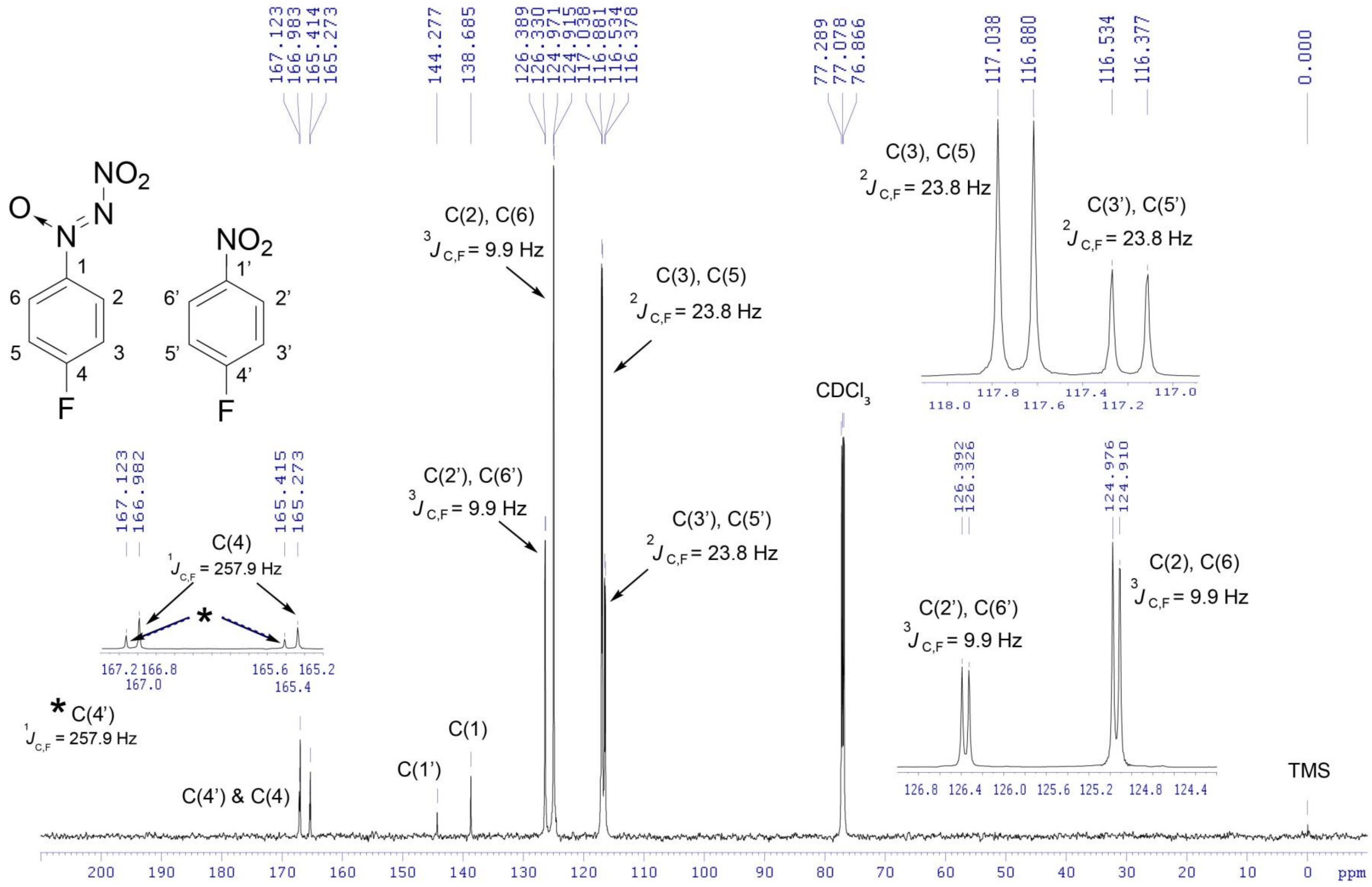

### 7.11.3 {$^1H$–$^{13}C$} HSQC spectrum of the mixture of compound 2l and 4-fluoro-1-nitrobenzene [600.13 MHz, $CDCl_3$]

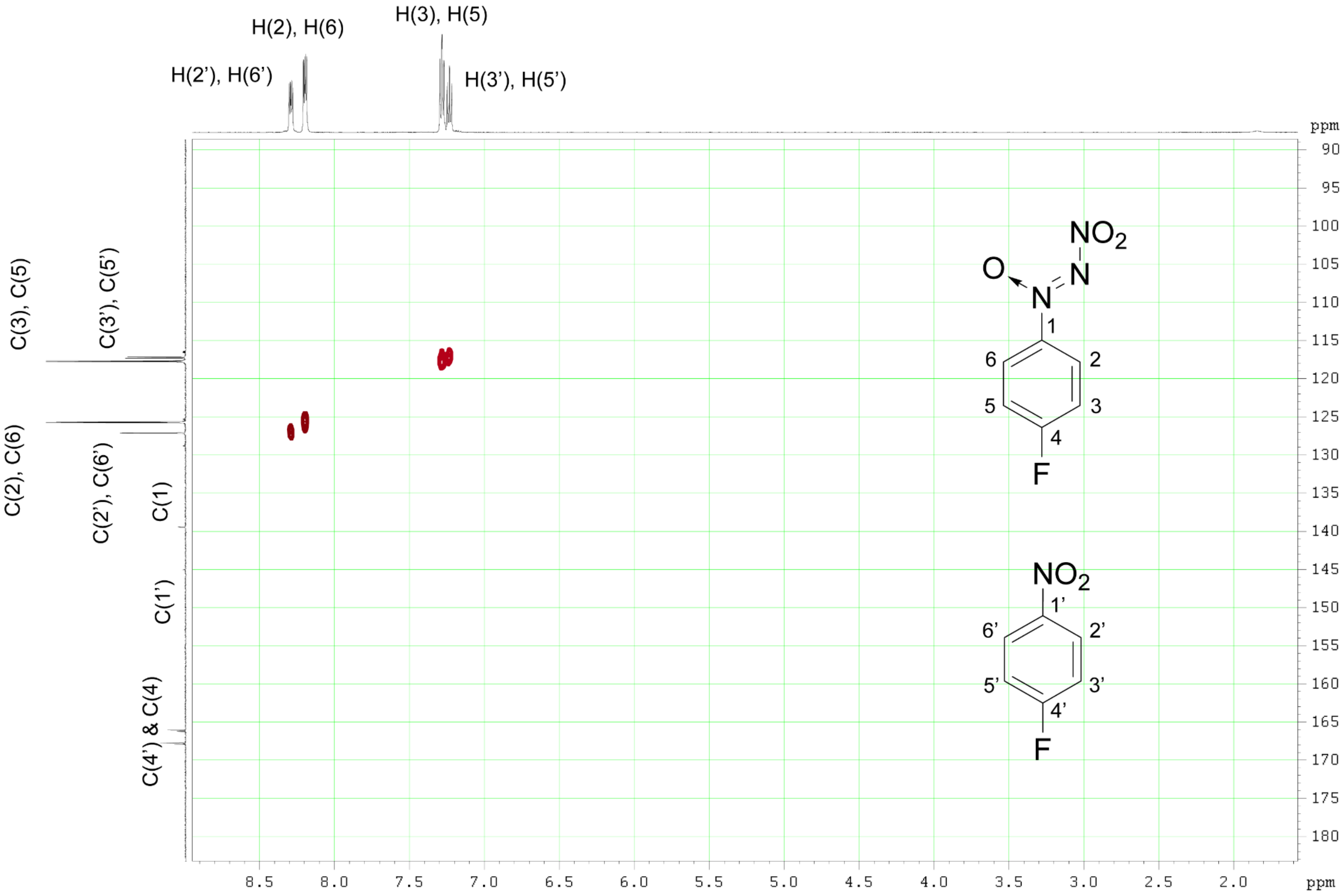

**7.11.4 {$^1$H–$^{13}$C} HMBC spectrum of the mixture of compound 2l and 4-fluoro-1-nitrobenzene [600.13 MHz, $CDCl_3$]**

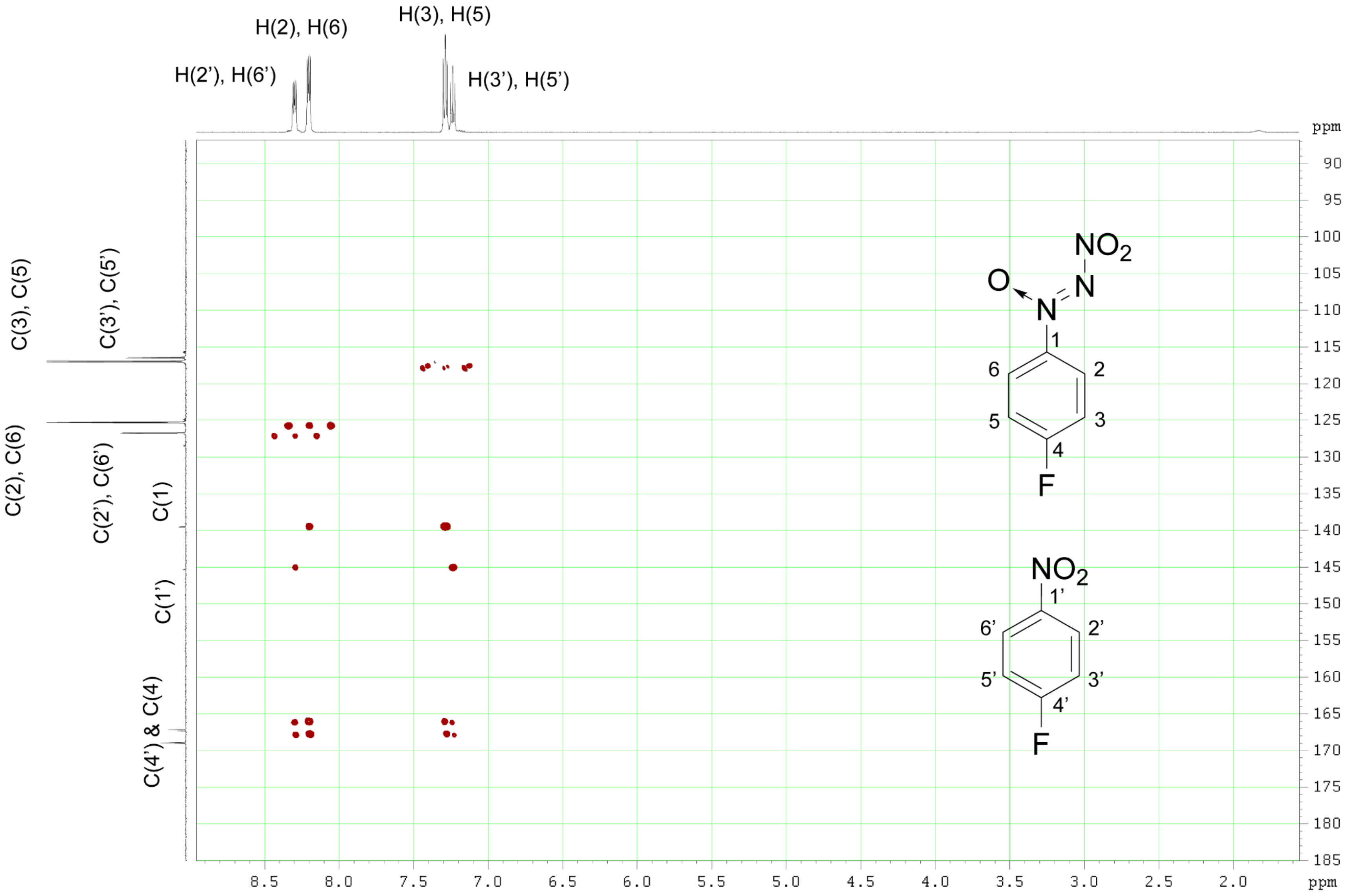

### 7.11.5 $^{14}N$ NMR spectrum of the mixture of compound 2l and 4-fluoro-1-nitrobenzene [43.37 MHz, $CDCl_3$]

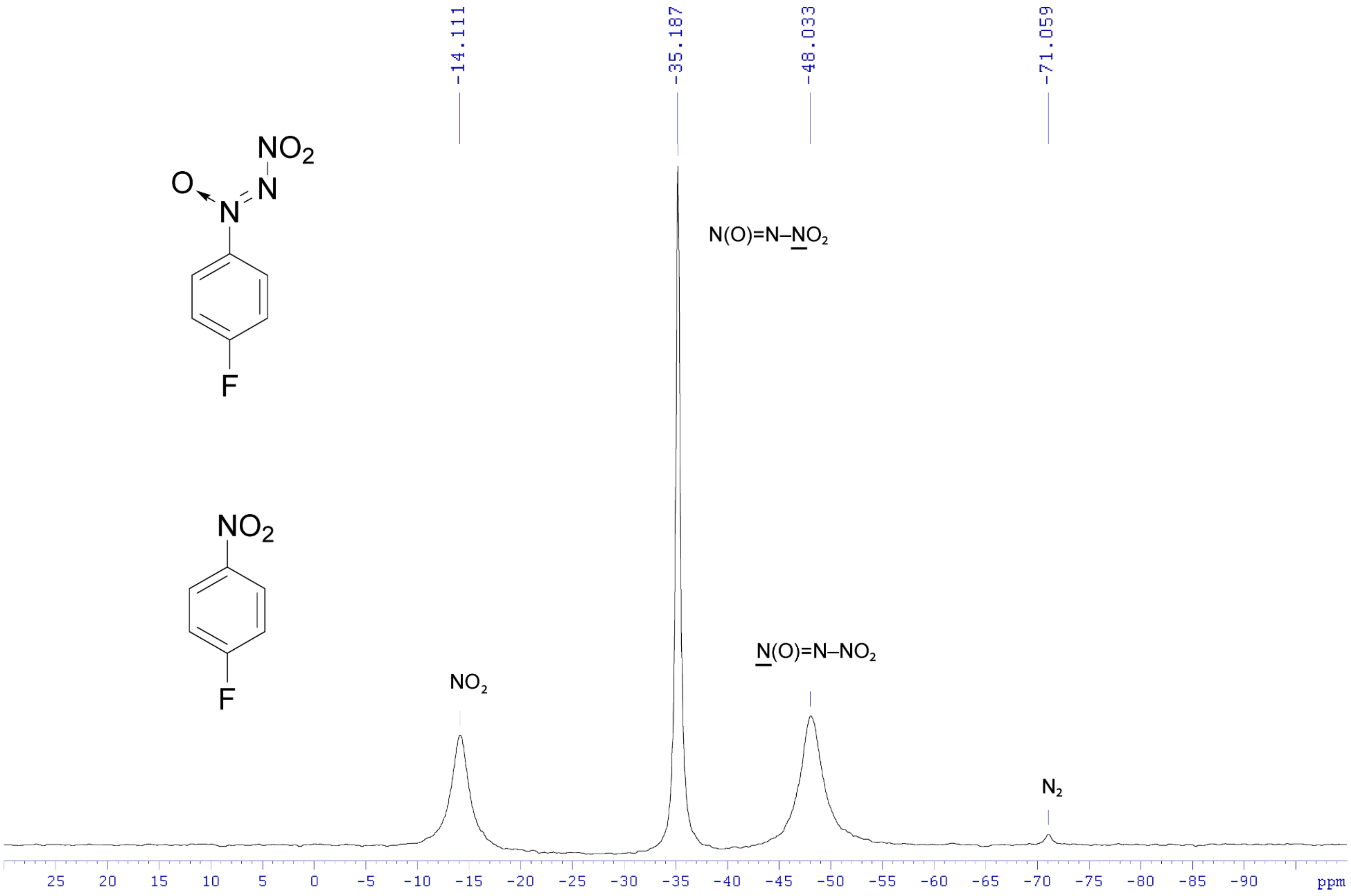

7.12.1 $^1$H NMR spectrum of compound 2m [600.13 MHz, $CDCl_3$]

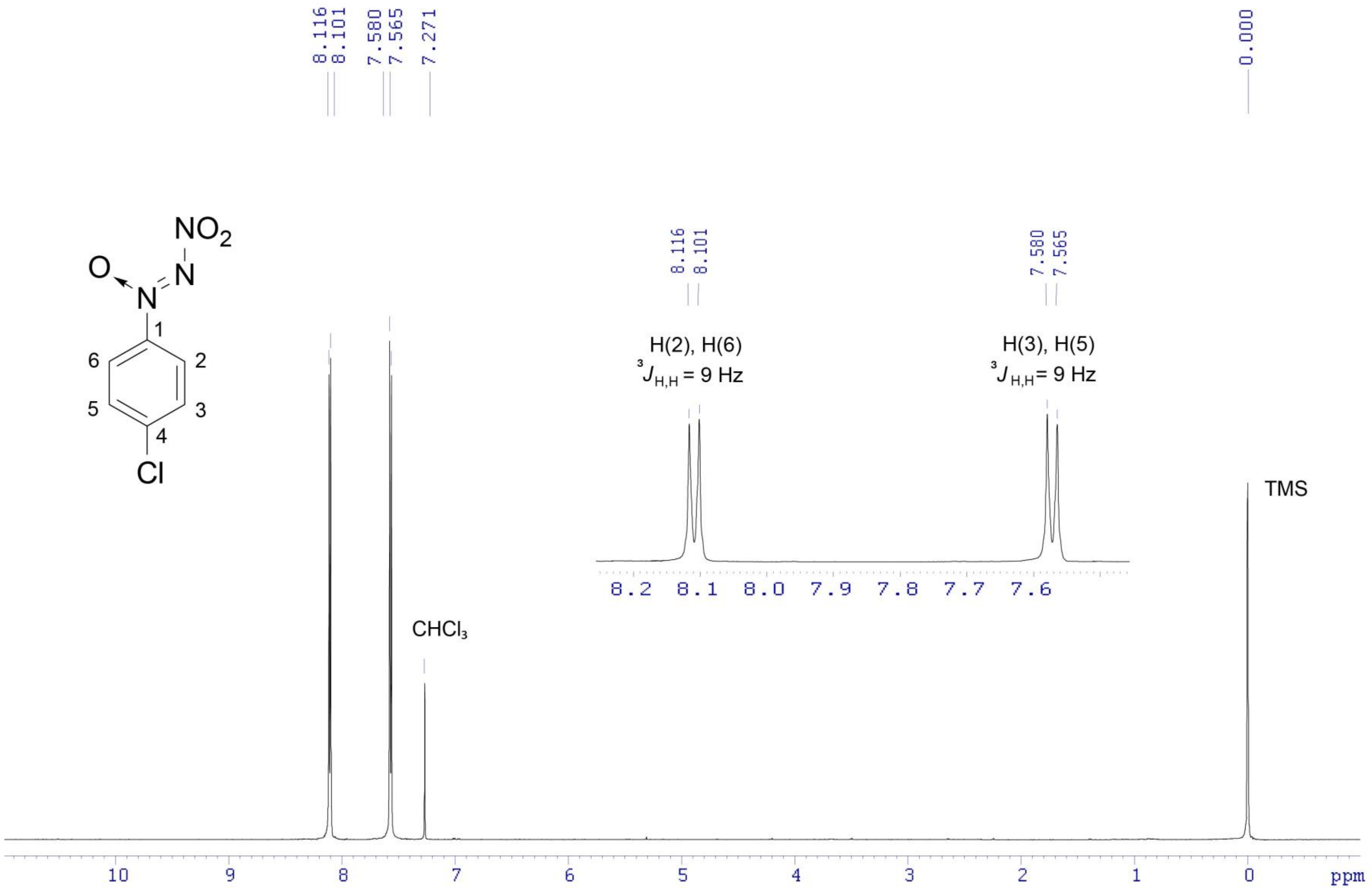

**7.12.2 $^{13}$C NMR spectrum of compound 2m [150.90 MHz, $CDCl_3$]**

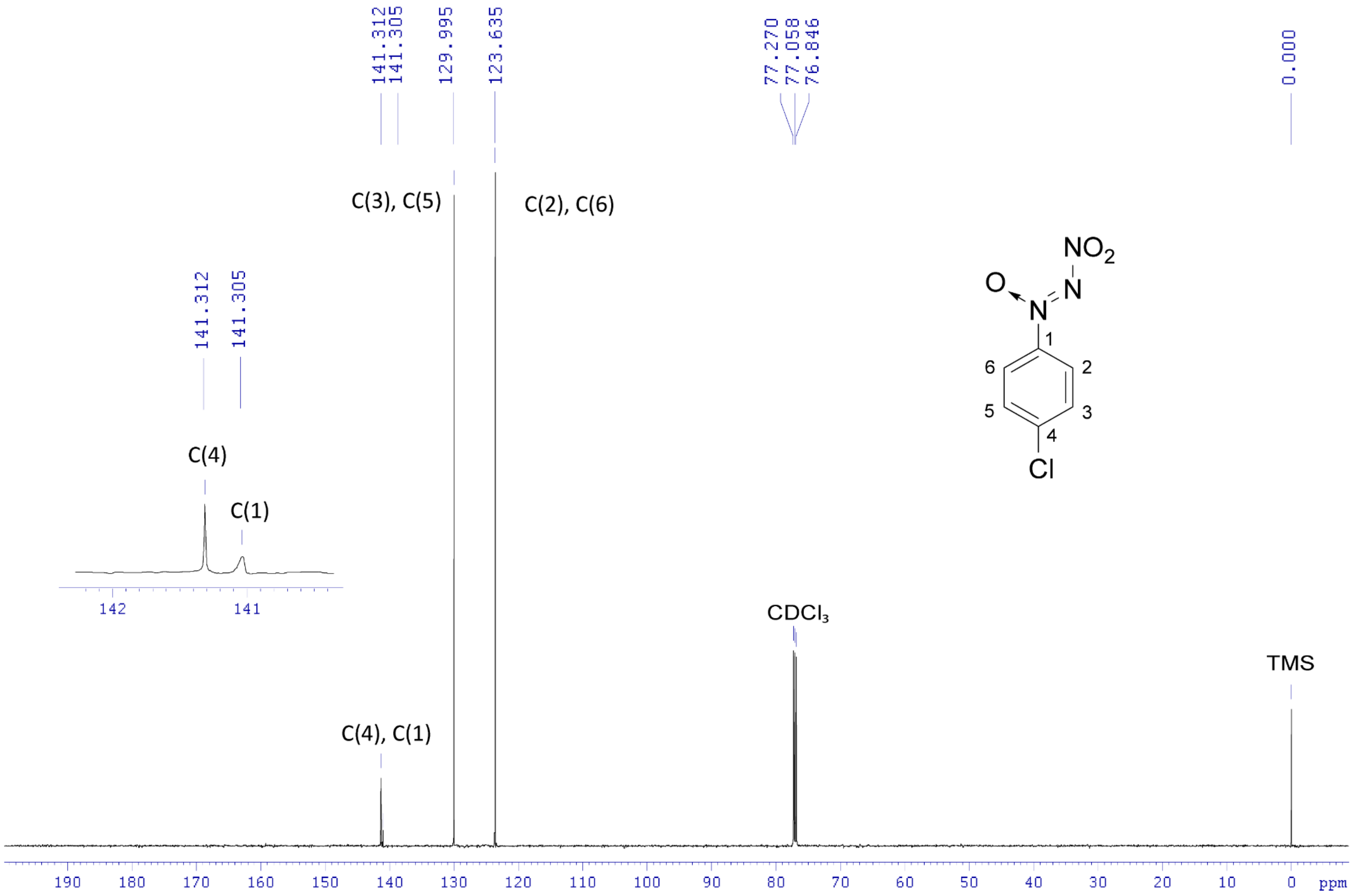

### 7.12.3 {$^1H$–$^{13}C$} HSQC spectrum of compound 2m [600.13 MHz, $CDCl_3$]

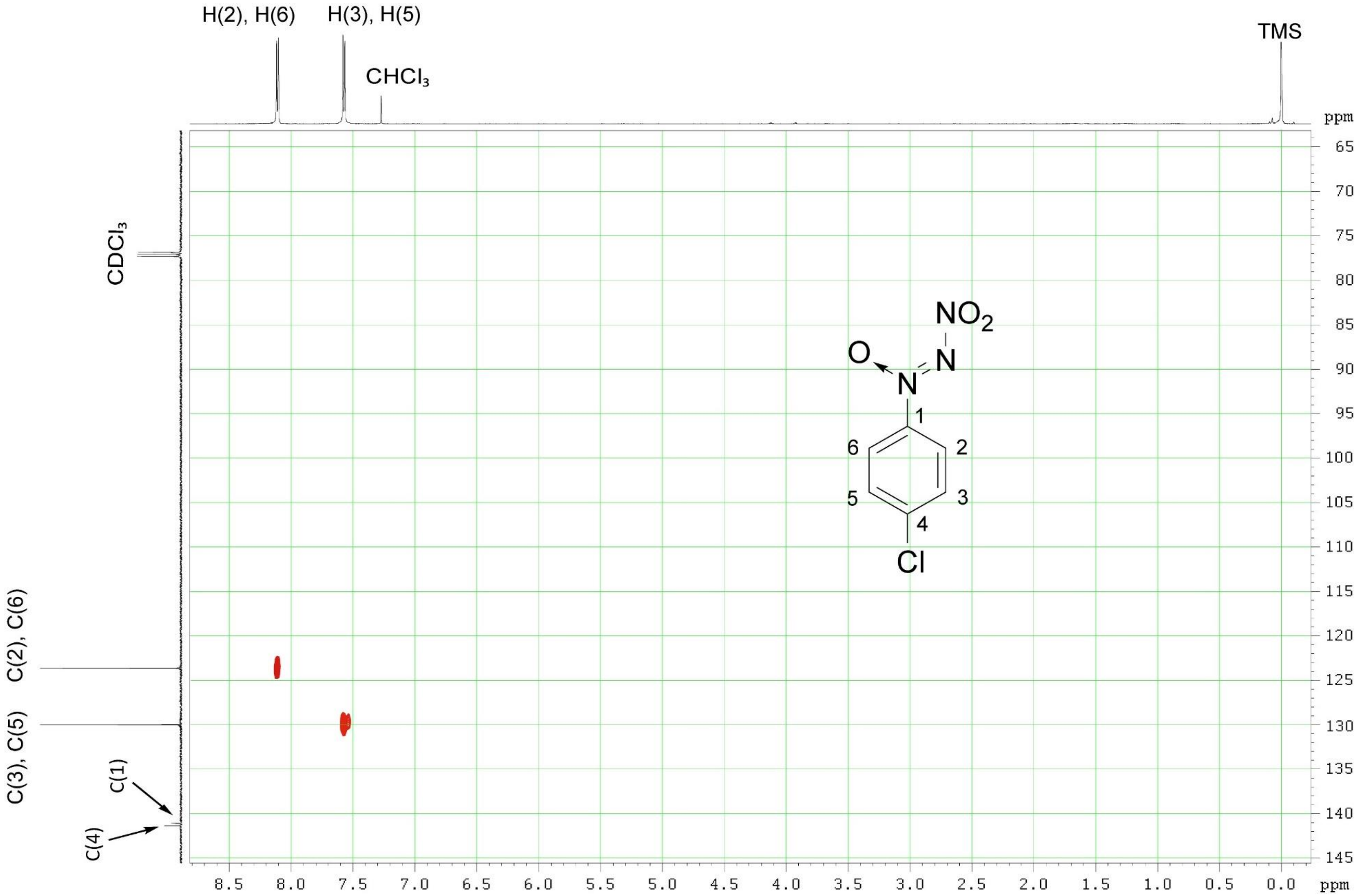

### 7.12.4 {$^1$H–$^{13}$C} HMBC spectrum of compound 2m [600.13 MHz, $CDCl_3$]

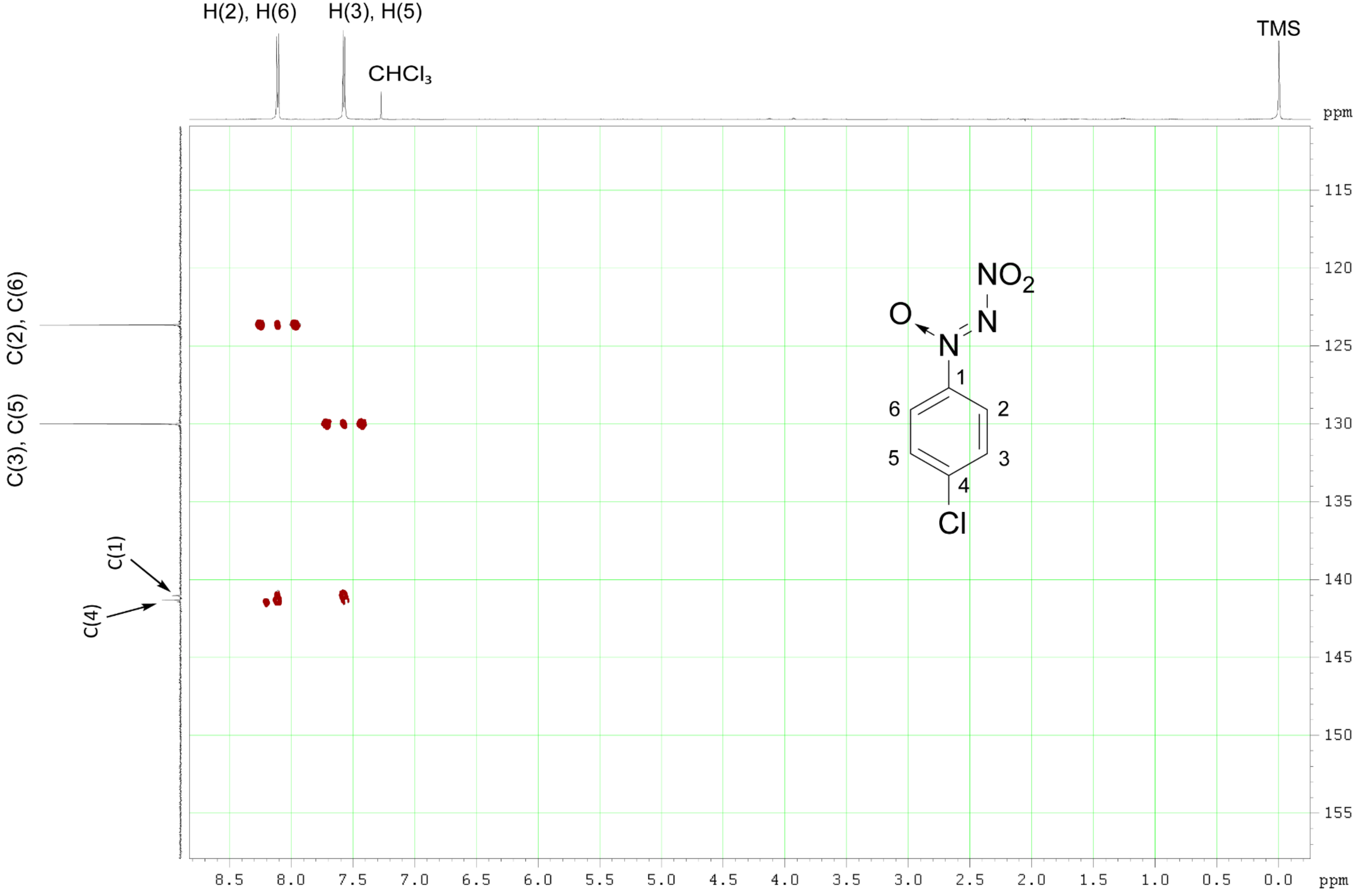

**7.12.5 $^{14}$N NMR spectrum of compound 2m [43.37 MHz, $CDCl_3$]**

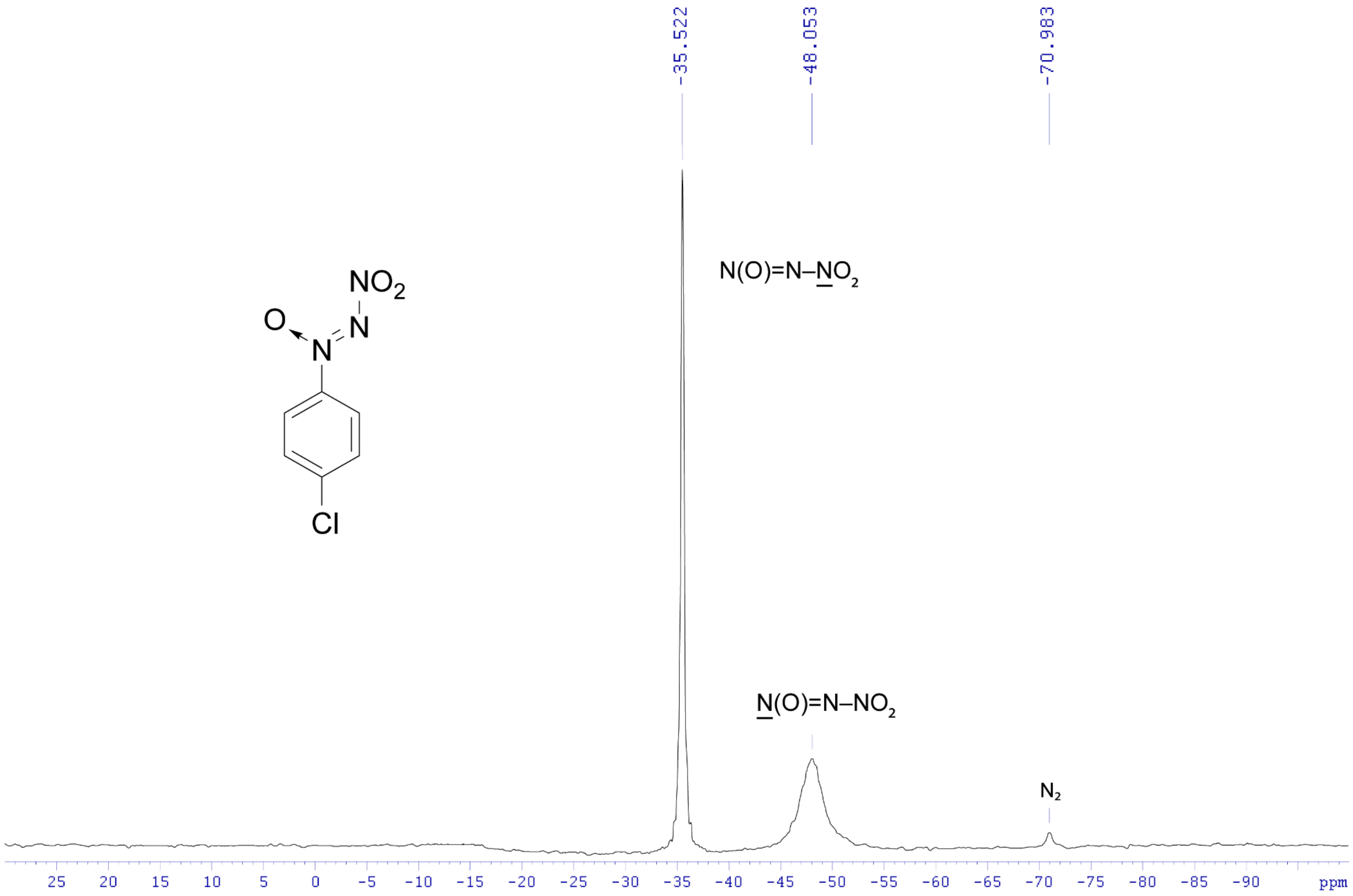

### 7.13.1 $^1$H NMR spectrum of compound 2n [500.13 MHz, $CDCl_3$]

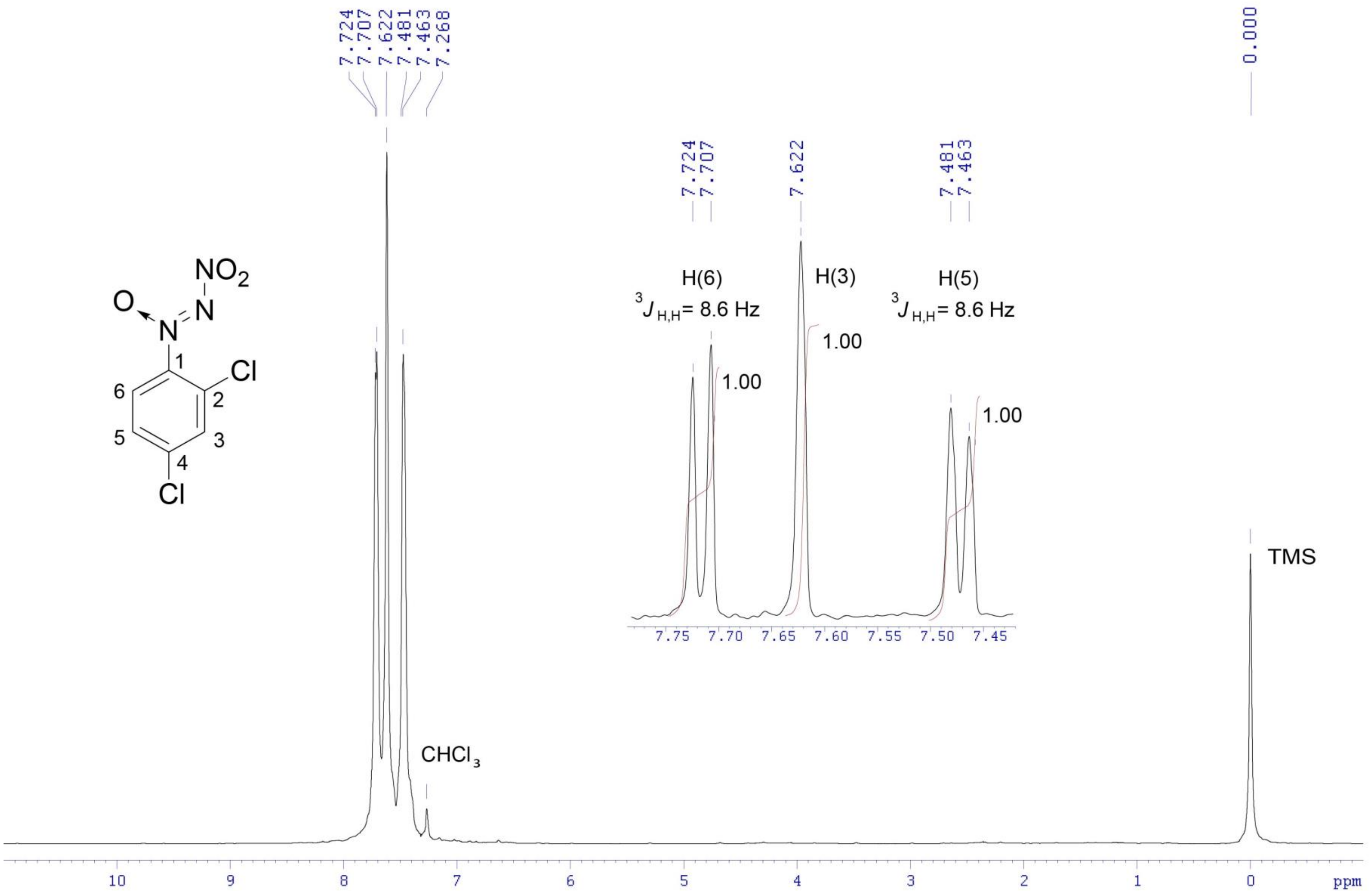

### 7.13.2 $^{13}$C NMR spectrum of compound 2n [125.76 MHz, $CDCl_3$]

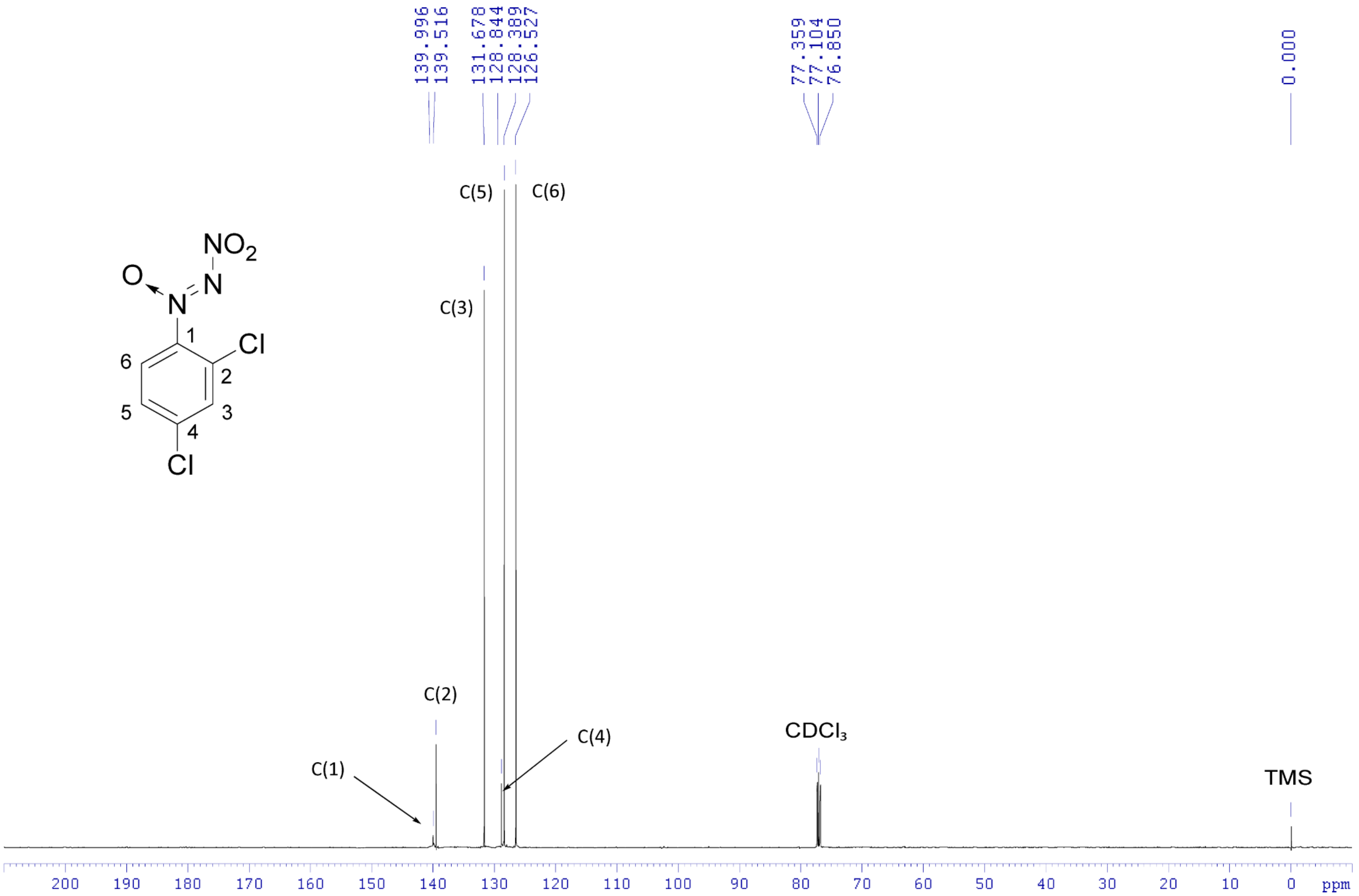

### 7.13.3 {$^{1}H$–$^{13}C$} HSQC spectrum of compound 2n [500.13 MHz, $CDCl_3$]

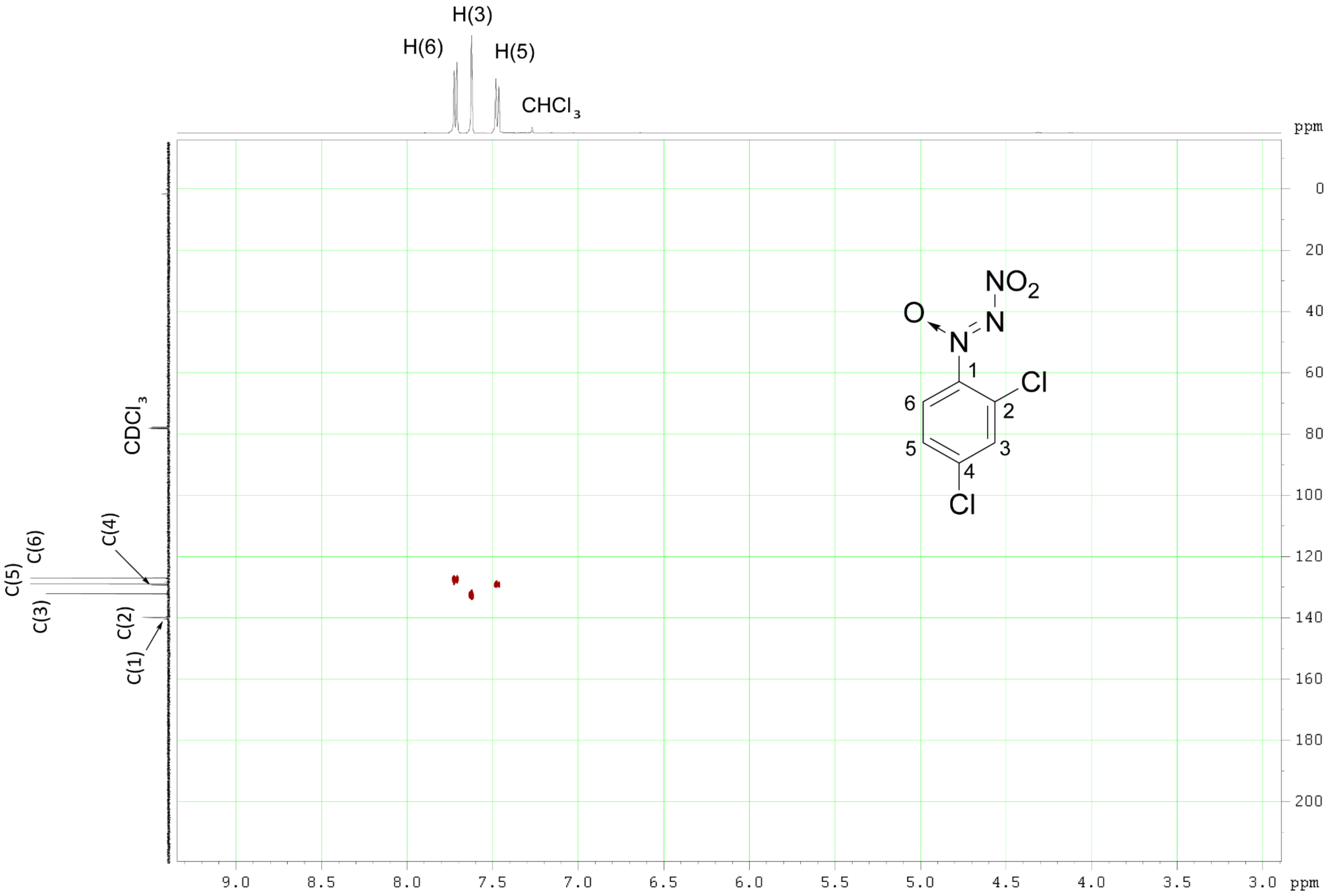

### 7.13.4 {$^1$H–$^{13}$C} HMBC spectrum of compound 2n [500.13 MHz, $CDCl_3$]

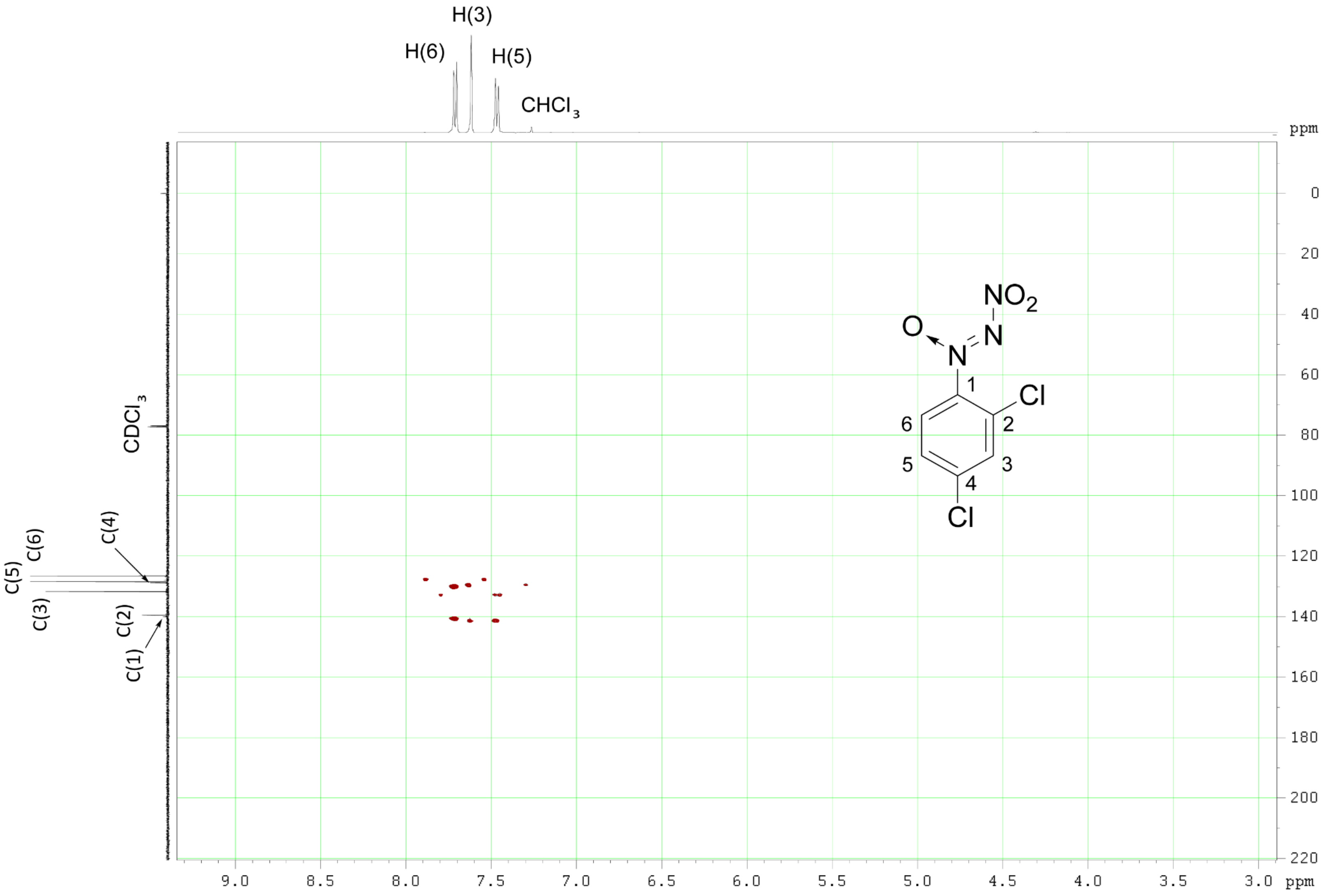

### 7.13.5 $^{14}$N NMR spectrum of compound 2n [36.14 MHz, $CDCl_3$]

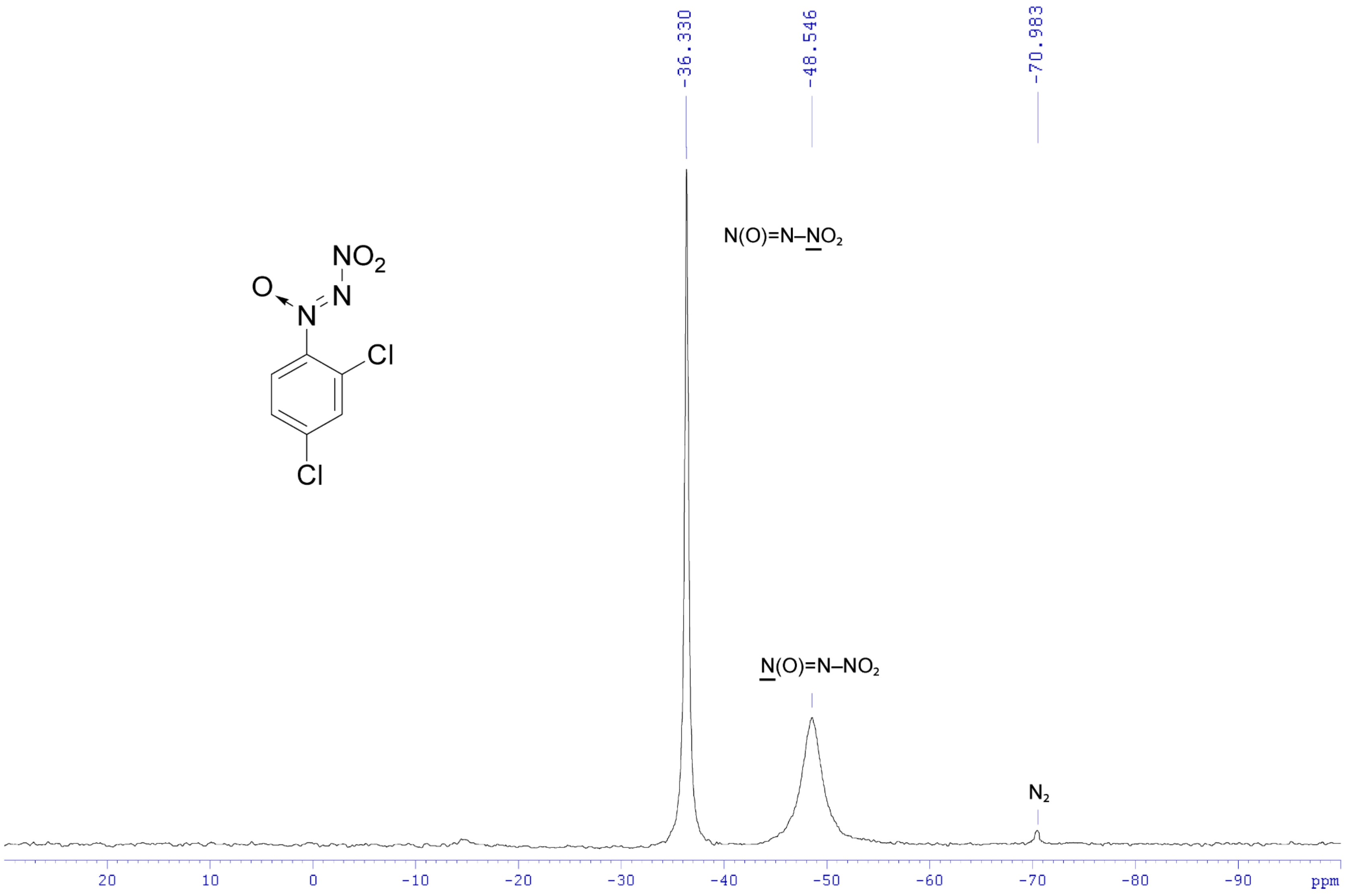

### 7.14.1 $^{1}$H NMR spectrum of compound 2o [300.13 MHz, $CDCl_3$]

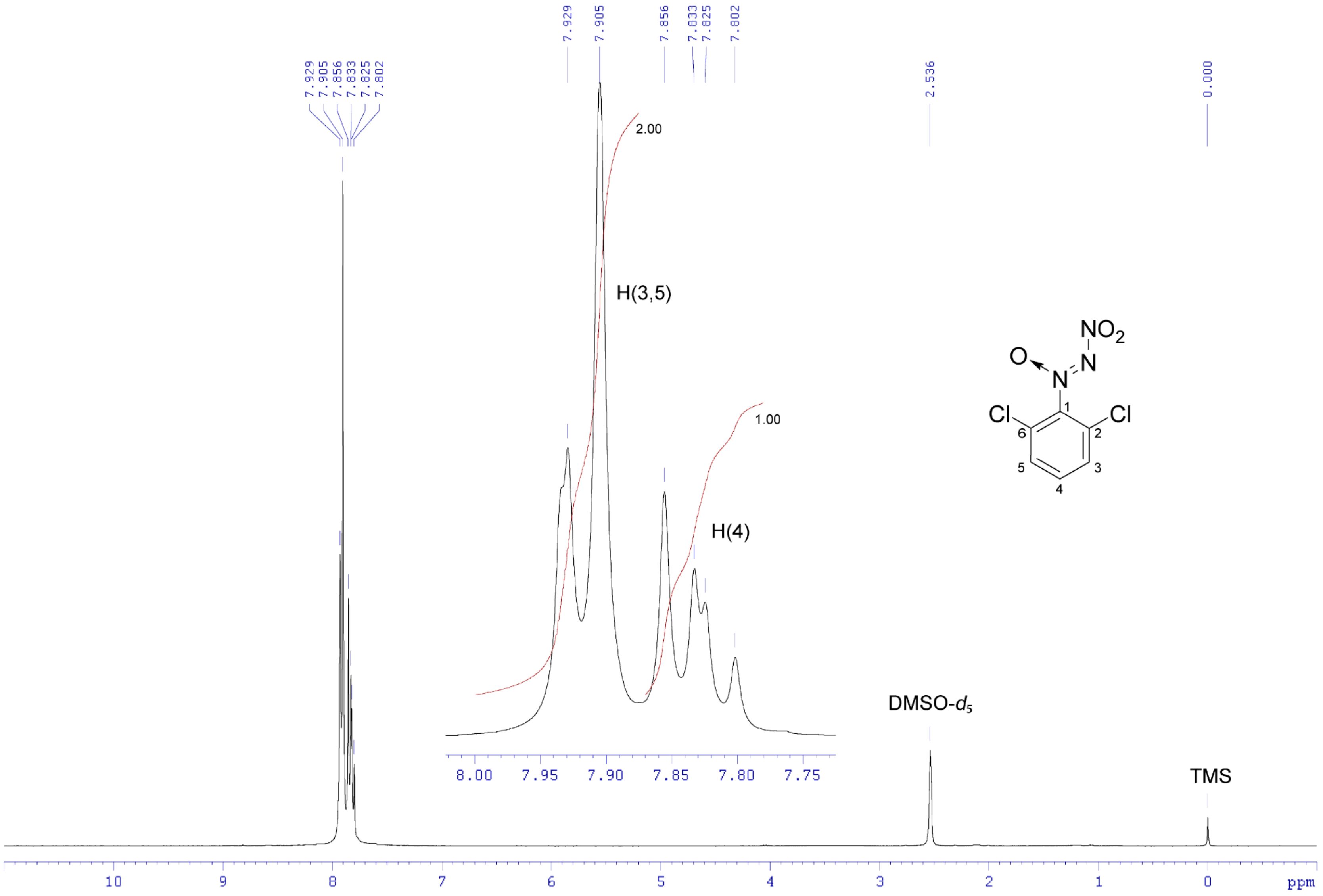

### 7.14.2 $^{13}C$ NMR spectrum of compound 2o [75.49 MHz, $CDCl_3$]

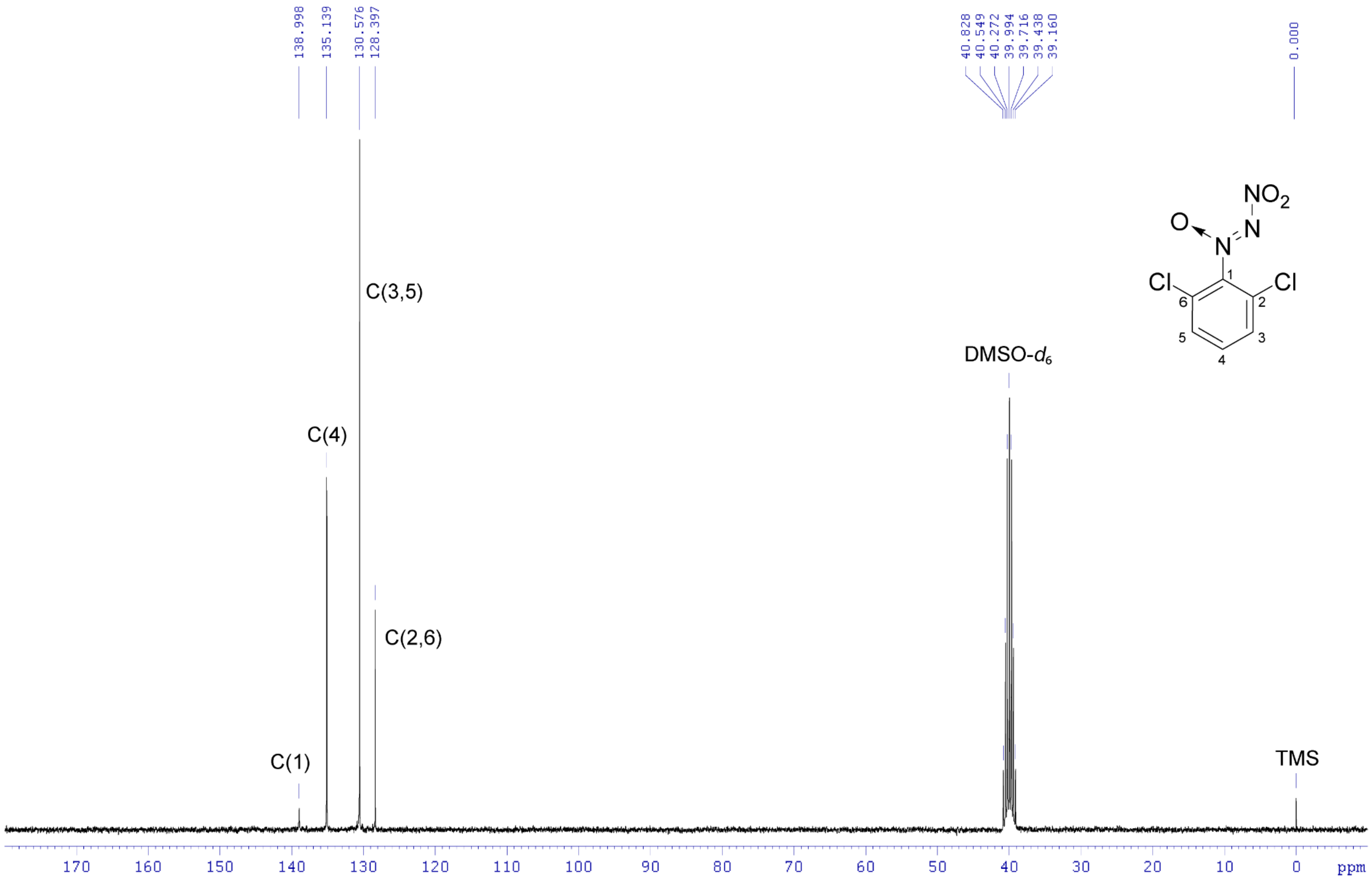

### 7.14.3 {$^{1}$H–$^{13}$C} HSQC spectrum of compound 2o [300.13 MHz, $CDCl_3$]

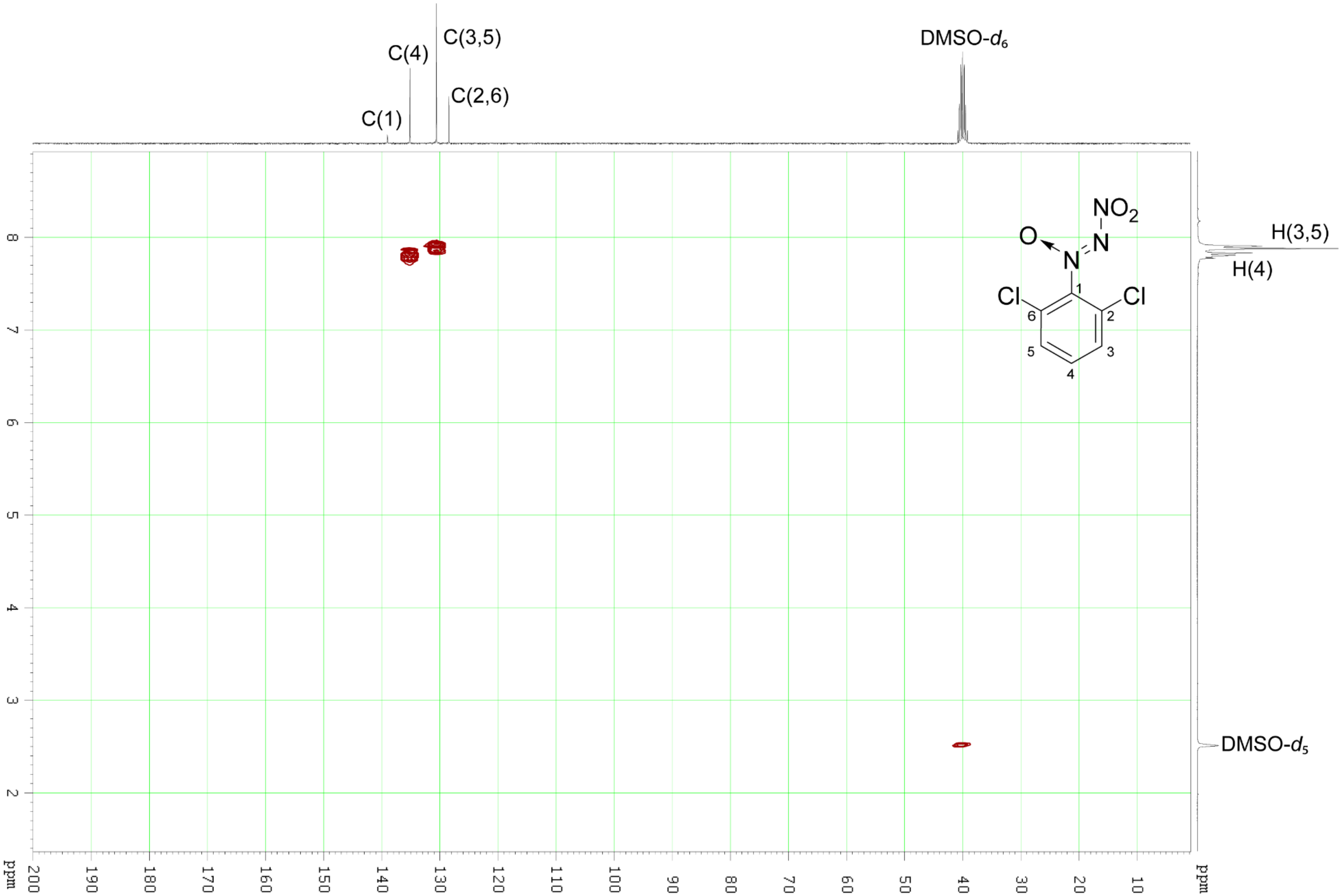

### 7.14.4 {$^{1}H$–$^{13}C$} HMBC spectrum of compound 2o [300.13 MHz, $CDCl_3$]

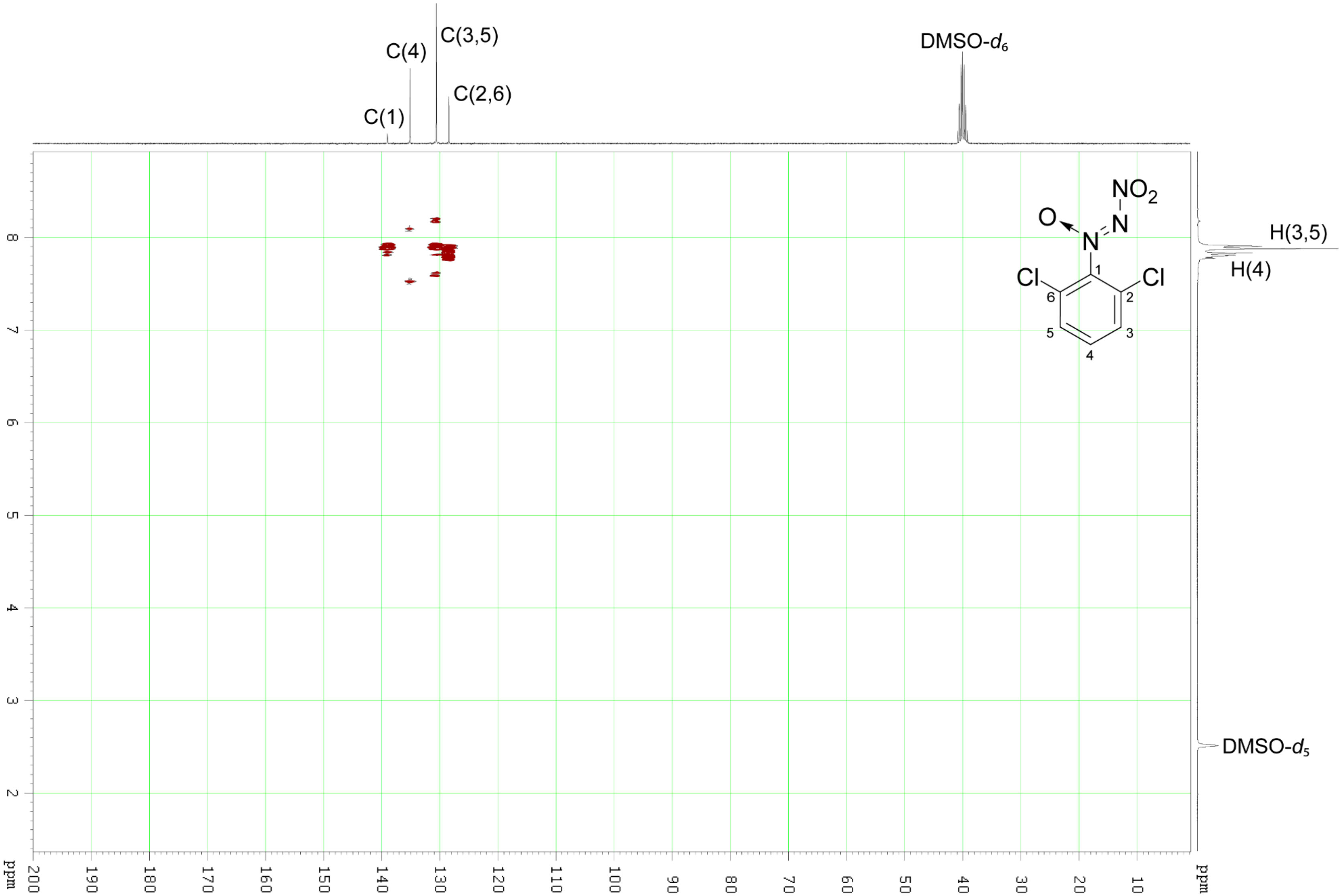

### 7.14.5 $^{14}N$ NMR spectrum of compound 2o [36.14 MHz, $CDCl_3$]

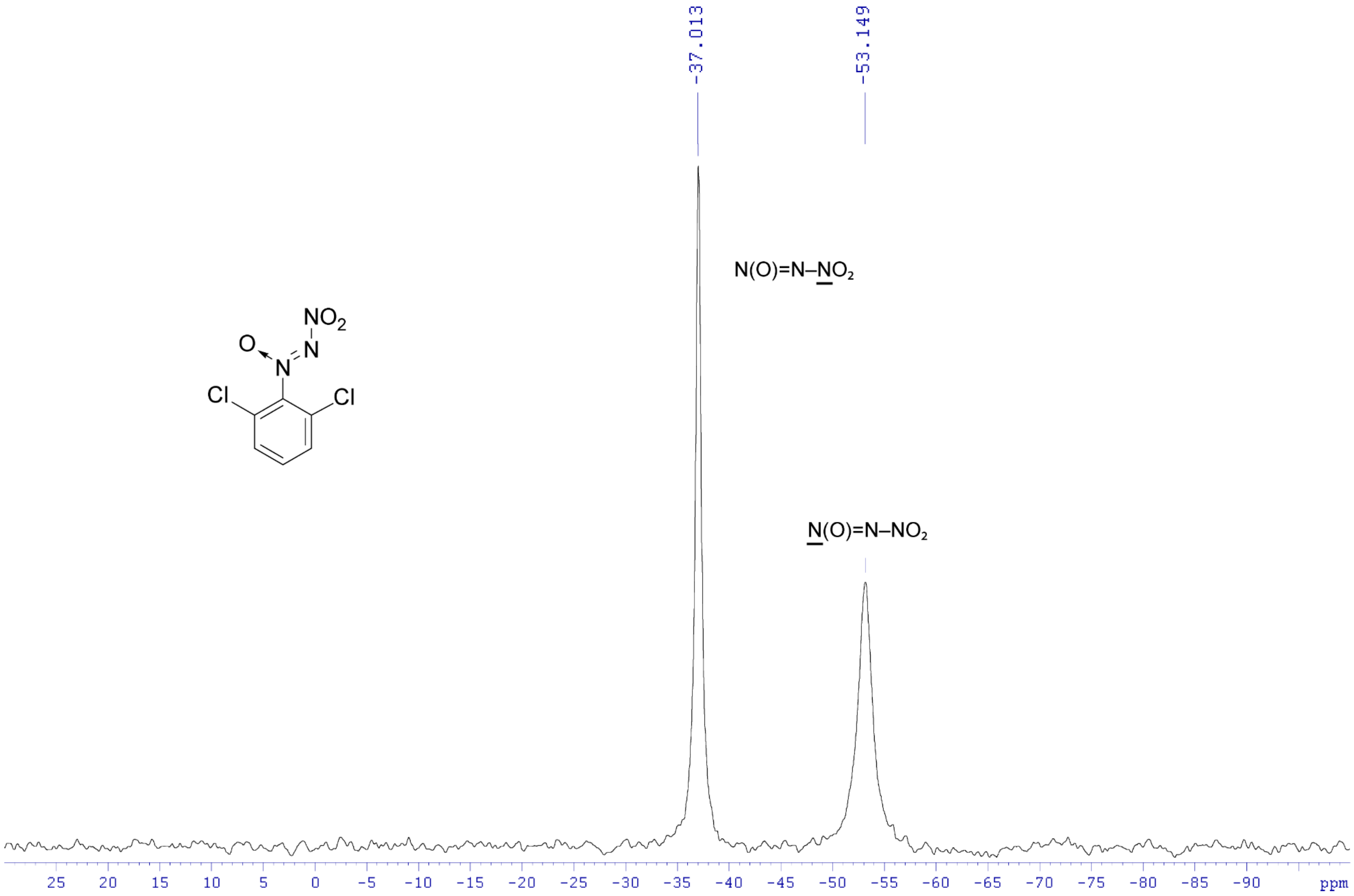

### 7.15.1 ¹H NMR spectrum of compound 2p [500.13 MHz, $CDCl_3$]

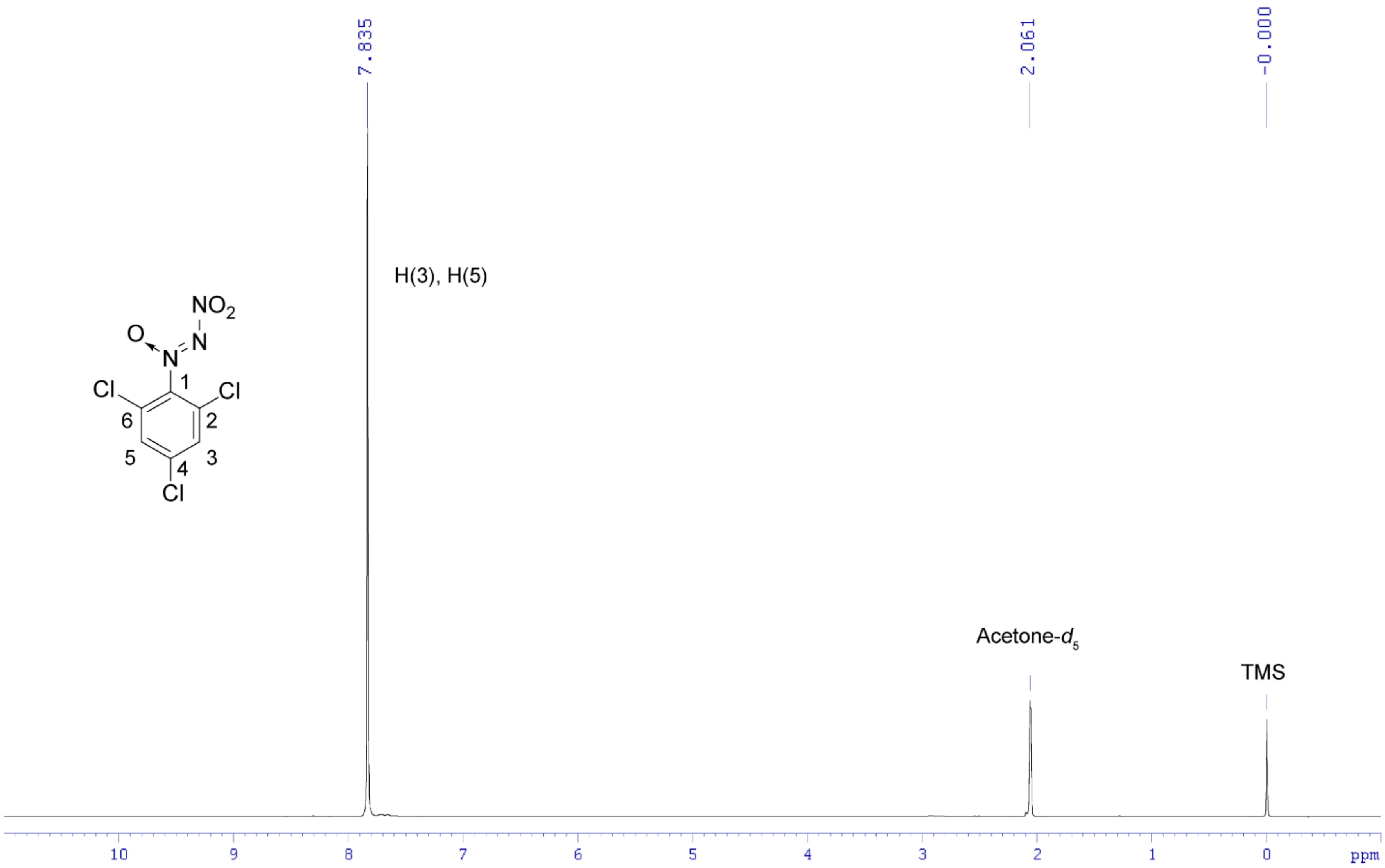

### 7.15.2 $^{13}$C NMR spectrum of compound 2p [125.76 MHz, $CDCl_3$]

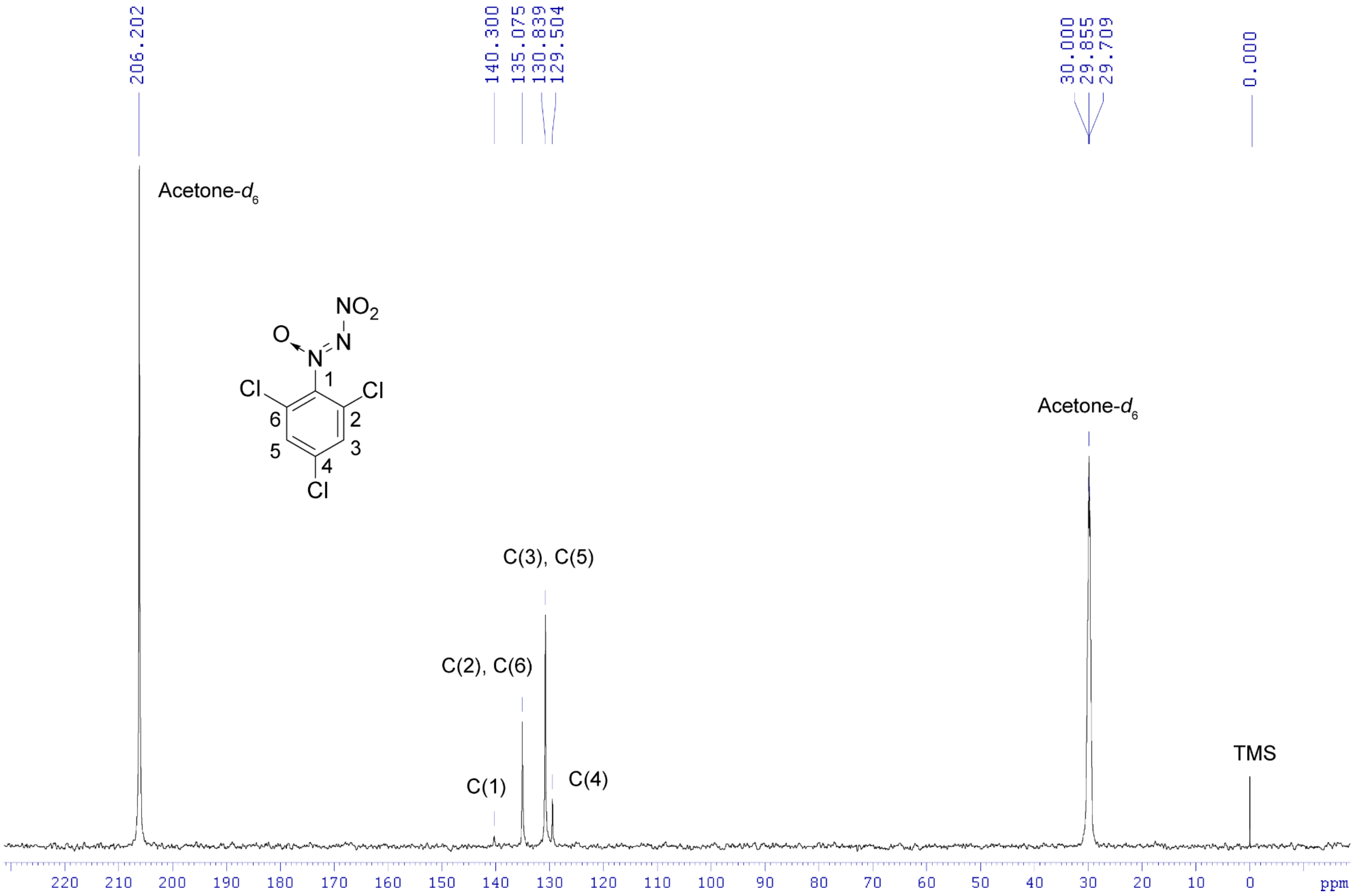

**7.15.3 {$^1$H–$^{13}$C} HSQC spectrum of compound 2p [500.13 MHz, $CDCl_3$]**

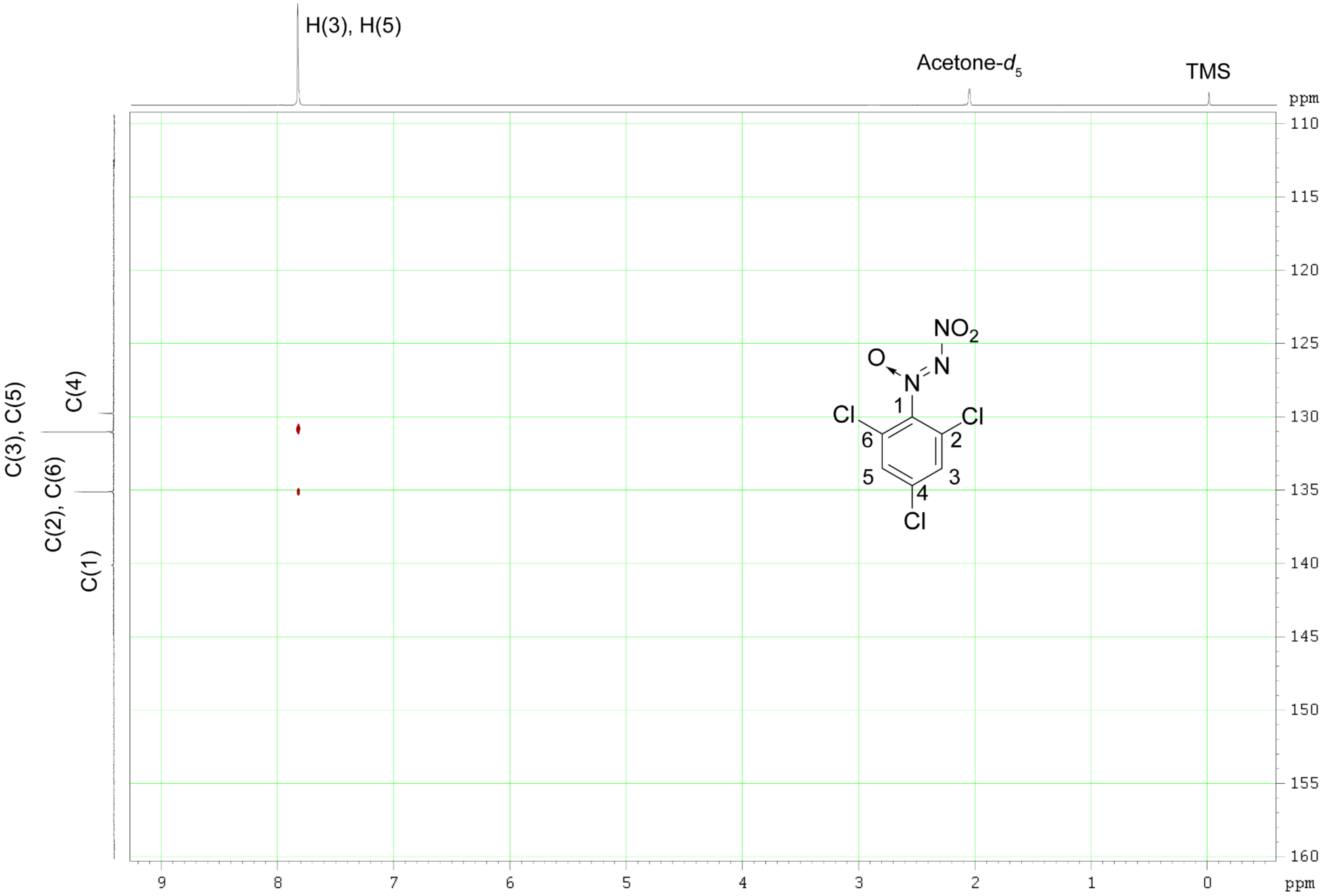

7.15.4 {$^{1}$H–$^{13}$C} HMBC spectrum of compound 2p [500.13 MHz, $CDCl_3$]

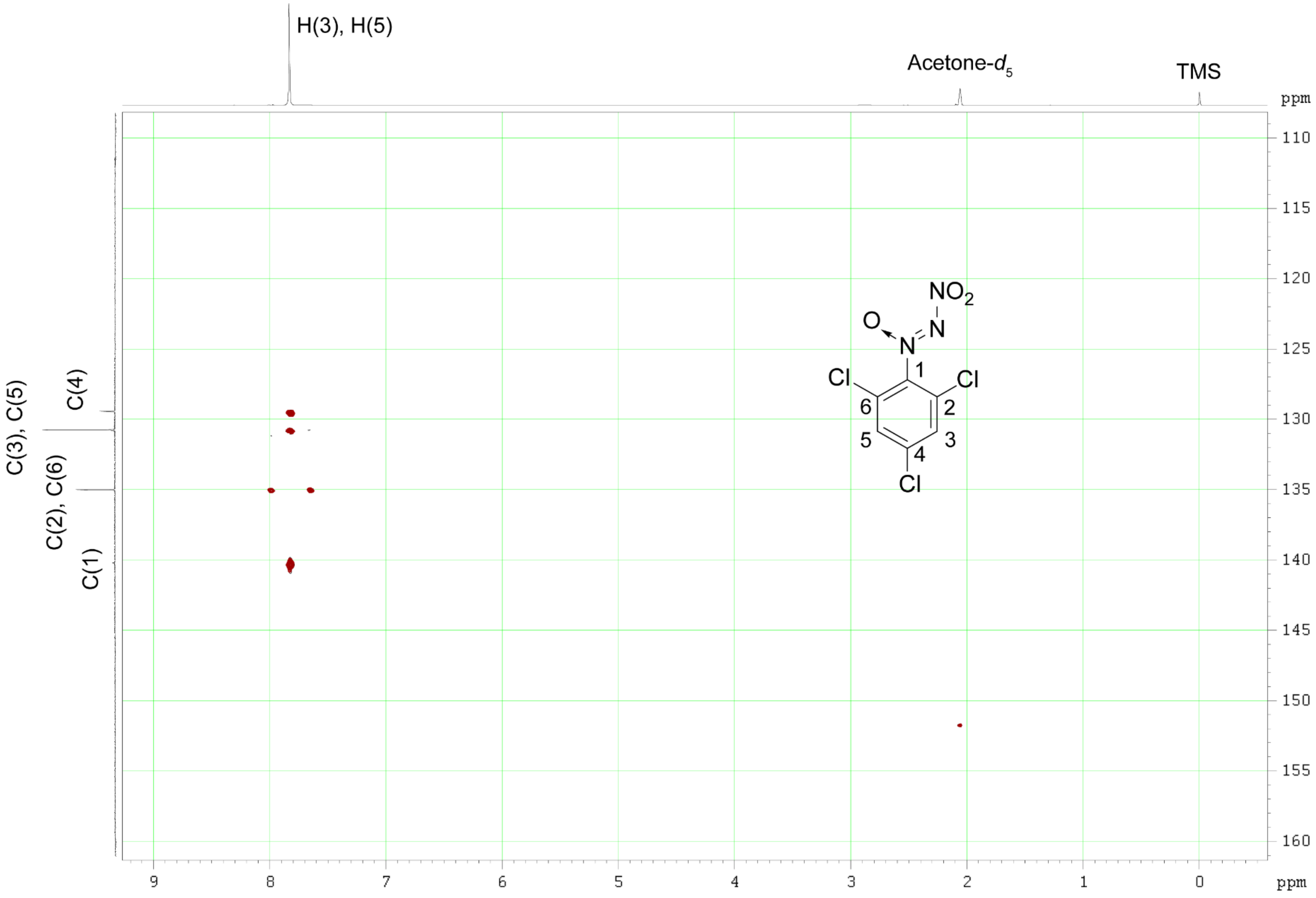

### 7.15.5 $^{14}N$ NMR spectrum of compound 2p [36.14 MHz, $CDCl_3$]

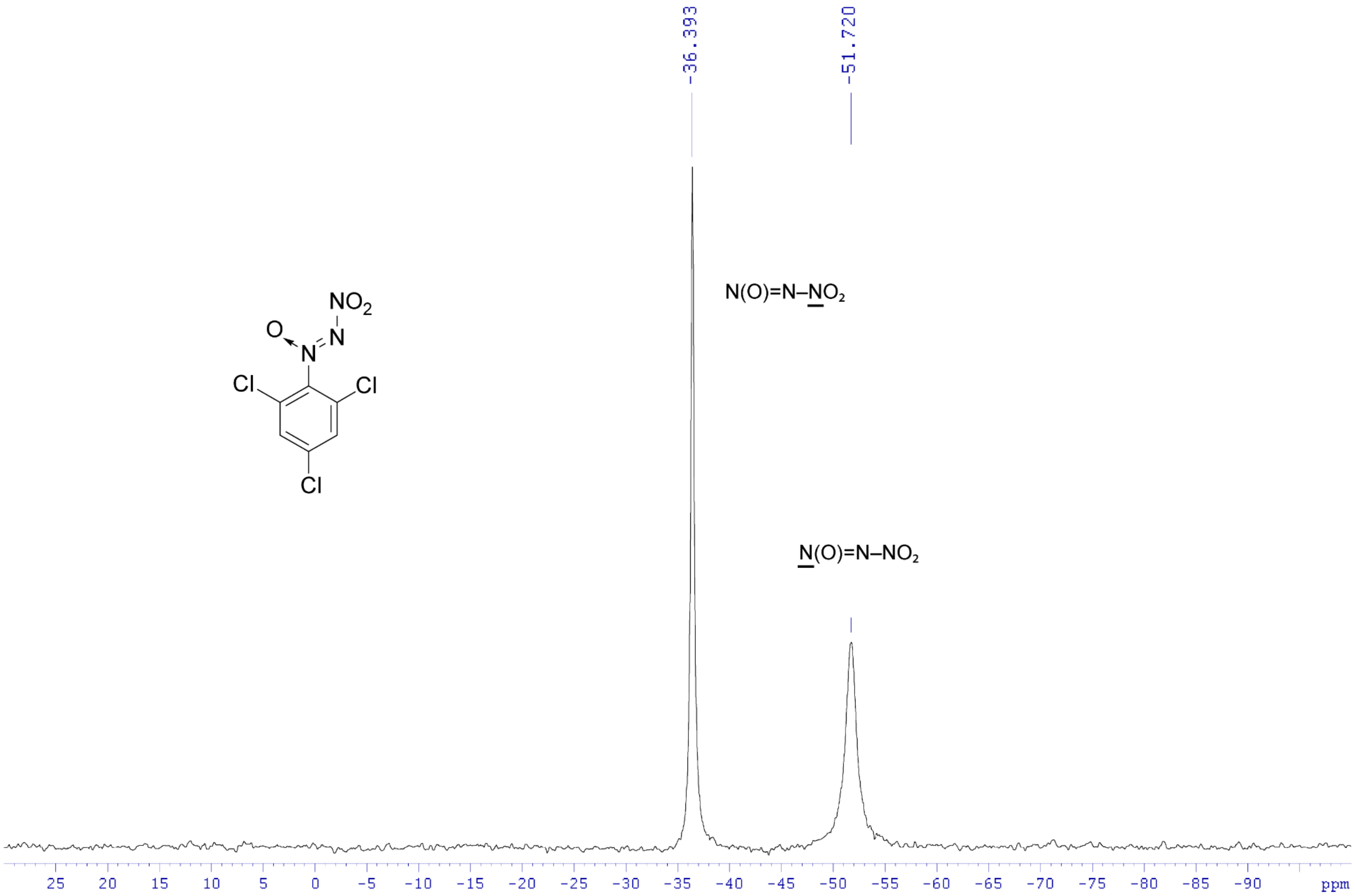

**7.16.1 $^1$H NMR spectrum of compound 2q [600.13 MHz, $CDCl_3$]**

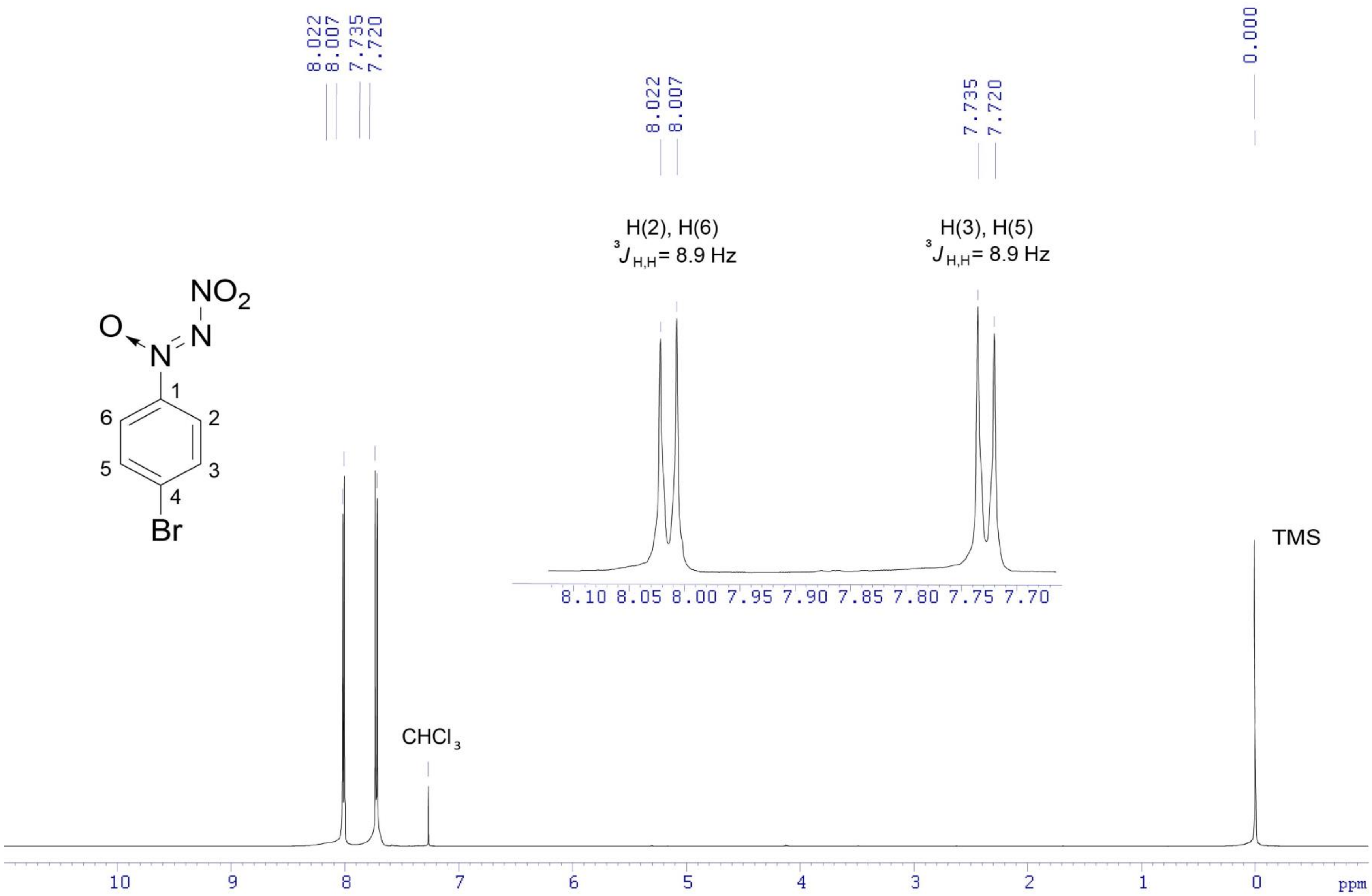

**7.16.2 $^{13}$C NMR spectrum of compound 2q [150.90 MHz, $CDCl_3$]**

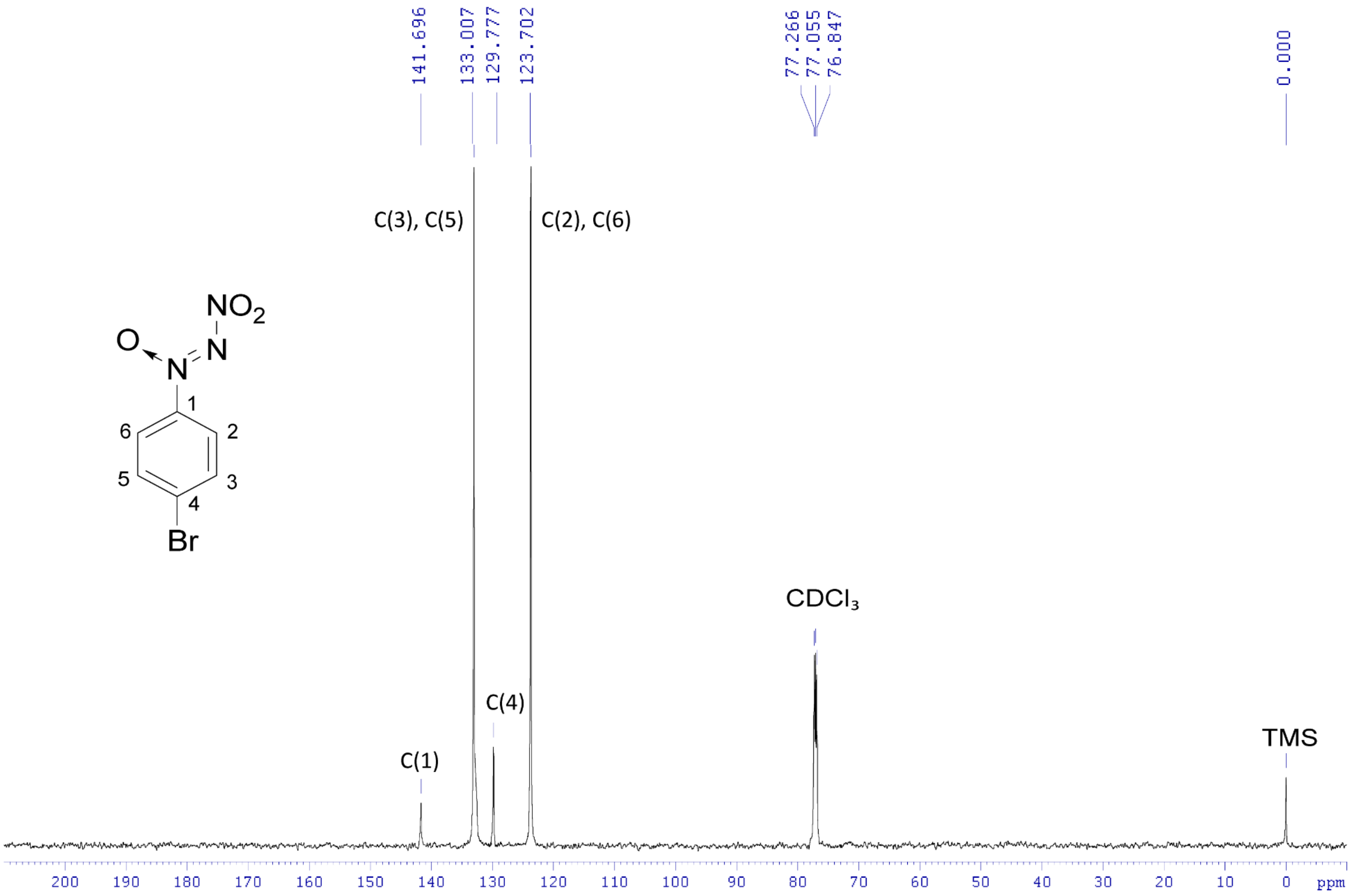

### 7.16.3 {$^1H$–$^{13}C$} HSQC spectrum of compound 2q [600.13 MHz, $CDCl_3$]

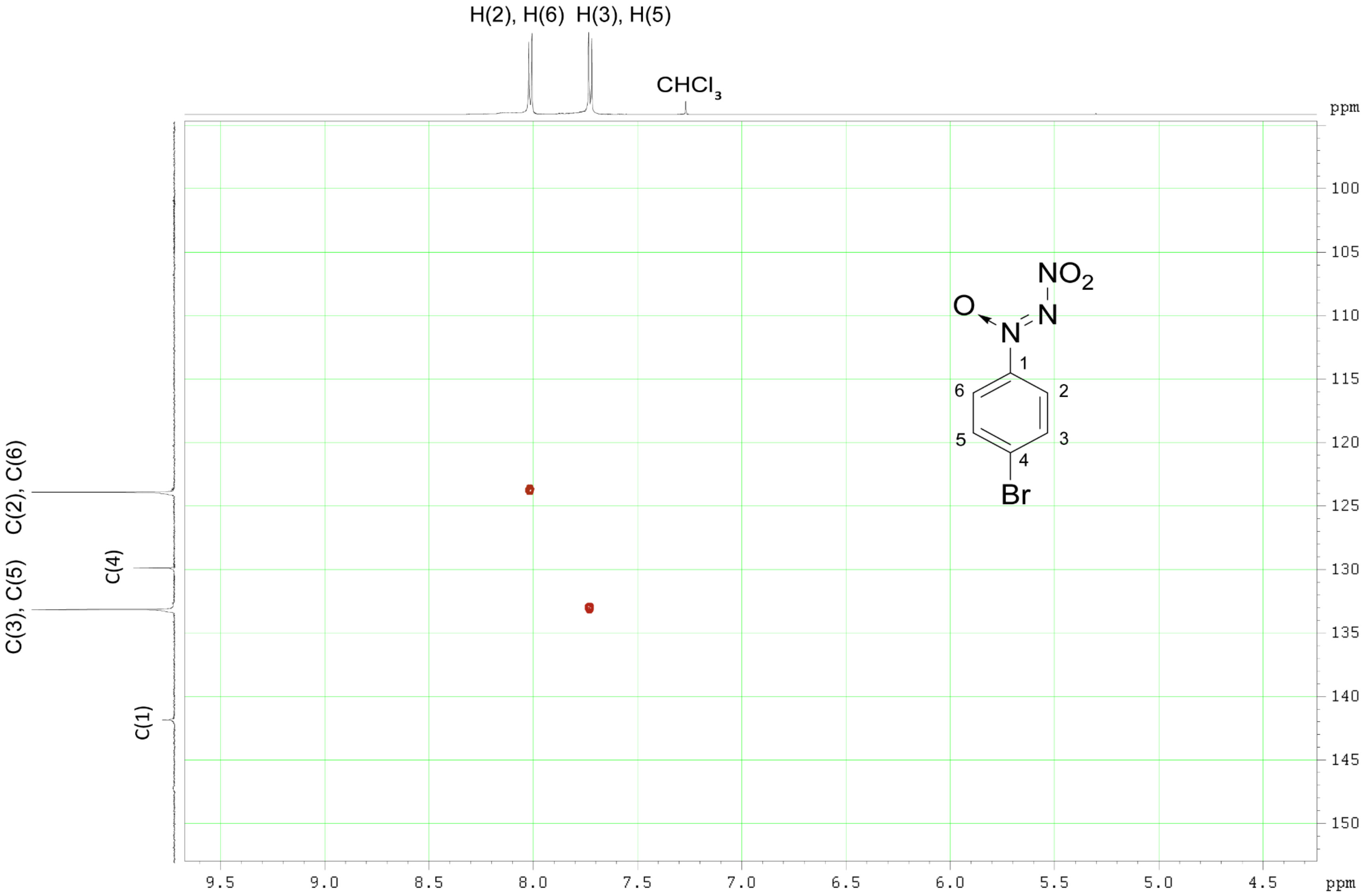

### 7.16.4 {$^1H$–$^{13}C$} HMBC spectrum of compound 2q [600.13 MHz, $CDCl_3$]

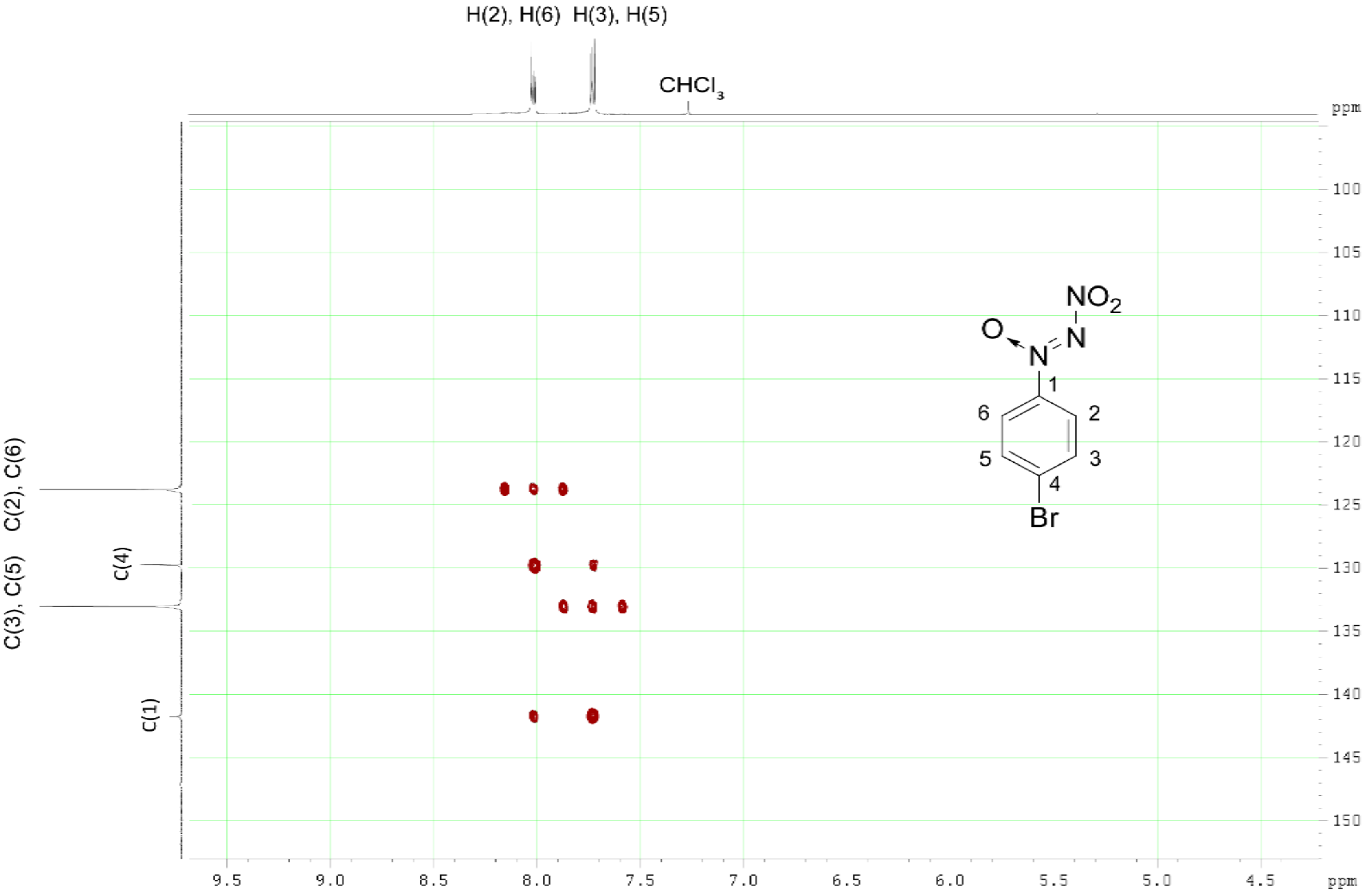

7.16.5 $^{14}$N NMR spectrum of compound 2q [43.37 MHz, $CDCl_3$]

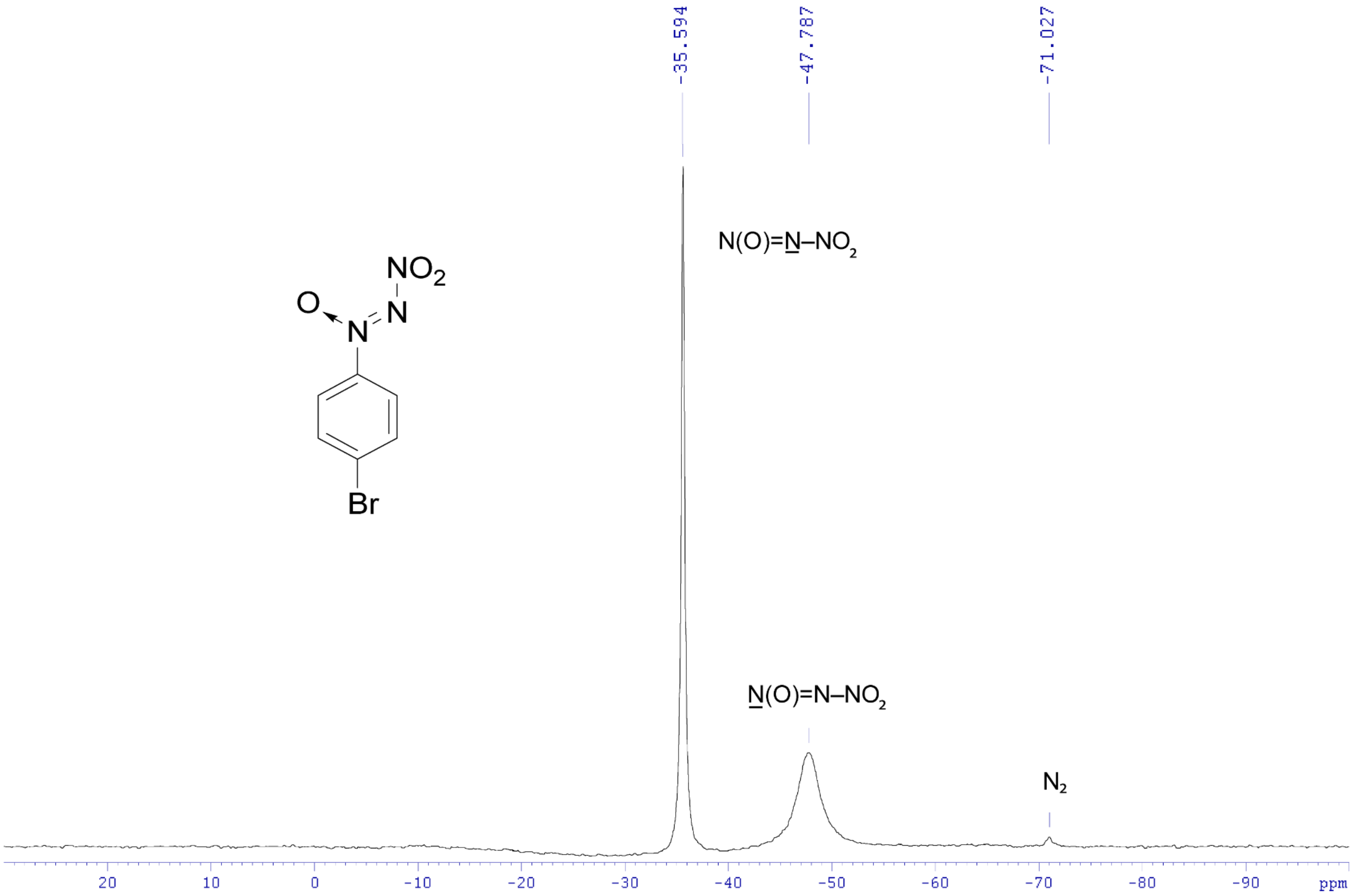

7.17.1 $^1$H NMR spectrum of compound 2r [600.13 MHz, $CDCl_3$]

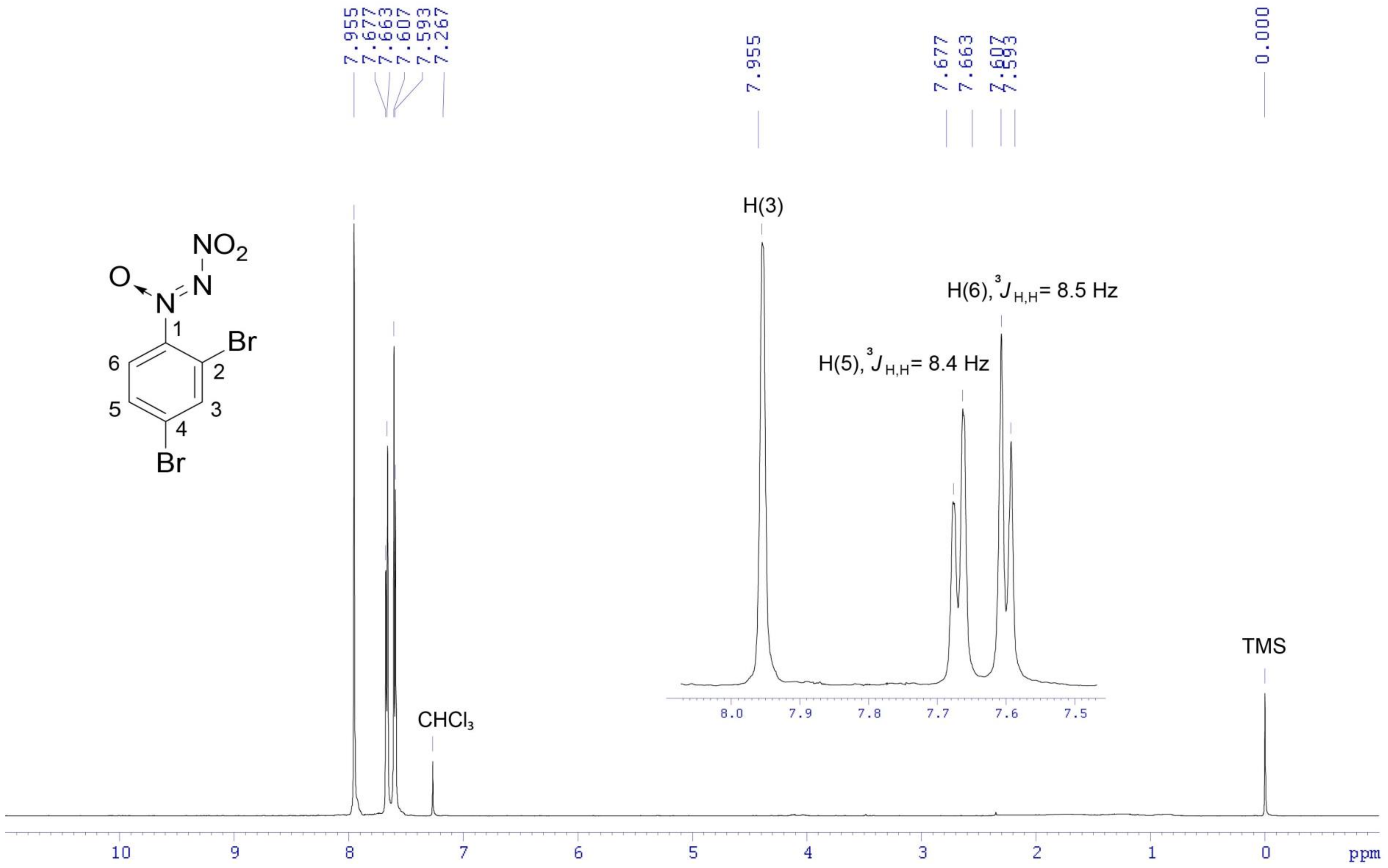

### 7.17.2 $^{13}C$ NMR spectrum of compound 2r [150.90 MHz, $CDCl_3$]

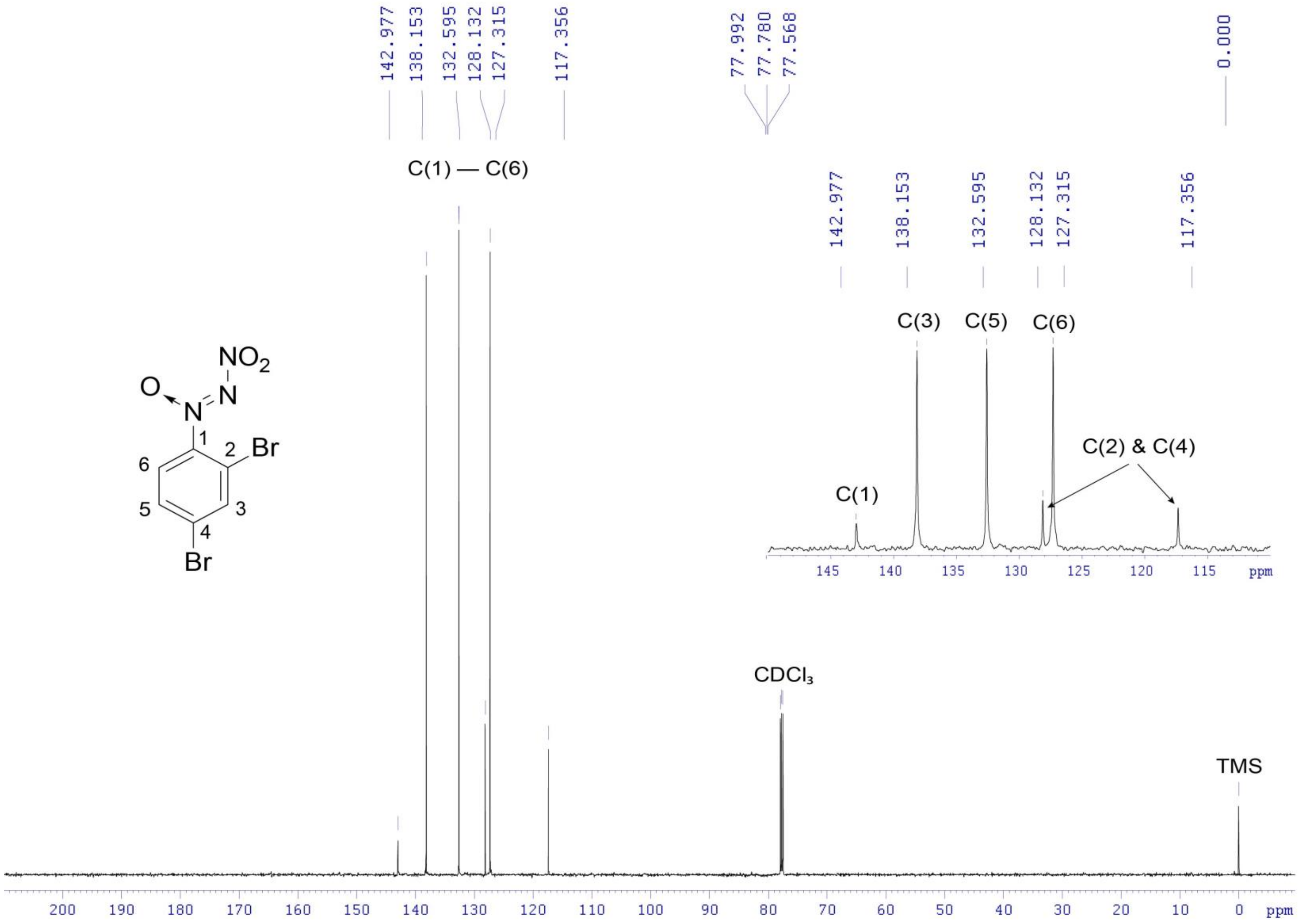

### 7.17.3 {$^{1}$H–$^{13}$C} HSQC spectrum of compound 2r [600.13 MHz, $CDCl_3$]

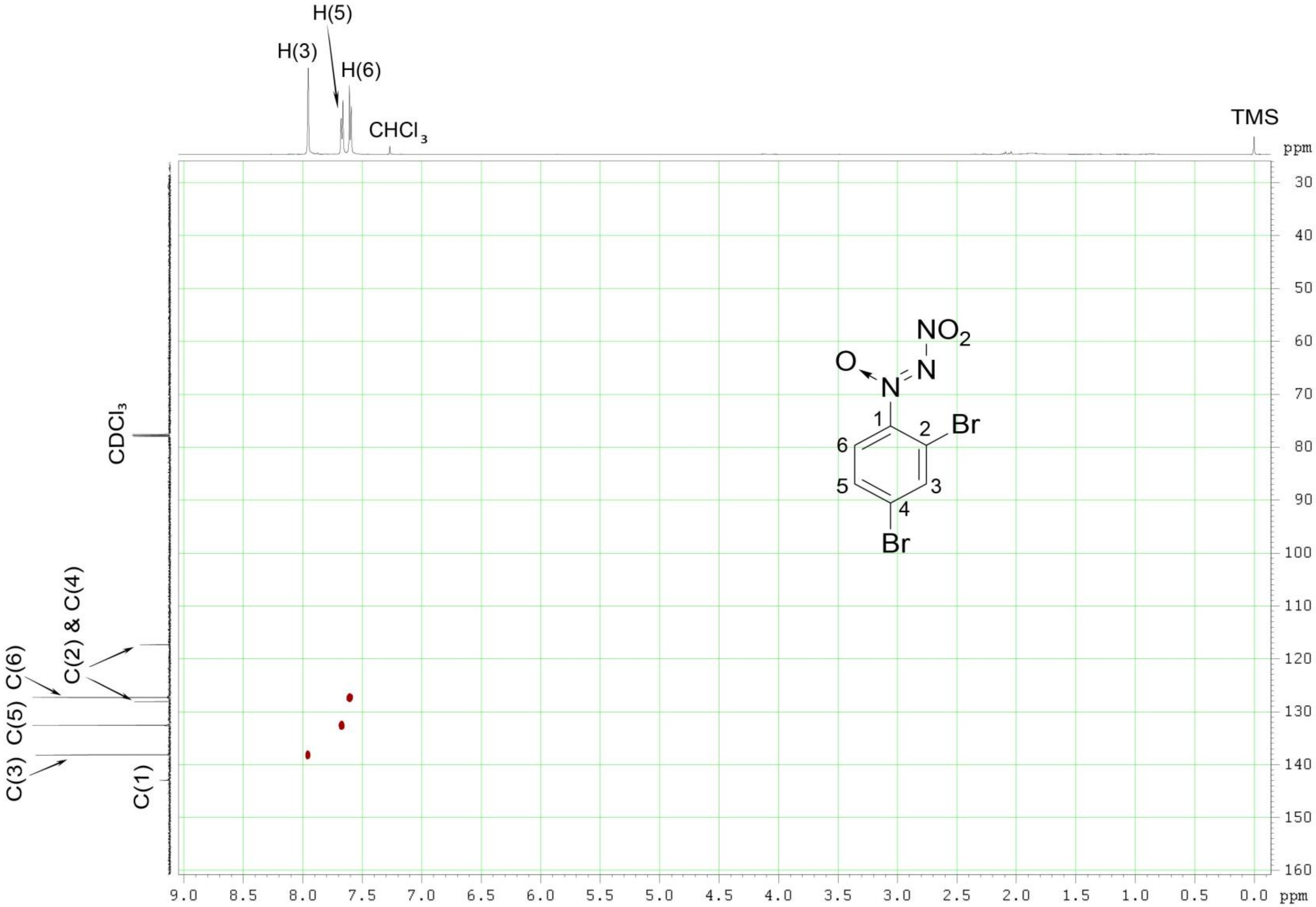

7.17.4 {$^{1}$H–$^{13}$C} HMBC spectrum of compound 2r [600.13 MHz, $CDCl_3$]

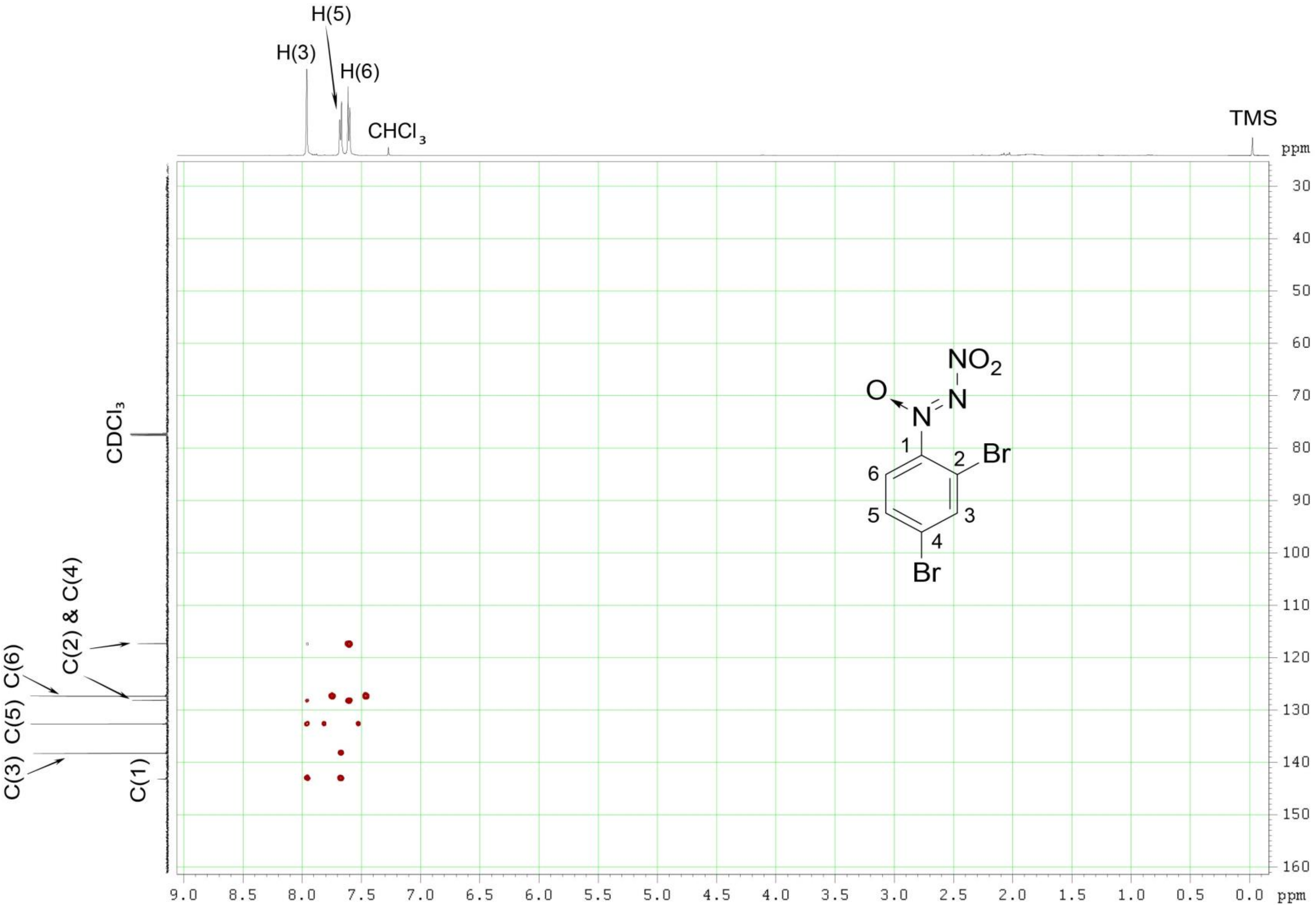

### 7.17.5 $^{14}N$ NMR spectrum of compound 2r [36.14 MHz, $CDCl_3$]

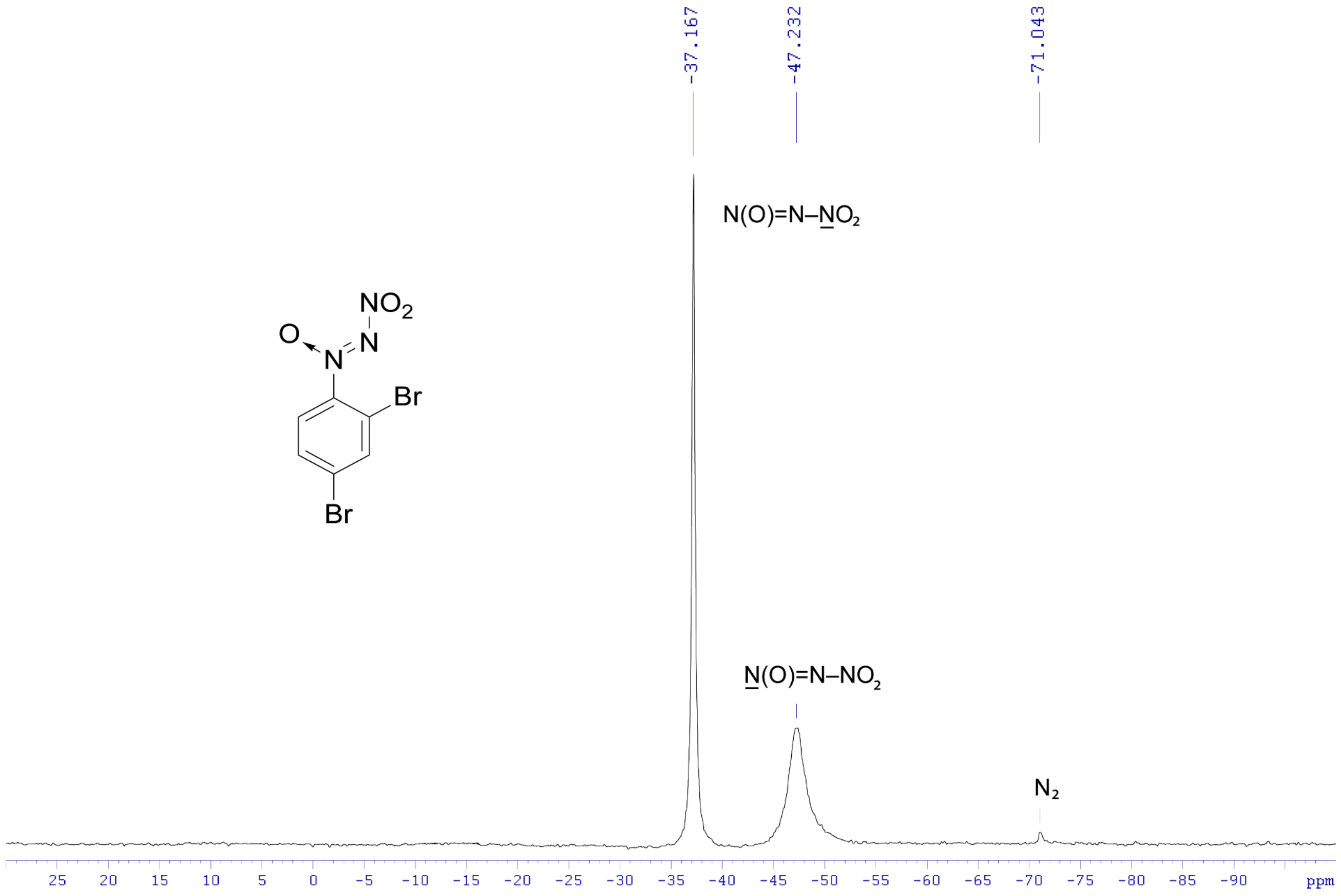

**7.18.1 $^{1}$H NMR spectrum of compound 2s [500.13 MHz, $CDCl_3$]**

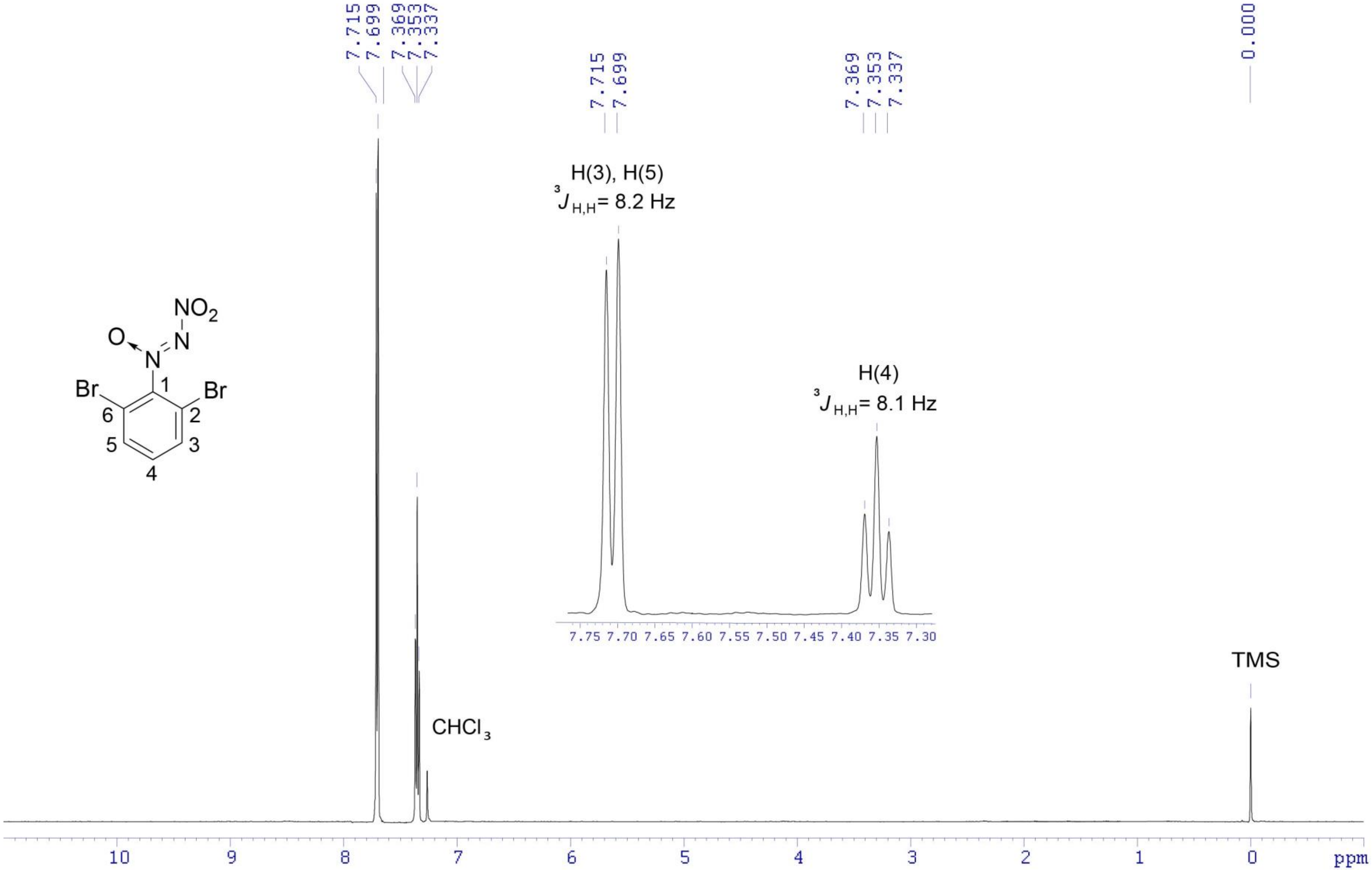

### 7.18.2 $^{13}$C NMR spectrum of compound 2s [125.76 MHz, $CDCl_3$]

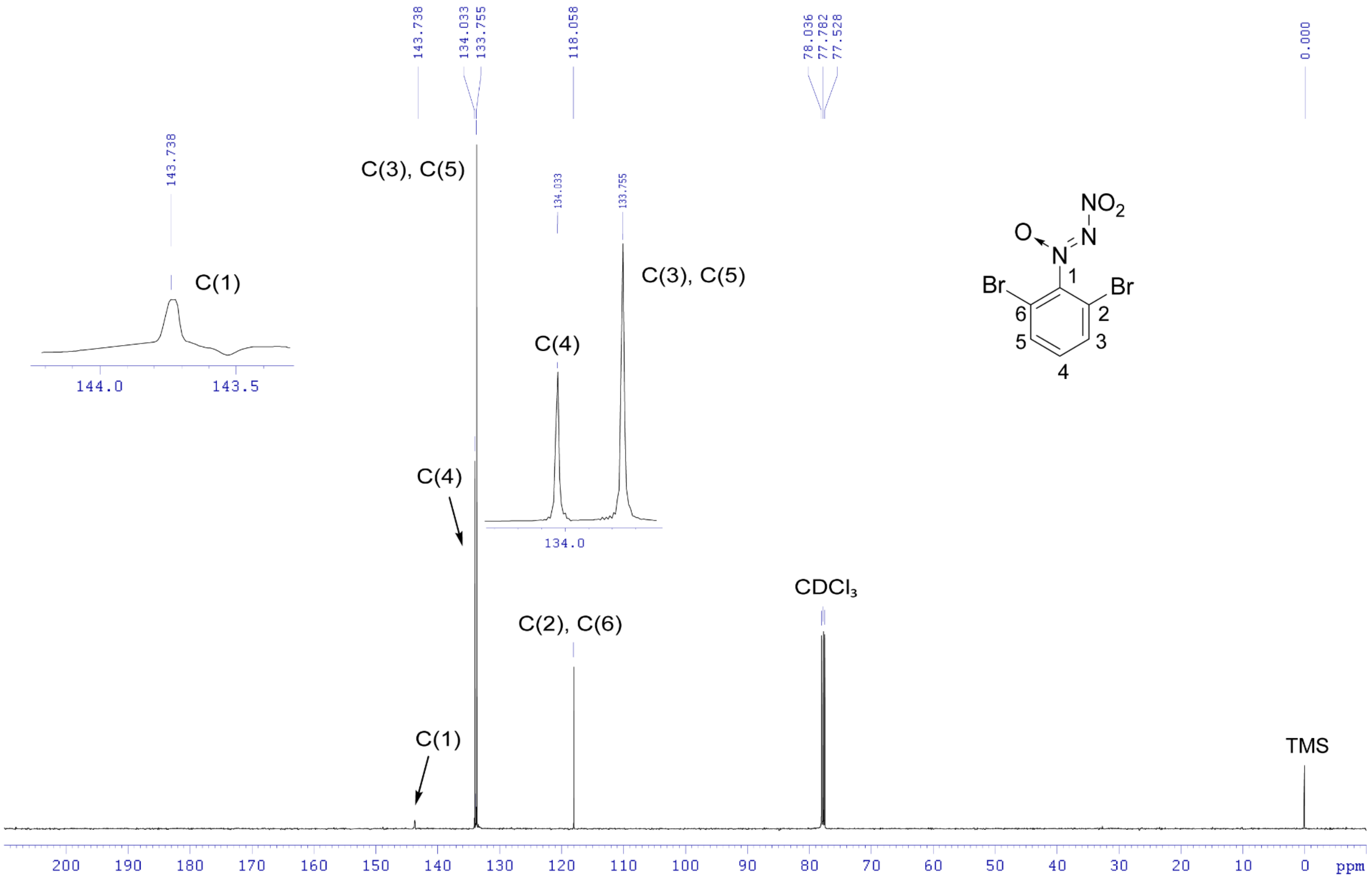

### 7.18.3 {$^{1}H$–$^{13}C$} HSQC spectrum of compound 2s [500.13 MHz, $CDCl_3$]

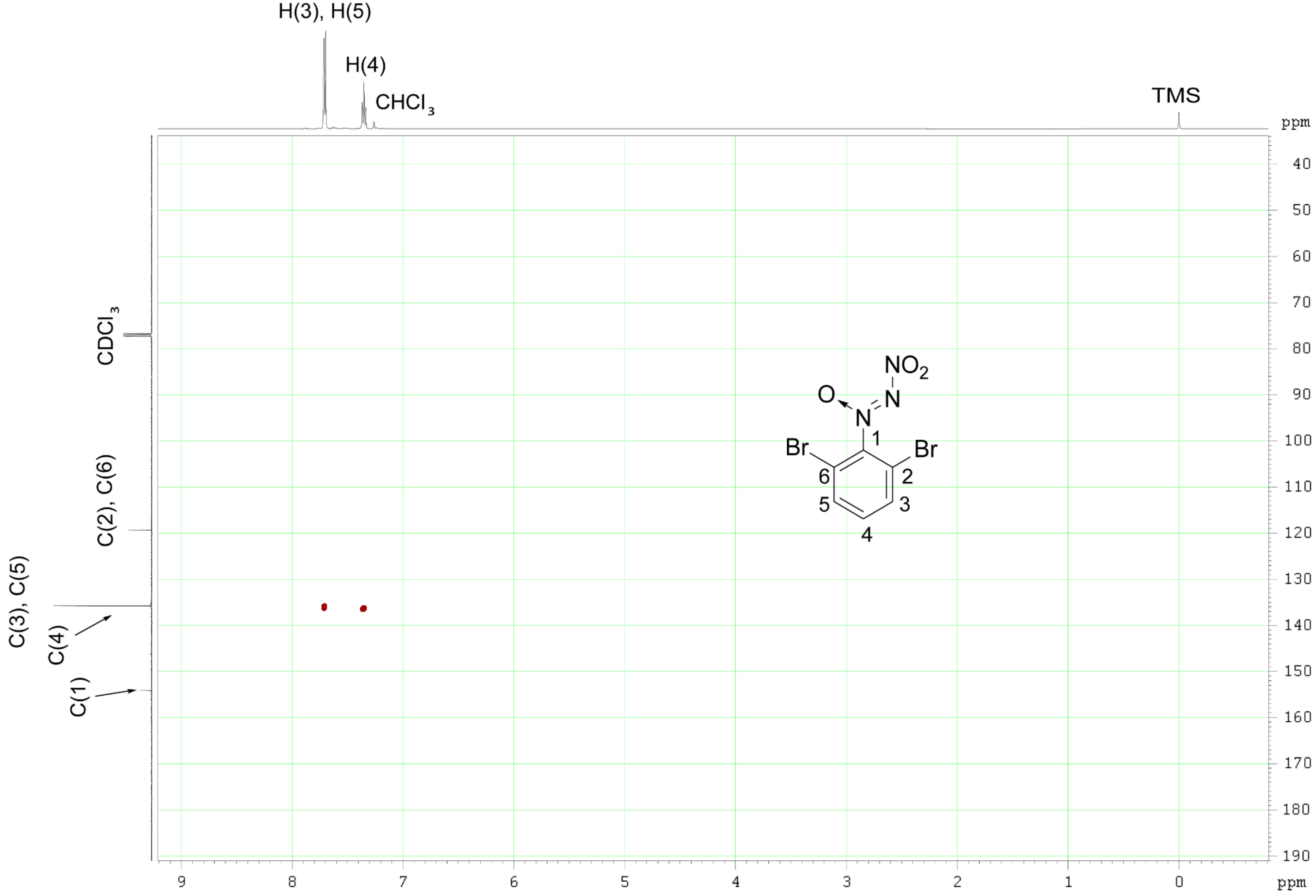

### 7.18.4 {$^1H$–$^{13}C$} HMBC spectrum of compound 2s [500.13 MHz, $CDCl_3$]

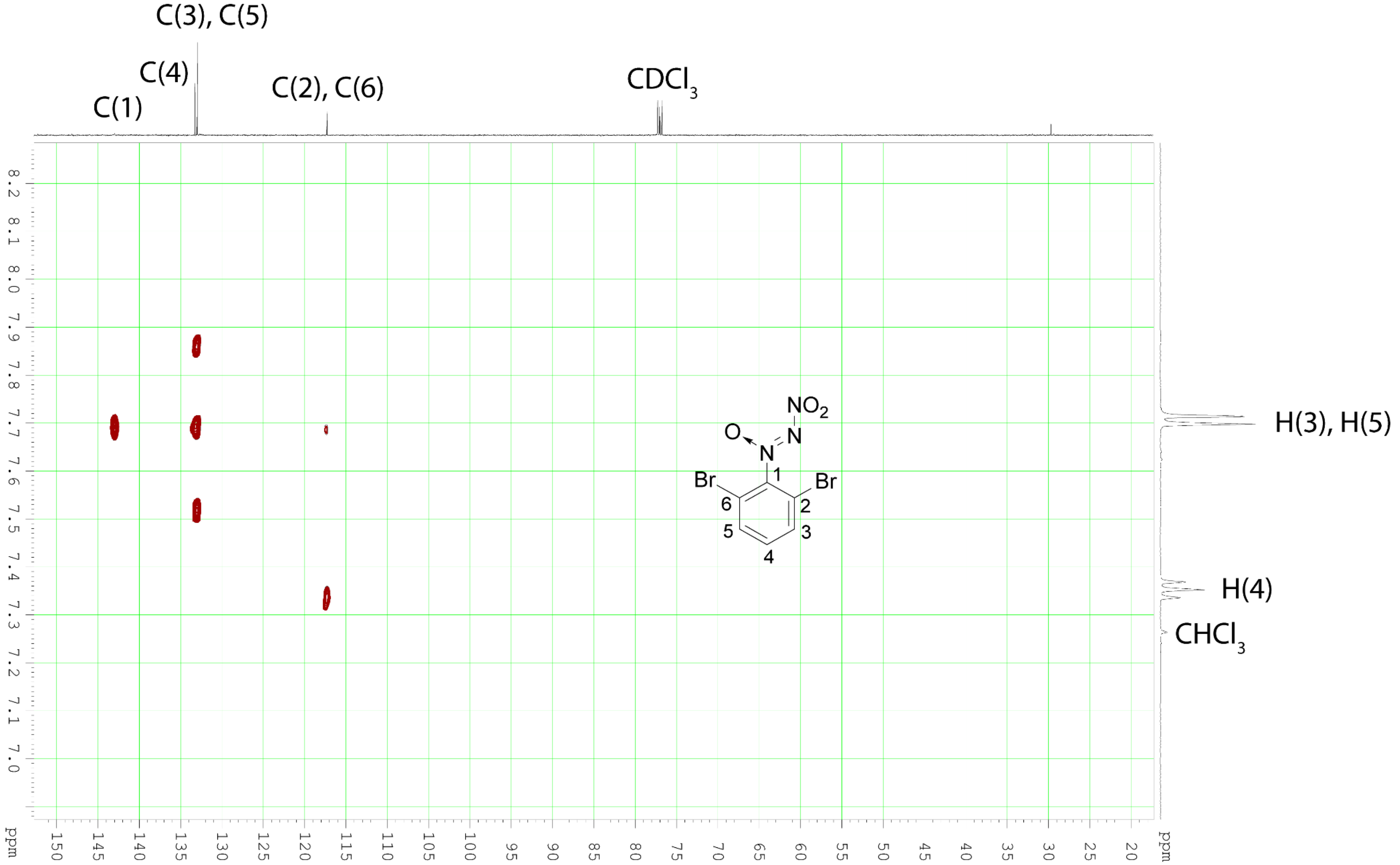

### 7.18.5 $^{14}N$ NMR spectrum of compound 2s [36.14 MHz, $CDCl_3$]

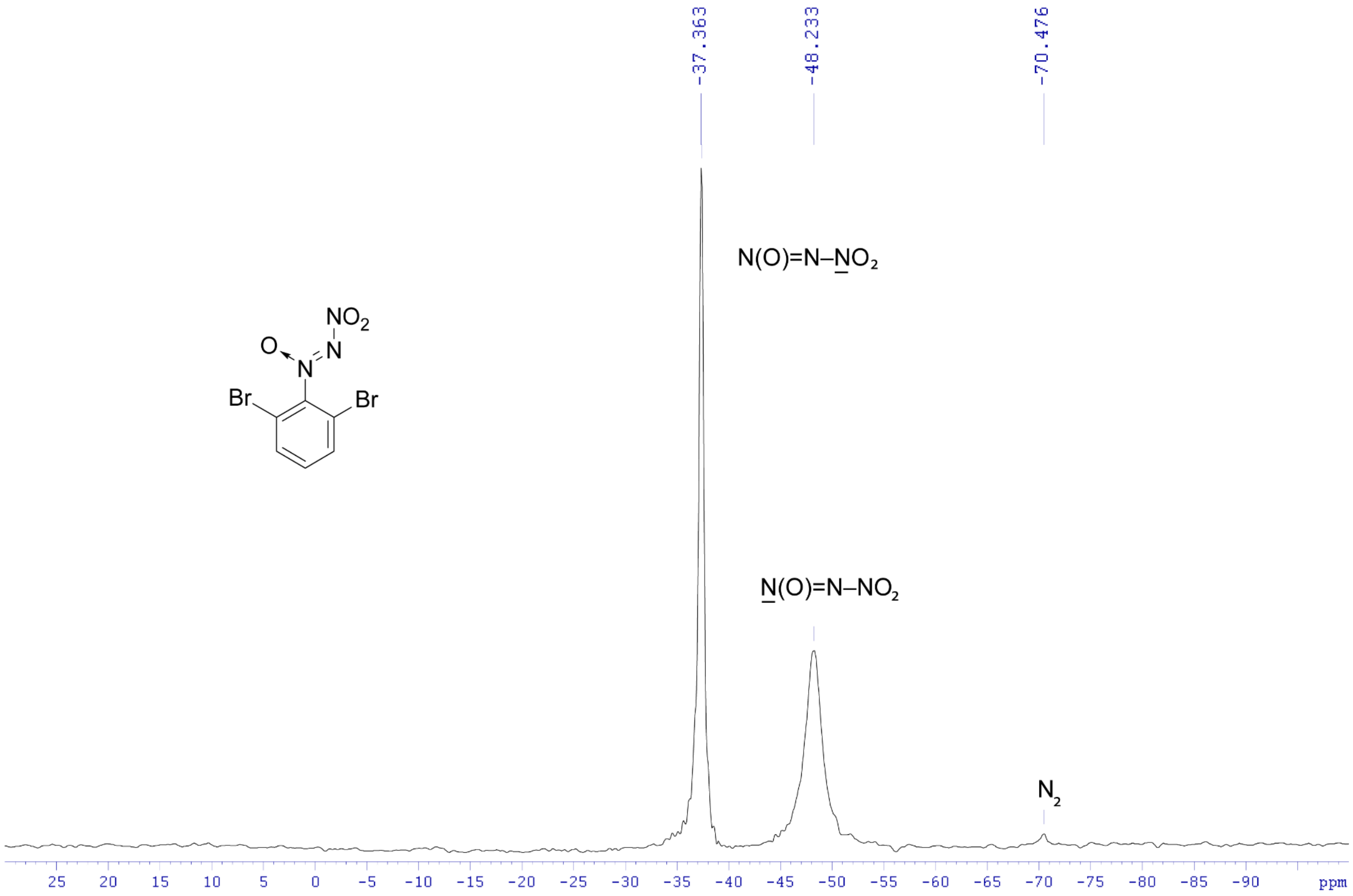

### 7.19.1 $^1$H NMR spectrum of compound 2t [500.13 MHz, $CDCl_3$]

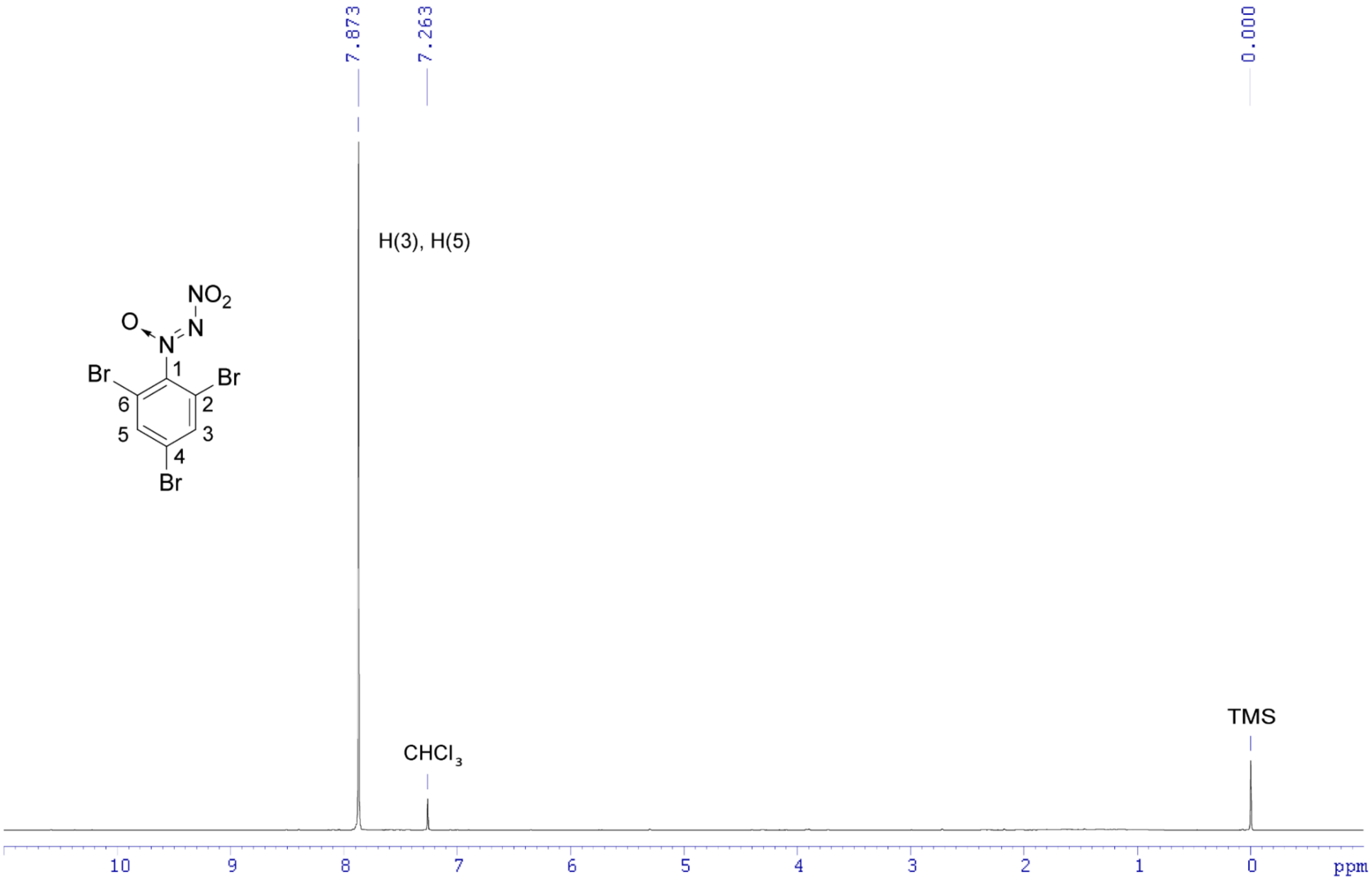

**7.19.2 $^{13}$C NMR spectrum of compound 2t [125.76 MHz, $CDCl_3$]**

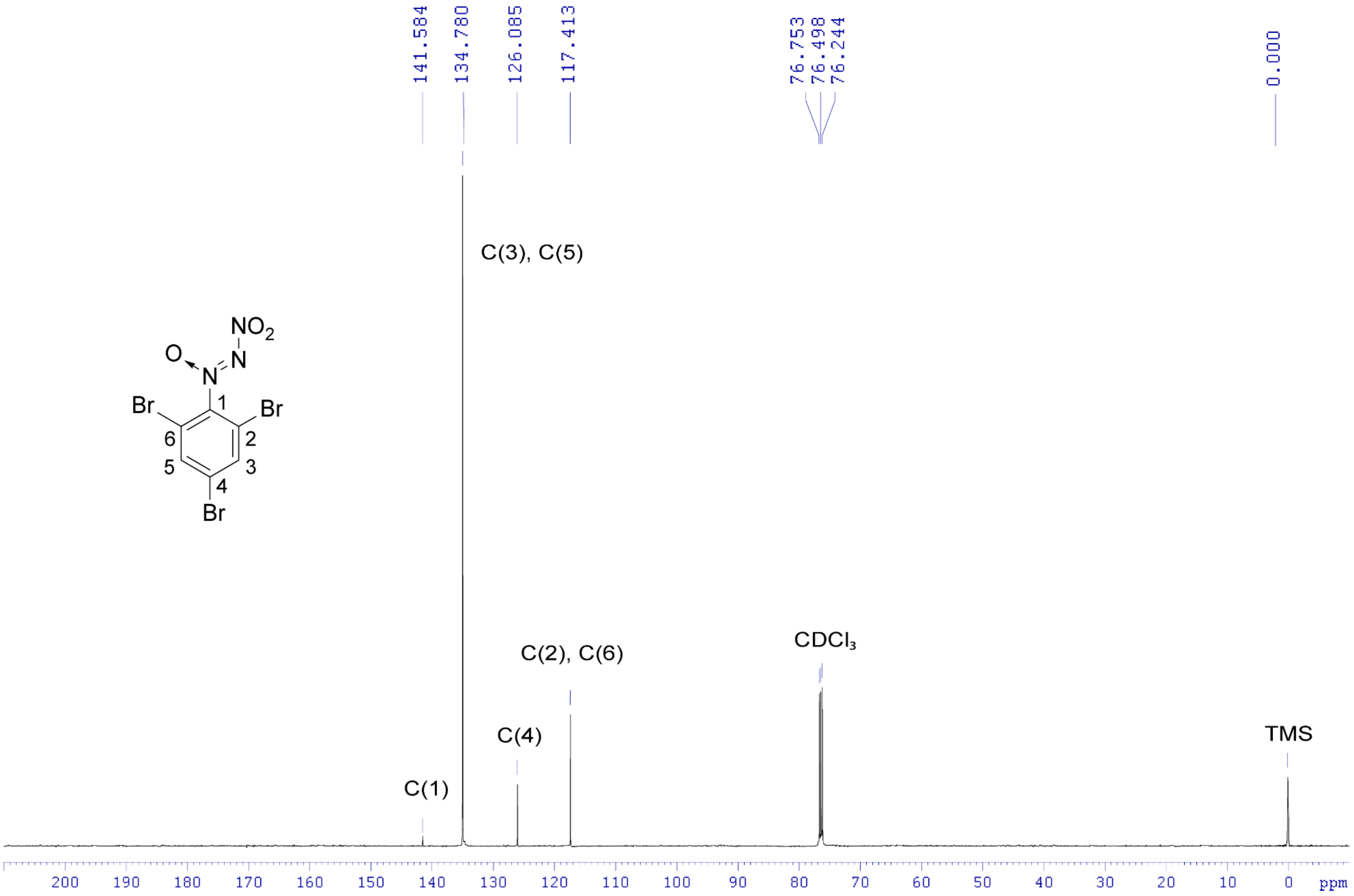

### 7.19.3 {$^1$H–$^{13}$C} HSQC spectrum of compound 2t [500.13 MHz, $CDCl_3$]

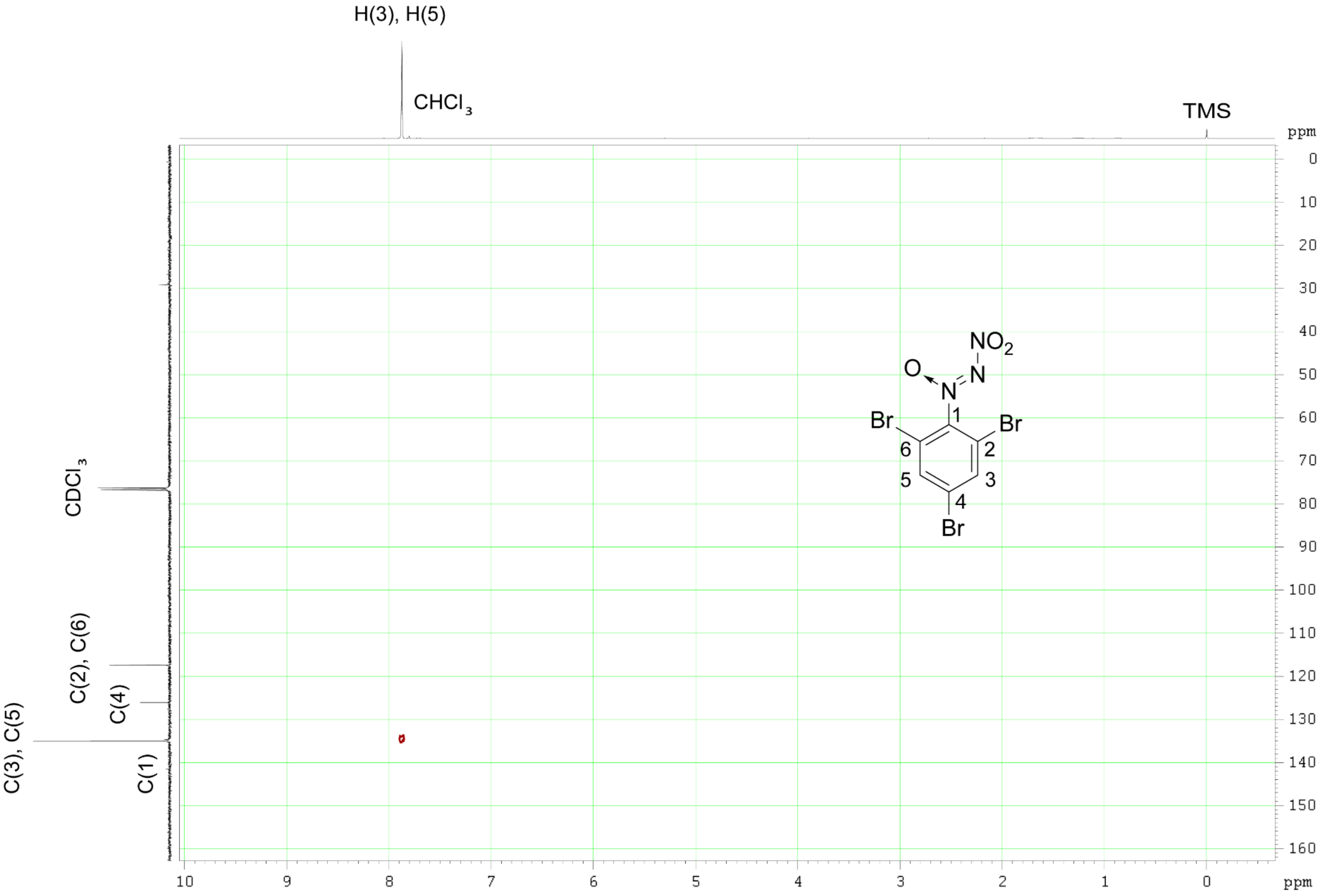

### 7.19.4 {$^{1}H$–$^{13}C$} HMBC spectrum of compound 2t [500.13 MHz, $CDCl_3$]

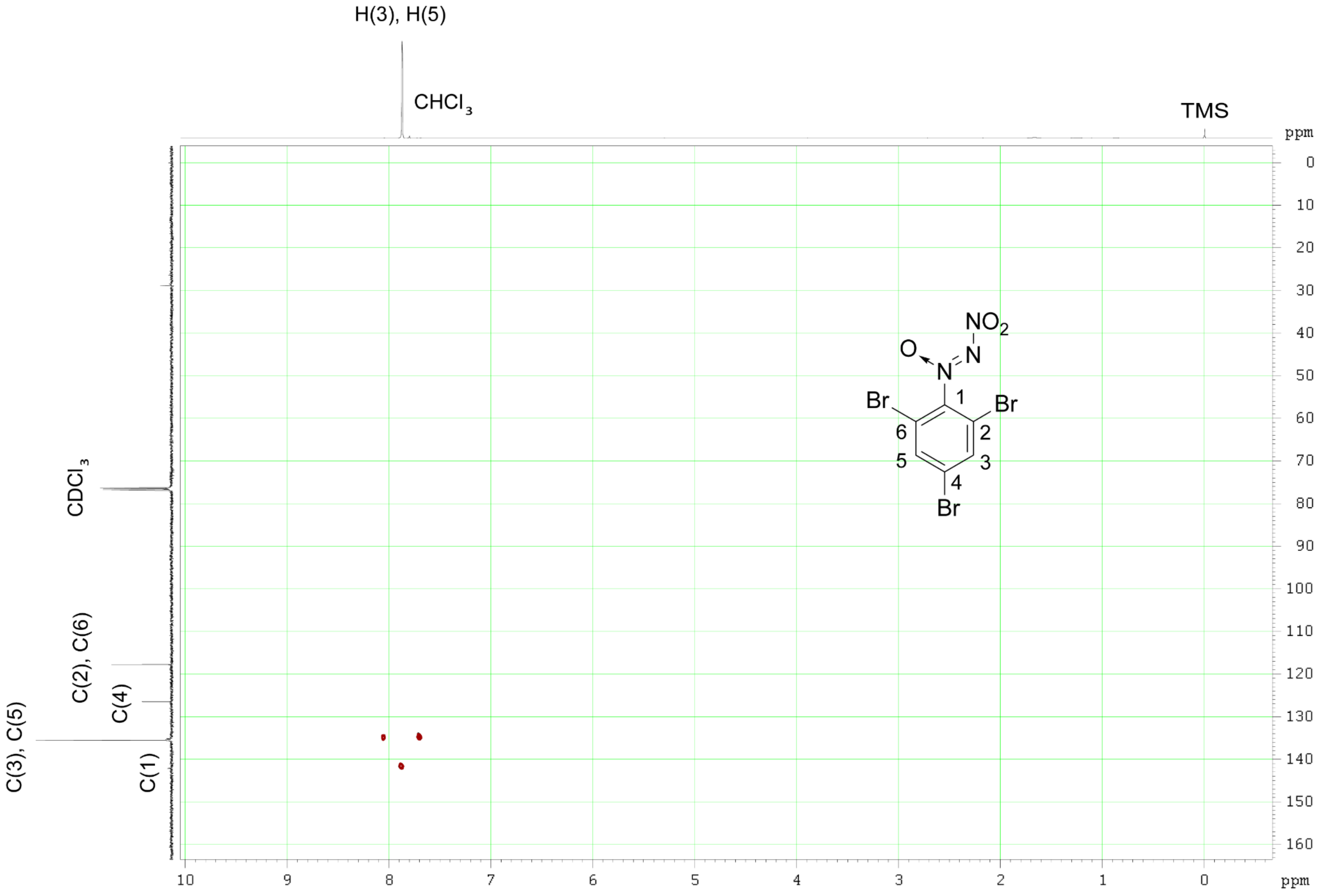

### 7.19.5 $^{14}N$ NMR spectrum of compound 2t [36.14 MHz, $CDCl_3$]

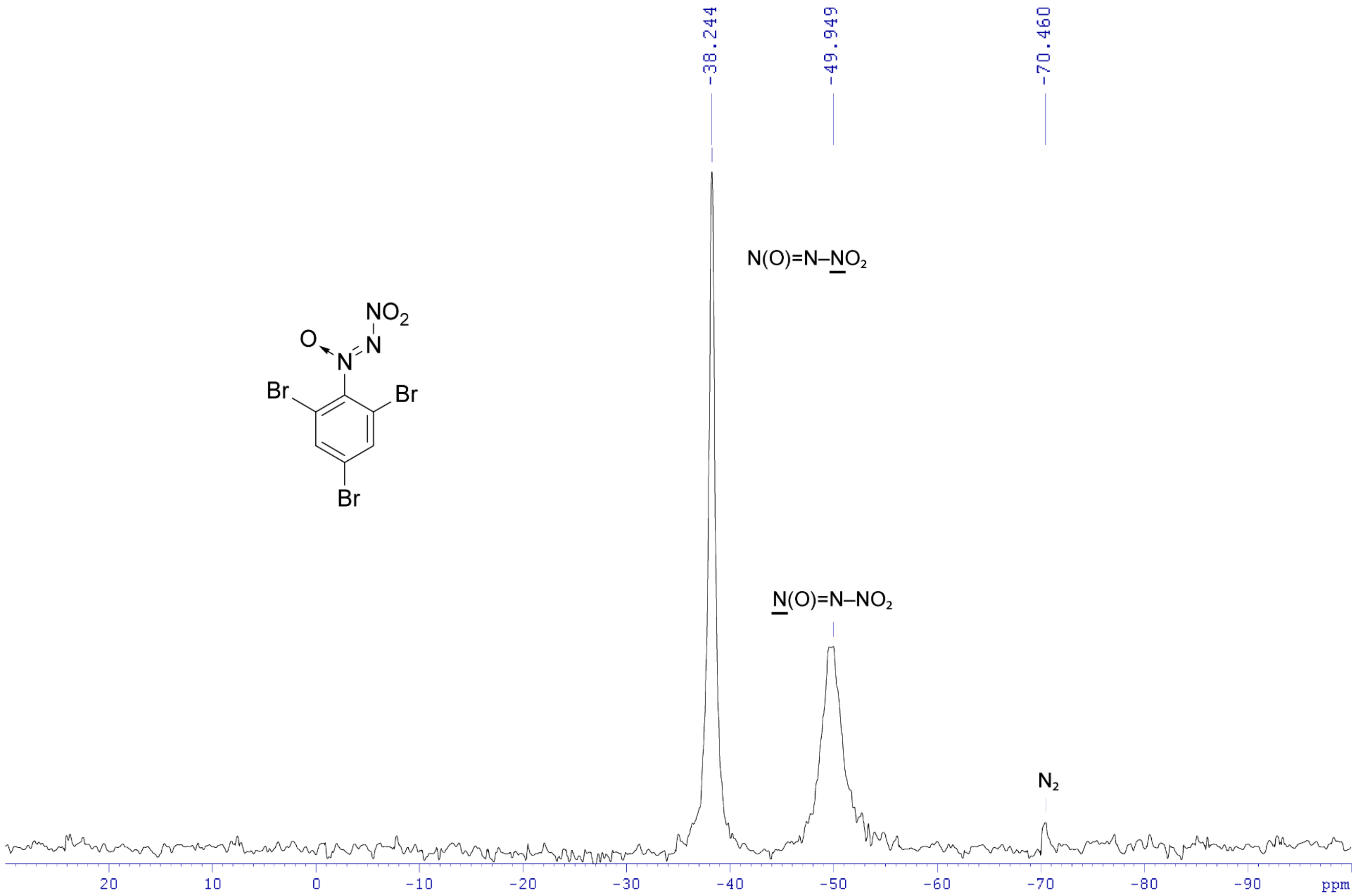

## 8. X-ray diffraction analysis

The crystal structures of compounds **2p** and **2t** were determined by single crystal X-ray diffraction analysis (Fig. S6). Bond lengths, angles and torsion angles were analyzed using the Mogul geometry tool.[25] All values are typical except for the bond distances and angles involving the nitro group. The N–$NO_2$ bonds in **2p** and **2t** (1.4751(10) and 1.478(3) Å) are significantly longer than in most of nitramines (ca. 1.35 Å). However, the longer distances are typical of several known compounds containing the nitro-*NNO*-azoxy fragment (ca. 1.39–1.53 Å, with a mean of 1.46 Å). A similar trend is found for the N–O bond lengths in nitro groups, which are shorter in **2p** and **2t** (1.2026(10), 1.204(3) and 1.1992(11), 1.206(3) Å) than in nitramides in general (1.24 Å), but typical for the nitro-*NNO*-azoxy fragment (1.20 Å).

Interestingly, the nature of the halogen type in the substituent does not affect the molecular geometry of the nitro-*NNO*-azoxy fragment, as most of the geometrical parameters are virtually the same in **2p** and **2t**. (See Table S2). An almost perpendicular orientation of the *NNO*-azoxy and the phenyl fragment is observed in both structures, which is essential to minimize the steric repulsion between the O atom and the Hal atom in the *ortho* position. On the other hand, the nitro group is perpendicular to the *NNO*-azoxy moiety to minimize the repulsion between the O atoms.

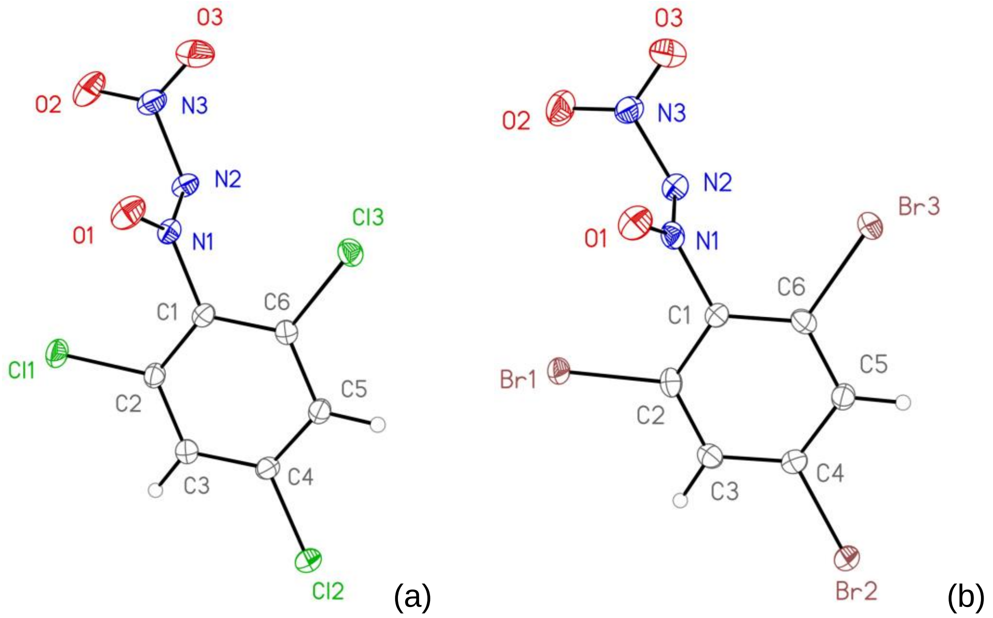


**Figure S6.** General view of the compounds **2p** (a) and **2t** (b) in their crystals; non-hydrogen atoms are represented by probability ellipsoids of atomic displacements (p = 50 %).

**Table S2.** Selected bond lengths (Å) and angles (°) in crystals **2p** and **2t**.

| | **2p** | **2t** |
|---|---|---|
| **bond lengths** | | |
| N1–C1 | 1.4556(10) | 1.466(4) |
| N1–O1 | 1.2348(9) | 1.240(3) |
| N1–N2 | 1.2824(9) | 1.279(4) |
| N2–N3 | 1.4751(10) | 1.478(3) |
| N3–O2 | 1.2026(10) | 1.204(3) |
| N3–O3 | 1.1992(11) | 1.206(3) |
| **bond angles** | | |
| O1–N1–C1 | 119.26(6) | 118.6(2) |
| O1–N1–N2 | 128.23(7) | 128.0(3) |
| N2–N1–C1 | 112.51(6) | 113.3(2) |
| N1–N2–N3 | 107.48(6) | 106.7(2) |
| O2–N3–N2 | 115.12(8) | 114.6(2) |
| O3–N3–N2 | 115.78(8) | 116.5(2) |
| O2–N3–O3 | 128.91(9) | 128.7(3) |
| **torsional angles** | | |
| O1–N1–C1–C2 | 86.02(9) | 82.8(3) |
| C1–N1–N2–N3 | -177.36(6) | –179.71(18) |
| O1–N1–N2–N3 | 1.85(11) | 1.1(3) |
| N1–N2–N3–O2 | -101.52(9) | –100.4(3) |
| N1–N2–N3–O3 | 83.06(9) | 84.6(3) |

X-ray diffraction data for compound **2p** were collected on a Bruker Smart Apex II diffractometer (equipped with Photon 2 area-detector, using φ- and ω-scan technique and graphite-monochromated MoKα-radiation), and a semi-empirical absorption correction was applied with the SADABS program.[26] X-ray diffraction data for compound **2t** were collected on a four-circle Rigaku Synergy S diffractometer (HyPix6000HE area-detector, kappa geometry, shutterless ω-scan technique, monochromated CoKα-radiation), with the gaussian-type absorption correction applied with the CrysAlisPro program[27] using the intensity data of the equivalent reflections. Structures were solved with the dual-space technique with the SHELXT program[28] and refined by a full-matrix least-squares technique on $F^2$ with anisotropic displacement parameters for non-hydrogen atoms. Hydrogen atoms were placed in calculated positions and refined in a riding model with

isotropic displacement parameters $U_{iso}$(H) equal to 1.2$U_{eq}$(C) of the connected carbon atoms. The refinement was performed using the SHELXL program.[29] Detailed crystallographic information is provided in Table S3. Full crystallographic data have been deposited at the Cambridge Crystallographic Data Center, CCDC 2384272 (for **2p**), 2384273 (for **2t**). Copies of the data can be obtained free of charge from https://www.ccdc.cam.ac.uk/structures/.

[25] Bruno, I. J., Cole, J. C., Kessler, M., Luo, J., Motherwell, W. D. S., Purkis, L. H., Smith, B. R., Taylor, R., Cooper, R. I., Harris, S. E., Orpen, A. G. Retrieval of Crystallographically-Derived Molecular Geometry Information. *J. Chem. Inf. Comput. Sci.* **2004***, 44* (6), 2133–2144. DOI: 10.1021/ci049780b

[26] Krause, L., Herbst-Irmer, R., Sheldrick, G. M., Stalke D. Comparison of silver and molybdenum microfocus X-ray sources for single-crystal structure determination. *J. Appl. Crystallogr.*, **2015**, *48* (1), 3–10. DOI: 10.1107/S1600576714022985

[27] CrysAlisPro. Version 1.171.41.106a. *Rigaku Oxford Diffraction*, **2021**.

[28] Sheldrick, G. M. SHELXT – Integrated space-group and crystal-structure determination. *Acta Crystallogr. A: Found. Adv.*, **2015**, *71* (1), 3–8. DOI: 10.1107/S2053273314026370

[29] Sheldrick, G. M. Crystal structure refinement with SHELXL. *Acta Crystallog. C: Struct. Chem.*, **2015**, *71* (1), 3–8. DOI: 10.1107/S2053229614024218

**Table S3.** Crystallographic data for crystal structures **2p** and **2t**.

| Compound | **2p** | **2t** |
|---|---|---|
| CCDC | 2384272 | 2384273 |
| Formula | $C_6H_2Cl_3N_3O_3$ | $C_6H_2Br_3N_3O_3$ |
| M, g cm$^{-3}$ | 270.46 | 403.84 |
| T, K | 120 | 100 |
| Crystal system | triclinic | monoclinic |
| Space group | *P*-1 | $P2_1/c$ |
| *Z* / *Z*' | 2 / 1 | 4 / 1 |
| *a*, Å | 7.0617(3) | 9.18854(9) |
| *b*, Å | 8.2877(3) | 12.03544(15) |
| *c*, Å | 8.4029(3) | 9.45204(9) |
| *α*, ° | 90.1273(13) | |
| *β*, ° | 93.1225(14) | 95.1932(9) |
| *γ*, ° | 101.3852(14) | |
| *V*, Å$^3$ | 481.35(3) | 1040.992(19) |
| $d_{calc}$, g cm$^{-3}$ | 1.866 | 2.577 |
| Radiation type | MoKα | CuKα |
| μ, см$^{-1}$ | 9.39 | 143.6 |
| $2\theta_{max}$, ° | 70.2 | 160.7 |
| Collected reflns. | 12199 | 13965 |
| Independent reflns. | 4190 | 2266 |
| Reflns with. I>2s(I) | 3659 | 2202 |
| $R_1$ | 0.0225 | 0.0256 |
| $wR_2$ | 0.0649 | 0.0728 |
| GOF | 1.073 | 1.106 |
| Residual density, e Å$^{-3}$($d_{max}$/$d_{min}$) | 0.527/–0.281 | 0.558/–0.778 |
